# Photocatalytic Synthesis of Polycyclic Indolones

**DOI:** 10.1002/chem.202001324

**Published:** 2020-05-14

**Authors:** Tanguy Saget, Burkhard König

**Affiliations:** ^1^ Institut de Chimie des Substances Naturelles, CNRS UPR 2301 Université Paris-Sud, Université Paris-Saclay 1, av. de la Terrasse 91198 Gif-sur-Yvette France; ^2^ Institut für Organische Chemie Universität Regensburg Universitätsstrasse 31 Regensburg Germany

**Keywords:** C−H functionalization, indoles, indolones, photoredox catalysis, visible light

## Abstract

In this work, a photocatalytic strategy for a rapid and modular access to polycyclic indolones starting from readily available indoles is reported. This strategy relies on the use of redox‐active esters in combination with an iridium‐based photocatalyst under visible‐light irradiation. The generation of alkyl radicals through decarboxylative single electron reductions enables intramolecular homolytic aromatic substitutions with a pending indole moiety to afford pyrrolo‐ and pyridoindolone derivatives under mild conditions. Furthermore, it was demonstrated that these radicals could also be engaged into cascades consisting of an intermolecular Giese‐type addition followed by an intramolecular homolytic aromatic substitution to rapidly assemble valuable azepinoindolones.

## Graphical abstract

**A variety of indoles** were readily transformed into valuable polycyclic indolones by using a photocatalytic C−H alkylation strategy. The use of redox‐active esters in combination with a reducing photocatalyst enabled the smooth generation of alkyl radicals that could undergo an intramolecular cyclization through a homolytic nucleophilic substitution pathway. The obtained indolones are useful synthetic intermediates for the synthesis of bioactive compounds.

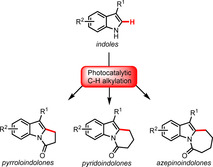

Indoles are prevalent motifs in bioactive natural products and pharmaceuticals.^1^ Therefore, the development of methods for the synthesis of functionalized of indoles under mild conditions is an important task in synthetic chemistry.^2^ In this respect, catalytic transformations enabling the direct functionalization of indole C−H bonds are particularly valuable because they afford complex indole structures with an excellent step and atom economy.^3^ We report herein a catalytic access to diverse polycyclic indolones starting from cheap and readily available indole precursors (Scheme 1). Importantly, such indolone motifs are found in a range of indole alkaloids^4^ and are valuable intermediates in the total synthesis of related natural products.^5^

**Scheme 1:**
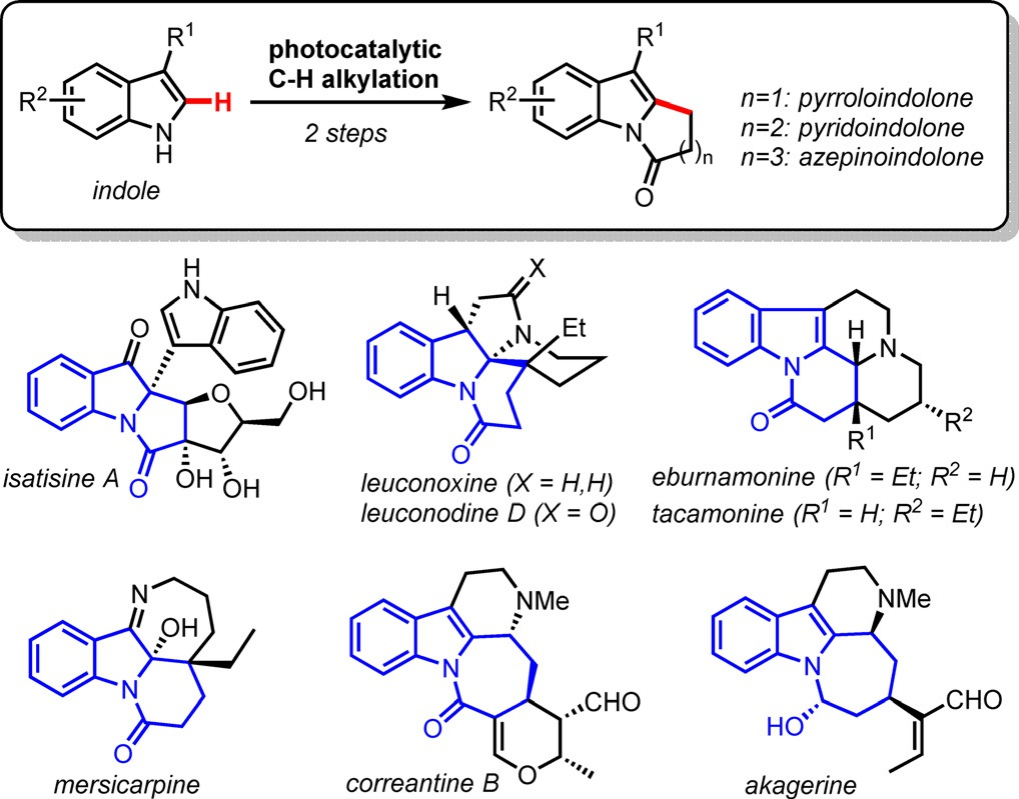
A photocatalytic strategy to access valuable polycyclic indolones.

Over the last decade, photoredox catalysis has emerged as a powerful tool for organic synthesis allowing for the generation of reactive free radical species under mild conditions and from simple precursors.^6^ Notably, photoredox catalysis can be an efficient tool for indole functionalization.^7^ Redox‐active esters, such as *N*‐acyloxyphthalimides (NAPs), are versatile precursors of alkyl radicals through single‐electron reduction followed by decarboxylation.^8^ In particular, NAPs have been used in photocatalytic Minisci‐type reactions to generate nucleophilic alkyl radicals which reacts with electron deficient heterocycles such as pyridines or (iso)quinolines.^9^ However, NAPs have rarely been applied to the functionalization of electron rich heterocycles like indoles.^10^ We reasoned that an intramolecular cyclization could overcome the mismatch polarity of radicals with a nucleophilic character reacting with electron‐rich aromatics.

To this purpose, we studied the use of NAPs **2** derived from carboxylic acids obtained from the reaction of indoles and commercially available cyclic anhydrides (Scheme 2 a). We expected these NAPs to undergo a single‐electron transfer with an excited reducing photocatalyst leading to alkyl radical **3** after fragmentation followed by decarboxylation. Radical **3** would then undergo a 5‐*exo*‐*trig* cyclization leading to dearomatized intermediate **4** which after oxidation and proton elimination would afford indolone product **6** (Scheme 2 b).

**Scheme 2:**
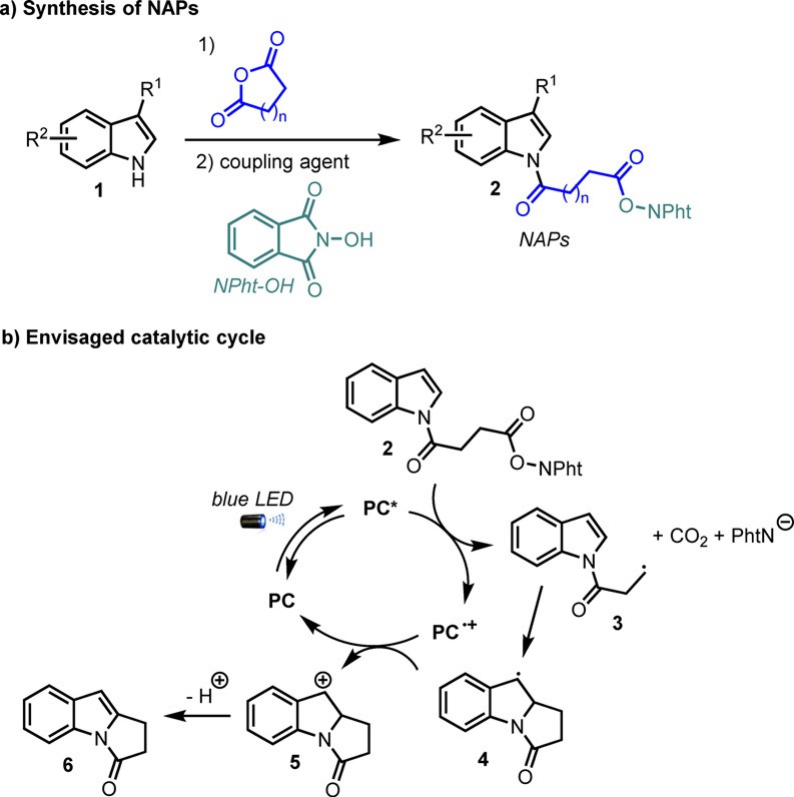
Catalytic cycle of the envisaged strategy.

We studied the feasibility of the envisioned process with substrate **2 a**, readily accessed in two steps from indole and succinic anhydride. We first evaluated the use of organic dyes as photocatalysts.^11^ When **2 a** was reacted with 5 mol % of commonly used 4‐CzIPN^12^ (**PC1**, *E* ^*red*^=−1.04 V vs. SCE) in DMSO under blue‐light irradiation, a small amount of **6 a** could be detected but most of the crude mixture consisted of unreacted starting material (Table 1, entry 1). Given the highly negative reduction potential of NAPs (*E* ^*red*^=−1.3 V vs. SCE), we reasoned that a more reducing photocatalyst would facilitate a photoinduced electron transfer (PET) to the substrate, thus increasing the conversion of **2 a**. To this purpose, we performed the reaction in the presence of **PC2**, a highly reducing phenoxazine photocatalyst recently developed by Miyake and co‐workers (*E* ^red*^=−1.93 V vs. SCE).^13^ Pleasingly, the yield of **6 a** significantly increased to 58 % (entry 2). Based on these results, we then further evaluated *fac*‐Ir(ppy)_3_ (*E* ^red*^=−1.73 V vs. SCE) which proved to be a very efficient photocatalyst for the targeted transformation leading to **6 a** in 67 % isolated yield (entry 3). The use of other common solvents, such as dimethylacetamide (DMA) or DMF, was detrimental (entries 4 and 5). Of note, the presence of up to ten equivalents of water does not affect the yield of the reaction so technical grade DMSO could be used as solvent for this study (entry 6). Furthermore, a control experiment revealed that the photocatalyst is required to observe the desired reactivity (entry 7). Finally, the use of a reduced catalyst loading (0.5 mol %) led to a similar yield after 14 h (entry 8).

**Table 1:** Optimization of the decarboxylative cyclization.^[a]^

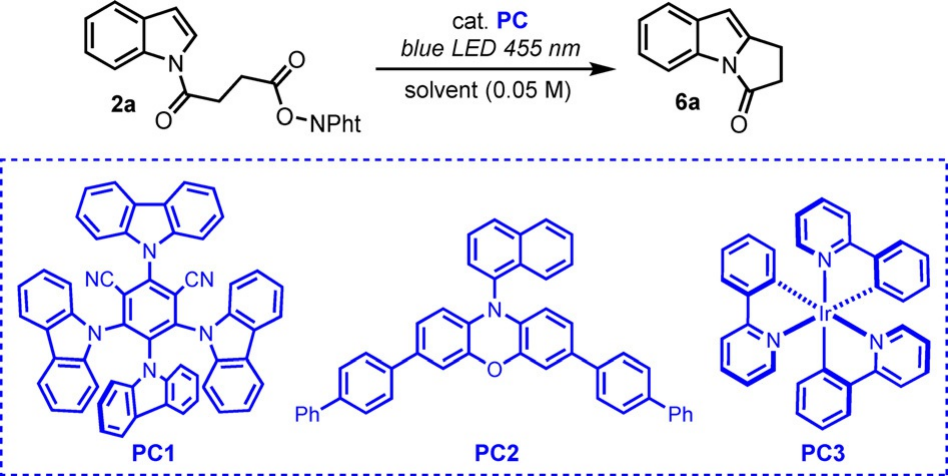			
Entry	PC	Solvent	Yield^[b]^
1	**PC1** (5 mol %)	DMSO	12 %
2	**PC2** (5 mol %)	DMSO	58 %
3	**PC3** (1 mol %)	DMSO	87 % [67 %]^[c]^
4	**PC3** (1 mol %)	DMA	70 %
5	**PC3** (1 mol %)	DMF	55 %
6	**PC3** (1 mol %)	DMSO (10 equiv H_2_O)	88 %
7	**–**	DMSO	<3 %
8	**PC3** (0.5 mol %)	DMSO	88 %

[a] General conditions: **2 a** (0.1 mmol) and **PC** in 2 mL of solvent (0.05 m) under a N_2_ atmosphere with 455 nm light irradiation for 14 h. [b] Yield determined by GC‐FID with an internal standard. [c] Isolated yield on a 0.25 mmol scale. DMSO=dimethylsulfoxide; DMA=dimethylacetamide; DMF=dimethylformamide.

With optimal conditions in hand to promote the desired cyclization, we developed a more efficient one‐pot protocol enabling the synthesis of indolone **6 a** starting directly from carboxylic acid **7 a**. To this purpose, we investigated the use of coupling agents, such as dicyclohexylcarbodiimide (DCC) and diisopropylcarbodiimide (DIC) (Table 2, entry 1–2). Pleasingly, the use of DIC led to a similar yield when compared to our previously optimized two‐step protocol (entry 2). The low yield obtained with DCC may be due to the formation of a poorly soluble dicyclohexylurea byproduct that might prevent a sufficient light penetration into the reaction medium. Notably, the addition of a catalytic amount of DMAP for the coupling was detrimental to the overall process (entry 3).

**Table 2:** Optimization of a one‐pot protocol starting from **7 a**.^[a]^

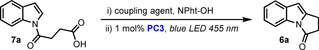			
Entry	Coupling agent	Additive	Isolated yield
1	DCC	**–**	25 %
2	DIC	**–**	66 %
3	DIC	DMAP (10 mol %)	53 %

[a] General conditions: i) **7 a** (0.25 mmol), NPht‐OH (0.25 mmol) and coupling agent (0.25 mmol) in THF (0.2 m) for 16 h; ii) *fac*‐Ir(ppy)_3_ (1 mol %) in 5 mL of DMSO (0.05 m) under a N_2_ atmosphere with 455 nm light irradiation for 8 h. NPht‐OH = *N*‐hydroxyphtalimide; DCC = dicyclohexylcarbodiimide; DIC = diisopropylcarbodiimide; DMAP = 4‐dimethylaminopyridine.

The scope of the reaction was then evaluated with a range of different anhydrides and indole derivatives (Scheme 3). Substrates derived from several succinic anhydrides and leading to the formation of primary, secondary and tertiary radicals afforded the desired pyrroloindolones **6 a**–**d** in good overall yields. Importantly, compounds **6 b** and **6 c** were obtained as single diastereoisomers. Pleasingly, substrates **7 e**–**g** derived from glutaric anhydrides also led to the formation of pyridoindolones through a cyclization step which then occurs through a 6‐*exo*‐*trig* addition. Then, a variety of indoles with different substitution patterns were also evaluated for this process. Substrates bearing both electron‐withdrawing and electron‐donating groups were successfully implemented in our methodology as shown with indolones **6 h**–**v**. The use of chlorinated and brominated indoles led to the desired indolones **6 m**–**o** uneventfully and allow for further modifications through cross‐coupling reactions. A pyrrole‐derived substrate was also competent for this process as shown with **6 p**. Finally, a range of 3‐substituted indoles could be used to access indolones **6 q**–**v**. Notably, several complex substrates derived from tryptamine, melatonine and tryptophan were successfully transformed into valuable indolones **6 t**–**v** in good yields. The structure of **6 v** was unambiguously confirmed by X‐ray crystallographic analysis.^14^

**Scheme 3:**
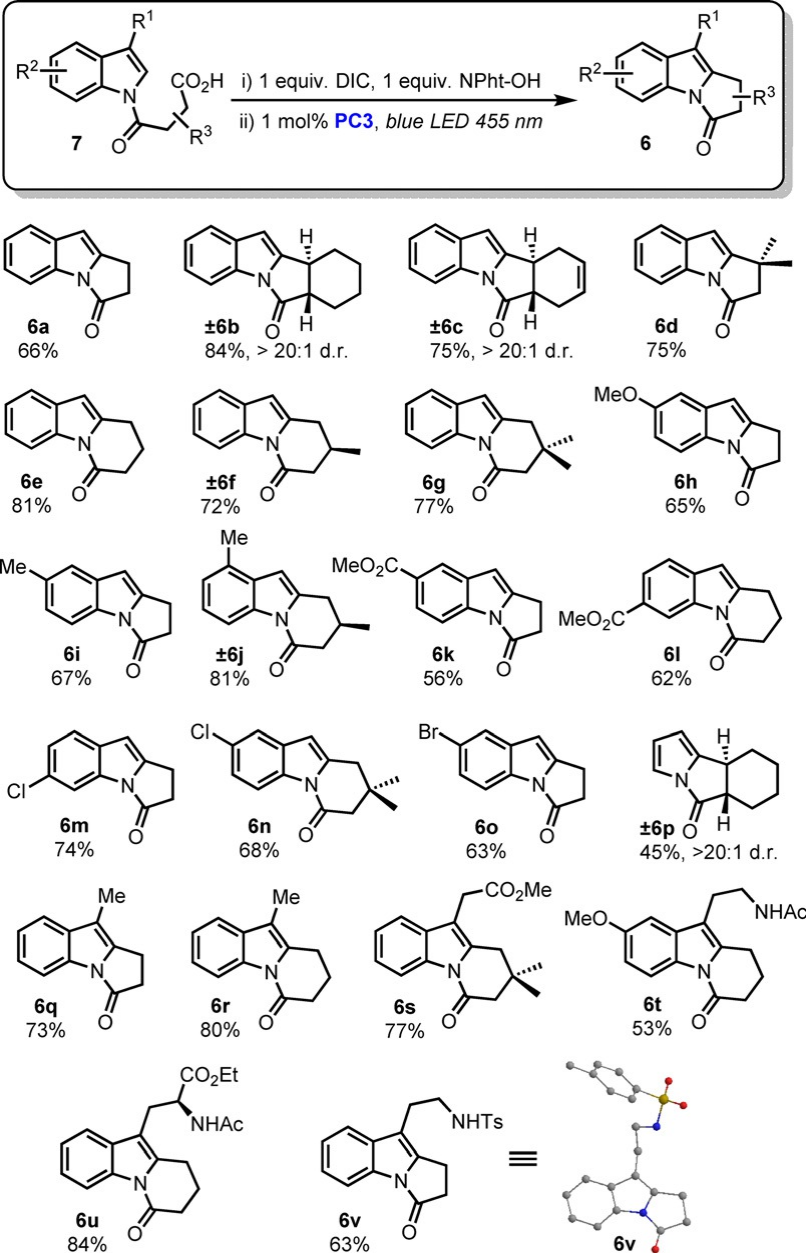
Scope of the reaction.

To showcase the scalability of the process, we performed a gram‐scale reaction using 4.25 mmol of **2 q** and a reduced catalyst loading of only 0.2 mol % without impacting the outcome of the reaction (Scheme 4). Then, to further demonstrate the utility of this method we performed some transformations on compounds **6** to prove their versatility as synthetic intermediates. First, **6 q** was reduced with borane to access in a single step the pyrroloindole scaffold (see **8**) which is found is many bioactive compounds,^15^ including the flinderole alkaloids^16^ and many pharmaceutically relevant small molecules.^17^ Importantly, **6 q** could also be selectively hydrogenated with a catalytic amount of palladium on charcoal to access the important indoline scaffold quantitatively (see **10**). Then, the indolone moiety was also reacted with soft nucleophiles to afford C2‐alkylated free indoles as exemplified with compound **9**. Finally, electrophilic bromination of compound **6 v** led to complex pyrroloindoline **11**, which is reminiscent of many naturally occurring alkaloids exhibiting a diverse range of biological activities.^18^

**Scheme 4:**
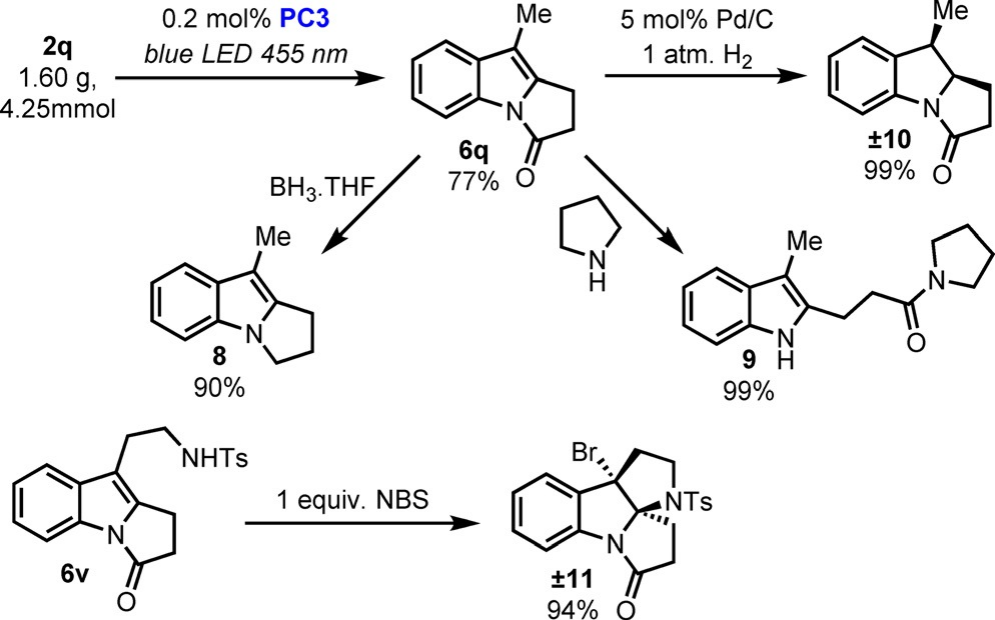
Gram‐scale reaction and synthetic applications.

The commercial availability of many succinic and glutaric anhydrides enabled us to efficiently synthesize a range of pyrrolo‐ and pyridoindolones by using our methodology. However, the scarce availability of adipic anhydrides, prevented us to access the valuable azepinoindolone scaffold.4a, 4b, 19 To circumvent this issue, we envisaged to intercept radical **3** with an external olefin to access radical **12** which would then add to the indole moiety to afford azepinoindolone **13** as described in Scheme 5 a. As an inherent challenge to this strategy, the intermolecular Giese‐type addition to the olefin must be kinetically favored over the intramolecular 5‐*exo*‐*trig* cyclization to the indole. After some experimentation, we discovered that the use of acrylonitrile as a trapping olefin efficiently led to the desired azepinoindolones, whereas only traces of the corresponding pyrroloindolones could be detected.^20^ This strategy allowed us to access valuable azepinoindolones **13 a**–**f** in moderate‐to‐good yields (Scheme 5 b).

**Scheme 5:**
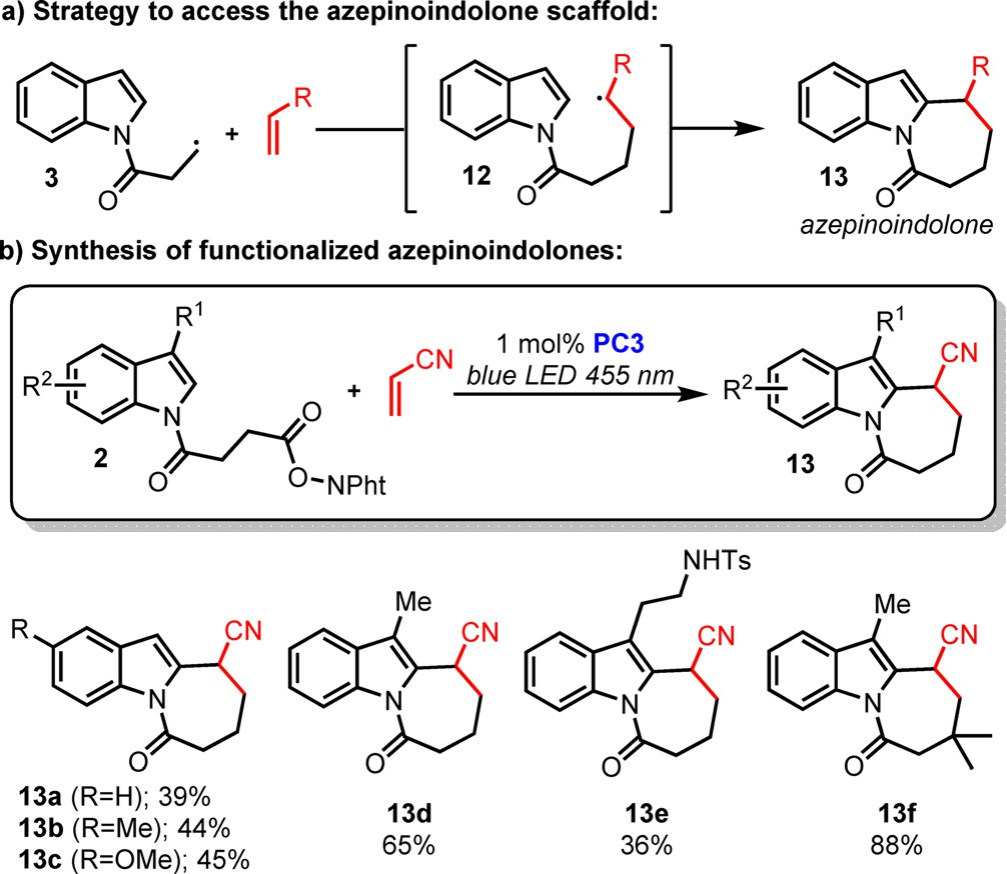
Synthesis of azepinoindolones.

In summary, we have developed a photocatalytic C−H alkylation strategy mediated by visible light that provides an efficient access to a variety of relevant polycyclic indolones. The reaction is scalable and the indolone products can be further used as valuable synthetic intermediates to access other important scaffolds, such as pyrroloindoles and (pyrrolo)indolines. Finally, the development of a challenging two‐component process enabled the straightforward synthesis of functionalized azepinoindolones. We expect this methodology to find a widespread use in the synthesis of indole‐containing natural products and bioactive compounds.

## Conflict of interest

The authors declare no conflict of interest.

## Supporting information

As a service to our authors and readers, this journal provides supporting information supplied by the authors. Such materials are peer reviewed and may be re‐organized for online delivery, but are not copy‐edited or typeset. Technical support issues arising from supporting information (other than missing files) should be addressed to the authors.

Supplementary
