# Supplementary material for: Photocatalytic Synthesis of Polycyclic Indolones
Source: Chemistry. 2020 May 14;26(31):7004–7. doi: 10.1002/chem.202001324 (PMC7317559; doi:10.1002/chem.202001324)
Supplement: Supplementary file 1 — Supplementary [file CHEM-26-7004-s001.pdf]

Chemistry–A European Journal

Supporting Information

Photocatalytic Synthesis of Polycyclic Indolones

Tanguy Saget*[a, b] and Burkhard König*[b]

Supporting Information on

Photocatalytic Synthesis of Polycyclic Indolones

Tanguy Saget*[a,b] and Burkhard König*[b].

[a] Institut de Chimie des Substances Naturelles, CNRS UPR 2301, Univ. Paris-Sud, Université Paris-Saclay, 1, av. de la Terrasse, 91198 Gif-sur-Yvette, France.

[b] Institute of Organic Chemistry, Faculty of Chemistry and Pharmacy, University of Regensburg, Universitätsstraße 31, 93053 Regensburg, Germany.

Table of Contents

General methods 2

Experimental section 3

Spectra 40

General methods

Starting materials and reagents were purchased from commercial suppliers (Sigma Aldrich, Alfa Ae-sar, Acros, Fluka, TCI or VWR) and used without further purification. Solvents were used as p.a. grade or dried and distilled according to literature known procedures. Liquids were added via syringe, needle and septum techniques unless otherwise stated.
All NMR spectra were measured at room temperature using a Bruker Avance 300 (300 MHz for 1H, 75 MHz for 13C) NMR spectrometer. All chemical shifts are reported in δ scale as parts per million [ppm] (multiplicity, coupling constant J, number of protons) relative to the solvent residual peaks as the internal standard. Coupling constants J are given in Hertz [Hz]. Abbreviations used for signal multiplicity: 1H-NMR: b = broad, s = singlet, d = doublet, t = triplet, q = quartet, p = pentet, hept = heptet, dd = doublet of doublets, dt = doublet of triplets, dq = doublet of quartets, and m = multiplet.
The mass spectrometrical measurements were performed at the Central Analytical Laboratory of the University of Regensburg. All mass spectra were recorded on a Finnigan MAT 95, ThermoQuest Fin-nigan TSQ 7000, Finnigan MAT SSQ 710 A or an Agilent Q TOF 6540 UHD instrument.
For the optimization using aldehydes following GC method was used: GC measurements were per-formed on a GC 6890 from Agilent Technologies. Data acquisition and evaluation was done with Agilent ChemStation Rev.C.01.04. A capillary column DB–WAX UI/30 m x 0.25 mm/0.25 µM film and helium as carrier gas (flow rate of 1 mL/min) were used. The injector temperature (split injection: 30:1 split) was 280 °C, detection temperature 310 °C (FID). GC measurements were made and inves-tigated via integration of the signal obtained. The GC oven temperature program was adjusted as fol-lows: initial temperature 40 °C was kept for 3 minutes, the temperature was increased at a rate of 15 °C/min over a period of 12 minutes until 220 °C was reached and kept for 5 minutes, the tempera-ture was again increased at a rate of 25 °C/min over a period of 48 seconds until the final temperature (240 °C) was reached and kept for 5 minutes. trimethoxybenzene was used as an internal standard.
Analytical TLC was performed on silica gel coated alumina plates (MN TLC sheets ALUGRAM® Xtra SIL G/UV254). Visualization was done by UV light (254 nm). If necessary, potassium permanganate, vanillin or ceric ammonium molybdate was used for chemical staining.
Purification by column chromatography was performed with silica gel 60 M (40 63 µm, 230-440 mesh, Merck) on a Biotage® IsoleraTM Spektra One device. For irradiation with blue light OSRAM Oslon SSL 80 LDCQ7P-1U3U (blue, λmax = 455 nm, Imax = 1000 mA, 1.12 W) was used.
For irradiation with green light Cree XPEGRN L1 G4 Q4 (green, λmax = 535 nm, Imax = 1000 mA, 1.12 W) was used.
Flash chromatography was performed on silica gel (230-400 mesh).

Experimental section

General Procedure (GP1) for the synthesis of carboxylic acids 7:

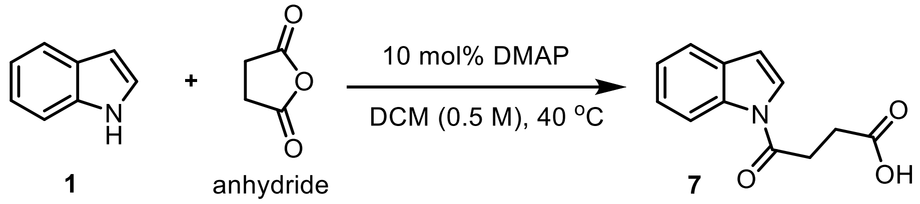

Indole **1** (1.0 equiv.), cyclic anhydride (1.5 equiv.) and DMAP (10 mol%) were weighed in a vial equipped with a magnetic stirrer bar. The vial was sealed. DCM (0.5 M) and NEt_3 (3 equiv.) were introduced and the resulting solution was stirred for 18-20 hours at 40 °C. The reaction was cooled down to room temperature and diluted with AcOEt. The organic layer was washed with 1M aqueous HCl (x3) then brine. The organic layer was dried over Na_2SO_4 and concentrated *in vacuo*. Purification by column chromatography on silica gel (eluent: DCM/AcOEt 100:0 to 50:50) afforded **7**.

General Procedure (GP2) for the synthesis of NAPs 2:

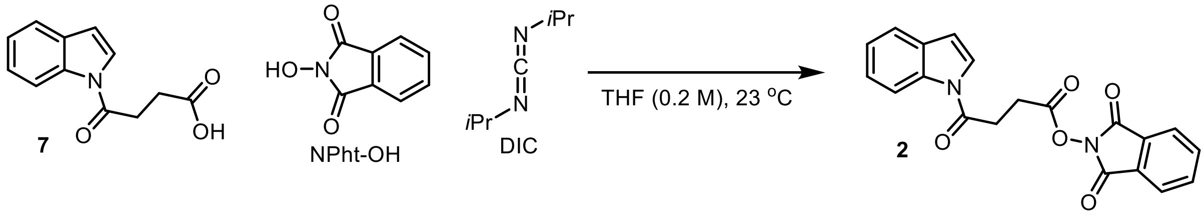

Carboxylic acid **7** (1.0 equiv.) and NPht-OH (1.0 equiv.) were weighed in a vial equipped with a magnetic stirrer bar. The vial was sealed. THF (0.2 M) was introduced and then DIC (1 equiv.) was added and the resulting solution was stirred for 16-20 hours at 23 °C. The solvent was removed *in vacuo*. Purification by column chromatography on silica gel (eluent: DCM) afforded **2**.

General Procedure (GP3) for the synthesis of indolone 6:

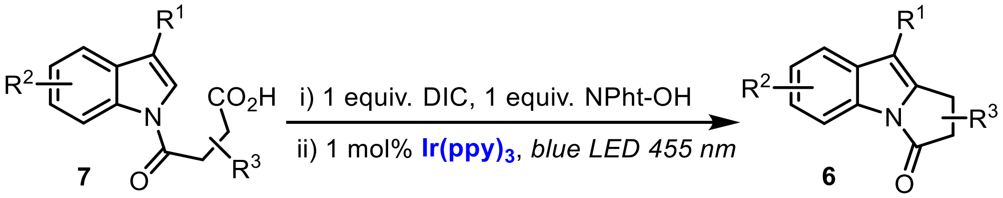

Carboxylic acid **7** (250 µmol, 1.0 equiv.) and NPht-OH (40.8 mg, 1.0 equiv.) were weighed in a vial equipped with a magnetic stirrer bar. The vial was sealed. THF (2 mL) was introduced and then DIC (39 µL, 1.0 equiv.) was added and the resulting solution was stirred for 16 hours at 23 °C. The solvent was removed *in vacuo*. Photocatalyst $Ir(ppy)_3$ (1,6 mg, 1.0 mol%) was

added to the vial. Reagent grade DMSO (5 mL, 0.05 M) was introduced. The reaction mixture was put under vacuum (4-5 mbar) and then backfilled with nitrogen (5 times). The reaction was stirred at 23°C under blue light irradiation (455 nm) for 8 hours. The reaction mixture was diluted in 25 mL of Et_2O and washed with 25 mL of water (x2), 2M NaOH solution (x2), then brine. The organic layer was dried over Na_2SO_4 and concentrated *in vacuo*. Purification by column chromatography on silica gel (eluent: petroleum ether/DCM 70/30 to 0/100) afforded **6**.

General Procedure (GP4) for the synthesis of azepinoindolone 13:

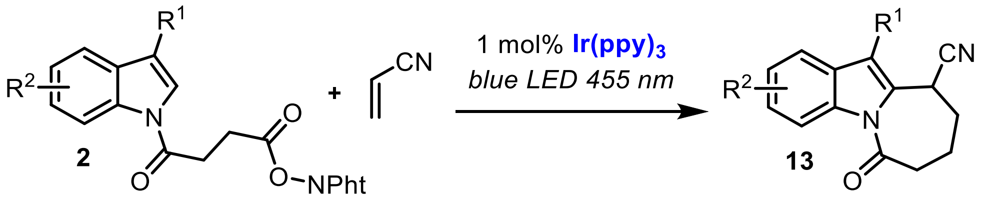

Compound **2** (0.10 mmol, 1.0 equiv.) and $Ir(ppy)_3$ (0,7 mg, 1 mol%) were weighed in a vial equipped with a magnetic stirrer bar. The vial was sealed. DMSO (2 mL, 0.05 M) was introduced. The reaction mixture was put under vacuum (4-5 mbar) and then backfilled with nitrogen (5 times). Acrylonitrile was then introduced (40 µL, 6.0 equiv.) and the reaction was stirred at 23°C under blue light irradiation (455 nm) for 8 hours. The reaction mixture was diluted in 25 mL of Et_2O and washed with 25 mL of water (x2), 2M NaOH solution (x2), then brine. The organic layer was dried over Na_2SO_4 and concentrated *in vacuo*. Purification by column chromatography on silica gel (eluent: petroleum ether/DCM 50/50 to 0/100) afforded **13**.

Comparison between different olefins for the synthesis of azepinoindolones:

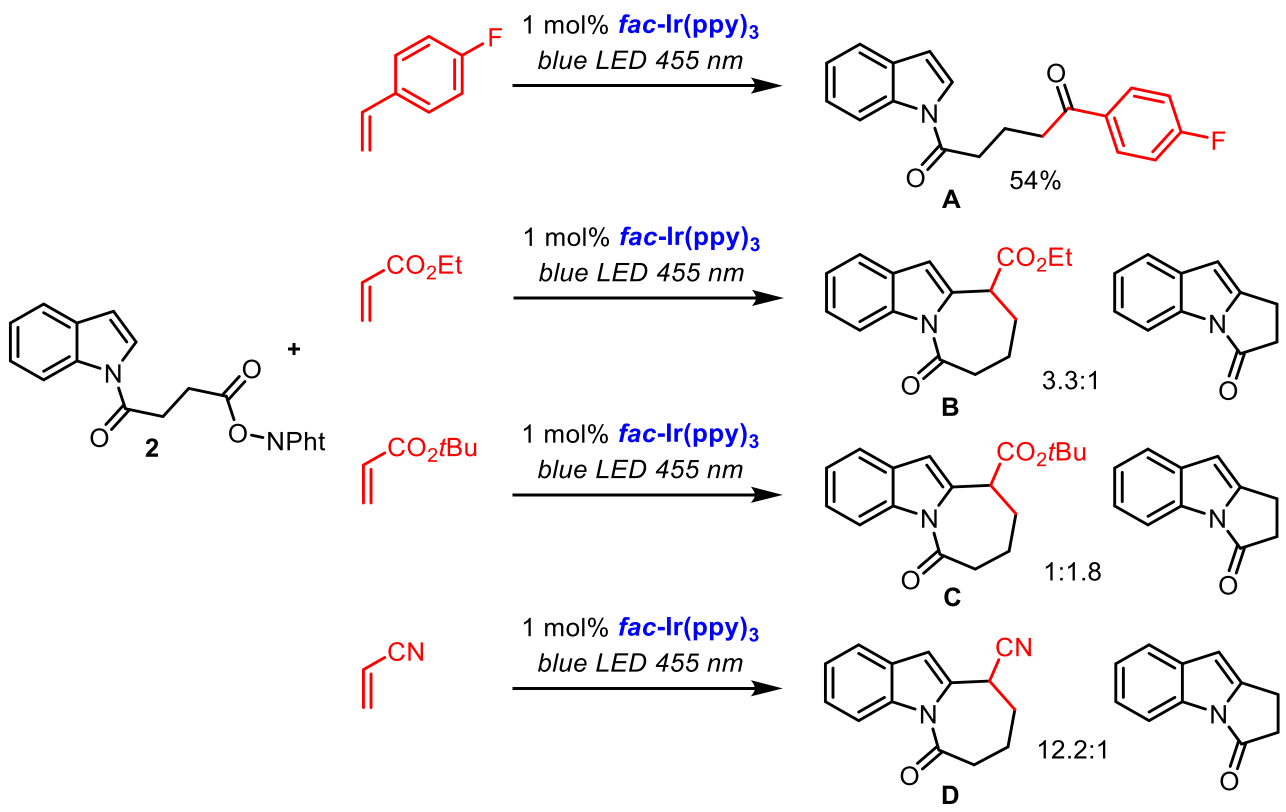

Compound **2** (0.10 mmol, 1.0 equiv.) and Ir(ppy)$_3$ (0,7 mg, 1 mol%) were weighed in a vial equipped with a magnetic stirrer bar. The vial was sealed. DMSO (2 mL, 0.05 M) was introduced. The reaction mixture was put under vacuum (4-5 mbar) and then backfilled with nitrogen (5 times). The olefin was then introduced (3.0 equiv.) and the reaction was stirred at 23°C under blue light irradiation (455 nm) for 8 hours. The reaction mixture was diluted in 25 mL of Et_2O and washed with 25 mL of water (x2), 2M NaOH solution (x2), then brine. The organic layer was dried over Na_2SO_4 and concentrated *in vacuo*. Evaluation of the crude mixture was performed by NMR analysis. Purification by column chromatography on silica gel afforded diketone **A** in 54% yield.

Synthesis of 8:

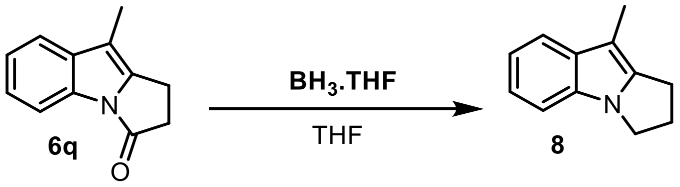

Compound **6q** (19.0 mg, 103 µmol) was weighed in a vial equipped with a magnetic stirrer bar. The vial was sealed and dry THF (700 µL) was introduced. Then BH_3.THF was added (1.0 M solution in THF, 310 µL, 3 equiv.). The reaction mixture was at 65°C for 20 hours. The reaction mixture was cooled down to 23°C and 1 mL of MeOH was added. The mixture was stirred at 23°C for 8 hours. The solvent was removed *in vacuo* and purification by column chromatography on silica gel (eluent: DCM) afforded **8** (15.8 mg, 92.3 µmol, 90% yield) as a colourless oil.

Synthesis of 9:

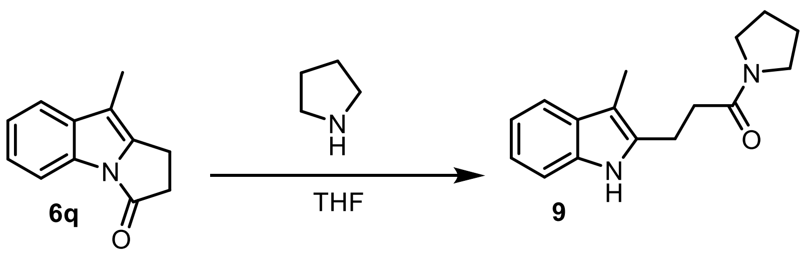

Compound **6q** (19.0 mg, 103 µmol) was weighed in a vial equipped with a magnetic stirrer bar. The vial was sealed and dry THF (1 mL) was introduced. Then pyrrolidine (26 µL, 308 µmol, 3 equiv.) and DBU (46 µL, 308 µmol, 3 equiv.) were added. The reaction mixture was at 65°C for 20 hours. The reaction mixture was cooled down to 23°C. The reaction mixture was diluted in 25 mL of Et_2O and washed with 15 mL of HCl 2N and 10 ml of brine, then 25 mL of brine. The organic layer was dried over Na_2SO_4 and concentrated *in vacuo*. Purification by column chromatography on silica gel (eluent: DCM/AcOEt 100/0 to 80/20) afforded **9** (26.4 mg, 103 µmol, 99% yield) as a white solid.

Synthesis of 10:

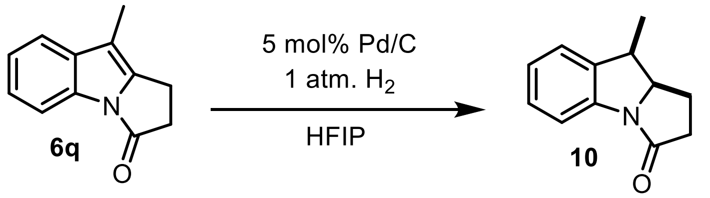

Compound **6q** (19.0 mg, 103 µmol) was weighed in a vial equipped with a magnetic stirrer bar. The vial was sealed and put under vacuum (5 mbar) and a balloon of H_2 was installed to obtain one atmosphere of H_2. Then HFIP (1.5 mL) and DCM (0.5 mL) were added. The mixture was stirred at 23°C for 14 hours. The reaction mixture was filtered over a plug of silica gel with DCM/AcOEt 2/1 as eluent. The solvent was removed *in vacuo* to afford **10** (19.2 mg, 103 µmol, 99% yield) as a white solid.

Synthesis of 11:

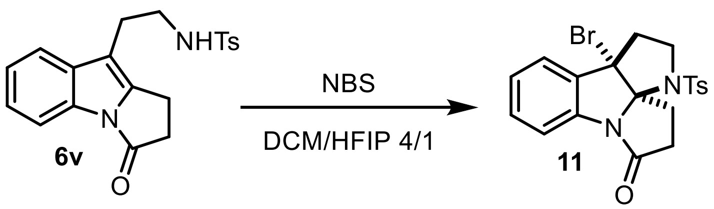

Compound **6v** (26.0 mg, 70.6 µmol) was weighed in a vial equipped with a magnetic stirrer bar. DCM (1 mL) and HFIP (0.25 mL) were added. Then NBS (12.6 mg, 70.6 µmol, 1.0 equiv.) was added. The vial was sealed. The reaction mixture was stirred at 23°C for 2 hours. The solvent was removed *in vacuo* and purification by column chromatography on silica gel (eluent: DCM/AcOEt 100/0 to 90/10) afforded **11** (29.6 mg, 66.2 µmol, 94% yield) as a white solid.

Gram-scale reaction:

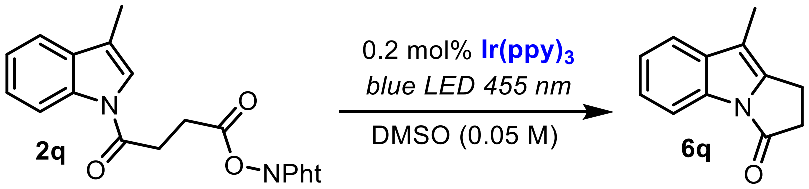

Compound **2q** (1.60 g, 4.25 mmol) and Ir(ppy)$_3$ (5,6 mg, 8.5µmol, 0.20 mol%) were weighed in a vial equipped with a magnetic stirrer bar. The vial was sealed and DMSO (85 mL, 0.05 M) was introduced. The reaction mixture was put under vacuum (4-5 mbar) and then backfilled with nitrogen (5 times). The reaction was stirred at 23°C under blue light irradiation (455 nm) for 16 hours. The reaction mixture was diluted in 250 mL of Et_2O and 50 mL of DCM. The organic layer was washed with 200 mL of water (x2), then 150 mL of 2M NaOH solution (x2), then 50 mL of brine. The organic layer was dried over Na_2SO_4 and concentrated *in vacuo*. Purification by column chromatography on silica gel (eluent: petroleum ether/DCM 50/50 to 0/100) afforded **6q** (606 mg, 3.27 mmol, 77% yield) as a white solid.

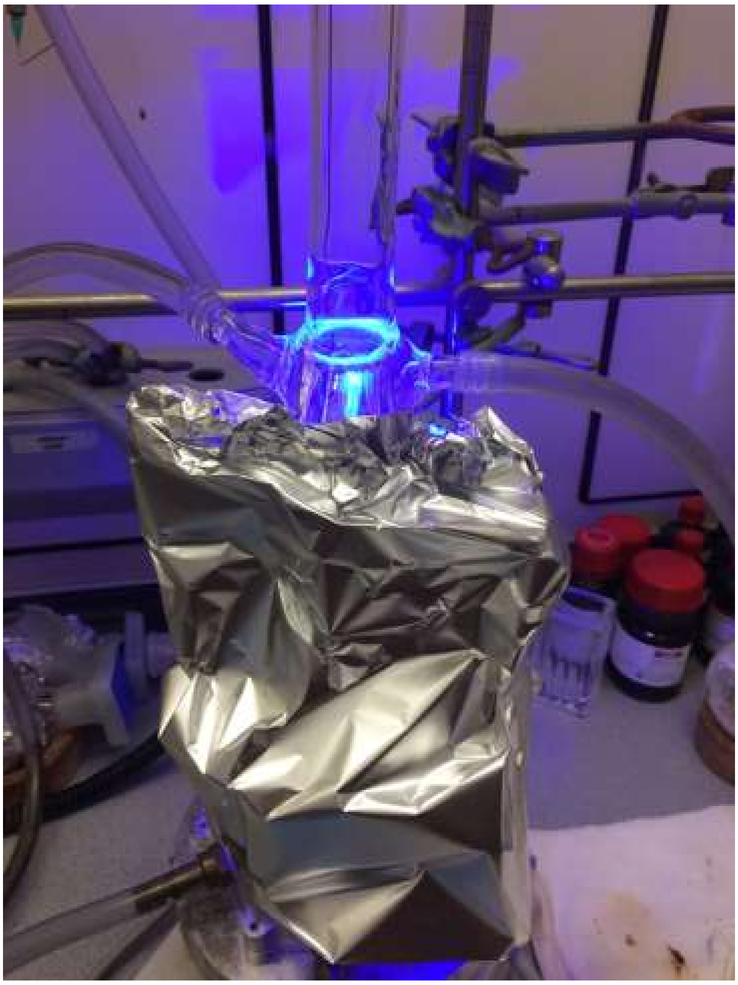

Reaction on-going

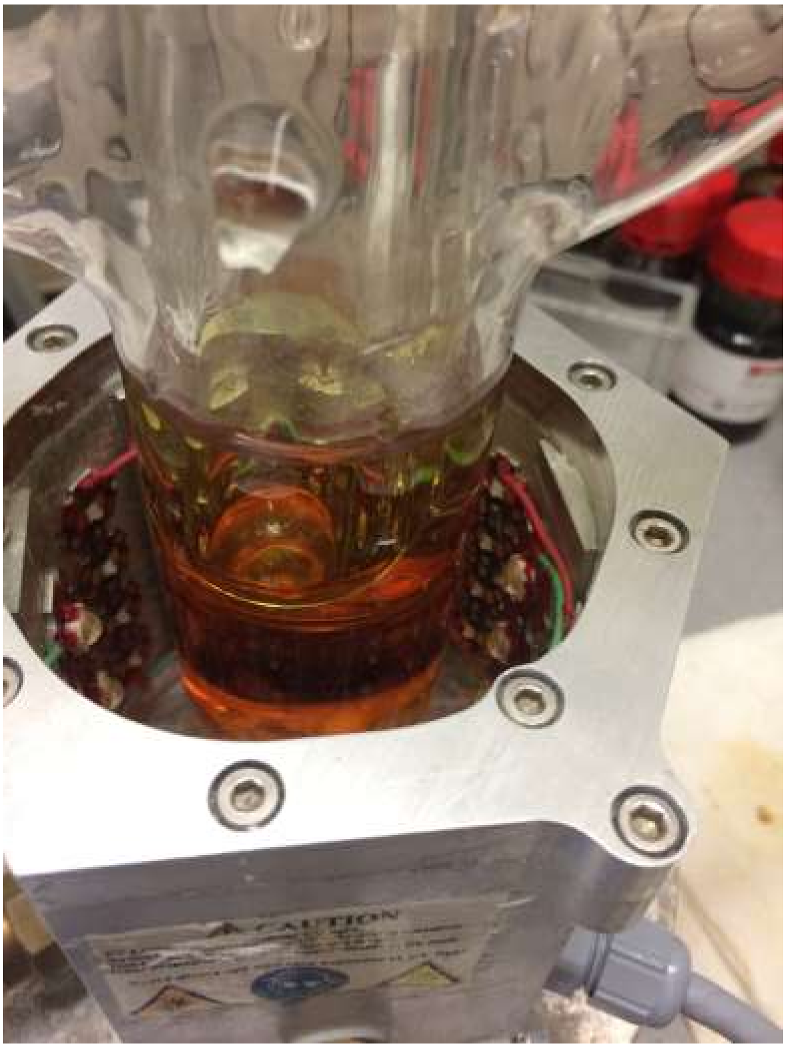

Reaction set-up before work-up

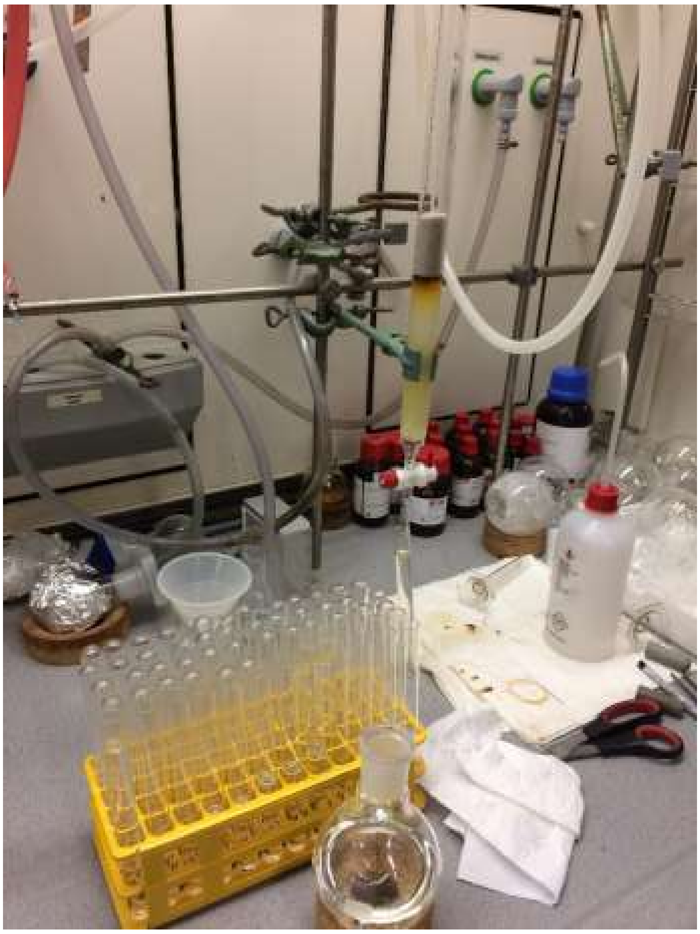

Purification by chromatography on silica gel

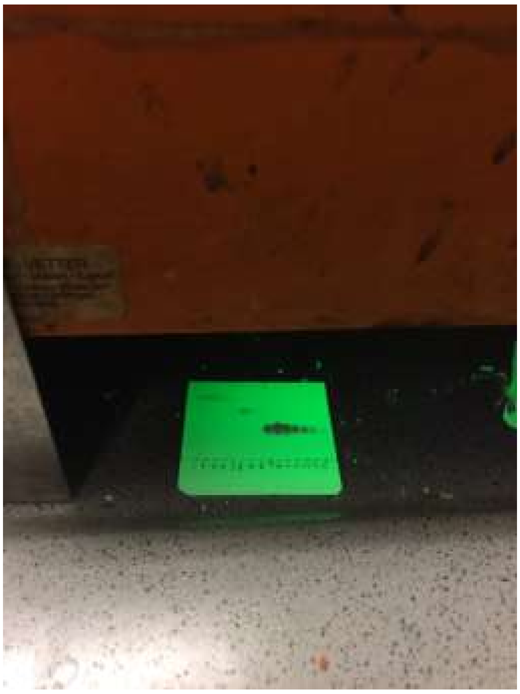

TLC of different chromatography fractions

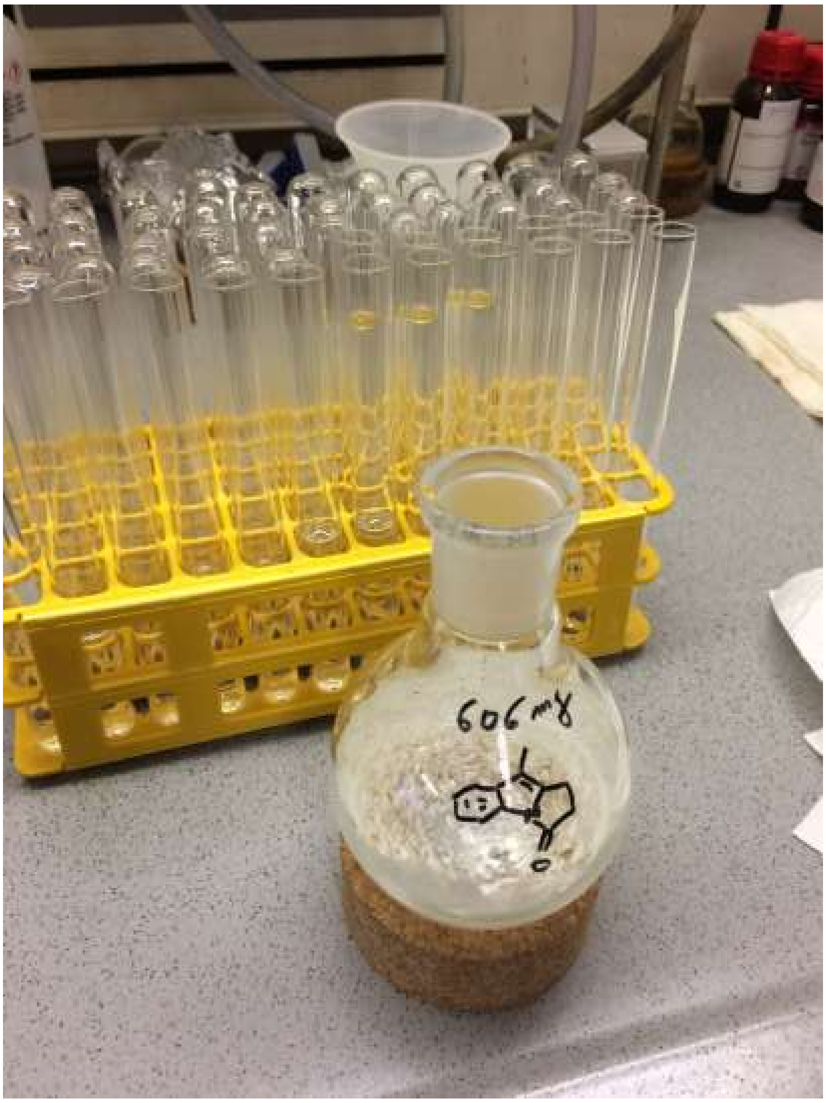

Final product after evaporation of the eluent by rotavapor distillation

Characterization data

4-(1H-indol-1-yl)-4-oxobutanoic acid (7a):

Obtained *via* GP1.

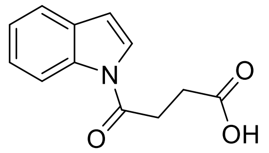

^{1}H NMR (300 MHz, $CDCl_3$) δ 8.44 (d, *J* = 8.4 Hz, 1H), 7.60 – 7.55 (m, 1H), 7.49 (d, *J* = 3.8 Hz, 1H), 7.39 – 7.27 (m, 2H), 6.67 (dd, *J* = 3.8, 0.6 Hz, 1H), 3.28 (dd, *J* = 7.2, 5.9 Hz, 2H), 2.92 (dd, *J* = 7.1, 5.9 Hz, 2H).
^{13}C NMR (75 MHz, $CDCl_3$): δ 177.5, 169.6, 135.6, 130.3, 125.3, 124.2, 123.8, 120.9, 116.6, 109.7, 30.5, 28.5.
IR: υ = 2970, 1740, 1707, 1449, 1364, 1308, 1230, 1207, 1110, 1041, 913, 767, 745 cm^{-1}.
HRMS: (ESI): calculated: m/z = 218.0810 $[M+H]^+$; found: m/z = 218.0812 $[M+H]^+$.
m.p.: 162-163°C.

(cis)-2-(1H-indole-1-carbonyl)cyclohexane-1-carboxylic acid (7b):

Obtained *via* GP1.

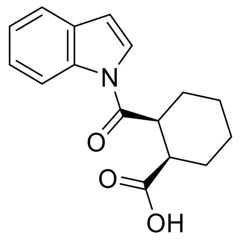

^{1}H NMR (300 MHz, $CDCl_3$) δ 8.43 (d, *J* = 8.1 Hz, 1H), 7.55 (d, *J* = 7.6 Hz, 1H), 7.43 (d, *J* = 3.7 Hz, 1H), 7.37 – 7.21 (m, 2H), 6.62 (d, *J* = 3.7 Hz, 1H), 3.76 (dd, *J* = 8.5, 4.2 Hz, 1H), 2.70 – 2.58 (m, 1H), 2.46 – 2.30 (m, 1H), 2.25 – 2.12 (m, 1H), 2.10 – 1.97 (m, 1H), 1.92 – 1.71 (m, 2H), 1.59 – 1.25 (m, 3H).
^{13}C NMR (75 MHz, $CDCl_3$): δ 179.6, 172.8, 135.7, 130.2, 125.1, 124.9, 123.6, 120.7, 116.8, 109.0, 43.0, 41.1, 28.3, 24.6, 22.0.
IR: υ = 2937, 2858, 1699, 1535, 1453, 1401, 1304, 1267, 1233, 1203, 1155, 1107, 909, 861, 752 cm^{-1}.
HRMS: (ESI): calculated: m/z = 272.1283 $[M+H]^+$; found: m/z = 272.1281 $[M+H]^+$.
m.p.: 146-147°C.

(cis)-(1H-indole-1-carbonyl)cyclohex-3-ene-1-carboxylic acid (7c):

Obtained *via* GP1.

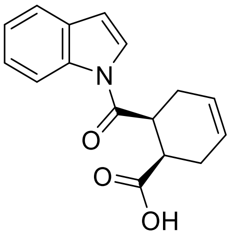

^{1}H NMR (300 MHz, $CDCl_3$) δ 8.41 (d, *J* = 8.2 Hz, 1H), 7.59 – 7.53 (m, 1H), 7.44 (d, *J* = 3.8 Hz, 1H), 7.37 – 7.23 (m, 2H), 6.65 (dd, *J* = 3.8, 0.6 Hz, 1H), 5.88 – 5.78 (m, 1H), 5.69 – 5.59 (m, 1H), 3.85 (td, *J* = 5.7, 3.6 Hz, 1H), 3.03 – 2.89 (m, 2H), 2.63 – 2.44 (m, 3H).
^{13}C NMR (75 MHz, $CDCl_3$): δ 178.9, 172.5, 135.7, 130.2, 126.1, 125.2, 124.6, 123.8, 122.9, 120.8, 116.9, 109.5, 39.6, 38.7, 27.3, 25.6.
IR: υ = 3034, 2922, 2847, 1699, 1535, 1453, 1358, 1304, 1207, 1084, 946, 902, 752 cm^{-1}.
HRMS: (ESI): calculated: m/z = 270.1128 $[M+H]^+$; found: m/z = 270.1125 $[M+H]^+$.
m.p.: 82-83°C.

4-(1H-indol-1-yl)-2,2-dimethyl-4-oxobutanoic acid (7d):

Obtained *via* GP1.

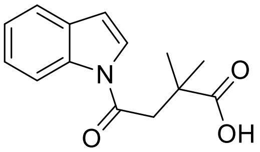

^{1}H NMR (300 MHz, DMSO) δ 8.32 (d, *J* = 8.0 Hz, 1H), 7.91 (d, *J* = 3.8 Hz, 1H), 7.65 – 7.59 (m, 1H), 7.35 – 7.21 (m, 2H), 6.75 (d, *J* = 3.8 Hz, 1H), 3.32 (s, 2H), 1.29 (s, *J* = 8.2 Hz, 6H).
^{13}C NMR (75 MHz, DMSO) δ 178.6, 170.7, 135.3, 130.6, 126.9, 125.1, 123.9, 121.4, 116.4, 108.8, 44.8, 25.9.
IR: υ = 2978, 2926, 1692, 1535, 1453, 1394, 1356, 1304, 1207, 1133, 939, 909, 879, 752 cm^{-1}.
HRMS: (ESI): calculated: m/z = 246.1125 $[M+H]^+$; found: m/z = 246.1125 $[M+H]^+$.
m.p.: 188-189°C.

5-(1H-indol-1-yl)-5-oxopentanoic acid (7e):

Obtained *via* GP1.

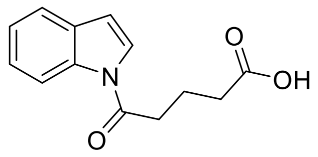

^{1}H NMR (300 MHz, $CDCl_3$) δ 8.46 (d, *J* = 8.2 Hz, 1H), 7.57 (d, *J* = 7.7 Hz, 1H), 7.47 (d, *J* = 3.7 Hz, 1H), 7.40 – 7.32 (m, 1H), 7.32 – 7.24 (m, 1H), 6.65 (d, *J* = 3.7 Hz, 1H), 3.03 (t, *J* = 7.2 Hz, 2H), 2.60 (t, *J* = 7.0 Hz, 2H), 2.18 (p, *J* = 7.0 Hz, 2H).

^{13}C NMR (75 MHz, $CDCl_3$) δ 179.0, 170.6, 135.6, 130.3, 125.2, 124.5, 123.8, 120.9, 116.6, 109.4, 34.6, 32.8, 19.4.

IR: υ = 2907, 1692, 1453, 1390, 1312, 1271, 1200, 1140, 1110, 1021, 935, 902, 741 cm^{-1}.

HRMS: (ESI): calculated: m/z = 232.0967 $[M+H]^+$; found: m/z = 232.0968 $[M+H]^+$.

m.p.: 124-125°C.

5-(1H-indol-1-yl)-3-methyl-5-oxopentanoic acid (7f):

Obtained *via* GP1.

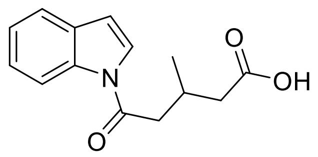

^{1}H NMR (300 MHz, Chloroform-*d*) δ 8.48 (d, *J* = 8.2 Hz, 1H), 7.61 – 7.55 (m, 1H), 7.50 (d, *J* = 3.8 Hz, 1H), 7.39 – 7.32 (m, 1H), 7.31 – 7.24 (m, 1H), 6.65 (dd, *J* = 3.8, 0.5 Hz, 1H), 3.17 – 3.03 (m, 1H), 2.85 – 2.68 (m, 2H), 2.58 (dd, *J* = 15.9, 6.5 Hz, 1H), 2.45 (dd, *J* = 15.9, 6.4 Hz, 1H), 1.17 (d, *J* = 6.5 Hz, 3H).

^{13}C NMR (75 MHz, Chloroform-*d*): δ 178.6, 170.2, 135.6, 130.4, 125.2, 124.7, 123.8, 120.9, 116.7, 109.4, 41.9, 40.5, 27.0, 20.0.

IR: υ = 3116, 2967, 1699, 1539, 1449, 1386, 1326, 1207, 1080, 943, 902, 752 cm^{-1}.

HRMS: (ESI): calculated: m/z = 246.1126 $[M+H]^+$; found: m/z = 246.1126 $[M+H]^+$.

m.p.: 113-114°C.

5-(1H-indol-1-yl)-3,3-dimethyl-5-oxopentanoic acid (7g):

Obtained *via* GP1.

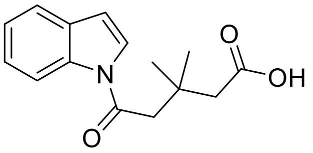

^{1}H NMR (300 MHz, $CDCl_3$) δ 8.50 (d, *J* = 8.2 Hz, 1H), 7.60 – 7.53 (m, 1H), 7.49 (d, *J* = 3.8 Hz, 1H), 7.40 – 7.32 (m, 1H), 7.32 – 7.25 (m, 1H), 6.63 (d, *J* = 3.8 Hz, 1H), 3.08 (s, 2H), 2.68 (s, 2H), 1.25 (s, 6H).

^{13}C NMR (75 MHz, $CDCl_3$) δ 177.2, 170.5, 135.7, 130.5, 125.2, 125.0, 123.8, 120.9, 116.9, 109.3, 44.7, 44.4, 33.3, 28.4.

IR: υ = 2963, 1699, 1530, 1449, 1382, 1349, 1304, 1230, 1207, 1155, 1103, 905, 749 cm^{-1}.

HRMS: (ESI): calculated: m/z = 260.1282 $[M+H]^+$; found: m/z = 260.1281 $[M+H]^+$.

4-(5-methoxy-1H-indol-1-yl)-4-oxobutanoic acid (7h):

Obtained *via* GP1.

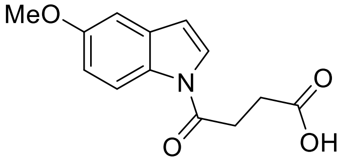

^{1}H NMR (300 MHz, DMSO-*d*) δ 12.29 (s, 1H), 8.21 (d, *J* = 9.0 Hz, 1H), 7.91 (d, *J* = 3.8 Hz, 1H), 7.14 (d, *J* = 2.5 Hz, 1H), 6.92 (dd, *J* = 9.0, 2.6 Hz, 1H), 6.69 (dd, *J* = 3.7, 0.5 Hz, 1H), 3.79 (s, 3H), 3.23 (dd, *J* = 7.0, 5.4 Hz, 2H), 2.65 (dd, *J* = 7.0, 5.5 Hz, 2H).

^{13}C NMR (75 MHz, DMSO-*d*): δ 174.1, 171.1, 156.3, 131.7, 130.0, 127.3, 117.00, 113.4, 108.9, 104.0, 55.7, 30.4, 28.6.

IR: υ = 2933, 2844, 2624, 1605, 1613, 1580, 1476, 1435; 1386, 1252, 1192, 1148, 1114, 1021, 924, 875, 823, 715 cm^{-1}.

HRMS: (ESI): calculated: m/z = 248.0917 $[M+H]^+$; found: m/z = 248.0919 $[M+H]^+$.

m.p.: 160-161°C.

4-(5-methyl-1H-indol-1-yl)-4-oxobutanoic acid (7i):

Obtained *via* GP1.

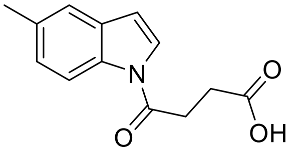

^{1}H NMR (300 MHz, DMSO) δ 12.29 (s, 1H), 8.20 (d, *J* = 8.4 Hz, 1H), 7.89 (d, *J* = 3.8 Hz, 1H), 7.43 – 7.38 (m, 1H), 7.14 (dd, *J* = 8.5, 1.5 Hz, 1H), 6.68 (dd, *J* = 3.7, 0.4 Hz, 1H), 3.24 (dd, *J* = 7.0, 5.4 Hz, 2H), 2.65 (dd, *J* = 6.9, 5.5 Hz, 2H), 2.39 (s, 3H).
^{13}C NMR (75 MHz, DMSO): δ 174.1, 171.3, 133.6, 132.8, 130.9, 126.8, 126.3, 121.2, 116.0, 108.7, 30.6, 28.6, 21.4.
IR: υ = 2594, 2922, 1692, 1580, 1543, 1464, 1394, 1358, 1312, 1218, 1203, 1054, 984, 909, 806, 767, 715 cm^{-1}.
HRMS: (ESI): calculated: m/z = 232.0968 $[M+H]^+$; found: m/z = 232.0968 $[M+H]^+$.
m.p.: 168-169°C.

3-methyl-5-(4-methyl-1H-indol-1-yl)-5-oxopentanoic acid (7j):

Obtained *via* GP1.

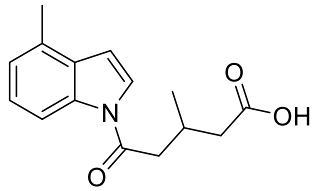

^{1}H NMR (300 MHz, $CDCl_3$) δ 8.31 (d, *J* = 8.3 Hz, 1H), 7.50 (d, *J* = 3.9 Hz, 1H), 7.26 (t, *J* = 7.8 Hz, 1H), 7.08 (d, *J* = 7.3 Hz, 1H), 6.69 (dd, *J* = 3.8, 0.6 Hz, 1H), 3.11 (dd, *J* = 15.2, 5.5 Hz, 1H), 2.85 – 2.68 (m, 2H), 2.62 – 2.41 (m, 2H), 2.53 (s, 3H), 1.17 (d, *J* = 6.5 Hz, 3H).
^{13}C NMR (75 MHz, $CDCl_3$) δ 178.4, 170.2, 135.4, 130.3, 130.0, 125.3, 124.2, 124.1, 114.2, 107.7, 42.0, 40.5, 27.0, 20.0, 18.5.
IR: υ = 3049, 2967, 2922, 1703, 1535, 1487, 1453, 1420, 1326, 1233, 1155, 1080, 916, 760, 715, 685 cm^{-1}.
HRMS: (ESI): calculated: m/z = 260.1282 $[M+H]^+$; found: m/z = 260.1281 $[M+H]^+$.
m.p.: 126-127°C.

4-(5-(methoxycarbonyl)-1H-indol-1-yl)-4-oxobutanoic acid (7k):

Obtained *via* GP1.

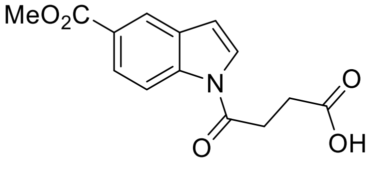

^{1}H NMR (300 MHz, DMSO) δ 12.32 (s, 1H), 8.42 (d, *J* = 8.7 Hz, 1H), 8.28 (d, *J* = 1.3 Hz, 1H), 8.08 (d, *J* = 3.8 Hz, 1H), 7.93 (dd, *J* = 8.7, 1.7 Hz, 1H), 6.90 (d, *J* = 3.7 Hz, 1H), 3.87 (s, 3H), 3.33 – 3.26 (m, 2H), 2.72 – 2.64 (m, 2H).

^{13}C NMR (75 MHz, DMSO): δ 174.0, 172.0, 166.9, 137.9, 130.6, 128.4, 126.0, 125.1, 123.2, 116.2, 109.2, 52.5, 30.8, 28.6.

IR: υ = 3138, 1692, 1539, 1438, 1396, 1289, 1259, 1181, 1088, 909, 760, 730 cm^{-1}.

HRMS: (ESI): calculated: m/z = 276.0867 $[M+H]^+$; found: m/z = 276.0866 $[M+H]^+$.

m.p.: 194-195°C.

5-(6-(methoxycarbonyl)-1H-indol-1-yl)-5-oxopentanoic acid (7l):

Obtained *via* GP1.

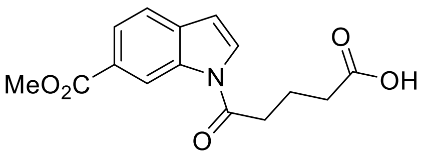

^{1}H NMR (300 MHz, DMSO) δ 12.16 (s, 1H), 9.01 (s, 1H), 8.11 (d, *J* = 3.7 Hz, 1H), 7.87 (dd, *J* = 8.2, 1.3 Hz, 1H), 7.73 (d, *J* = 8.2 Hz, 1H), 6.84 (d, *J* = 3.6 Hz, 1H), 3.88 (s, 3H), 3.11 (t, *J* = 7.2 Hz, 2H), 2.39 (t, *J* = 7.3 Hz, 2H), 1.93 (p, *J* = 7.2 Hz, 2H).

^{13}C NMR (75 MHz, DMSO) δ 174.6, 172.4, 167.1, 134.8, 134.5, 130.3, 126.0, 124.6, 121.3, 117.7, 108.6, 52.6, 34.6, 33.0, 19.9.

IR: υ = 3015, 2952, 1703, 1431, 1378, 1293, 1252, 1192, 1110, 946, 905; 888, 760; 678 cm^{-1}.

HRMS: (ESI): calculated: m/z = 290.1025 $[M+H]^+$; found: m/z = 290.1023 $[M+H]^+$.

m.p.: 182-183°C.

4-(6-chloro-1H-indol-1-yl)-4-oxobutanoic acid (7m):

Obtained *via* GP1.

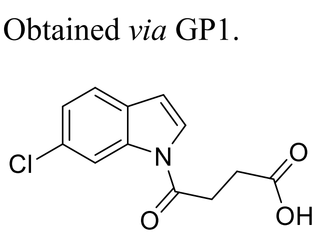

^{1}H NMR (300 MHz, DMSO) δ 12.32 (s, 1H), 8.35 (d, *J* = 1.9 Hz, 1H), 8.00 (d, *J* = 3.8 Hz, 1H), 7.65 (d, *J* = 8.4 Hz, 1H), 7.32 (dd, *J* = 8.4, 2.0 Hz, 1H), 6.79 (dd, *J* = 3.8, 0.6 Hz, 1H), 3.27 (dd, *J* = 6.9, 5.5 Hz, 2H), 2.67 (dd, *J* = 6.8, 5.6 Hz, 2H).

^{13}C NMR (75 MHz, DMSO): δ 174.0, 171.9, 135.6, 129.6, 129.4, 127.8, 124.1, 122.7, 116.0, 108.6, 30.7, 28.6.

IR: υ = 3119, 2929, 2676, 1688, 1531, 1431, 1390, 1364, 1304, 1259, 1181, 920, 894, 823, 723 cm^{-1}.

HRMS: (ESI): calculated: m/z = 252.0423 $[M+H]^+$; found: m/z = 252.0422 $[M+H]^+$.

m.p.: 183-184°C.

<u>5-(5-chloro-1H-indol-1-yl)-3,3-dimethyl-5-oxopentanoic acid (7n):</u>

Obtained *via* GP1.

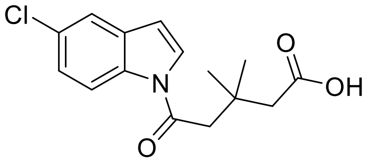

^{1}H NMR (300 MHz, $CDCl_3$) δ 8.42 (d, *J* = 8.9 Hz, 1H), 7.51 (dd, *J* = 3.9, 3.1 Hz, 2H), 7.29 (dd, *J* = 8.9, 2.1 Hz, 1H), 6.56 (dd, *J* = 3.8, 0.6 Hz, 1H), 3.07 (s, 2H), 2.67 (s, 2H), 1.24 (s, 6H).

^{13}C NMR (75 MHz, $CDCl_3$) δ 177.7, 170.2, 134.0, 131.7, 129.3, 126.2, 125.3, 120.5, 117.9, 108.4, 44.4, 44.2, 33.1, 28.3.

IR: υ = 2963, 1703, 1576, 1535, 1446, 1375, 1341, 1308, 1253, 1222, 1192, 1151, 1069, 905, 812, 786, 719 cm^{-1}.

HRMS: (ESI): calculated: m/z = 294.0892 $[M+H]^+$; found: m/z = 294.0891 $[M+H]^+$.

m.p.: 113-114°C.

<u>4-(5-bromo-1H-indol-1-yl)-4-oxobutanoic acid (7o):</u>

Obtained *via* GP1.

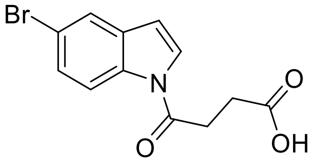

^{1}H NMR (300 MHz, DMSO-*d*) δ 12.31 (s, 1H), 8.27 (d, *J* = 8.8 Hz, 1H), 8.02 (d, *J* = 3.8 Hz, 1H), 7.85 (d, *J* = 1.9 Hz, 1H), 7.47 (dd, *J* = 8.8, 2.0 Hz, 1H), 6.75 (dd, *J* = 3.8, 0.5 Hz, 1H), 3.27 (dd, *J* = 6.9, 5.5 Hz, 2H), 2.66 (dd, *J* = 6.9, 5.5 Hz, 2H).

^{13}C NMR (75 MHz, DMSO-*d*): δ 174.0, 171.7, 134.2, 132.7, 128.3, 127.6, 123.8, 118.0, 116.3, 108.1, 30.6, 28.6.

IR: υ = 1692, 1528, 1446, 1386, 1312, 1263, 1200, 1118, 1054, 913, 715 cm^{-1}.

HRMS: (ESI): calculated: m/z = 295.9917 $[M+H]^+$; found: m/z = 295.9917 $[M+H]^+$.

m.p.: 176-177°C.

(cis)-(1H-pyrrole-1-carbonyl)cyclohexane-1-carboxylic acid (7p):

Obtained *via* GP1.

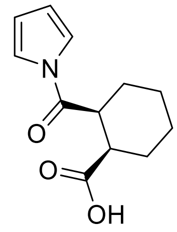

^{1}H NMR (300 MHz, $CDCl_3$) δ 7.33 – 7.26 (m, 2H), 6.31 – 6.22 (m, 2H), 3.68 (dd, *J* = 9.2, 4.6 Hz, 1H), 2.66 (dt, *J* = 10.7, 4.6 Hz, 1H), 2.37 – 2.22 (m, 1H), 2.21 – 2.11 (m, 1H), 2.07 – 1.96 (m, 1H), 1.91 – 1.72 (m, 2H), 1.59 – 1.47 (m, 1H), 1.43 – 1.25 (m, 2H).

^{13}C NMR (75 MHz, $CDCl_3$): δ 180.0, 171.7, 119.2, 112.9, 43.1, 40.3, 28.4, 24.6, 21.9.

IR: υ = 2937, 2858, 1699, 1468, 1412, 1382, 1274, 1233, 1110; 1073, 909, 861, 734 cm^{-1}.

HRMS: (ESI): calculated: m/z = 222.1124 $[M+H]^+$; found: m/z = 222.1125 $[M+H]^+$.

m.p.: 150-151°C.

4-(3-methyl-1H-indol-1-yl)-4-oxobutanoic acid (7q):

Obtained *via* GP1.

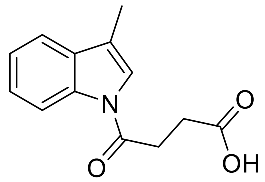

^{1}H NMR (300 MHz, $CDCl_3$) δ 8.41 (d, *J* = 7.2 Hz, 1H), 7.50 (d, *J* = 7.4 Hz, 1H), 7.39 – 7.20 (m, 3H), 3.22 (t, *J* = 6.4 Hz, 2H), 2.89 (t, *J* = 6.5 Hz, 2H), 2.29 (s, 3H).

^{13}C NMR (75 MHz, DMSO) δ (75 MHz, $CDCl_3$) δ 177.9, 169.2, 135.9, 131.4, 125.3, 123.6, 121.2, 118.9, 118.9, 116.6, 30.4, 28.4, 9.8.

IR: υ = 3058, 2922, 2967, 1695, 1457, 1401, 1358, 1267, 1237, 1181, 1073, 909, 730 cm^{-1}.

HRMS: (ESI): calculated: m/z = 232.0968 $[M+H]^+$; found: m/z = 232.0968 $[M+H]^+$.

m.p.: 147-148°C.

5-(3-methyl-1H-indol-1-yl)-5-oxopentanoic acid (7r):

Obtained *via* GP1.

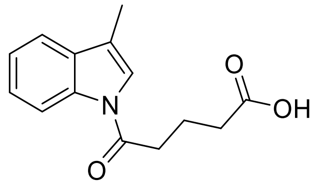

^{1}H NMR (300 MHz, $CDCl_3$) δ 8.43 (d, *J* = 7.7 Hz, 1H), 7.56 – 7.44 (m, 1H), 7.41 – 7.16 (m, 3H), 2.98 (t, *J* = 7.2 Hz, 2H), 2.58 (t, *J* = 7.0 Hz, 2H), 2.28 (d, *J* = 1.2 Hz, 3H), 2.16 (p, *J* = 7.1 Hz, 2H).

^{13}C NMR (75 MHz, $CDCl_3$) δ 179.1, 170.2, 135.9, 131.4, 125.3, 123.5, 121.4, 118.9, 118.6, 116.6, 34.6, 32.8, 19.4, 9.8.

IR: υ = 3026, 2967, 2914, 2624, 1744, 1710, 1446, 1397, 1330, 1282, 1200, 1080, 935, 752, cm^{-1}.

HRMS: (ESI): calculated: m/z = 246.1124 $[M+H]^+$; found: m/z = 246.1125 $[M+H]^+$.

m.p.: 116-117°C.

5-(3-(2-methoxy-2-oxoethyl)-1H-indol-1-yl)-3,3-dimethyl-5-oxopentanoic acid (7s):

Obtained *via* GP1.

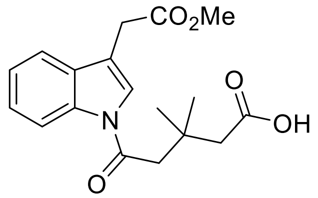

^{1}H NMR (300 MHz, $CDCl_3$) δ 8.49 (d, *J* = 8.0 Hz, 1H), 7.55 – 7.49 (m, 2H), 7.33 (dtd, *J* = 20.2, 7.4, 1.2 Hz, 2H), 3.73 (s, 3H), 3.73 (s, 2H), 3.07 (s, 2H), 2.68 (s, 2H), 1.25 (s, 6H).

^{13}C NMR (75 MHz, $CDCl_3$) δ 177.4, 171.4, 170.2, 135.9, 130.1, 125.5, 123.8, 123.6, 118.8, 117.1, 114.9, 52.3, 44.5, 44.4, 33.2, 30.8, 28.4.

IR: υ = 2955, 1699, 1606, 1449, 1319, 1304, 1237, 1151, 1058, 1017, 905, 745 cm^{-1}.

HRMS: (ESI): calculated: m/z = 332.1493 $[M+H]^+$; found: m/z = 332.1492 $[M+H]^+$.

5-(3-(2-acetamidoethyl)-5-methoxy-1H-indol-1-yl)-5-oxopentanoic acid (7t):

Obtained *via* GP1

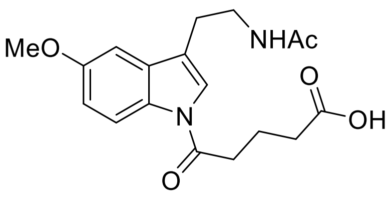

^{1}H NMR (300 MHz, DMSO) δ 12.15 (s, 1H), 8.22 (d, *J* = 9.0 Hz, 1H), 8.01 (t, *J* = 5.5 Hz, 1H), 7.67 (s, 1H), 7.13 (d, *J* = 2.4 Hz, 1H), 6.92 (dd, *J* = 9.0, 2.5 Hz, 1H), 3.81 (s, 3H), 3.36 (dd, *J* = 12.8, 6.9 Hz, 2H), 2.99 (t, *J* = 7.3 Hz, 2H), 2.77 (t, *J* = 7.1 Hz, 2H), 2.37 (t, *J* = 7.3 Hz, 2H), 1.96 – 1.85 (m, 2H), 1.81 (s, 3H).
^{13}C NMR (75 MHz, DMSO): δ 174.6, 171.3, 169.7, 156.3, 131.9, 130.3, 124.2, 119.5, 117.2, 113.4, 102.3, 55.8, 38.6, 34.3, 33.1, 25.3, 23.1, 20.2.
IR: υ = 3414, 2955, 1736, 1692, 1602, 1524, 1464, 1401, 1259, 1207, 1170, 1036, 898, 864, 812, 760 cm^{-1}.
HRMS: (ESI): calculated: m/z = 347.1603 $[M+H]^+$; found: m/z = 347.1601 $[M+H]^+$.
m.p.: 157-158°C.

5-(3-(2-acetamido-3-ethoxy-3-oxopropyl)-1H-indol-1-yl)-5-oxopentanoic acid (7u):

Obtained *via* GP1.

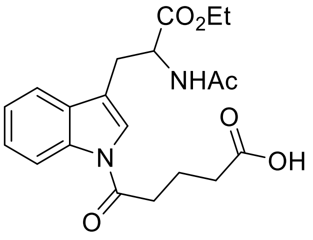

^{1}H NMR (300 MHz, $CDCl_3$) δ 8.41 (d, *J* = 8.0 Hz, 1H), 7.46 (d, *J* = 7.4 Hz, 1H), 7.39 – 7.24 (m, 3H), 6.38 (d, *J* = 7.8 Hz, 1H), 4.96 (dt, *J* = 7.7, 5.8 Hz, 1H), 4.14 (qq, *J* = 10.8, 7.2 Hz, 2H), 3.23 (qd, *J* = 14.9, 5.7 Hz, 2H), 2.97 (t, *J* = 7.3 Hz, 2H), 2.54 (t, *J* = 6.9 Hz, 2H), 2.14 (p, *J* = 7.0 Hz, 2H), 2.00 (s, 3H), 1.20 (t, *J* = 7.1 Hz, 3H).
^{13}C NMR (75 MHz, $CDCl_3$) δ 177.1, 171.8, 170.6, 170.5, 135.7, 130.5, 125.5, 123.7, 122.9, 118.6, 117.1, 116.7, 62.0, 52.4, 34.7, 32.6, 27.6, 23.1, 19.8, 14.1.
IR: υ = 3309, 2974, 2940, 1733, 1703, 1640, 1535, 1543, 1371, 1319, 1203, 1129, 1021, 939, 861, 745 cm^{-1}.
HRMS: (ESI): calculated: m/z = 389.1710 $[M+H]^+$; found: m/z = 389.1707 $[M+H]^+$.
m.p.: 59-60°C.

4-(3-(2-((4-methylphenyl)sulfonamido)ethyl)-1H-indol-1-yl)-4-oxobutanoic acid (7v):

Obtained *via* GP1.

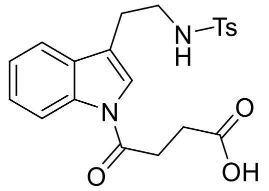

^{1}H NMR (300 MHz, $CDCl_3$) δ 8.31 (d, *J* = 8.1 Hz, 1H), 7.59 (d, *J* = 8.3 Hz, 2H), 7.32 – 7.23 (m, 3H), 7.20 – 7.09 (m, 3H), 5.32 (t, *J* = 6.0 Hz, 1H), 3.27 (q, *J* = 6.3 Hz, 2H), 3.10 (t, *J* = 6.4 Hz, 2H), 2.79 (dt, *J* = 12.6, 6.2 Hz, 4H), 2.32 (s, 3H).

^{13}C NMR (75 MHz, $CDCl_3$): δ 177.7, 169.6, 143.5, 136.5, 135.9, 129.8, 129.6, 126.8, 125.4, 123.6, 122.5, 118.8, 118.6, 116.7, 42.2, 30.4, 28.3, 25.3, 21.5.

IR: υ = 3272, 2929, 1699, 1602, 1453, 1397, 1319, 1253, 1215, 1161, 1155, 1092, 909, 812, 730, 663 cm^{-1}.

HRMS: (ESI): calculated: m/z = 415.1325 $[M+H]^+$; found: m/z = 415.1322 $[M+H]^+$.

m.p.: 63-64°C.

1,3-dioxoisoindolin-2-yl 4-(1H-indol-1-yl)-4-oxobutanoate (2a):

Obtained *via* GP2.

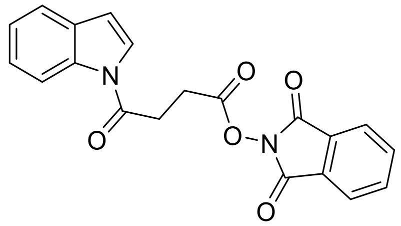

^{1}H NMR (300 MHz, Chloroform-*d*) δ 8.46 (d, *J* = 8.2 Hz, 1H), 7.93 – 7.85 (m, 2H), 7.83 – 7.75 (m, 2H), 7.59 – 7.53 (m, 1H), 7.47 (d, *J* = 3.8 Hz, 1H), 7.40 – 7.33 (m, 1H), 7.31 – 7.25 (m, 1H), 6.67 (dd, *J* = 3.8, 0.5 Hz, 1H), 3.45 – 3.36 (m, 2H), 3.29 – 3.20 (m, 2H).

^{13}C NMR (75 MHz, Chloroform-*d*): δ 168.9, 168.4, 161.8, 135.6, 134.8, 130.3, 128.9, 125.4, 124.1, 124.1, 123.9, 120.9, 116.7, 109.9, 30.5, 25.9.

IR: υ = 1818, 1789, 1736, 1703, 1529, 1453, 1397, 1360, 1207, 1080, 969, 909, 723, 697 cm^{-1}.

HRMS: (ESI): calculated: m/z = 363.0975 $[M+H]^+$; found: m/z = 363.0977 $[M+H]^+$.

m.p.: 158-159°C.

1,3-dioxoisoindolin-2-yl 4-(5-methyl-1H-indol-1-yl)-4-oxobutanoate (2i):

Obtained *via* GP2.

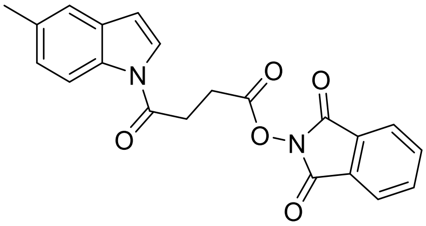

¹H NMR (300 MHz, DMSO) δ 8.21 (d, *J* = 8.4 Hz, 1H), 8.03 – 7.92 (m, 4H), 7.89 (d, *J* = 3.7 Hz, 1H), 7.40 (s, 1H), 7.16 (d, *J* = 8.4 Hz, 1H), 6.69 (d, *J* = 3.7 Hz, 1H), 3.48 (t, *J* = 6.0 Hz, 2H), 3.20 (t, *J* = 6.0 Hz, 2H), 2.39 (s, 3H).

¹³C NMR (75 MHz, DMSO) δ 170.2, 170.0, 162.2, 136.0, 133.6, 133.0, 130.9, 128.6, 126.8, 126.4, 124.4, 121.2, 116.0, 108.9, 30.2, 25.8, 21.4.

IR: υ = 1811, 1781, 1736, 1699, 1610, 1535, 1464, 1312, 1252, 1133, 1088, 1039, 987, 909, 875, 805, 726, 693 cm^{-1}.

HRMS: (ESI): calculated: m/z = 377.1132 $[M+H]^+$; found: m/z = 377.1138 $[M+H]^+$.

m.p.: 184-185°C.

1,3-dioxoisoindolin-2-yl 4-(5-methoxy-1H-indol-1-yl)-4-oxobutanoate (2h):

Obtained *via* GP2.

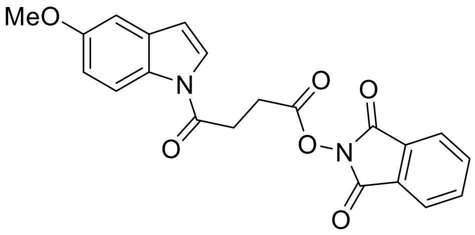

¹H NMR (300 MHz, Chloroform-*d*) δ 8.33 (d, *J* = 9.0 Hz, 1H), 7.92 – 7.85 (m, 2H), 7.81 – 7.74 (m, 2H), 7.43 (d, *J* = 3.8 Hz, 1H), 7.01 (d, *J* = 2.4 Hz, 1H), 6.95 (dd, *J* = 9.0, 2.6 Hz, 1H), 6.59 (dd, *J* = 3.8, 0.5 Hz, 1H), 3.84 (s, 3H), 3.38 (dd, *J* = 10.3, 3.8 Hz, 2H), 3.23 (dd, *J* = 10.5, 4.0 Hz, 2H).

¹³C NMR (75 MHz, Chloroform-*d*): δ 168.9, 168.0, 161.8, 156.6, 134.8, 131.3, 130.4, 128.9, 124.7, 124.0, 117.4, 113.6, 109.7, 103.7, 55.7, 30.2, 25.9.

IR: υ = 1815, 1785, 1740, 1677, 1591, 1472, 1442, 1397, 1274, 1185, 1148, 1080, 1054, 1028, 969, 939, 838, 700 cm^{-1}.

HRMS: (ESI): calculated: m/z = 393.1081 $[M+H]^+$; found: m/z = 393.1084 $[M+H]^+$.

m.p.: 165-166°C.

1,3-dioxoisoindolin-2-yl 4-(3-methyl-1H-indol-1-yl)-4-oxobutanoate (2q):

Obtained *via* GP2.

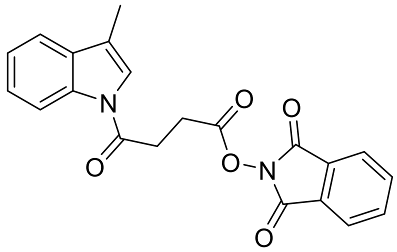

^{1}H NMR (300 MHz, Chloroform-*d*) δ 8.43 (d, *J* = 7.7 Hz, 1H), 7.93 – 7.86 (m, 2H), 7.82 – 7.75 (m, 2H), 7.52 – 7.47 (m, 1H), 7.33 (dtd, *J* = 19.5, 7.3, 1.2 Hz, 2H), 7.23 (s, 1H), 3.41 – 3.32 (m, 2H), 3.30 – 3.20 (m, 2H), 2.28 (d, *J* = 1.3 Hz, 3H).
^{13}C NMR (75 MHz, Chloroform-*d*): δ 169.0, 168.0, 161.8, 135.9, 134.8, 131.3, 128.9, 125.4, 124.1, 123.7, 121.0, 119.2, 118.9, 116.7, 30.4, 25.9, 9.8.
IR: υ = 1818, 1744, 1699, 1606, 1453, 1401, 1349, 1322, 1215, 1069, 749, 697 cm^{-1}.
HRMS: (ESI): calculated: m/z = 377.1132 $[M+H]^+$; found: m/z = 377.1136 $[M+H]^+$.
m.p.: 185-186°C.

1,3-dioxoisoindolin-2-yl 4-(3-(2-((4-methylphenyl)sulfonamido)ethyl)-1H-indol-1-yl)-4-oxobutanoate (2v):

Obtained *via* GP2.

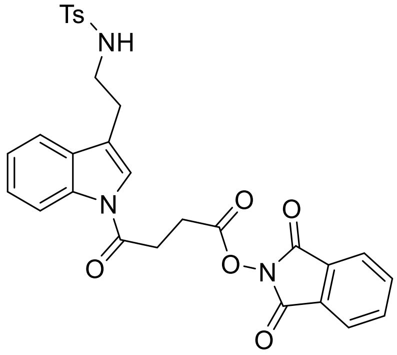

^{1}H NMR (300 MHz, Chloroform-*d*) δ 8.34 (d, *J* = 8.1 Hz, 1H), 7.89 – 7.82 (m, 2H), 7.79 – 7.74 (m, 2H), 7.61 (d, *J* = 8.3 Hz, 2H), 7.31 (t, *J* = 7.2 Hz, 2H), 7.23 – 7.11 (m, 4H), 5.16 (t, *J* = 6.1 Hz, 1H), 3.34 – 3.20 (m, 4H), 3.18 – 3.08 (m, 2H), 2.83 (t, *J* = 6.5 Hz, 2H), 2.34 (s, 3H).

^{13}**C NMR** (75 MHz, Chloroform-*d*): δ 169.0, 168.3, 161.8, 143.5, 136.7, 135.9, 134.9, 129.8, 129.6, 128.8, 126.9, 125.5, 124.0, 123.7, 122.2, 119.0, 118.6, 116.8, 42.3, 30.3, 25.8, 25.4, 21.5.

IR: υ = 1815, 1785, 1736, 1699, 1599, 1453, 1397, 1358, 1319, 1215, 1155, 1069, 965, 875, 812, 749, 697 cm^{-1}.

HRMS: (ESI): calculated: m/z = 560.1486 [M+H]$^+$; found: m/z = 560.1493 [M+H]$^+$.

m.p.: 94-95°C.

1,3-dioxoisoindolin-2-yl 2,2-dimethyl-4-(3-methyl-1H-indol-1-yl)-4-oxobutanoate (2w):

Obtained *via* GP2.

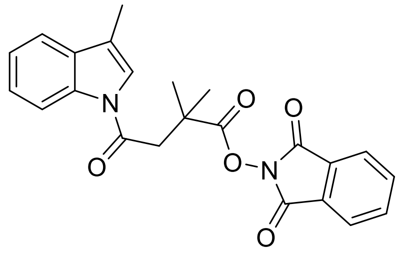

^{1}**H NMR** (300 MHz, Chloroform-*d*) δ 8.53 (d, *J* = 7.4 Hz, 1H), 7.86 (dd, *J* = 5.4, 3.2 Hz, 2H), 7.75 (dd, *J* = 5.6, 3.1 Hz, 2H), 7.49 (d, *J* = 7.0 Hz, 1H), 7.41 – 7.19 (m, 3H), 3.37 (s, 2H), 2.28 (d, *J* = 1.1 Hz, 3H), 1.64 (s, 6H).

^{13}**C NMR** (75 MHz, Chloroform-*d*): δ 173.2, 167.4, 162.0, 136.0, 134.6, 131.3, 129.0, 125.4, 123.9, 123.5, 121.1, 118.9, 118.8, 117.0, 45.3, 40.2, 25.7, 9.8.

IR: υ = 2978, 2922, 1811, 1785, 1740, 1699, 1610, 1449, 1379, 1315, 1237, 1133, 1051, 969, 909, 879, 697 cm^{-1}.

HRMS: (ESI): calculated: m/z = 405.1445 [M+H]$^+$; found: m/z = 405.1451 [M+H]$^+$.

m.p.: 149-150°C.

1,2-dihydro-3H-pyrrolo[1,2-a]indol-3-one (6a):

Obtained *via* GP3.

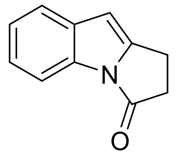

^{1}**H NMR** (300 MHz, Chloroform-*d*) δ 8.11 – 8.04 (m, 1H), 7.54 – 7.46 (m, 1H), 7.32 – 7.22 (m, 2H), 6.29 – 6.26 (m, 1H), 3.18 – 3.12 (m, 2H), 3.10 – 3.04 (m, 2H).

^{13}C NMR (75 MHz, Chloroform-*d*): δ 171.7, 143.7, 135.3, 130.4, 124.0, 123.2, 120.5, 113.6, 100.4, 34.8, 19.6.
IR: υ = 1707, 1602, 1453, 1386, 1353, 1319, 1170, 909, 816, 752 cm^{-1}.
HRMS: (EI): calculated: m/z = 171.0679 $[M]^{\cdot+}$; found: m/z = 171.0682 $[M]^{\cdot+}$.
m.p.: 150-151°C.

6a,7,8,9,10,10a-hexahydro-6H-isoindolo[2,1-a]indol-6-one (6b):

Obtained *via* GP3.

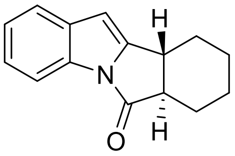

^{1}H NMR (300 MHz, Chloroform-*d*) δ 8.09 – 8.01 (m, 1H), 7.54 – 7.47 (m, 1H), 7.31 – 7.22 (m, 2H), 6.30 – 6.25 (m, 1H), 3.47 (td, *J* = 7.5, 0.9 Hz, 1H), 3.21 (td, *J* = 7.0, 5.8 Hz, 1H), 2.11 – 1.96 (m, 2H), 1.95 – 1.84 (m, 1H), 1.62 – 1.36 (m, 5H).
^{13}C NMR (75 MHz, Chloroform-*d*): δ 173.8, 147.7, 134.8, 130.6, 123.8, 123.3, 120.6, 113.7, 99.5, 46.7, 33.0, 28.9, 23.7, 21.9, 21.8.
IR: υ = 2929, 2855, 1710, 1587, 1449, 1382, 1353, 1297, 1170, 1133, 827, 801, 749 cm^{-1}.
HRMS: (EI): calculated: m/z = 225.1148 $[M]^{\cdot+}$; found: m/z = 225.1142 $[M]^{\cdot+}$.
m.p.: 87-89°C.

6a,7,10,10a-tetrahydro-6H-isoindolo[2,1-a]indol-6-one (6c):

Obtained *via* GP3.

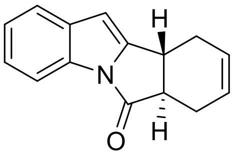

^{1}H NMR (300 MHz, Chloroform-*d*) δ 8.09 – 8.01 (m, 1H), 7.56 – 7.47 (m, 1H), 7.32 – 7.22 (m, 2H), 6.28 (dd, *J* = 1.3, 0.7 Hz, 1H), 5.99 – 5.81 (m, 2H), 3.76 – 3.67 (m, 1H), 3.42 (ddd, *J* = 8.7, 7.7, 3.7 Hz, 1H), 2.67 (ddd, *J* = 15.6, 5.9, 3.7 Hz, 1H), 2.52 – 2.33 (m, 3H).
^{13}C NMR (75 MHz, Chloroform-*d*): δ 174.2, 148.4, 135.4, 130.2, 128.2, 128.0, 124.1, 123.2, 120.7, 113.8, 99.3, 46.2, 32.1, 28.1, 24.1.
IR: υ = 3041, 2944, 2844, 1725, 1587, 1449, 1386, 1326, 1207, 1170, 1088, 976, 887, 797, 745, 700 cm^{-1}.

HRMS: (EI): calculated: m/z = 223.0992 $[M]^{\cdot+}$; found: m/z = 223.0994 $[M]^{\cdot+}$.

m.p.: 82-83°C.

1,1-dimethyl-1,2-dihydro-3H-pyrrolo[1,2-a]indol-3-one (6d):

Obtained *via* GP3.

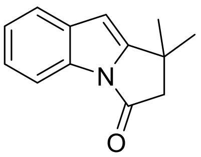

^{1}H NMR (300 MHz, Chloroform-*d*) δ 8.09 – 8.01 (m, 1H), 7.55 – 7.47 (m, 1H), 7.32 – 7.22 (m, 2H), 6.25 (d, *J* = 0.6 Hz, 1H), 2.94 (s, 2H), 1.50 (s, 6H).

^{13}C NMR (75 MHz, Chloroform-*d*): δ 170.5, 153.4, 135.3, 130.1, 124.1, 123.4, 120.7, 113.9, 97.9, 50.9, 34.6, 29.5.

IR: υ = 2963, 2929, 2870, 1733, 1587, 1453, 1379, 1274, 1211, 1166, 1025, 981, 898, 797, 749 cm^{-1}.

HRMS: (EI): calculated: m/z = 199.0992 $[M]^{\cdot+}$; found: m/z = 199.0992 $[M]^{\cdot+}$.

8,9-dihydropyrido[1,2-a]indol-6(7H)-one (6e):

Obtained *via* GP3.

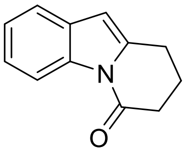

^{1}H NMR (300 MHz, Chloroform-*d*) δ 8.48 – 8.42 (m, 1H), 7.49 – 7.44 (m, 1H), 7.32 – 7.22 (m, 2H), 6.32 (dd, *J* = 2.0, 1.3 Hz, 1H), 3.02 – 2.94 (m, 2H), 2.78 (t, *J* = 6.5 Hz, 2H), 2.13 – 2.03 (m, 2H).

^{13}C NMR (75 MHz, Chloroform-*d*): δ 169.5, 138.1, 134.9, 129.8, 124.1, 124.0, 119.7, 116.4, 104.9, 34.5, 23.8, 21.5.

IR: υ = 2948, 2877, 2117, 1699, 1595, 1458, 1353, 1174, 1132, 1002, 801, 753, 682 cm^{-1}.

HRMS: (EI): calculated: m/z = 185.0835 $[M]^{\cdot+}$; found: m/z = 185.0837 $[M]^{\cdot+}$.

m.p.: 72-73°C.

8-methyl-8,9-dihydropyrido[1,2-a]indol-6(7H)-one (6f):

Obtained *via* GP3.

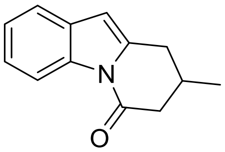

^{1}H NMR (300 MHz, Chloroform-*d*) δ 8.49 – 8.40 (m, 1H), 7.50 – 7.43 (m, 1H), 7.32 – 7.22 (m, 2H), 6.32 – 6.30 (m, 1H), 3.09 (dddd, *J* = 15.8, 3.9, 1.9, 0.9 Hz, 1H), 2.83 (ddd, *J* = 16.7, 3.7, 1.9 Hz, 1H), 2.59 (ddd, *J* = 15.7, 10.3, 1.5 Hz, 1H), 2.51 – 2.40 (m, 1H), 2.40 – 2.25 (m, 1H), 1.14 (d, *J* = 6.4 Hz, 3H).

^{13}C NMR (75 MHz, Chloroform-*d*): δ 169.2, 137.6, 134.8, 130.0, 124.0, 123.9, 119.7, 116.3, 105.1, 42.2, 31.7, 28.8, 20.6.

IR: υ = 3049, 2955, 2926, 2870, 1700, 1595, 1453, 1349, 1271, 1203, 1133, 1066, 1010, 793, 745, 674 cm^{-1}.

HRMS: (EI): calculated: m/z = 199.0991 $[M]^{\cdot+}$; found: m/z = 199.0992 $[M]^{\cdot+}$.

m.p.: 93-94°C.

<u>8,8-dimethyl-8,9-dihydropyrido[1,2-a]indol-6(7H)-one (6g):</u>

Obtained *via* GP3.

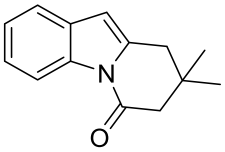

^{1}H NMR (300 MHz, Chloroform-*d*) δ 8.48 – 8.42 (m, 1H), 7.50 – 7.45 (m, 1H), 7.33 – 7.22 (m, 2H), 6.33 (dd, *J* = 2.0, 1.3 Hz, 1H), 2.80 (s, 2H), 2.60 (s, 2H), 1.10 (s, 6H).

^{13}C NMR (75 MHz, Chloroform-*d*): δ 168.9, 137.2, 134.8, 130.1, 124.1, 123.9, 119.7, 116.4, 105.9, 47.9, 37.4, 33.0, 27.7.

IR: υ = 2959, 2870, 2117, 1703, 1595, 1453, 1353, 1323, 1203, 1159, 1066, 939, 812,n 752, 670 cm^{-1}.

HRMS: (EI): calculated: m/z = 213.1148 $[M]^{\cdot+}$; found: m/z = 213.1148 $[M]^{\cdot+}$.

m.p.: 97-98°C.

<u>7-methoxy-1,2-dihydro-3H-pyrrolo[1,2-a]indol-3-one (6h):</u>

Obtained *via* GP3.

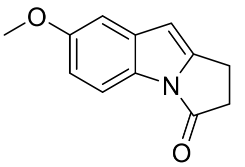

^{1}H NMR (300 MHz, Chloroform-*d*) δ 7.93 (d, *J* = 8.8 Hz, 1H), 6.96 (d, *J* = 2.4 Hz, 1H), 6.86 (dd, *J* = 8.8, 2.5 Hz, 1H), 6.21 – 6.19 (m, 1H), 3.84 (s, 3H), 3.15 – 3.09 (m, 2H), 3.07 – 3.01 (m, 2H).

^{13}C NMR (75 MHz, Chloroform-*d*): δ 171.3, 156.8, 144.6, 136.4, 125.2, 114.2, 111.5, 103.6, 100.4, 55.7, 34.7, 19.7.

IR: υ = 2937, 2836, 1718, 1587, 1476, 1438, 1394, 1356, 1327, 1259, 1196, 1162, 1129, 842, 812, 711 cm^{-1}.

HRMS: (EI): calculated: m/z = 201.0784 $[M]^{\cdot+}$; found: m/z = 201.0780 $[M]^{\cdot+}$.

m.p.: 141-142°C.

7-methyl-1,2-dihydro-3H-pyrrolo[1,2-a]indol-3-one (6i):

Obtained *via* GP3.

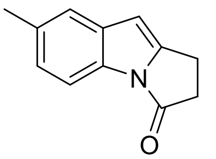

^{1}H NMR (300 MHz, Chloroform-*d*) δ 7.94 (d, *J* = 8.2 Hz, 1H), 7.30 – 7.27 (m, 1H), 7.09 (dd, *J* = 8.2, 1.0 Hz, 1H), 6.22 – 6.18 (m, 1H), 3.16 – 3.09 (m, 2H), 3.09 – 3.02 (m, 2H), 2.44 (s, 3H).

^{13}C NMR (75 MHz, Chloroform-*d*): δ 171.5, 143.8, 135.6, 133.7, 128.6, 124.5, 120.5, 113.2, 100.1, 34.8, 21.7, 19.6.

IR: υ = 3026, 2933, 2866, 1714, 1587, 1461, 1390, 1349, 1297, 1226, 1162, 1051, 879, 808, 741, 708 cm^{-1}.

HRMS: (EI): calculated: m/z = 185.0835 $[M]^{\cdot+}$; found: m/z = 185.0837 $[M]^{\cdot+}$.

m.p.: 113-114°C.

1,8-dimethyl-8,9-dihydropyrido[1,2-a]indol-6(7H)-one (6j):

Obtained *via* GP3.

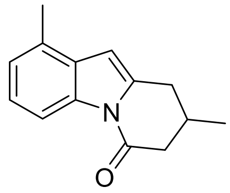

^{1}H NMR (300 MHz, Chloroform-*d*) δ 8.28 (d, *J* = 8.2 Hz, 1H), 7.19 (t, *J* = 7.8 Hz, 1H), 7.06 (dd, *J* = 6.6, 0.7 Hz, 1H), 6.37 – 6.35 (m, 1H), 3.16 – 3.06 (m, 1H), 2.83 (ddd, *J* = 16.7, 3.7, 1.9 Hz, 1H), 2.61 (ddd, *J* = 15.6, 10.2, 1.4 Hz, 1H), 2.52 – 2.40 (dd, *J* = 16.7, 10.3 Hz, 1H), 2.48 (s, 3H), 2.40 – 2.27 (m, 1H), 1.15 (d, *J* = 6.4 Hz, 3H).

^{13}C NMR (75 MHz, Chloroform-*d*): δ 169.2, 137.0, 134.5, 129.4, 129.1, 124.4, 124.1, 113.9, 103.6, 42.3, 31.8, 28.8, 20.6, 18.5.

IR: υ = 2955, 2926, 2873, 1703, 1580, 1490, 1453, 1420, 1349, 1326, 1230, 1133, 1073, 775, 682 cm^{-1}.

HRMS: (EI): calculated: m/z = 213.1148 $[M]^{\cdot+}$; found: m/z = 213.1151 $[M]^{\cdot+}$.

m.p.: 112-113°C.

methyl 3-oxo-2,3-dihydro-1H-pyrrolo[1,2-a]indole-7-carboxylate (6k):

Obtained *via* GP3.

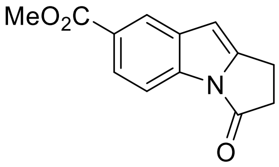

^{1}H NMR (300 MHz, Chloroform-*d*) δ 8.23 (dd, *J* = 1.5, 0.5 Hz, 1H), 8.08 (dt, *J* = 8.5, 0.7 Hz, 1H), 7.99 – 7.95 (m, 1H), 6.36 – 6.33 (m, *J* = 0.7 Hz, 1H), 3.93 (s, 3H), 3.23 – 3.16 (m, 2H), 3.14 – 3.08 (m, 2H).

^{13}C NMR (75 MHz, Chloroform-*d*): δ 171.7, 167.5, 144.8, 135.1, 132.9, 125.9, 124.7, 122.8, 113.2, 100.8, 52.1, 34.7, 19.7.

IR: υ = 3104, 2952, 1736, 1710, 1610, 1580, 1476, 1438, 1386, 1282, 1237, 1170, 1133, 1088, 1047, 984, 820, 767, 734 cm^{-1}.

HRMS: (EI): calculated: m/z = 229.0733 $[M]^{\cdot+}$; found: m/z = 229.0734 $[M]^{\cdot+}$.

m.p.: 149-150°C.

methyl 6-oxo-6,7,8,9-tetrahydropyrido[1,2-a]indole-3-carboxylate (6l):

Obtained *via* GP3.

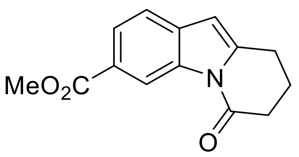

^{1}H NMR (300 MHz, Chloroform-*d*) δ 9.13 – 9.05 (m, 1H), 7.94 (dd, *J* = 8.2, 1.5 Hz, 1H), 7.46 (d, *J* = 8.2 Hz, 1H), 6.38 – 6.30 (m, *J* = 0.6 Hz, 1H), 3.93 (s, 3H), 3.00 (t, *J* = 5.8 Hz, 2H), 2.85 – 2.76 (m, 2H), 2.16 – 2.05 (m, 2H).

^{13}C NMR (75 MHz, Chloroform-*d*): δ 169.2, 167.7, 141.6, 134.3, 133.6, 125.7, 125.4, 119.3, 117.9, 104.8, 52.0, 34.4, 23.9, 21.3.

IR: υ = 2948, 1707, 1610, 1435, 1353, 1274, 1203, 1118, 1077, 1013, 972, 909, 846, 745 cm^{-1}.

HRMS: (EI): calculated: m/z = 243.0890 [M]$^{\cdot+}$; found: m/z = 243.0890 [M]$^{\cdot+}$.

m.p.: 163-164°C.

6-chloro-1,2-dihydro-3H-pyrrolo[1,2-a]indol-3-one (6m):

Obtained *via* GP3.

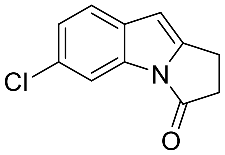

^{1}H NMR (300 MHz, Chloroform-*d*) δ 8.04 (d, *J* = 1.8 Hz, 1H), 7.38 (d, *J* = 8.4 Hz, 1H), 7.21 (dd, *J* = 8.4, 1.9 Hz, 1H), 6.27 – 6.20 (m, 1H), 3.20 – 3.03 (m, 4H).

^{13}C NMR (75 MHz, Chloroform-*d*): δ 171.5, 144.1, 133.7, 130.5, 129.0, 124.5, 121.3, 113.8, 100.1, 34.7, 19.7.

IR: υ = 3082, 2937, 1729, 1587, 1565, 1438, 1353, 1319, 1207, 1162, 1118, 1047, 961, 872, 820, 708 cm^{-1}.

HRMS: (EI): calculated: m/z = 205.0290 [M]$^{\cdot+}$; found: m/z = 205.0290 [M]$^{\cdot+}$.

m.p.: 144-145°C.

2-chloro-8,8-dimethyl-8,9-dihydropyrido[1,2-a]indol-6(7H)-one (6n):

Obtained *via* GP3.

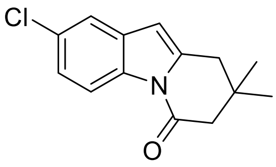

^{1}H NMR (300 MHz, Chloroform-*d*) δ 8.34 (d, *J* = 8.7 Hz, 1H), 7.42 (d, *J* = 2.0 Hz, 1H), 7.22 (dd, *J* = 8.7, 2.1 Hz, 1H), 6.28 – 6.24 (m, 1H), 2.80 (s, 2H), 2.60 (s, 2H), 1.09 (s, 6H).
^{13}C NMR (75 MHz, Chloroform-*d*): δ 168.8, 138.7, 133.1, 131.4, 129.4, 124.1, 119.4, 117.2, 105.2, 47.7, 37.4, 33.1, 27.7.
IR: υ = 2948, 1707, 1610, 1435, 1353, 1274, 1203, 1118, 1077, 1013, 972, 909, 846, 745 cm^{-1}.
HRMS: (EI): calculated: m/z = 247.0754 [M]$^{\cdot+}$; found: m/z = 247.0754 [M]$^{\cdot+}$.
m.p.: 98-99°C.

7-bromo-1,2-dihydro-3H-pyrrolo[1,2-a]indol-3-one (6o):

Obtained *via* GP3.

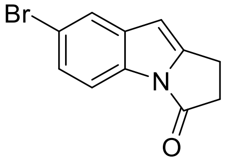

^{1}H NMR (300 MHz, Chloroform-*d*) δ 7.90 (d, *J* = 8.5 Hz, 1H), 7.61 (d, *J* = 1.8 Hz, 1H), 7.35 (dd, *J* = 8.5, 1.8 Hz, 1H), 6.22 – 6.18 (m, 1H), 3.19 – 3.12 (m, 2H), 3.11 – 3.03 (m, 2H).
^{13}C NMR (75 MHz, Chloroform-*d*): δ 171.5, 145.0, 136.9, 129.0, 126.1, 123.3, 117.3, 114.8, 99.7, 34.6, 19.7.
IR: υ = 2940, 1729, 1602, 1390, 1349, 1146, 1349, 1162, 1043, 939, 875, 812, 738 cm^{-1}.
HRMS: (EI): calculated: m/z = 248.9784 [M]$^{\cdot+}$; found: m/z = 248.9780 [M]$^{\cdot+}$.
m.p.: 138-139°C.

(5aS,9aS)-5a,6,7,8,9,9a-hexahydro-5H-pyrrolo[2,1-a]isoindol-5-one (6p):

Obtained *via* GP3.

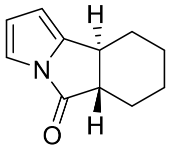

^{1}H NMR (300 MHz, Chloroform-*d*) δ 7.00 (dd, *J* = 3.1, 0.9 Hz, 1H), 6.41 (t, *J* = 3.1 Hz, 1H), 5.94 (dt, *J* = 2.9, 1.0 Hz, 1H), 3.33 (q, *J* = 6.8 Hz, 1H), 3.14 (dd, *J* = 12.6, 7.0 Hz, 1H), 2.07 – 1.91 (m, 2H), 1.90 – 1.79 (m, 1H), 1.60 – 1.30 (m, 5H).

^{13}C NMR (75 MHz, Chloroform-*d*): δ 174.3, 143.9, 118.0, 110.9, 103.6, 46.5, 32.7, 29.0, 23.4, 21.5, 21.3.

IR: υ = 2933, 2858, 1744, 1572, 1468, 1397, 1267, 1058, 879, 827, 797, 711 cm^{-1}.

HRMS: (EI): calculated: m/z = 175.0991 $[M]^{\cdot+}$; found: m/z = 175.0987 $[M]^{\cdot+}$.

9-methyl-1,2-dihydro-3H-pyrrolo[1,2-a]indol-3-one (6q):

Obtained *via* GP3.

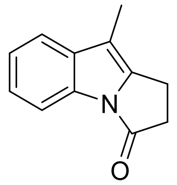

^{1}H NMR (300 MHz, Chloroform-*d*) δ 8.06 – 8.00 (m, 1H), 7.46 – 7.40 (m, 1H), 7.32 – 7.23 (m, 2H), 3.05 – 3.00 (m, 4H), 2.17 (s, 3H).

^{13}C NMR (75 MHz, Chloroform-*d*): δ 171.4, 139.1, 136.2, 130.3, 123.7, 123.2, 118.5, 113.5, 108.7, 34.9, 18.4, 8.3.

IR: υ = 2937, 2858, 2117, 1729, 1632, 1442, 1397, 1353, 1312, 1226, 1185, 1129, 1088, 797, 745, 705, 667 cm^{-1}.

HRMS: (EI): calculated: m/z = 185.0835 $[M]^{\cdot+}$; found: m/z = 185.0838 $[M]^{\cdot+}$.

m.p.: 172-173°C.

10-methyl-8,9-dihydropyrido[1,2-a]indol-6(7H)-one (6r):

Obtained *via* GP3.

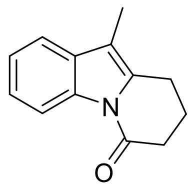

^{1}H NMR (300 MHz, Chloroform-*d*) δ 8.48 – 8.42 (m, 1H), 7.45 – 7.39 (m, 1H), 7.33 – 7.24 (m, 2H), 2.89 (t, *J* = 6.1 Hz, 2H), 2.79 – 2.72 (m, 2H), 2.18 (m, 3H), 2.12 – 2.03 (m, 2H).

^{13}C NMR (75 MHz, Chloroform-*d*): δ 169.3, 134.5, 133.2, 131.1, 124.2, 123.7, 117.8, 116.3, 112.2, 34.5, 21.8, 21.2, 8.5.

IR: υ = 3049, 2944, 2877, 1695, 1625, 1453, 1360, 1334, 1259, 1174, 1129, 1058, 954, 857, 827, 745, 600 cm^{-1}.

HRMS: (EI): calculated: m/z = 199.0992 $[M]^{\cdot+}$; found: m/z = 199.0995 $[M]^{\cdot+}$.

m.p.: 79-80°C.

methyl 2-(8,8-dimethyl-6-oxo-6,7,8,9-tetrahydropyrido[1,2-a]indol-10-yl)acetate (6s):

Obtained *via* GP3.

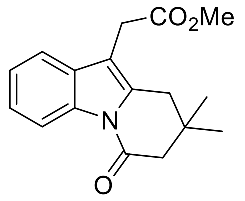

^{1}H NMR (300 MHz, Chloroform-*d*) δ 8.49 – 8.41 (m, 1H), 7.52 – 7.46 (m, 1H), 7.34 – 7.27 (m, 2H), 3.68 (s, 3H), 3.65 (s, 2H), 2.80 (s, 2H), 2.62 (s, 2H), 1.12 (s, 6H).

^{13}C NMR (75 MHz, Chloroform-*d*): δ 171.2, 168.8, 134.7, 134.3, 130.1, 124.5, 124.0, 118.0, 116.4, 110.5, 52.2, 47.8, 35.5, 32.7, 29.9, 27.9.

IR: υ = 2955, 1736, 1699, 1621, 1457, 1364, 1330, 1263, 1162, 1099, 1028, 753 cm^{-1}.

HRMS: (EI): calculated: m/z = 285.1346 $[M]^{\cdot+}$; found: m/z = 285.1351 $[M]^{\cdot+}$.

N-(2-(2-methoxy-6-oxo-6,7,8,9-tetrahydropyrido[1,2-a]indol-10-yl)ethyl)acetamide (6t):

Obtained *via* GP3.

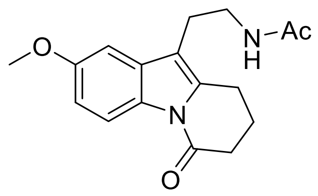

^{1}H NMR (300 MHz, Chloroform-*d*) δ 8.28 (dd, *J* = 8.9, 0.4 Hz, 1H), 6.92 (d, *J* = 2.4 Hz, 1H), 6.86 (dd, *J* = 8.9, 2.5 Hz, 1H), 6.00 – 5.91 (m, 1H), 3.84 (s, 3H), 3.46 (q, *J* = 6.6 Hz, 2H), 2.88 – 2.79 (m, 4H), 2.63 – 2.57 (m, 2H), 2.06 – 1.97 (m, 2H), 1.93 (s, 3H).

^{13}C NMR (75 MHz, Chloroform-*d*): δ 170.3, 168.9, 156.9, 135.6, 131.3, 129.2, 117.2, 113.6, 111.9, 101.4, 55.8, 39.2, 34.0, 24.0, 23.3, 21.8, 21.3.

IR: υ = 3298, 2937, 1692, 1651, 1548, 1476, 1371, 1334, 1289, 1237, 1203, 1177, 1110, 1043, 909, 812, 730 cm^{-1}.

HRMS: (ESI): calculated: m/z = 301.1547 $[M + H]^+$; found: m/z = 301.1552 $[M + H]^+$.

ethyl (S)-2-acetamido-3-(6-oxo-6,7,8,9-tetrahydropyrido[1,2-a]indol-10-yl)propanoate (6u):

Obtained *via* GP3.

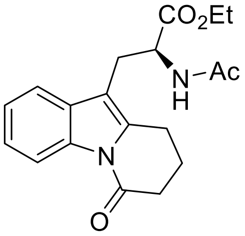

^{1}H NMR (300 MHz, Chloroform-*d*) δ 8.47 – 8.40 (m, 1H), 7.44 – 7.37 (m, 1H), 7.32 – 7.22 (m, 2H), 6.16 (d, *J* = 7.8 Hz, 1H), 4.87 (dt, *J* = 7.8, 5.9 Hz, 1H), 4.15 (dq, *J* = 10.8, 7.2 Hz, 1H), 4.00 (dq, *J* = 10.8, 7.2 Hz, 1H), 3.23 – 3.17 (m, 2H), 2.88 (dd, *J* = 7.3, 5.2 Hz, 2H), 2.74 (dd, *J* = 5.9, 6.9 Hz, 2H), 2.10 – 2.00 (m, 2H), 1.95 (s, 3H), 1.14 (t, *J* = 7.1 Hz, 3H).

^{13}C NMR (75 MHz, Chloroform-*d*): δ 171.9, 169.7, 169.3, 135.7, 134.5, 130.4, 124.5, 123.9, 117.8, 116.5, 111.3, 61.8, 52.4, 34.4, 26.9, 23.2, 22.0, 21.3, 13.9.

IR: υ = 3295, 2937, 1736, 1703, 1654, 1539, 1461, 1371, 1334, 1177, 1133, 1025, 752 cm^{-1}.

HRMS: (ESI): calculated: m/z = 343.1652 $[M + H]^{+}$; found: m/z = 343.1655 $[M + H]^{+}$.

m.p.: 105-106°C.

4-methyl-N-(2-(3-oxo-2,3-dihydro-1H-pyrrolo[1,2-a]indol-9-yl)ethyl)benzenesulfonamide (6v):

Obtained *via* GP3.

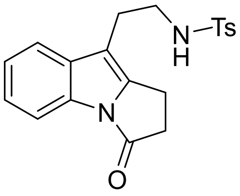

^{1}H NMR (300 MHz, DMSO-*d*) δ 7.89 – 7.82 (m, 1H), 7.65 (t, *J* = 5.9 Hz, 1H), 7.58 – 7.53 (m, 2H), 7.45 – 7.38 (m, 1H), 7.30 – 7.20 (m, 4H), 3.10 – 2.97 (m, 6H), 2.71 (t, *J* = 6.9 Hz, 2H), 2.34 (s, 3H).

^{13}C NMR (75 MHz, DMSO-*d*): δ 172.2, 142.9, 142.0, 138.2, 135.3, 130.0, 129.8, 126.7, 123.9, 123.2, 119.0, 113.1, 109.1, 42.5, 34.8, 24.5, 21.4, 18.9.

IR: υ = 3239, 2922, 2855, 1729, 1625, 1461, 1397, 1312, 1159, 1073, 935, 816, 760, 723, 667 cm^{-1}.

HRMS: (ESI): calculated: m/z = 369.1267 $[M + H]^+$; found: m/z = 369.1267 $[M + H]^+$.

m.p.: 191-192°C.

9-methyl-2,3-dihydro-1H-pyrrolo[1,2-a]indole (8):

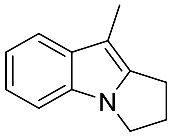

^{1}H NMR (300 MHz, Chloroform-*d*) δ 7.53 – 7.47 (m, 1H), 7.24 – 7.19 (m, 1H), 7.10 (dqd, *J* = 14.3, 7.0, 1.4 Hz, 2H), 4.04 t, *J* = 6.9 Hz, 2H), 2.95 (t, *J* = 7.2 Hz, 2H), 2.65 – 2.54 (m, 2H), 2.28 (t, *J* = 0.8 Hz, 3H).

^{13}C NMR (75 MHz, Chloroform-*d*): δ 141.3, 133.1, 132.5, 120.0, 118.4, 109.1, 100.7, 43.6, 27.9, 22.9, 9.0.

IR: υ = 3049, 2952, 2877, 1740, 1461, 1379, 1297, 1233, 738 cm^{-1}.

HRMS: (EI): calculated: m/z = 170.0964 $[M]^{\cdot+}$; found: m/z = 170.0969 $[M]^{\cdot+}$.

3-(3-methyl-1H-indol-2-yl)-1-(pyrrolidin-1-yl)propan-1-one (9):

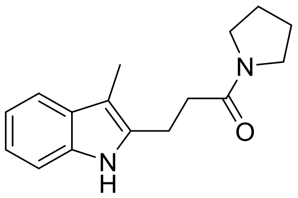

^{1}H NMR (300 MHz, Chloroform-*d*) δ 9.22 (s, 1H), 7.51 – 7.46 (m, 1H), 7.31 – 7.27 (m, 1H), 7.14 – 7.02 (m, 2H), 3.50 (t, *J* = 6.7 Hz, 2H), 3.31 (t, *J* = 6.6 Hz, 2H), 3.12 – 3.05 (m, 2H), 2.63 – 2.55 (m, 2H), 2.26 (s, 3H), 1.97 – 1.81 (m, 4H).

^{13}C NMR (75 MHz, Chloroform-*d*): δ 171.5, 135.2, 135.1, 128.9, 120.9, 118.6, 118.0, 110.6, 106.0, 46.5, 45.9, 34.9, 26.0, 24.4, 20.1, 8.5.

IR: υ = 3395, 3272, 2970, 2922, 2873, 1617, 1446, 1338, 1237, 1192, 913, 738 cm^{-1}.

HRMS: (ESI): calculated: m/z = 257.1648 $[M+H]^+$; found: m/z = 257.1650 $[M+H]^+$.

m.p.: 163-164°C.

9-methyl-1,2,9,9a-tetrahydro-3H-pyrrolo[1,2-a]indol-3-one (10):

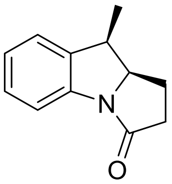

^{1}H NMR (300 MHz, Chloroform-*d*) δ 7.60 (d, *J* = 7.7 Hz, 1H), 7.24 – 7.16 (m, 2H), 7.04 (td, *J* = 7.5, 1.1 Hz, 1H), 4.69 (q, *J* = 8.3 Hz, 1H), 3.25 (p, *J* = 7.4 Hz, 1H), 2.95 – 2.81 (m, 1H), 2.69 – 2.56 (m, 1H), 2.18 – 2.06 (m, 2H), 1.11 (d, *J* = 7.3 Hz, 3H).

^{13}C NMR (75 MHz, Chloroform-*d*): δ 171.1, 140.5, 137.6, 127.8, 124.9, 124.3, 114.5, 65.5, 36.9, 36.6, 22.3, 16.9.

IR: υ = 2967, 2873, 1692, 1602, 1483, 1405, 1304, 1215, 1159, 1118, 756 cm^{-1}.

HRMS: (EI): calculated: m/z = 187.0992 $[M]^{\cdot+}$; found: m/z = 187.0992 $[M]^{\cdot+}$.

m.p.: 79-80°C.

<u>11b-bromo-3-tosyl-1,2,3,4,5,11b-hexahydro-6H-dipyrrolo[1,2-a:2',3'-b]indol-6-one (11):</u>

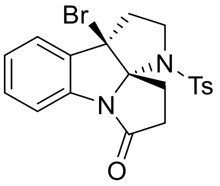

^{1}H NMR (300 MHz, Chloroform-*d*) δ 7.49 (d, *J* = 8.3 Hz, 2H), 7.25 – 7.19 (m, 1H), 7.13 (d, *J* = 8.0 Hz, 2H), 7.07 – 6.95 (m, 3H), 3.81 – 3.66 (m, 1H), 3.46 (ddd, *J* = 17.7, 10.3, 7.4 Hz, 1H), 3.13 (ddd, *J* = 14.4, 11.0, 7.4 Hz, 1H), 2.98 – 2.59 (m, 5H), 2.33 (s, 3H).

^{13}C NMR (75 MHz, Chloroform-*d*): δ 173.8, 143.5, 137.3, 135.0, 134.3, 130.0, 129.4, 127.3, 125.9, 123.3, 116.4, 95.5, 70.2, 48.2, 37.0, 33.4, 31.3, 21.4.

IR: υ = 2952, 2877, 1714, 1602, 1479, 1367, 1162, 1092, 1006, 920, 730, 663 cm^{-1}.

HRMS: (ESI): calculated: m/z = 447.0363 $[M+H]^{+}$; found: m/z = 447.0373 $[M+H]^{+}$.

m.p.: 117-118°C.

<u>6-oxo-7,8,9,10-tetrahydro-6H-azepino[1,2-a]indole-10-carbonitrile (13a):</u>

Obtained *via* GP4.

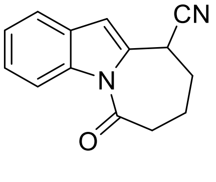

^{1}H NMR (300 MHz, Chloroform-*d*) δ 8.44 – 8.36 (m, 1H), 7.58 – 7.52 (m, 1H), 7.34 (dtd, *J* = 13.6, 7.3, 3.8 Hz, 2H), 6.88 – 6.83 (m, 1H), 4.35 (ddd, *J* = 10.2, 5.2, 1.0 Hz, 1H), 3.12 (ddd, *J* = 16.8, 7.4, 2.9 Hz, 1H), 2.87 (ddd, *J* = 16.8, 11.1, 3.2 Hz, 1H), 2.47 – 2.21 (m, 2H), 2.19 – 1.93 (m, 2H).

^{13}C NMR (75 MHz, Chloroform-*d*): δ 171.9, 137.4, 131.4, 128.3, 125.7, 124.3, 120.6, 118.7, 116.4, 110.5, 35.5, 29.3, 29.2, 19.6.

IR: υ = 2948, 1703, 1591, 1452, 1379, 1345, 1315, 1218, 1151, 946, 812, 753 cm^{-1}.

HRMS: (ESI): calculated: m/z = 225.1022 $[M+H]^{+}$; found: m/z = 225.1023 $[M+H]^{+}$.

m.p.: 114-115°C.

2-methyl-6-oxo-7,8,9,10-tetrahydro-6H-azepino[1,2-a]indole-10-carbonitrile (13b):

Obtained *via* GP4.

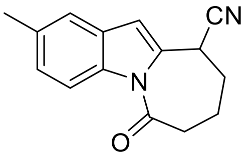

^{1}H NMR (300 MHz, Chloroform-*d*) δ 8.26 (d, *J* = 8.5 Hz, 1H), 7.35 – 7.30 (m, 1H), 7.18 (dd, *J* = 8.5, 1.4 Hz, 1H), 6.77 (s, *J* = 0.8 Hz, 1H), 4.32 (ddd, *J* = 10.2, 5.1, 1.0 Hz, 1H), 3.09 (ddd, *J* = 16.8, 7.3, 2.9 Hz, 1H), 2.85 (ddd, *J* = 16.7, 11.1, 3.2 Hz, 1H), 2.44 (s, *J* = 7.4 Hz, 3H), 2.45 – 2.19 (m, 2H), 2.18 – 1.89 (m, 2H).

^{13}C NMR (75 MHz, Chloroform-*d*): δ 171.7, 135.6, 133.9, 131.3, 128.6, 127.0, 120.5, 118.8, 116.1, 110.3, 35.4, 29.3, 29.2, 21.4, 19.6.

IR: υ = 2948, 2873, 2251, 1699, 1587, 1472, 1382, 1345, 1312, 1233, 1185, 946, 812, 730 cm^{-1}.

HRMS: (EI): calculated: m/z = 238.1101 $[M]^{\cdot+}$; found: m/z = 238.1100 $[M]^{\cdot+}$.

m.p.: 141-142°C.

2-methoxy-6-oxo-7,8,9,10-tetrahydro-6H-azepino[1,2-a]indole-10-carbonitrile (13c):

Obtained *via* GP4.

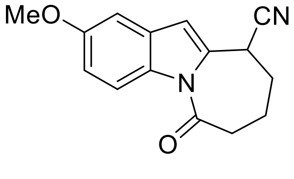

¹H NMR (300 MHz, Chloroform-*d*) δ 8.29 (d, *J* = 9.0 Hz, 1H), 6.99 (d, *J* = 2.3 Hz, 1H), 6.96 (dd, *J* = 9.0, 2.6 Hz, 1H), 6.78 (s, 1H), 4.31 (ddd, *J* = 10.3, 5.1, 0.9 Hz, 1H), 3.85 (s, 3H), 3.08 (ddd, *J* = 16.7, 7.3, 2.9 Hz, 1H), 2.84 (ddd, *J* = 16.7, 11.2, 3.2 Hz, 1H), 2.45 – 1.91 (m, 4H).
¹³C NMR (75 MHz, Chloroform-*d*): δ 171.5, 156.8, 132.1, 131.9, 129.3, 118.8, 117.3, 114.1, 110.4, 103.1, 55.7, 35.3, 29.2, 19.6.
IR: υ = 2944, 2251, 1695, 1613, 1476, 1446, 1379, 1312, 1230, 1203, 1140, 1110, 1032, 946, 849, 805, 771, 728 cm^{-1}.
HRMS: (EI): calculated: m/z = 254.1050 $[M]^{\cdot+}$; found: m/z = 245.1048 $[M]^{\cdot+}$.
m.p.: 119-120°C.

11-methyl-6-oxo-7,8,9,10-tetrahydro-6H-azepino[1,2-a]indole-10-carbonitrile (13d):

Obtained *via* GP4.

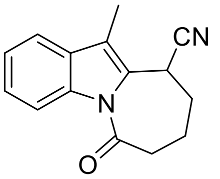

¹H NMR (300 MHz, Chloroform-*d*) δ 8.41 (d, *J* = 8.1 Hz, 1H), 7.53 – 7.47 (m, 1H), 7.42 – 7.36 (m, 1H), 7.35 – 7.28 (m, 1H), 4.58 (dd, *J* = 4.7, 3.8 Hz, 1H), 3.37 (ddd, *J* = 15.3, 11.7, 5.7 Hz, 1H), 2.96 (dt, *J* = 15.4, 4.3 Hz, 1H), 2.54 – 2.41 (m, 1H), 2.33 (s, 3H), 2.26 – 2.05 (m, 3H).
¹³C NMR (75 MHz, Chloroform-*d*): δ 171.7, 136.3, 129.4, 126.2, 126.2, 123.9, 119.0, 118.7, 118.6, 116.3, 36.2, 27.2, 26.2, 19.7, 9.2.
IR: υ = 2944, 2873, 2240, 1699, 1610, 1453, 1353, 1319, 1256, 1215, 1166, 1125, 1017, 842, 752 cm^{-1}.
HRMS: (EI): calculated: m/z = 238.1101 $[M]^{\cdot+}$; found: m/z = 238.1100 $[M]^{\cdot+}$.

N-(2-(10-cyano-6-oxo-7,8,9,10-tetrahydro-6H-azepino[1,2-a]indol-11-yl)ethyl)-4-methylbenzenesulfonamide (13e):

Obtained *via* GP4.

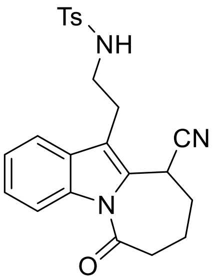

^{1}H NMR (300 MHz, Chloroform-*d*) δ 8.40 (d, *J* = 8.3 Hz, 1H), 7.65 (d, *J* = 8.3 Hz, 2H), 7.44 – 7.34 (m, 2H), 7.29 (dd, *J* = 7.5, 1.0 Hz, 1H), 7.22 (d, *J* = 8.0 Hz, 2H), 4.77 – 4.67 (m, 2H (including 1 N-H)), 3.43 – 3.15 (m, 3H), 3.10 – 2.88 (m, 3H), 2.50 – 2.41 (m, 1H), 2.39 (s, 3H), 2.29 – 2.01 (m, 3H).

^{13}C NMR (75 MHz, Chloroform-*d*): δ 171.9, 143.7, 136.5, 129.7, 128.1, 127.0, 126.3, 124.0, 119.0, 118.9, 118.4, 116.5, 42.5, 36.2, 27.4, 26.0, 25.7, 21.6, 19.8.

IR: υ = 3287, 2944, 1699, 1602, 1453, 1367, 1319, 1218, 1159, 1095, 913, 816, 752, 663 cm^{-1}.

HRMS: (ESI): calculated: m/z = 422.1533 [M+H]$^{+}$; found: m/z = 422.1541 [M+H]$^{+}$.

<u>8,8,11-trimethyl-6-oxo-7,8,9,10-tetrahydro-6H-azepino[1,2-a]indole-10-carbonitrile (13f):</u>

Obtained *via* GP4 using 12 equiv. of acrylonitrile (80 µL). The compound was obtained in 88% yield together with 7% of the corresponding compound **6** resulting from a direct intramolecular cyclization.

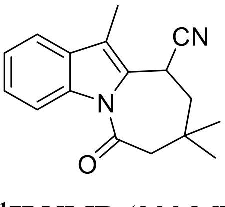

^{1}H NMR (300 MHz, Chloroform-*d*) δ 8.50 – 8.44 (m, 1H), 7.49 (ddd, *J* = 7.5, 1.5, 0.7 Hz, 1H), 7.42 – 7.29 (m, 2H), 4.49 (dd, *J* = 5.7, 2.9 Hz, 1H), 3.45 (d, *J* = 13.7 Hz, 1H), 2.66 (dd, *J* = 13.7, 0.9 Hz, 1H), 2.32 (s, 3H), 2.35 – 2.27 (ddd, *J* = 14.8, 5.7, 0.9 Hz, 1H), 1.93 (dd, *J* = 14.8, 2.9 Hz, 1H), 1.40 (s, 3H), 1.19 (s, 3H).

^{13}C NMR (75 MHz, Chloroform-*d*): δ 170.0, 136.1, 129.4, 126.9, 126.2, 124.0, 118.90, 118.80, 118.6, 116.6, 49.4, 40.2, 32.1, 30.7, 30.1, 25.3, 9.2.

IR: υ = 2963, 2933, 2873, 2236, 1699, 1606, 1453, 1356, 1326, 1263, 1155, 1121, 909, 749, 700 cm^{-1}.

HRMS: (EI): calculated: m/z = 266.1414 [M]$^{\cdot+}$; found: m/z = 266.1415 [M]$^{\cdot+}$.

Spectra

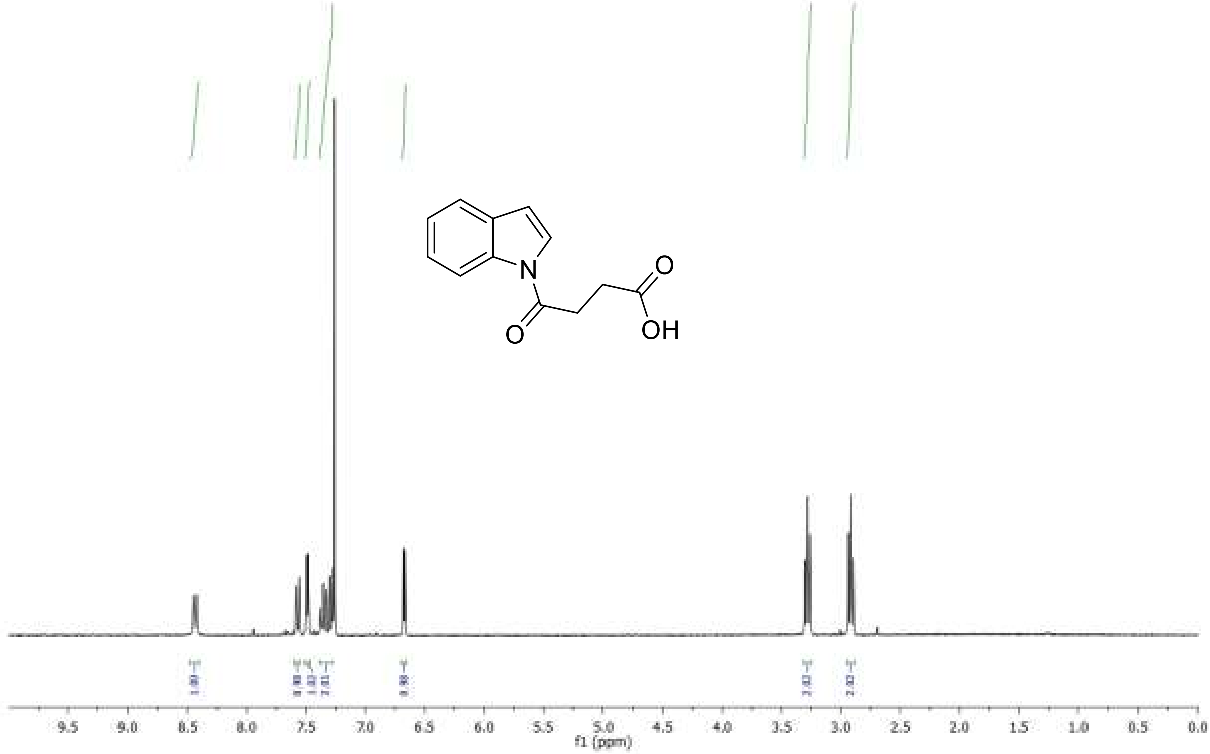
O
N
OH
O
f1 (ppm)

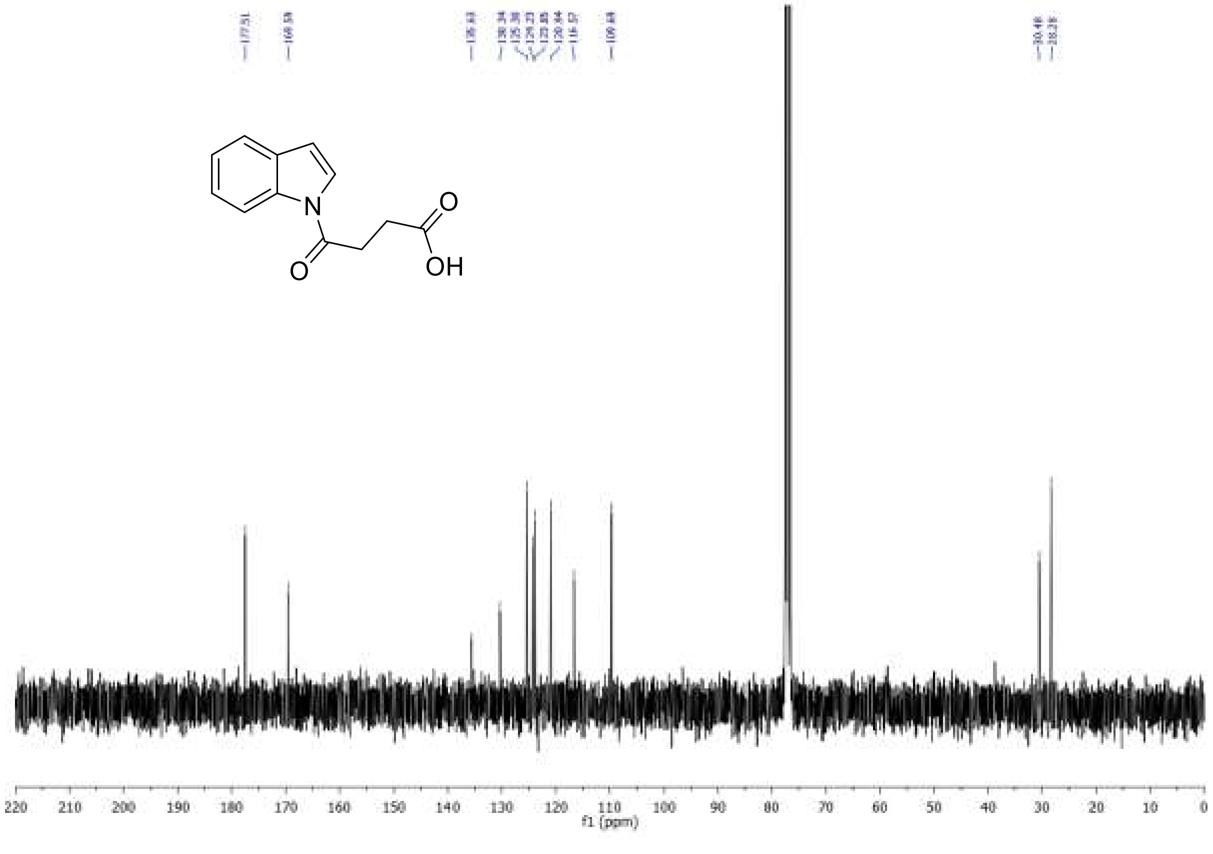
O
N
OH
O
f1 (ppm)

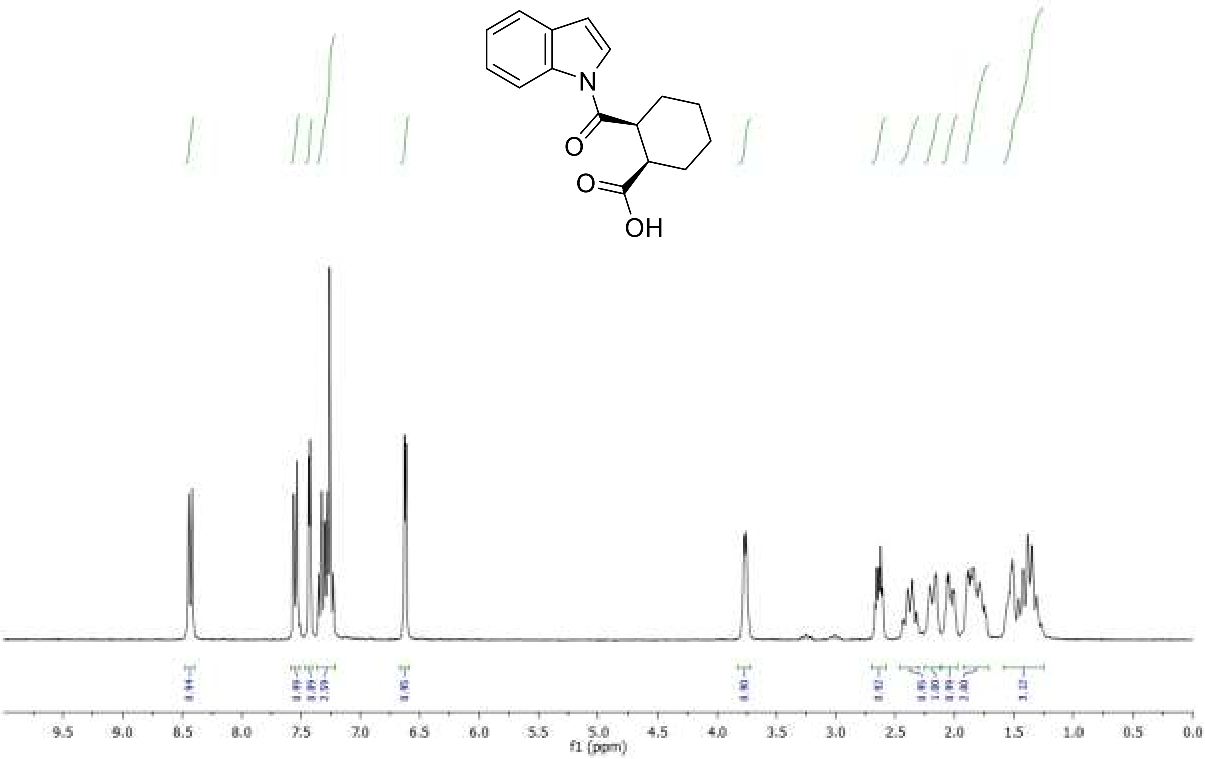
N
O
O
OH
9.5 9.0 8.5 8.0 7.5 7.0 6.5 6.0 5.5 5.0 4.5 4.0 3.5 3.0 2.5 2.0 1.5 1.0 0.5 0.0
f1 (ppm)

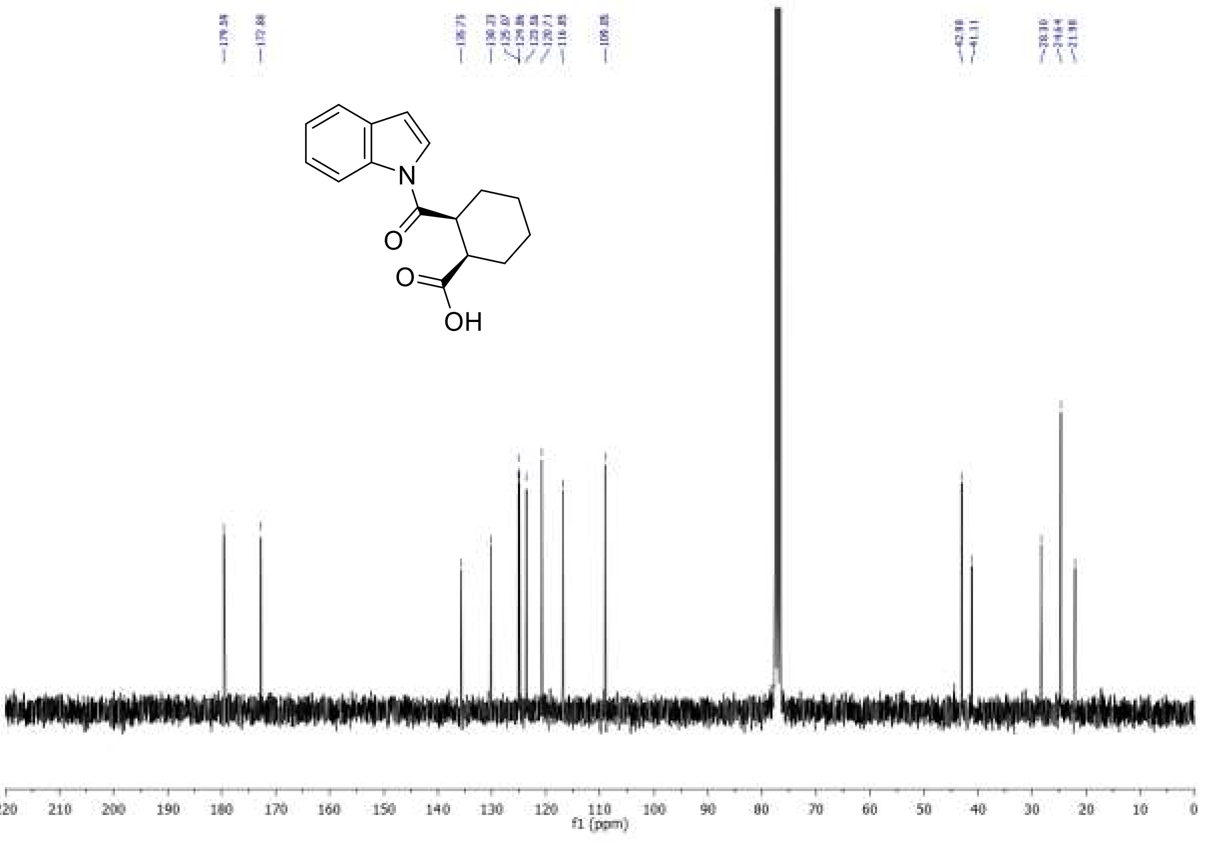
N
O
O
OH
220 210 200 190 180 170 160 150 140 130 120 110 100 90 80 70 60 50 40 30 20 10 0
f1 (ppm)

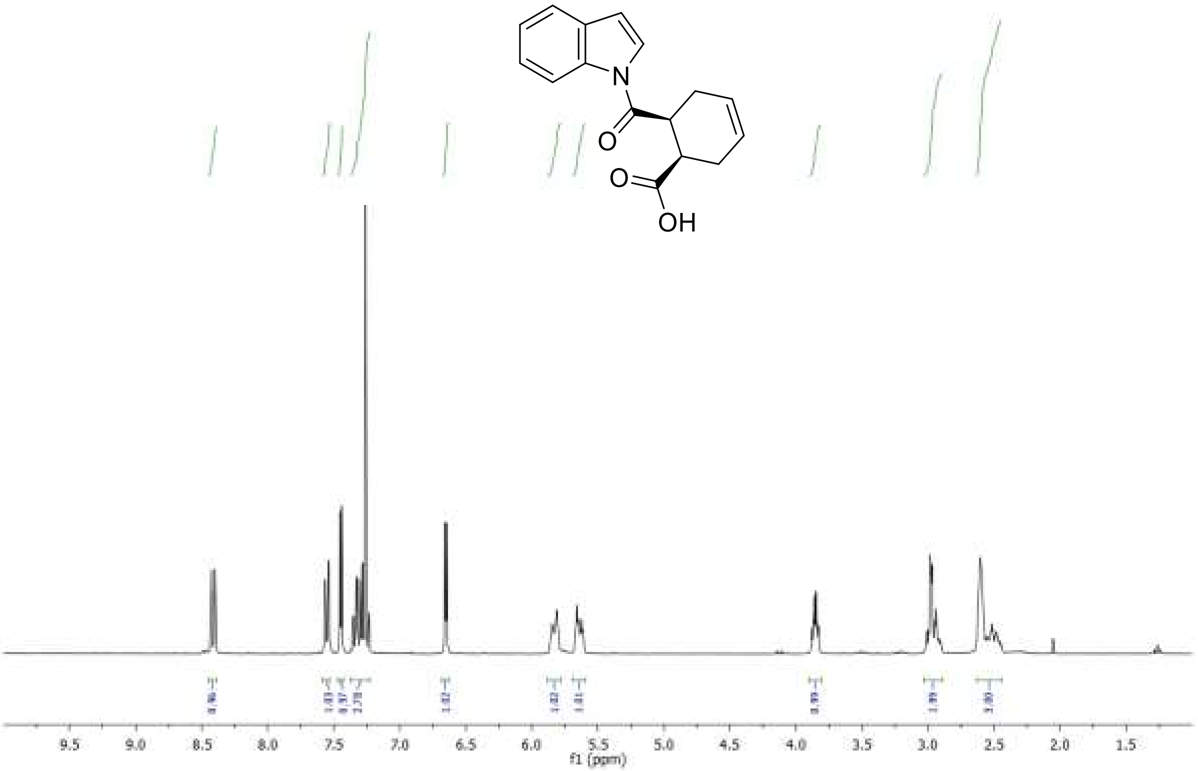
N
O
O
OH
f1 (ppm)

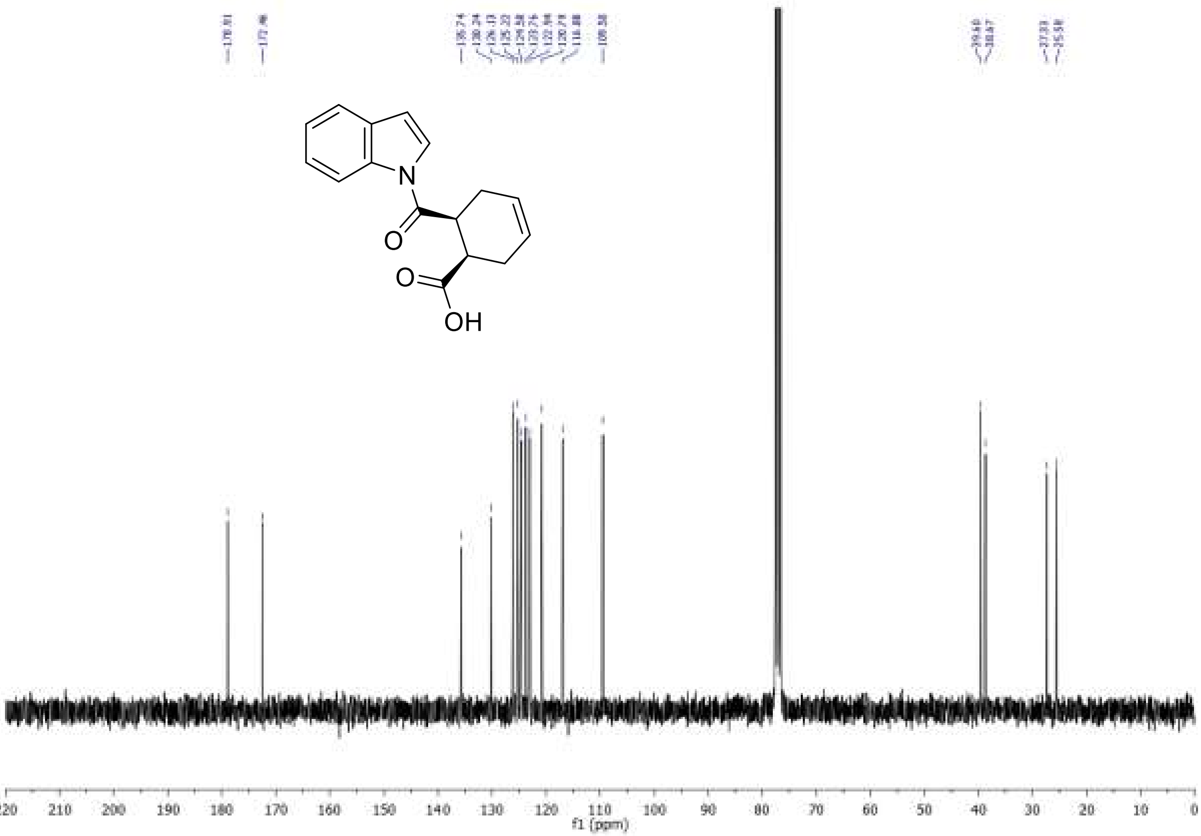
N
O
O
OH
f1 (ppm)

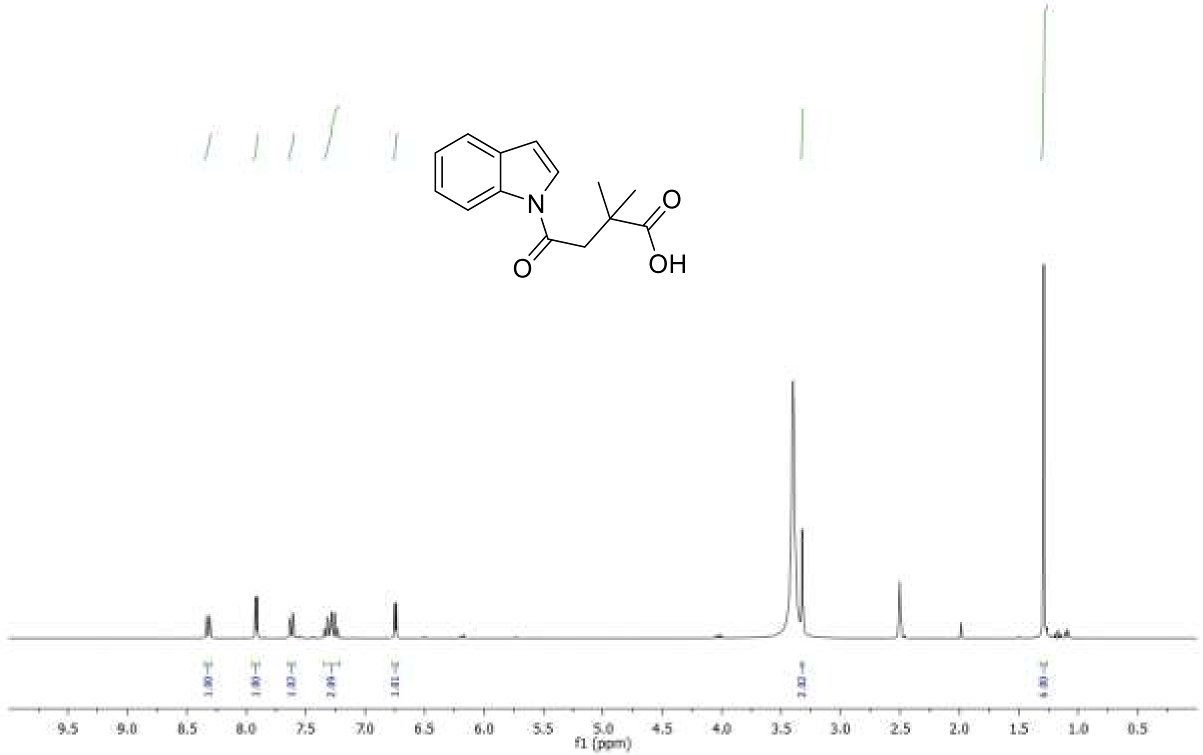
N
O
O
OH
9.5 9.0 8.5 8.0 7.5 7.0 6.5 6.0 5.5 5.0 4.5 4.0 3.5 3.0 2.5 2.0 1.5 1.0 0.5
f1 (ppm)

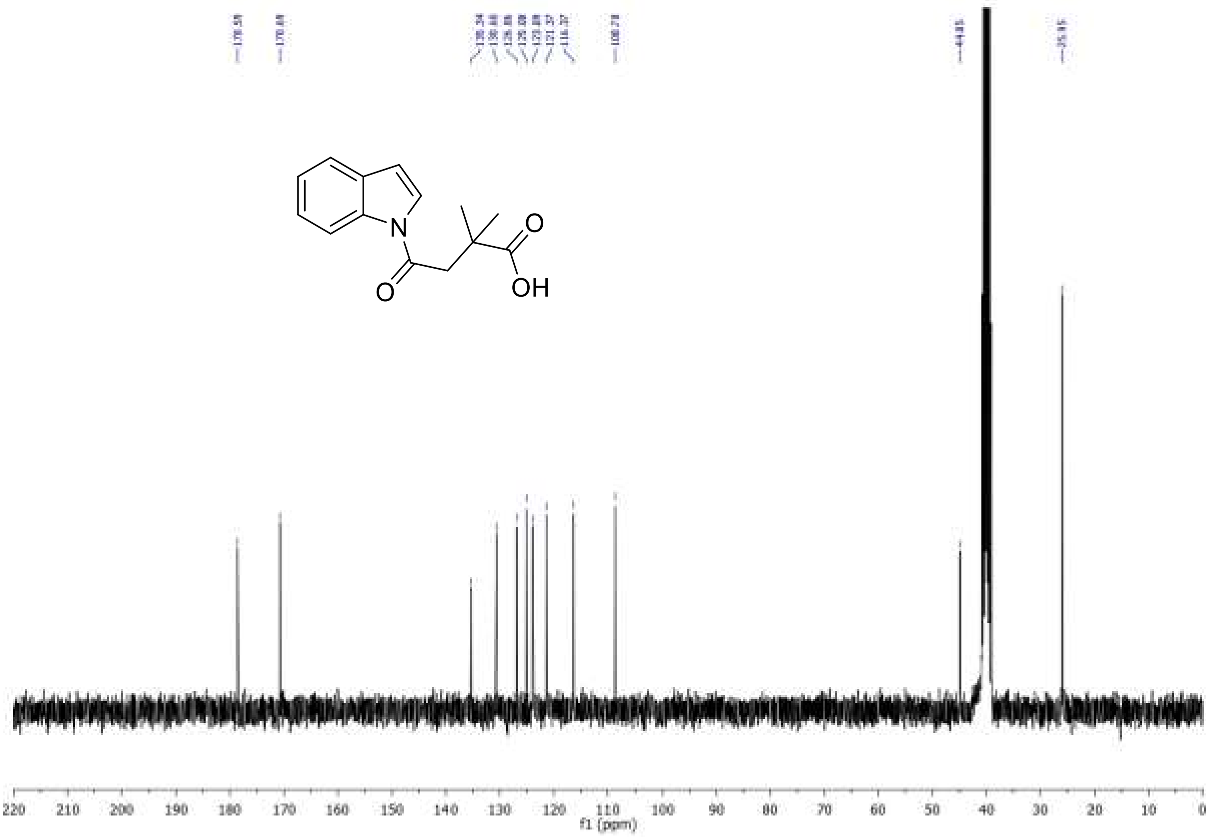
N
O
O
OH
220 210 200 190 180 170 160 150 140 130 120 110 100 90 80 70 60 50 40 30 20 10 0
f1 (ppm)

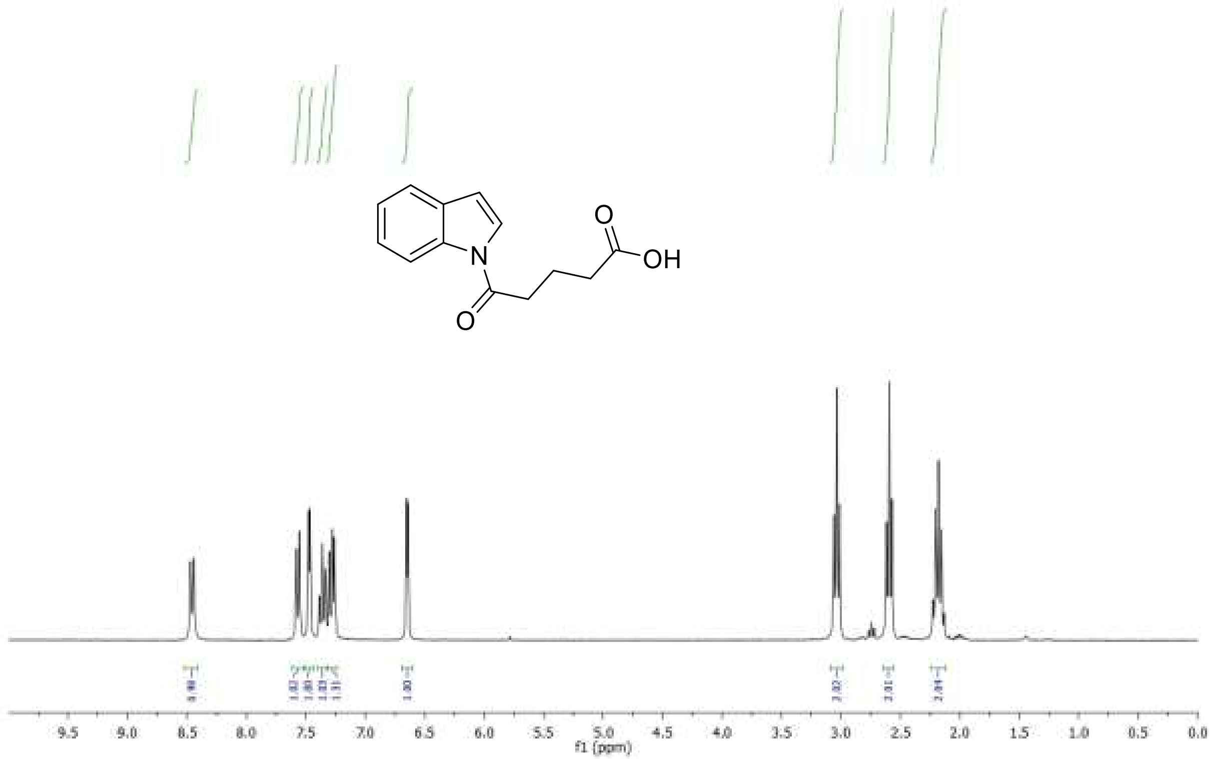
N
O
OH
O
f1 (ppm)

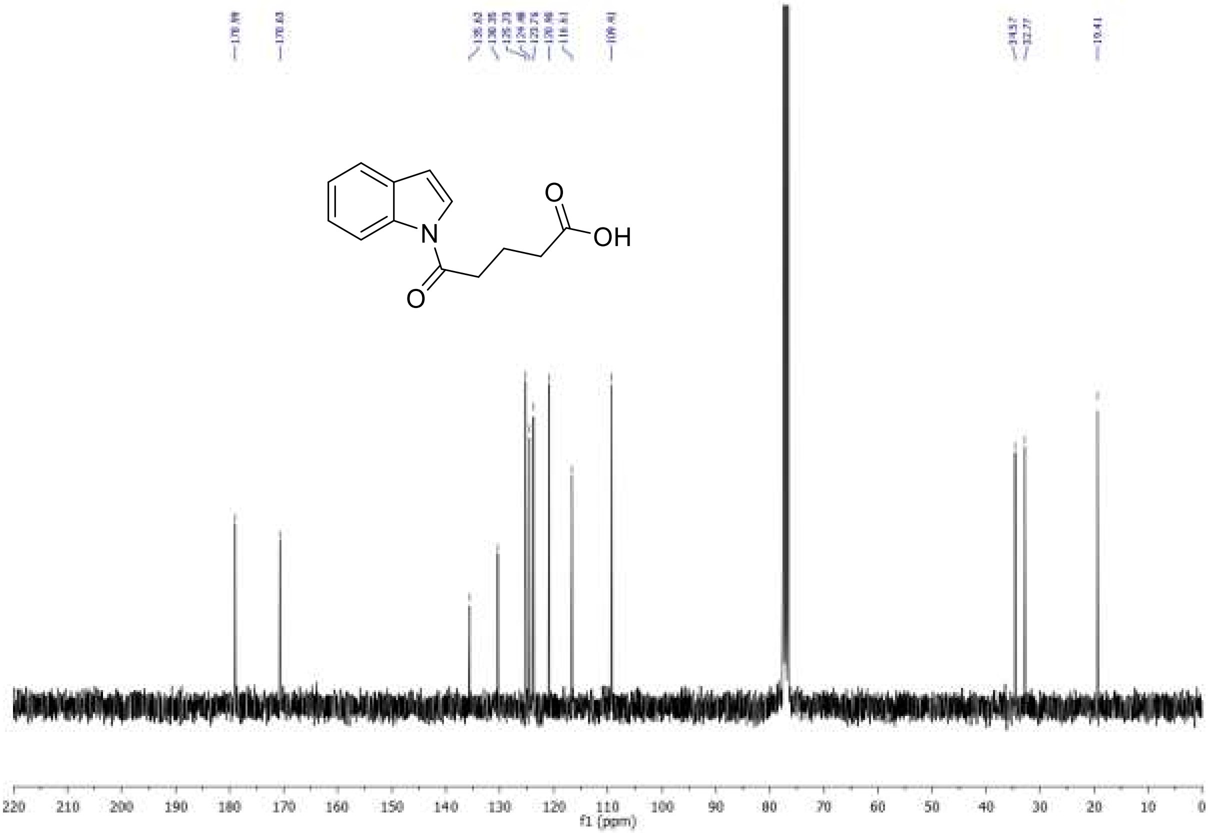
N
O
OH
O
f1 (ppm)

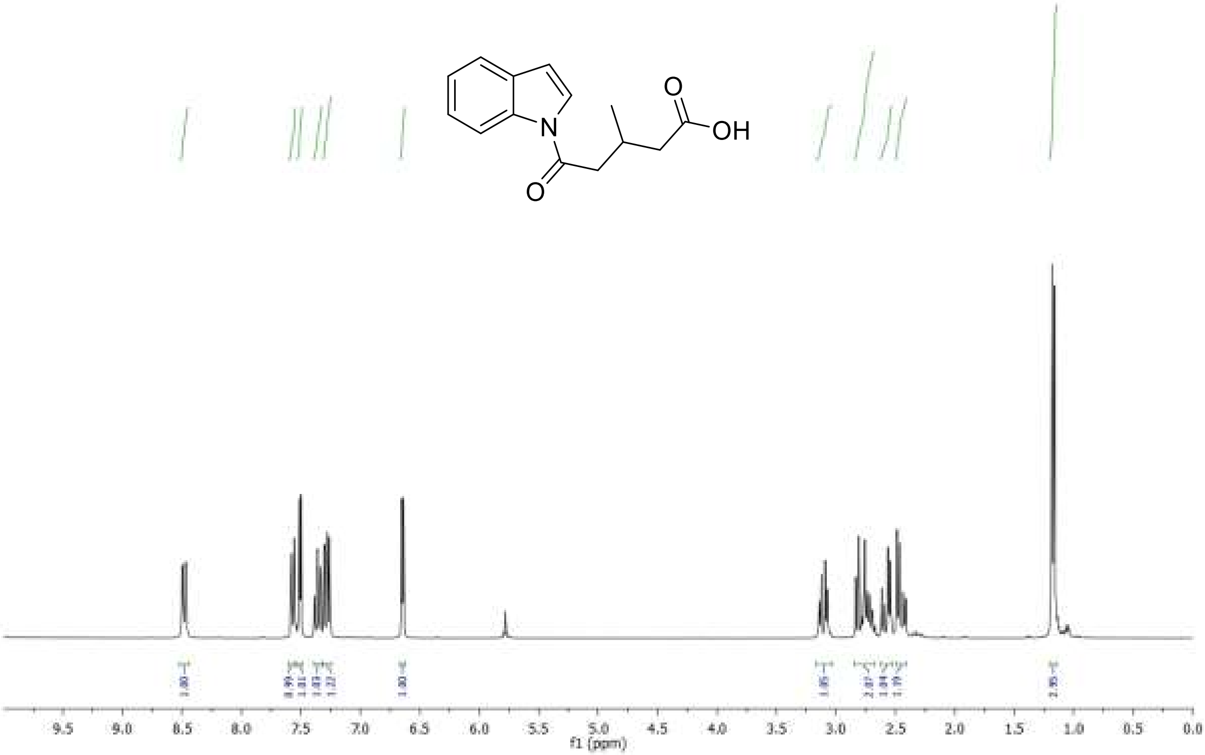
N
O
OH
f1 (ppm)

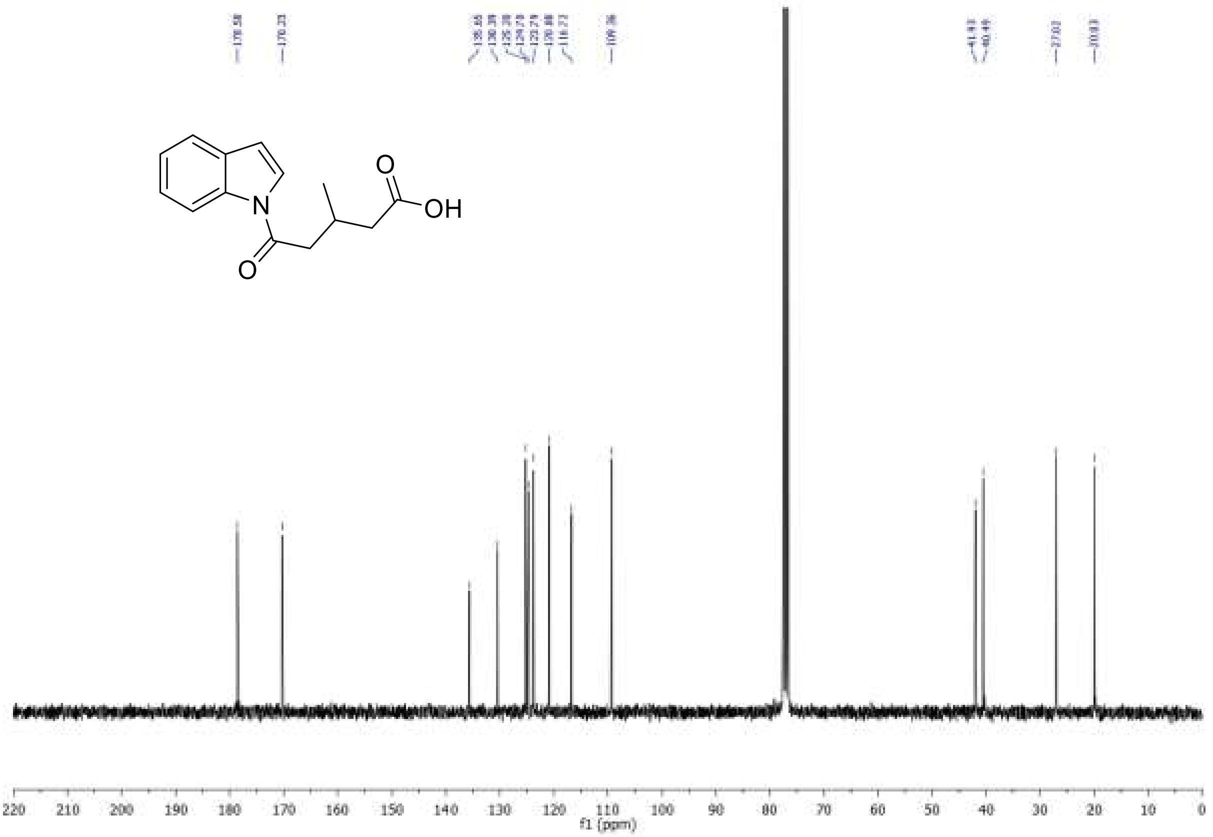
N
O
OH
f1 (ppm)

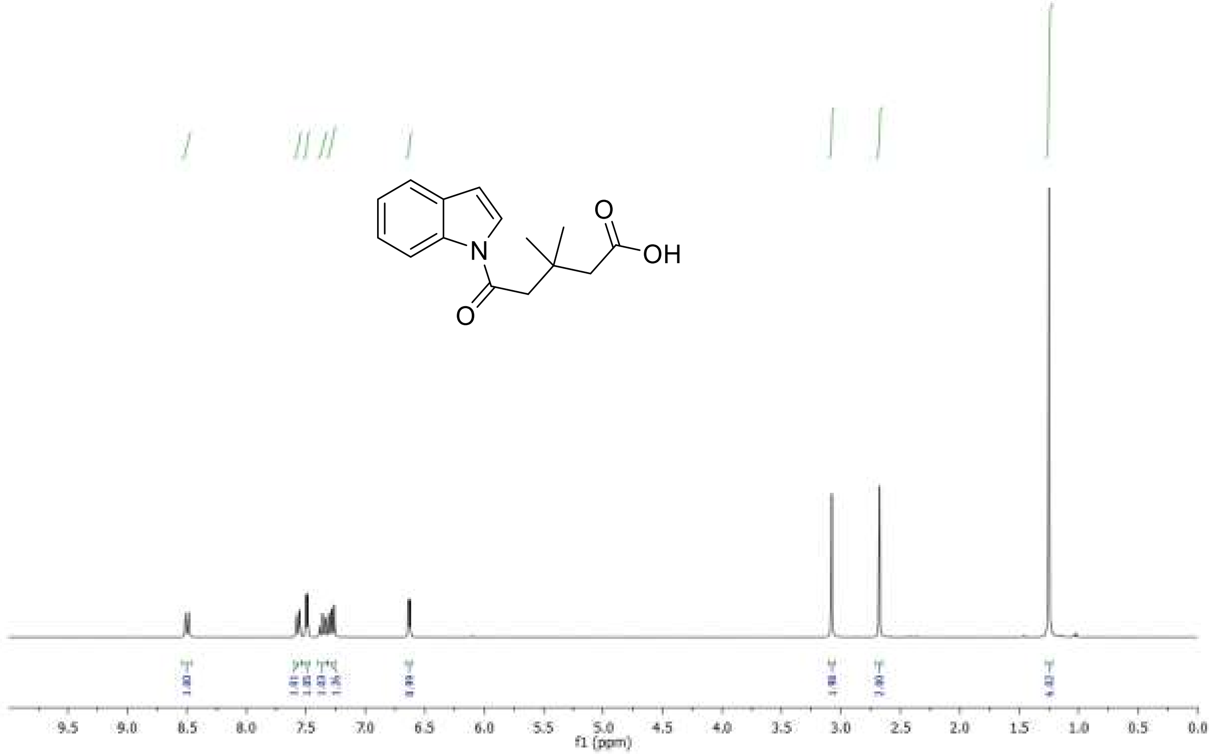
N
O
OH
f1 (ppm)

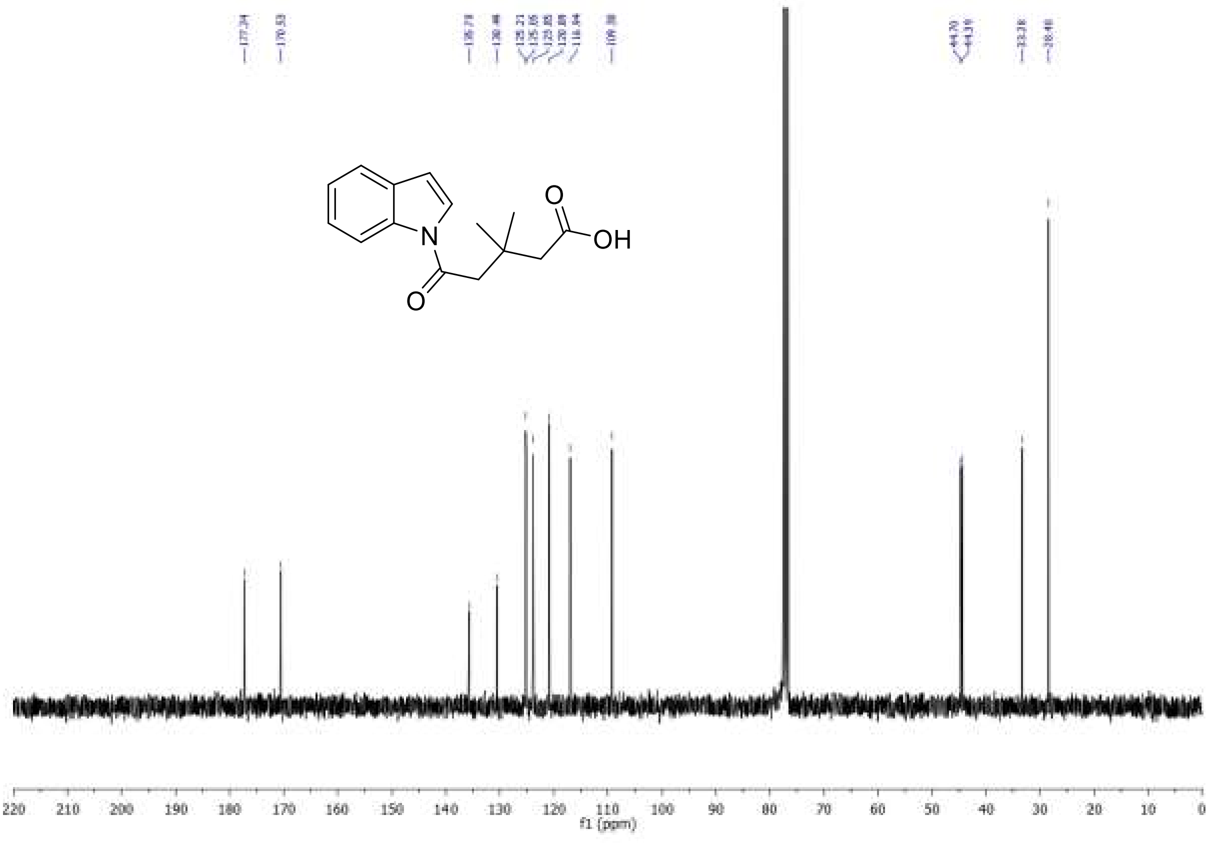
N
O
OH
f1 (ppm)

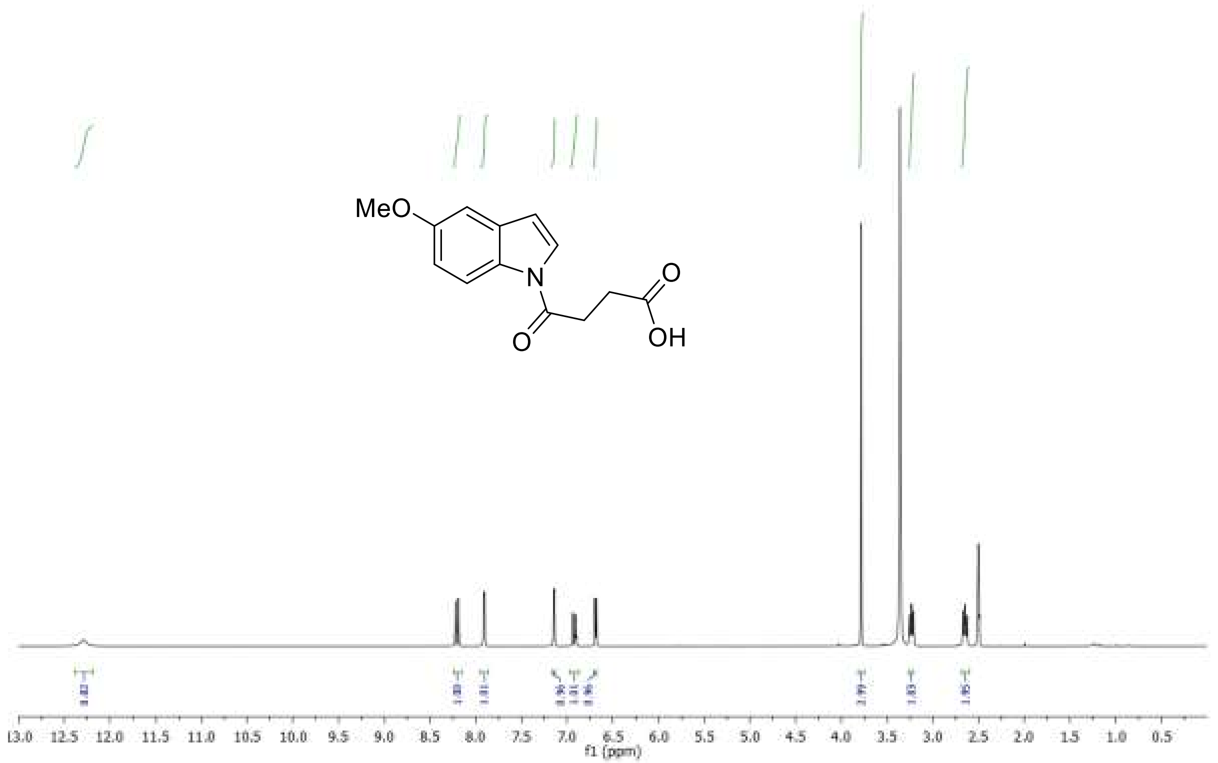

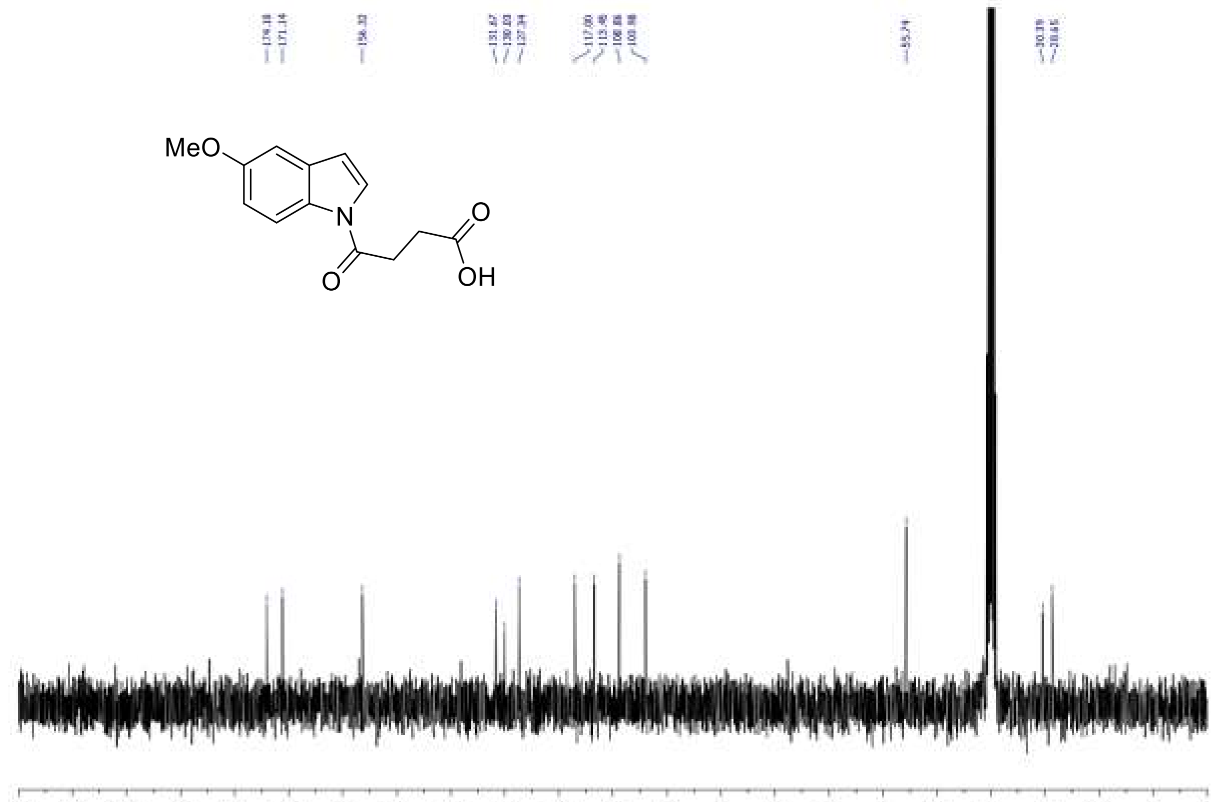

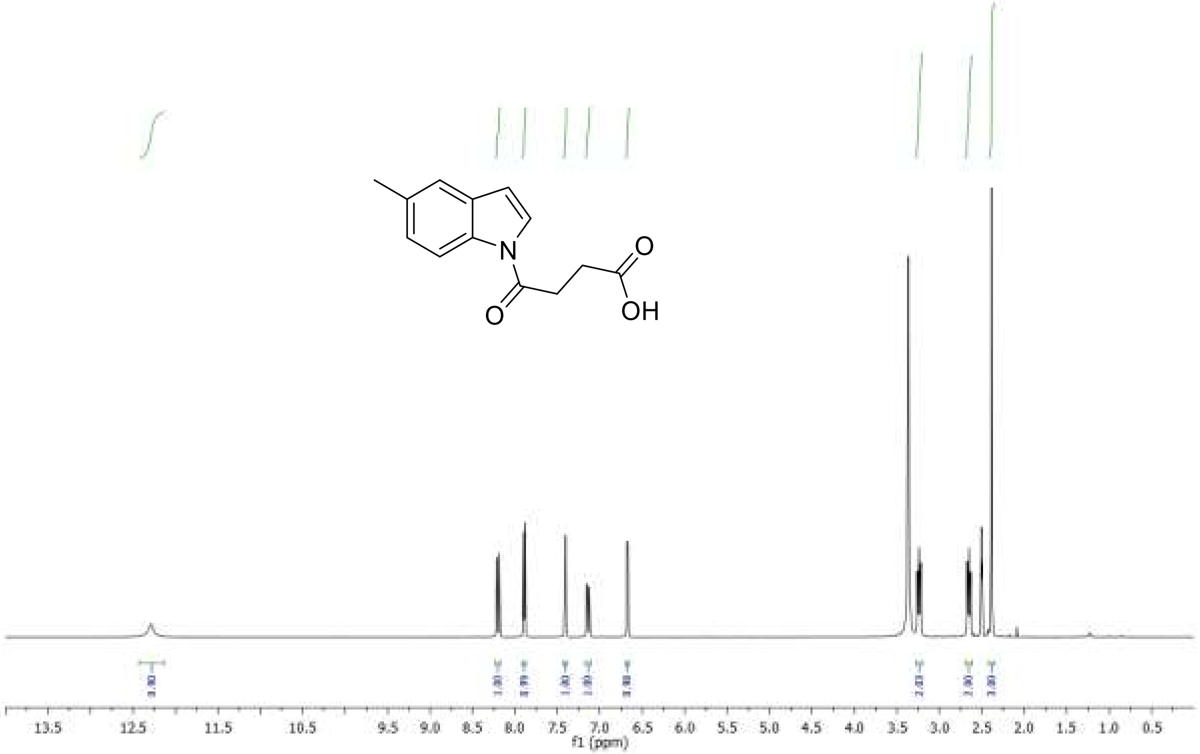
N
O
OH
13.5 12.5 11.5 10.5 9.5 9.0 8.5 8.0 7.5 7.0 6.5 6.0 5.5 5.0 4.5 4.0 3.5 3.0 2.5 2.0 1.5 1.0 0.5
f1 (ppm)

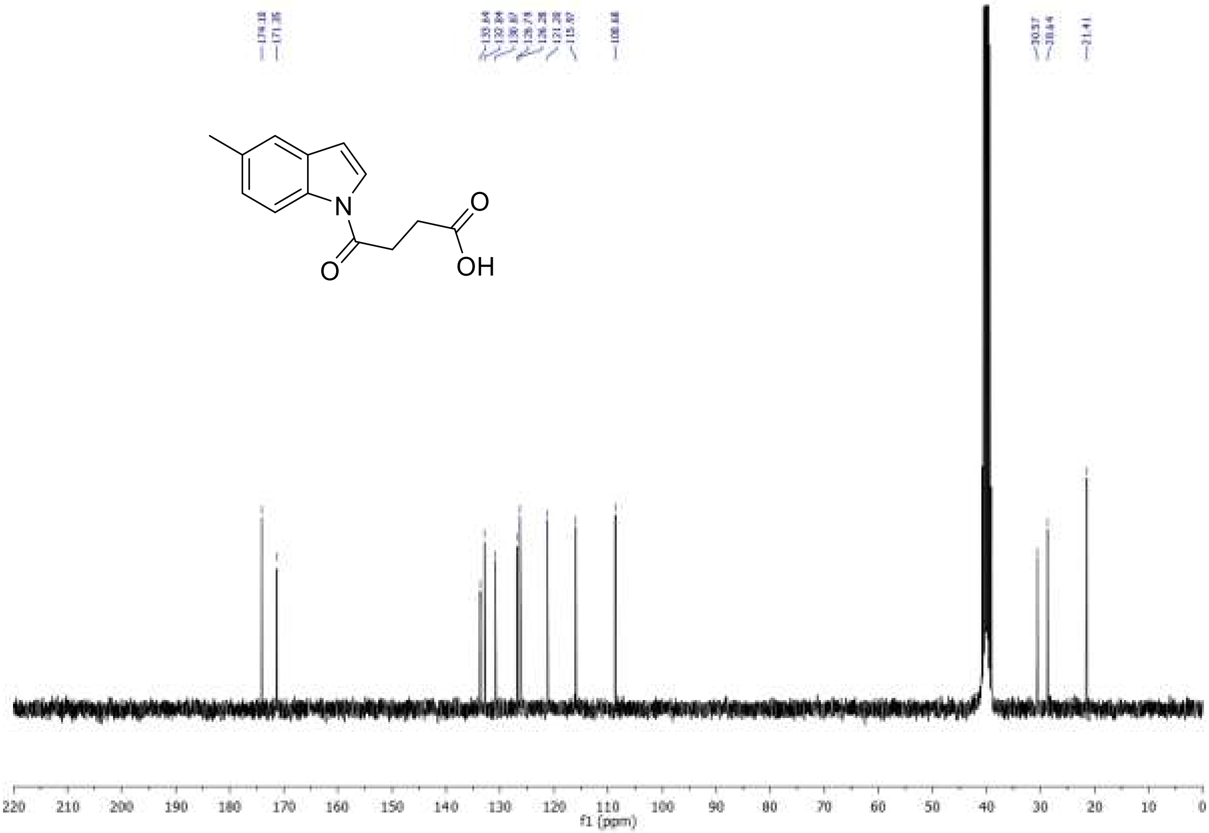
N
O
OH
220 210 200 190 180 170 160 150 140 130 120 110 100 90 80 70 60 50 40 30 20 10 0
f1 (ppm)

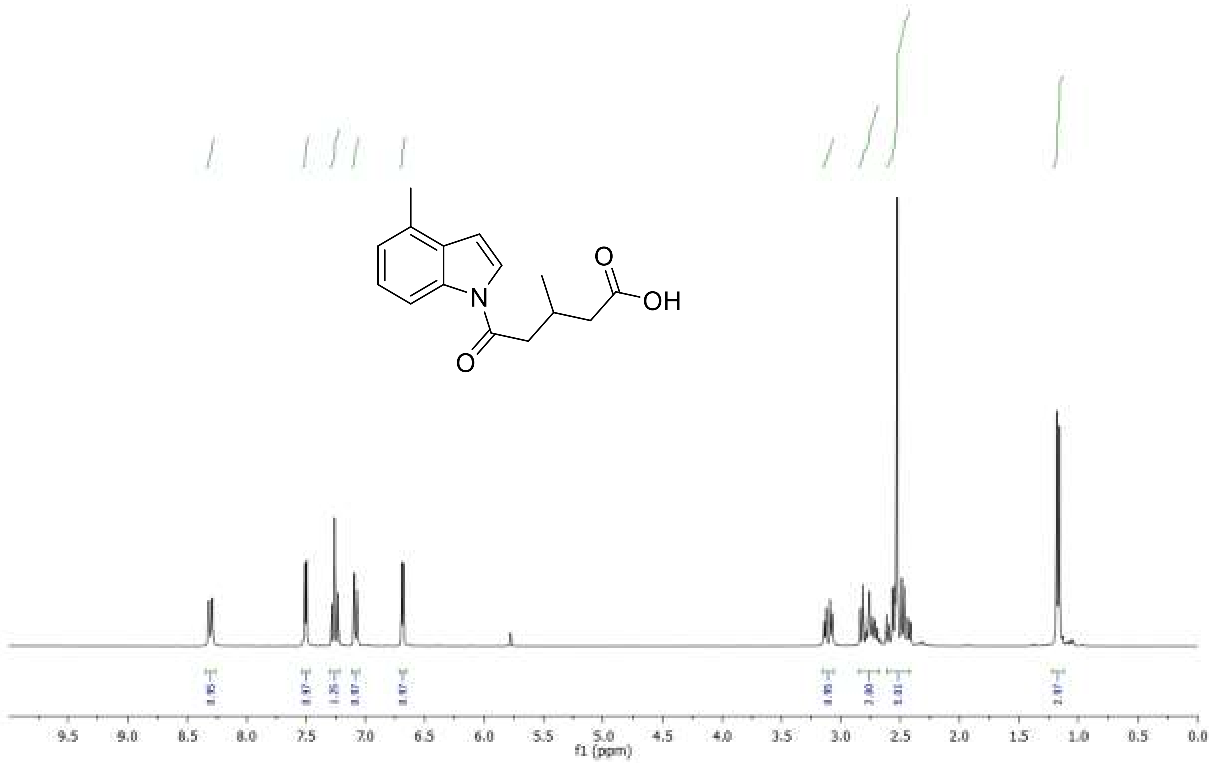

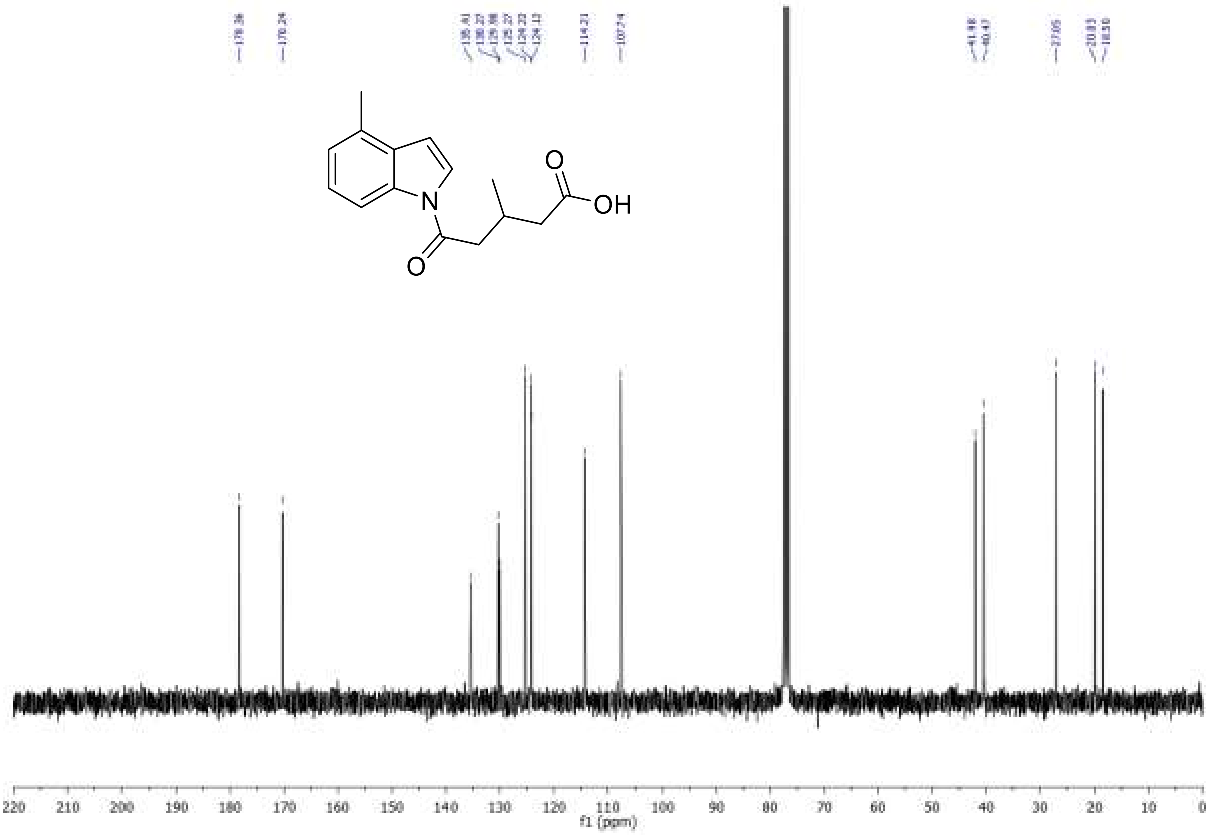

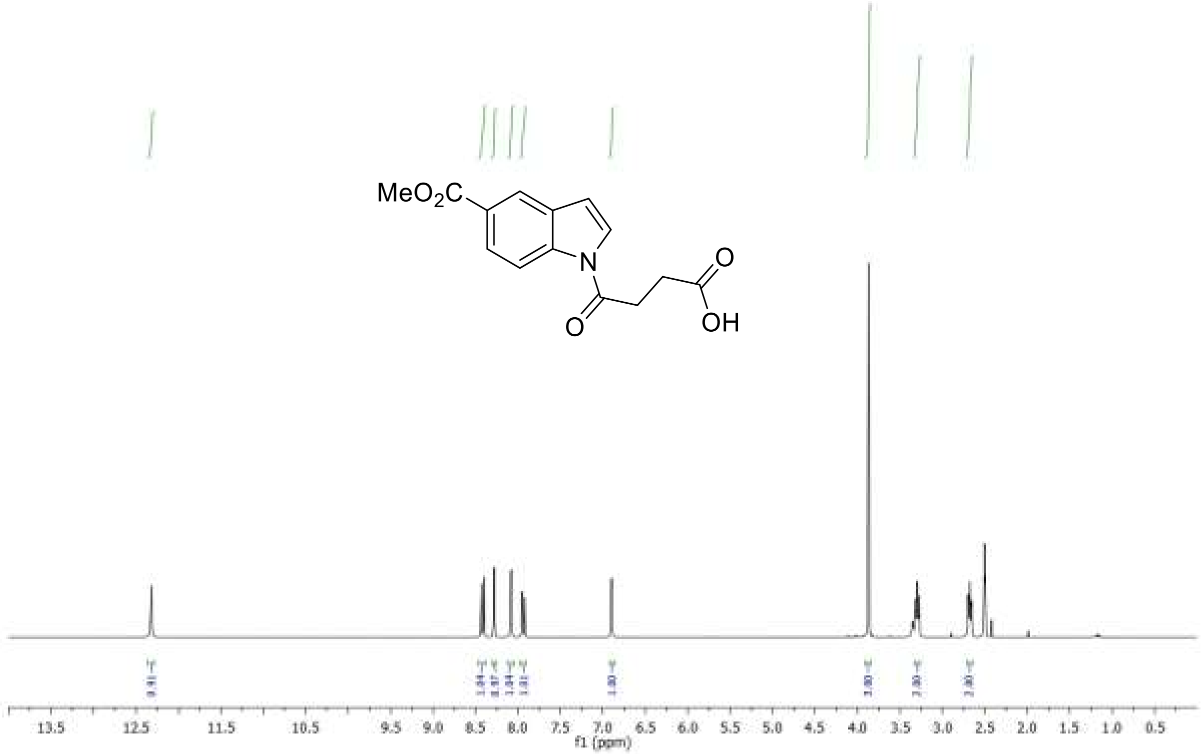
MeO2C
N
O
OH
O
f1 (ppm)

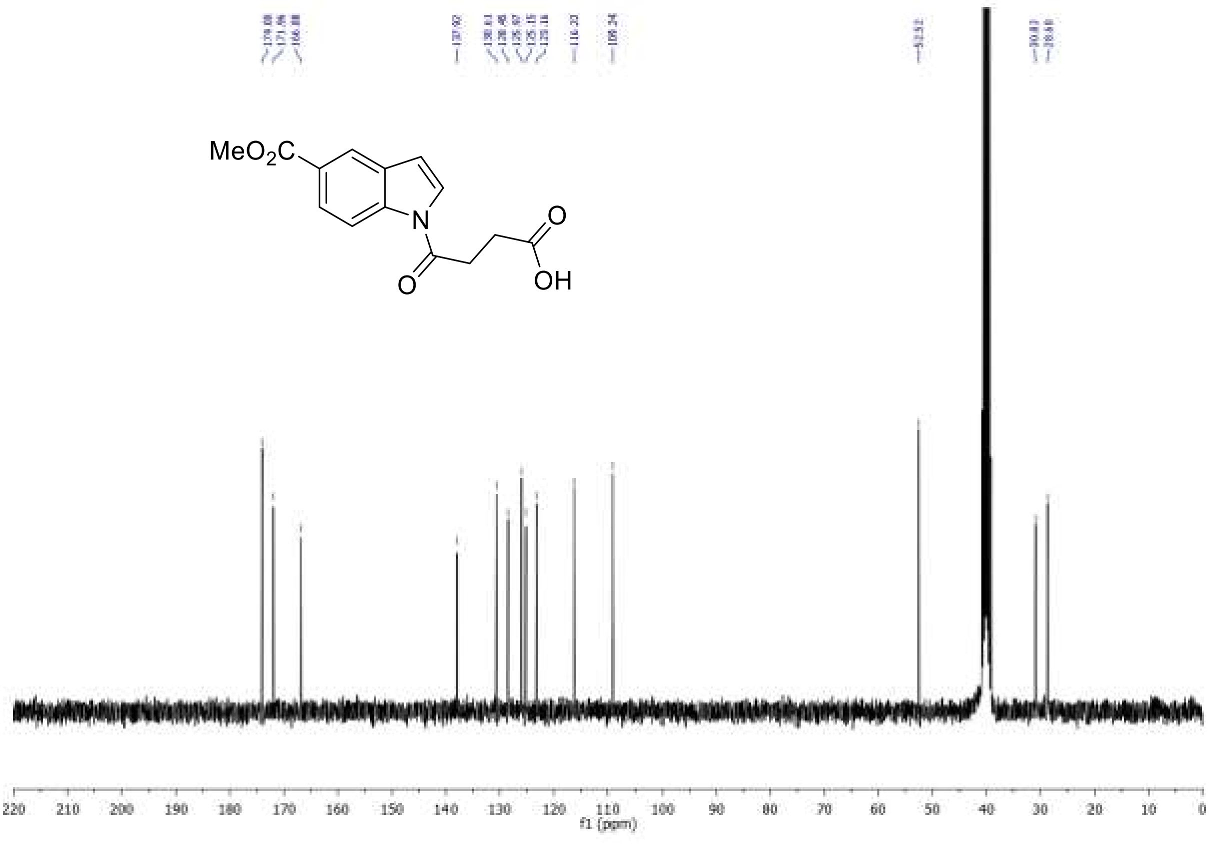
MeO2C
N
O
OH
O
f1 (ppm)

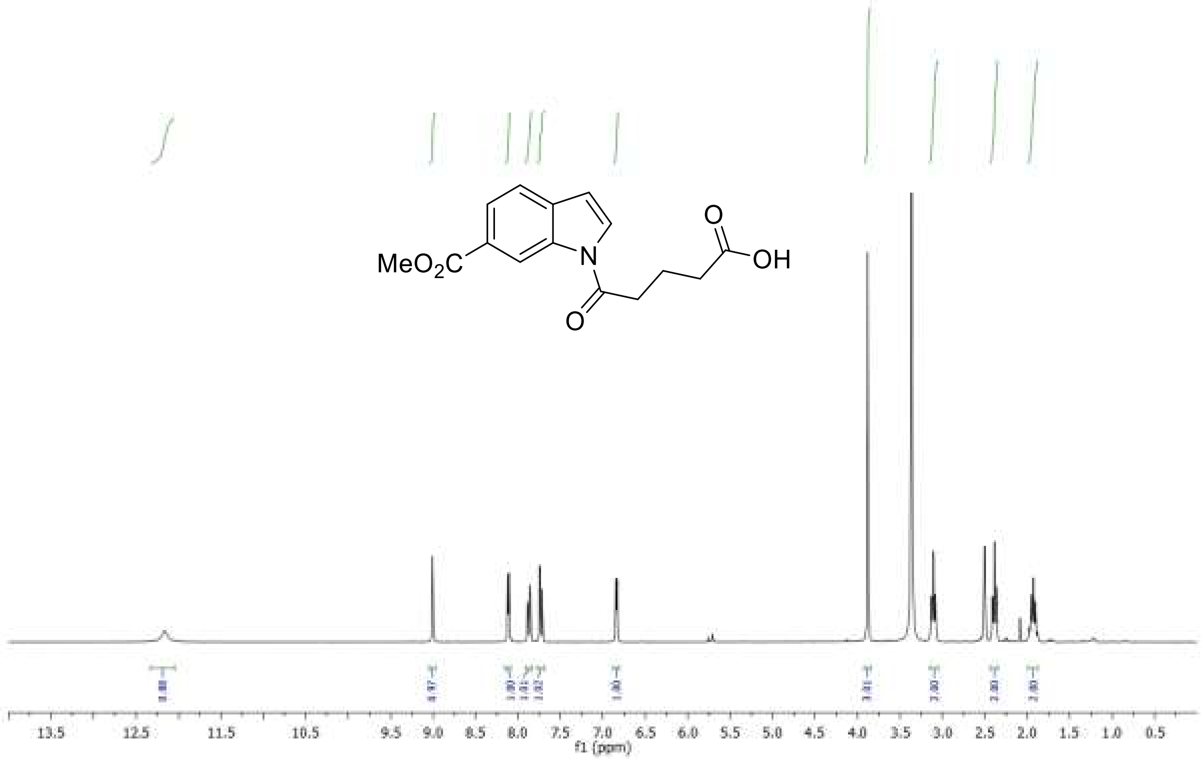

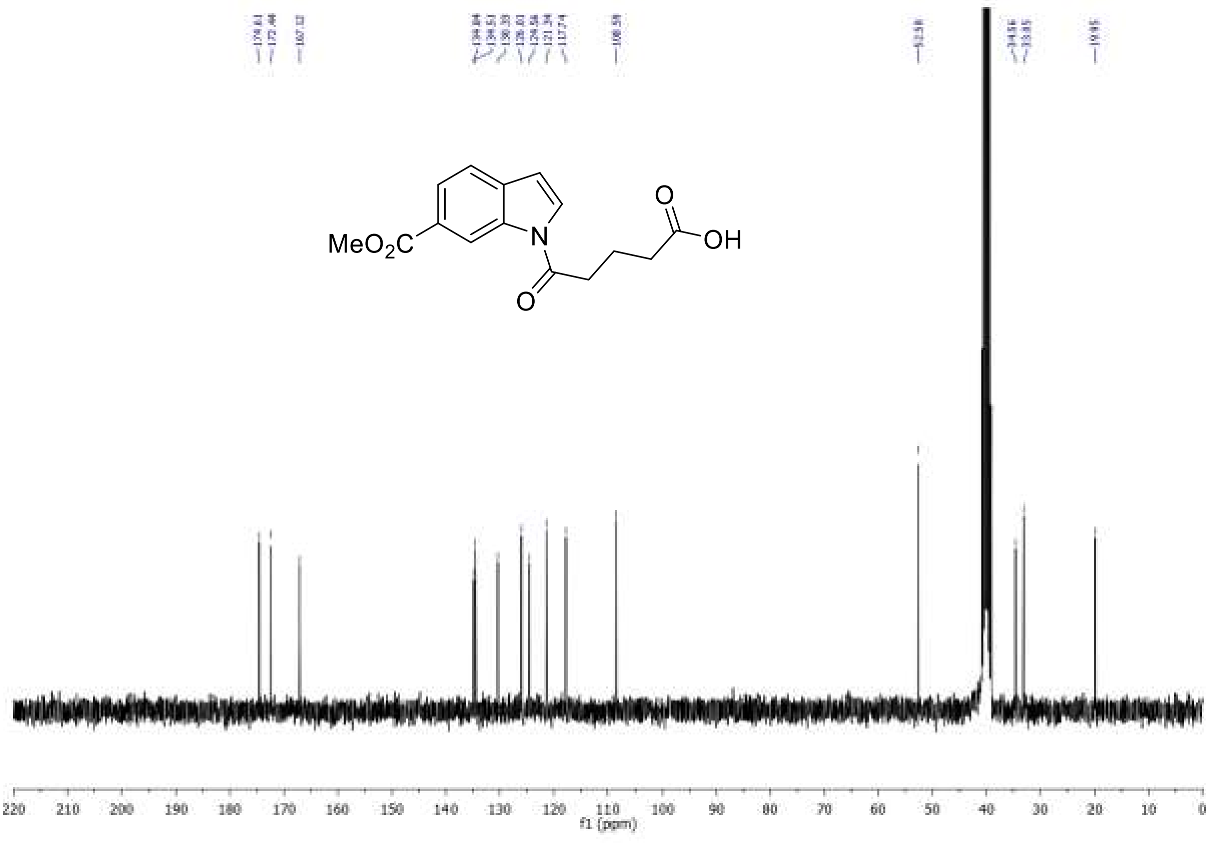

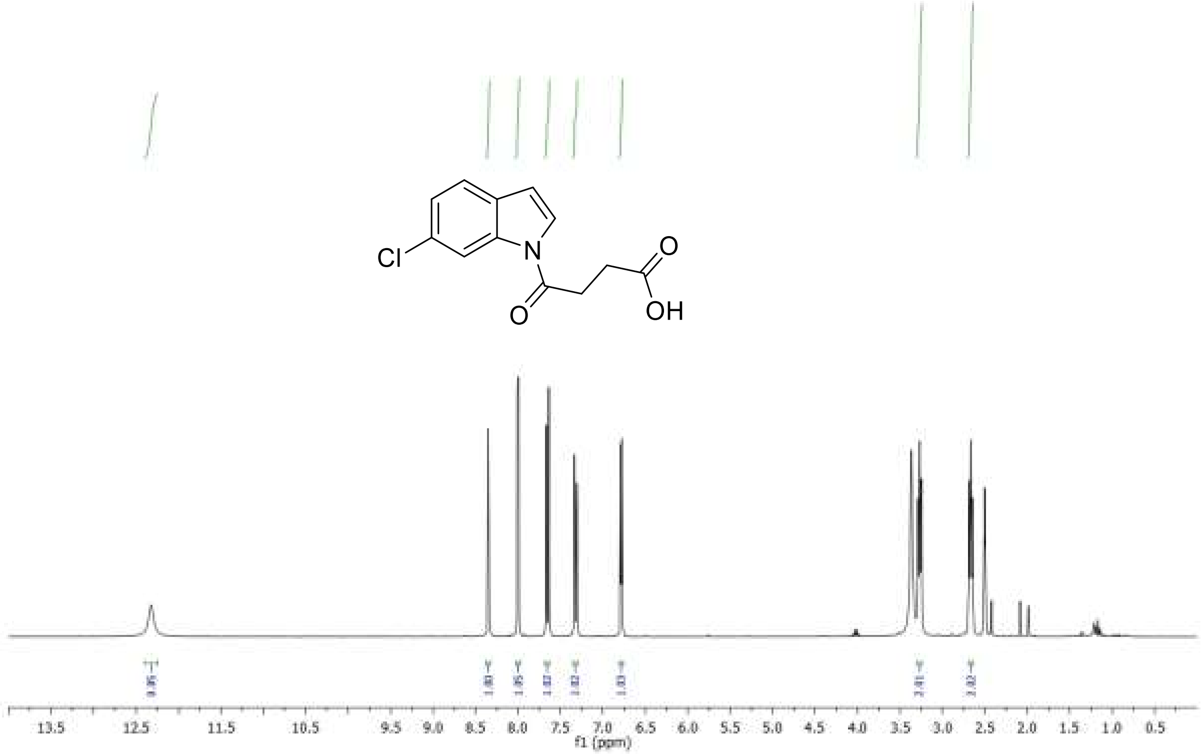

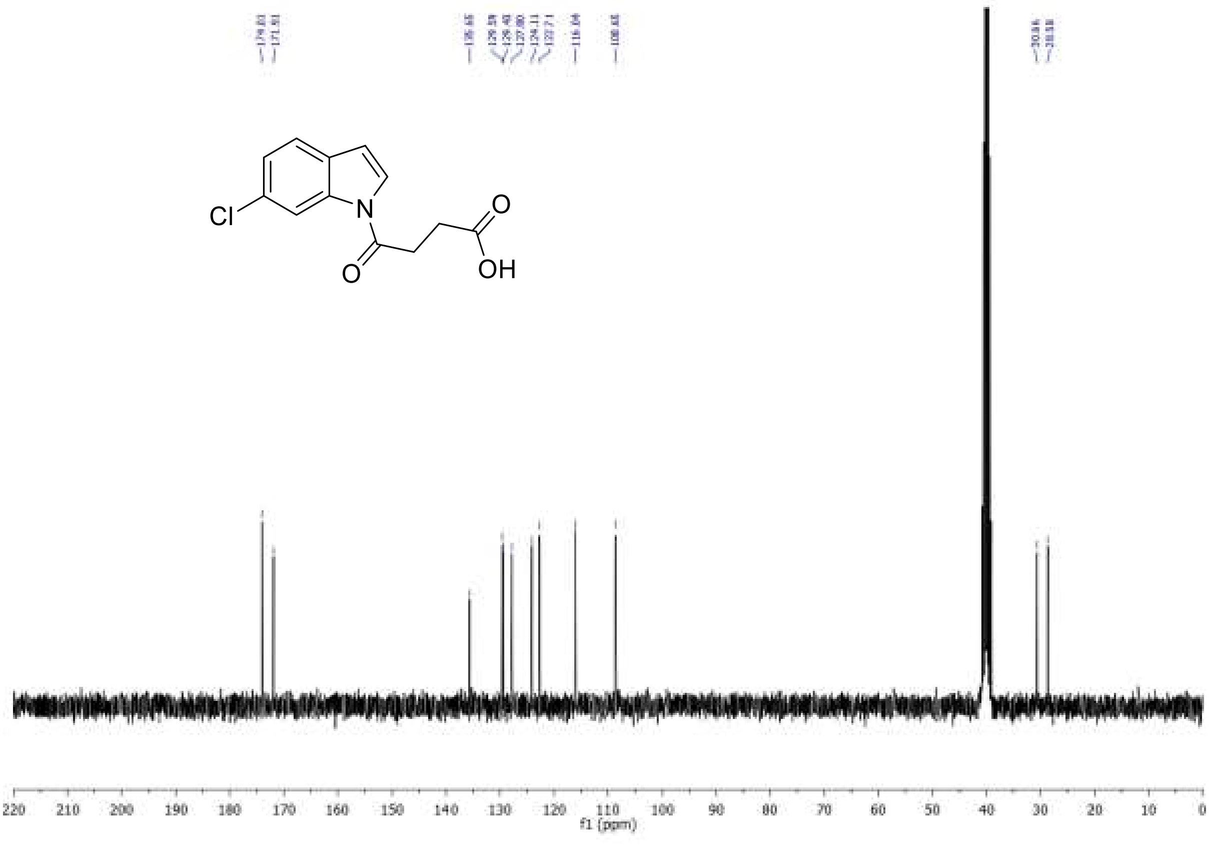

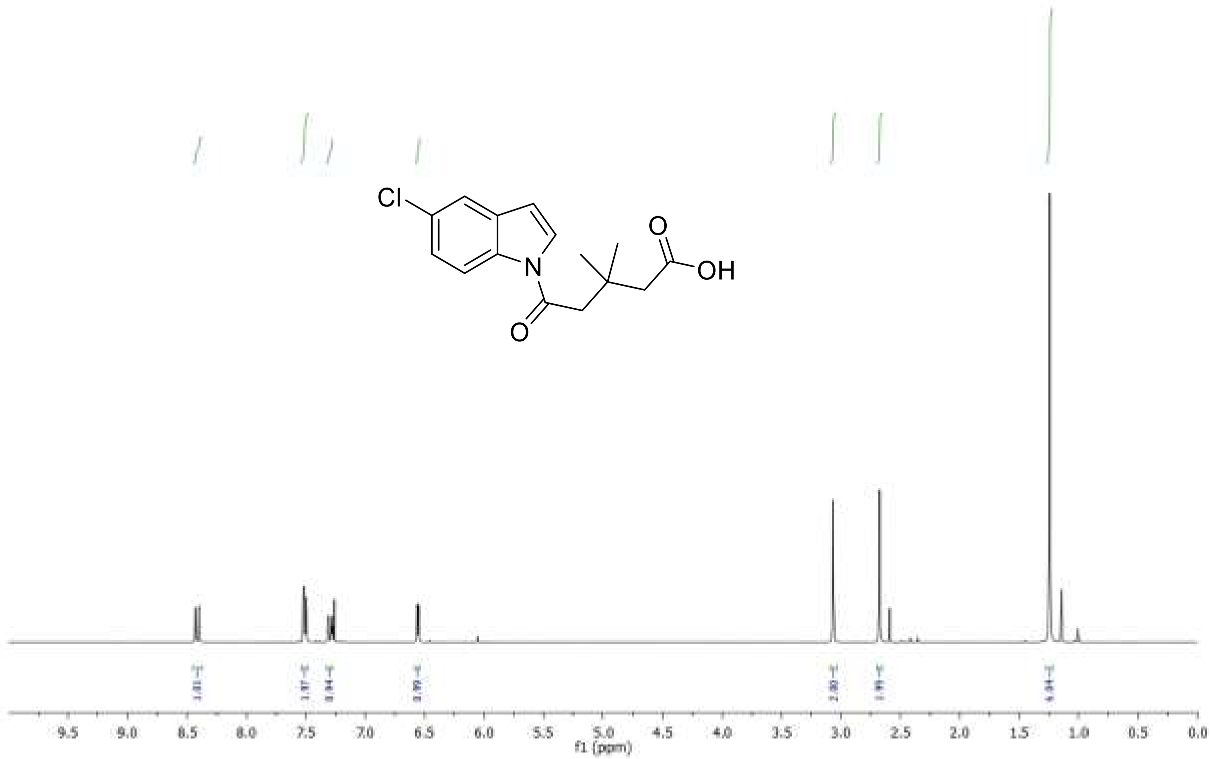
Cl
N
O
OH
f1 (ppm)

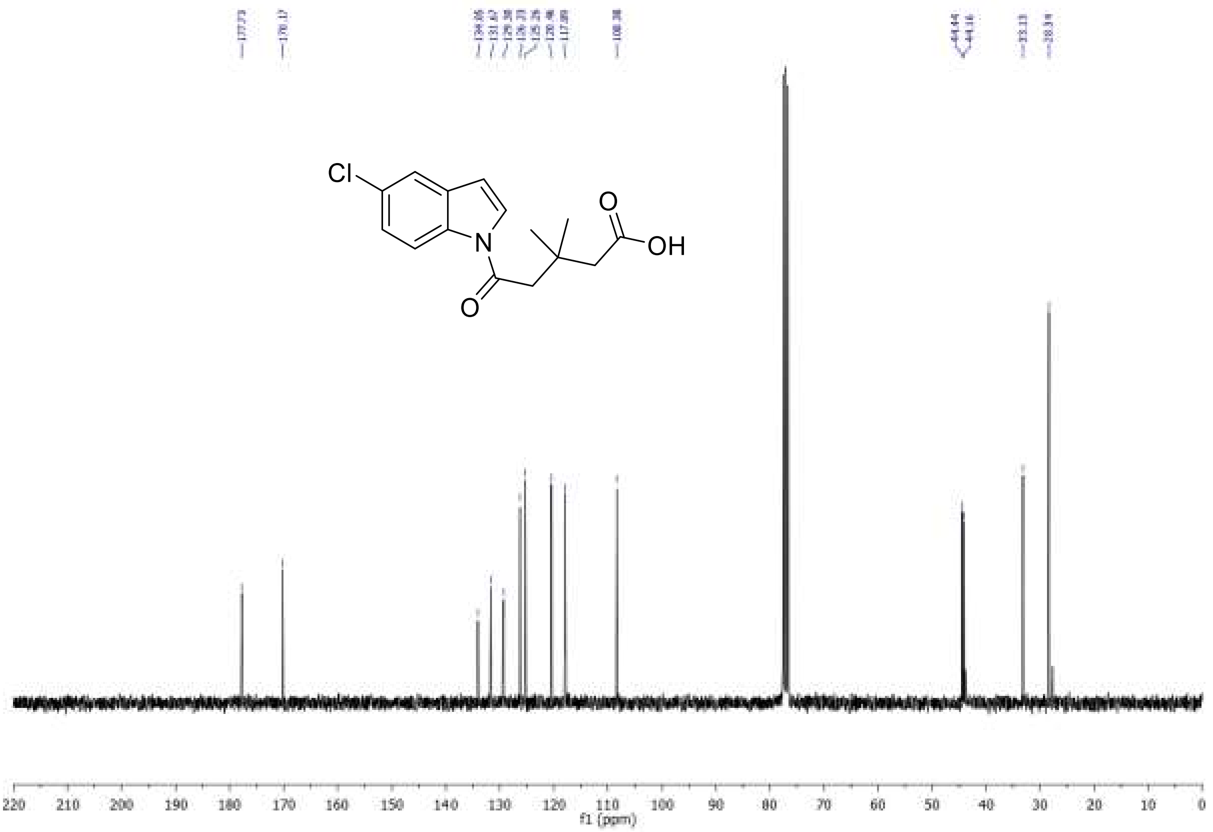
Cl
N
O
OH
f1 (ppm)

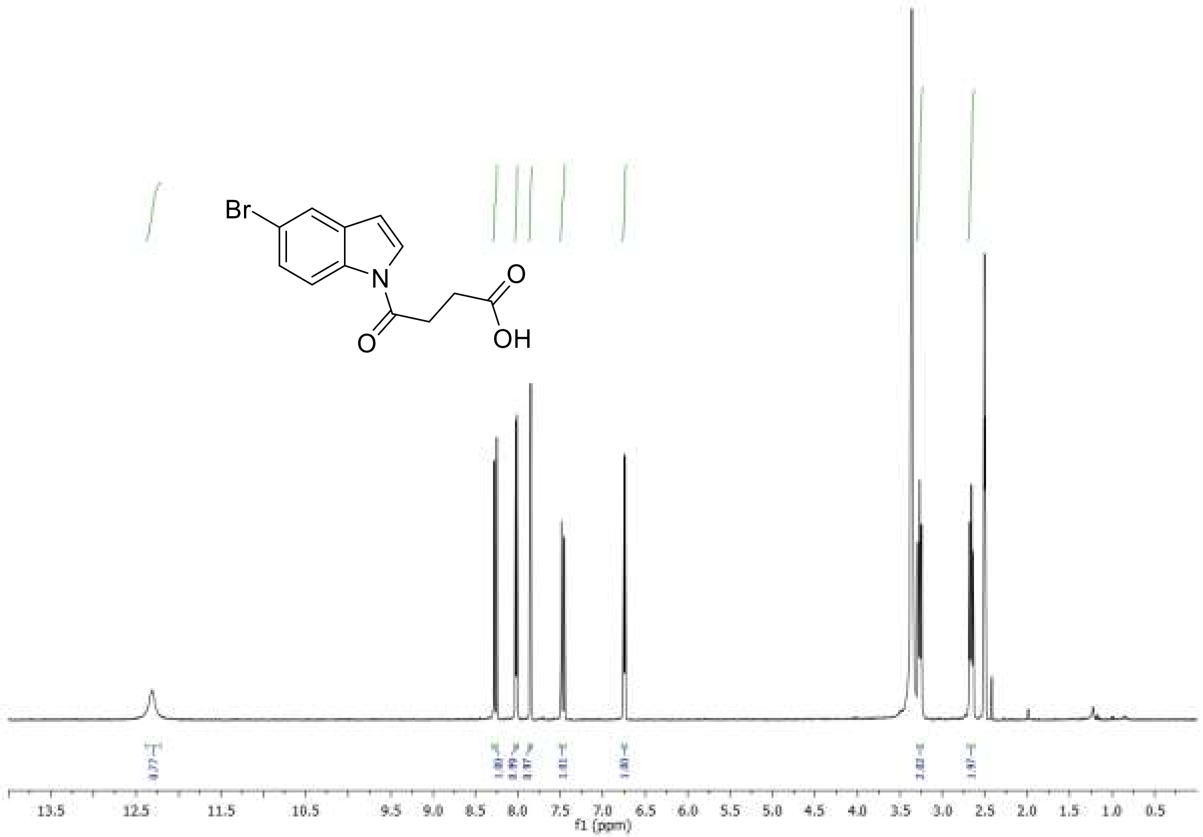

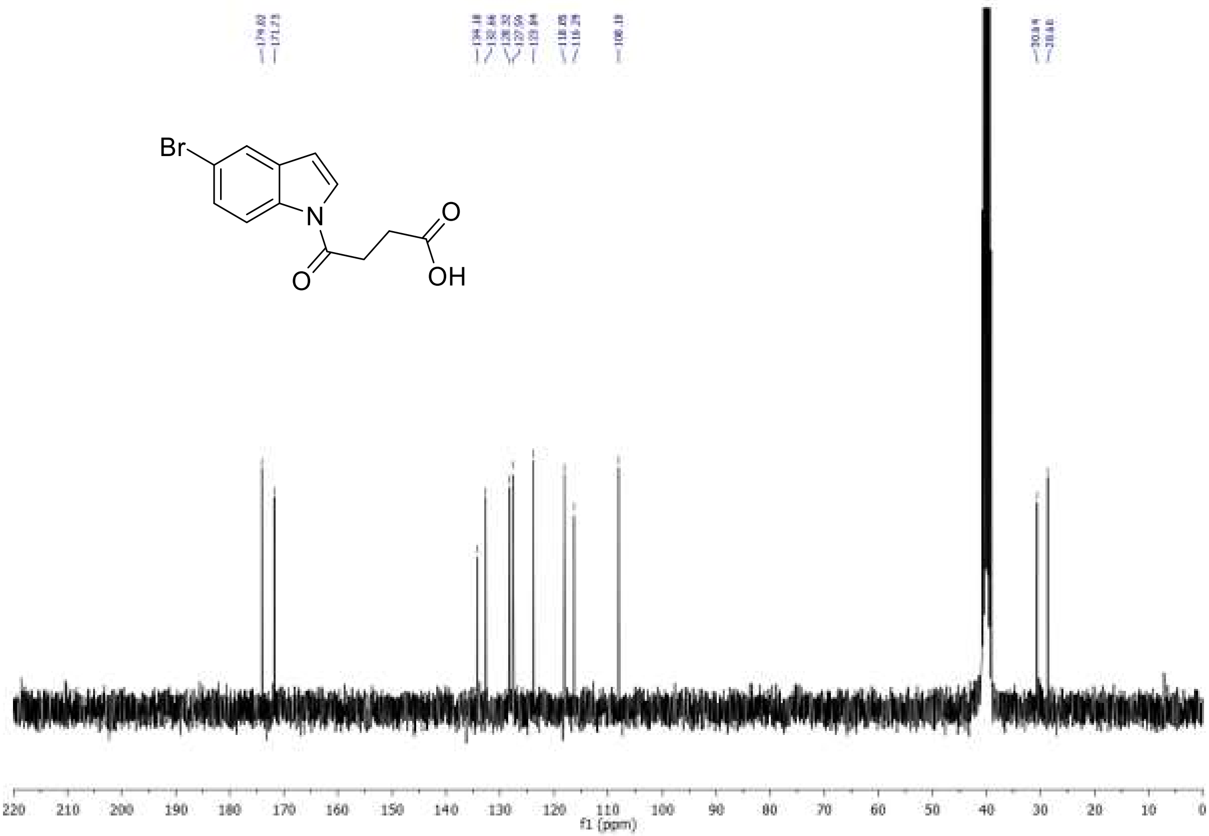

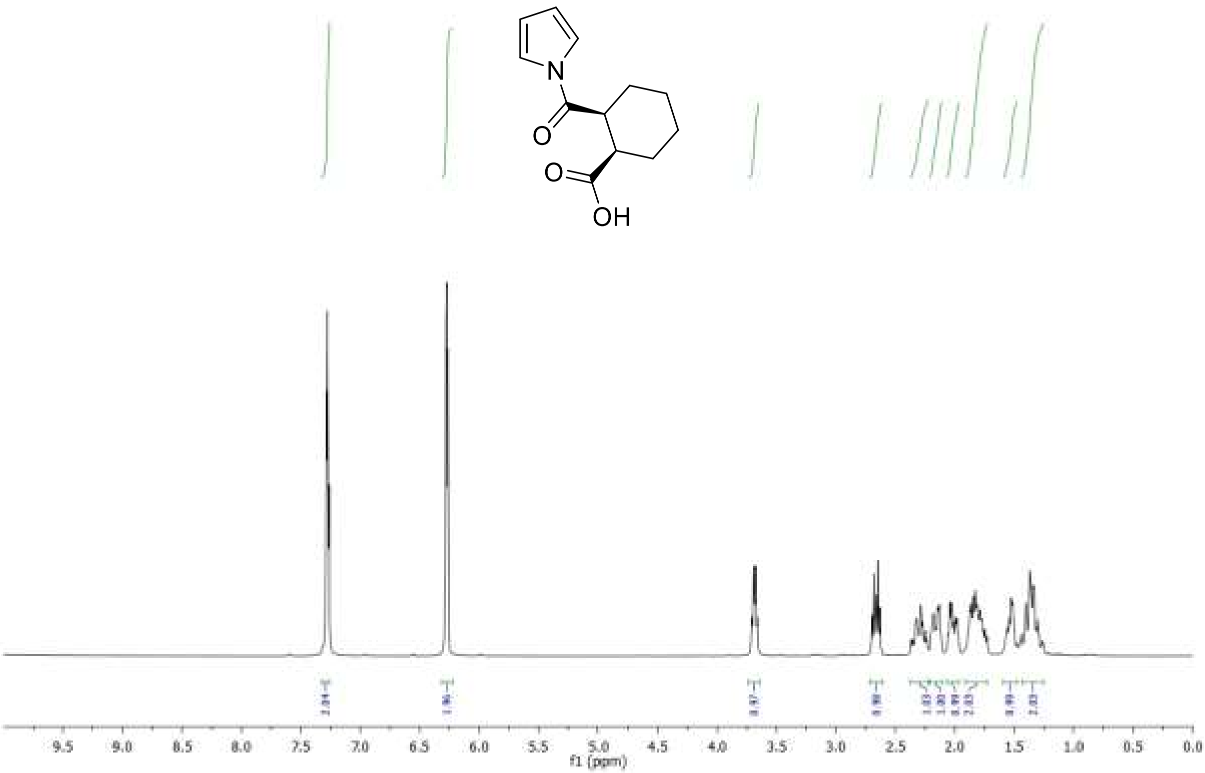

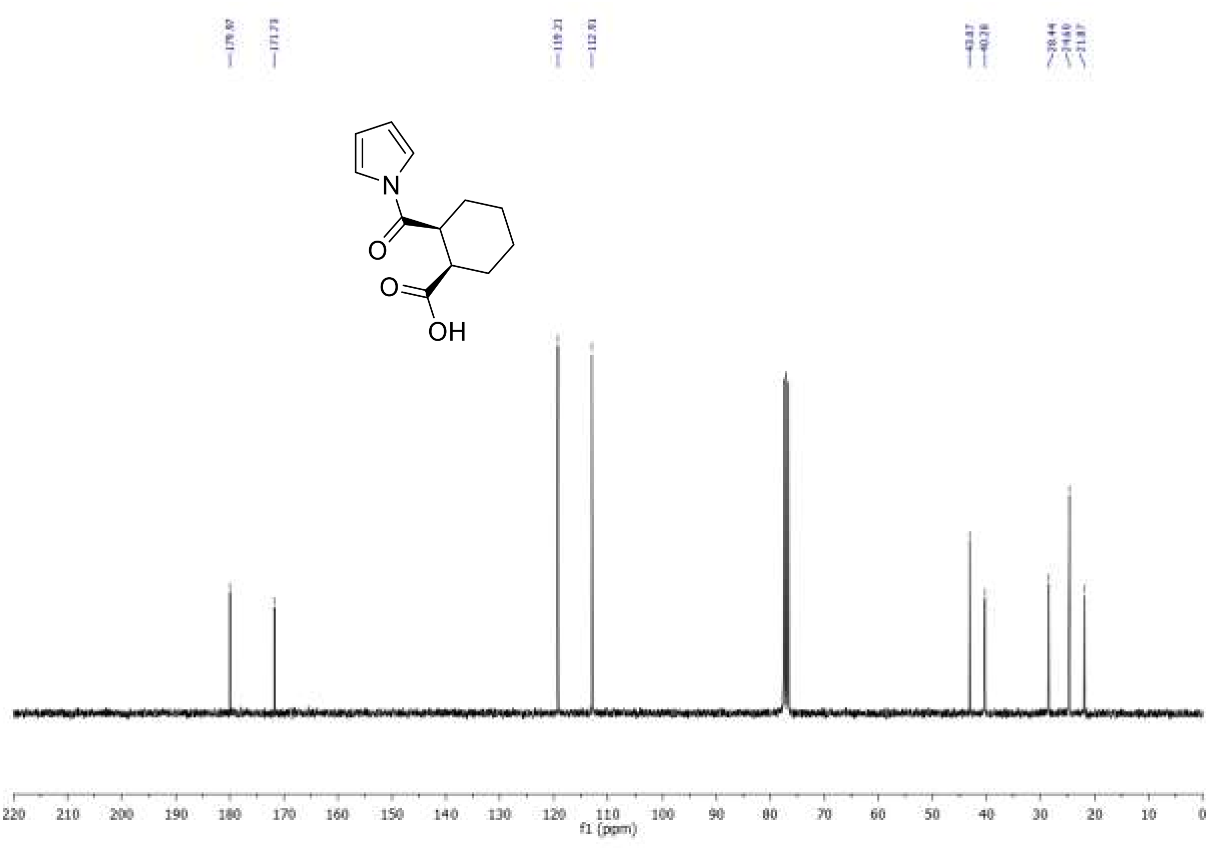

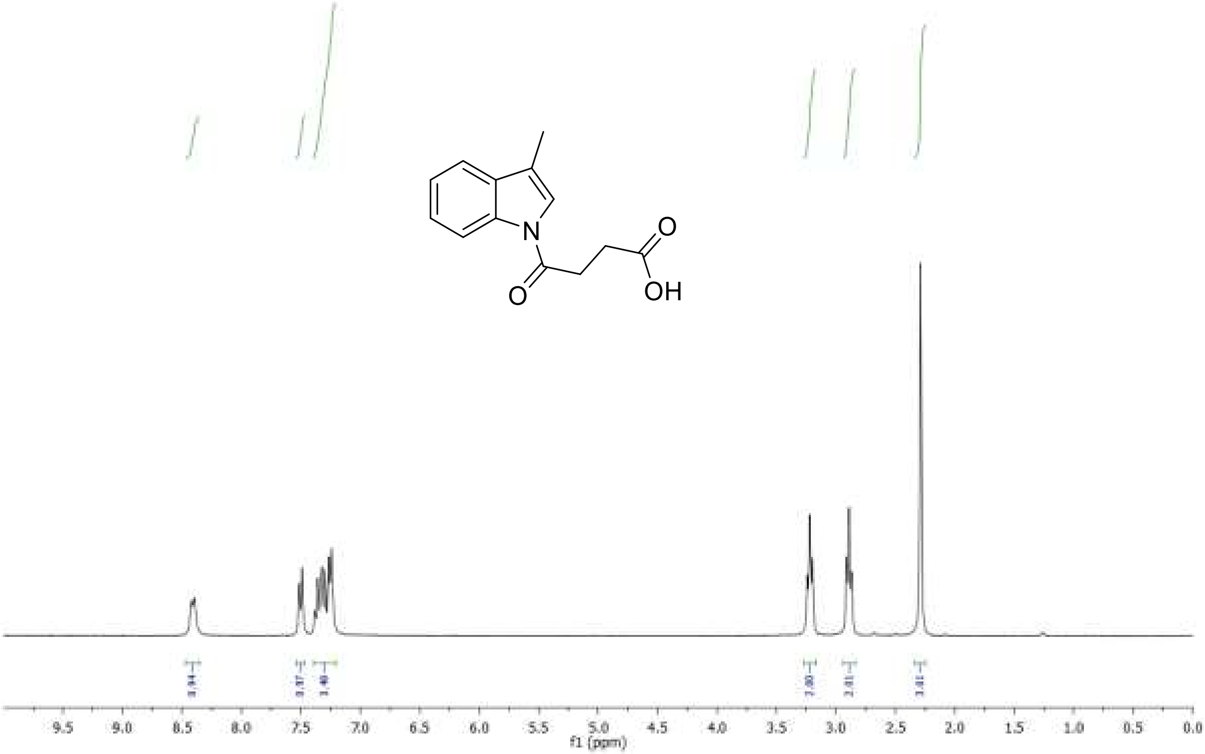
N
O
OH
9.5 9.0 8.5 8.0 7.5 7.0 6.5 6.0 5.5 5.0 4.5 4.0 3.5 3.0 2.5 2.0 1.5 1.0 0.5 0.0
f1 (ppm)

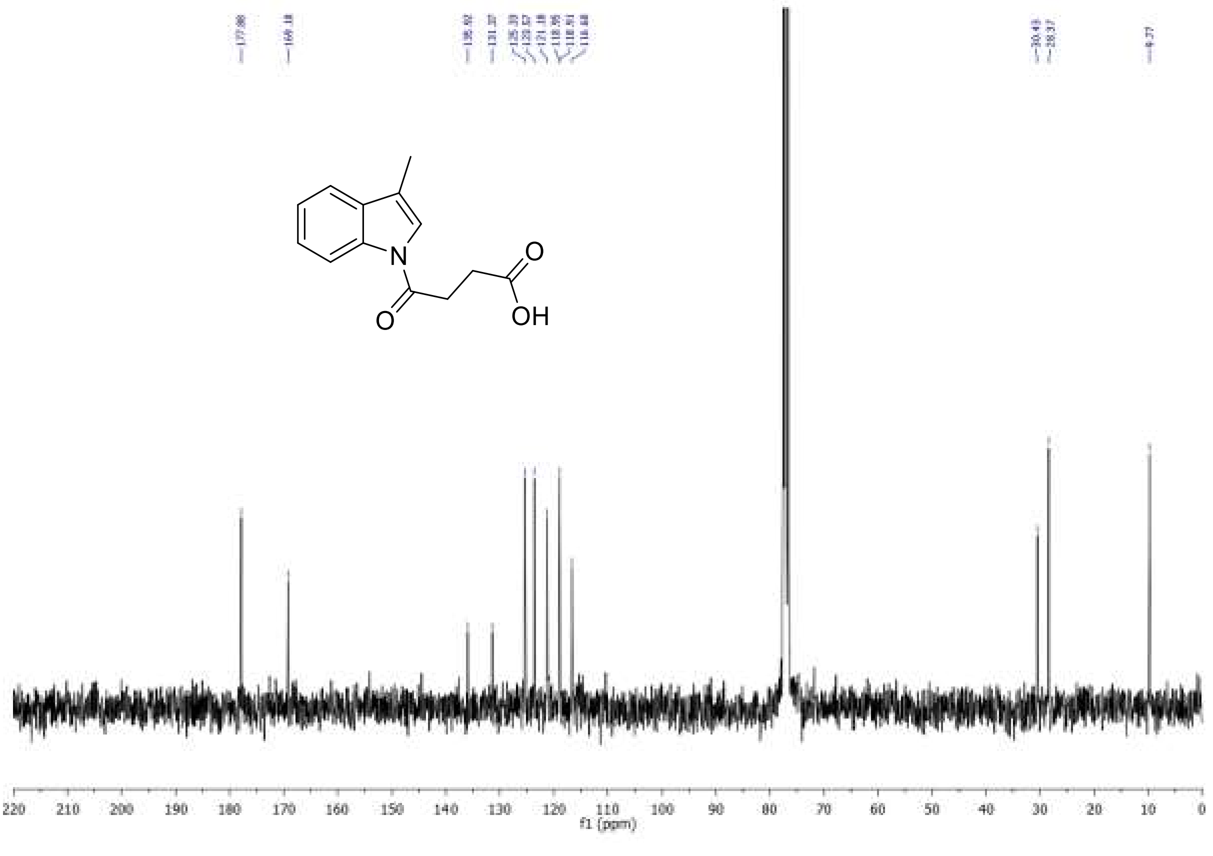
N
O
OH
220 210 200 190 180 170 160 150 140 130 120 110 100 90 80 70 60 50 40 30 20 10 0
f1 (ppm)

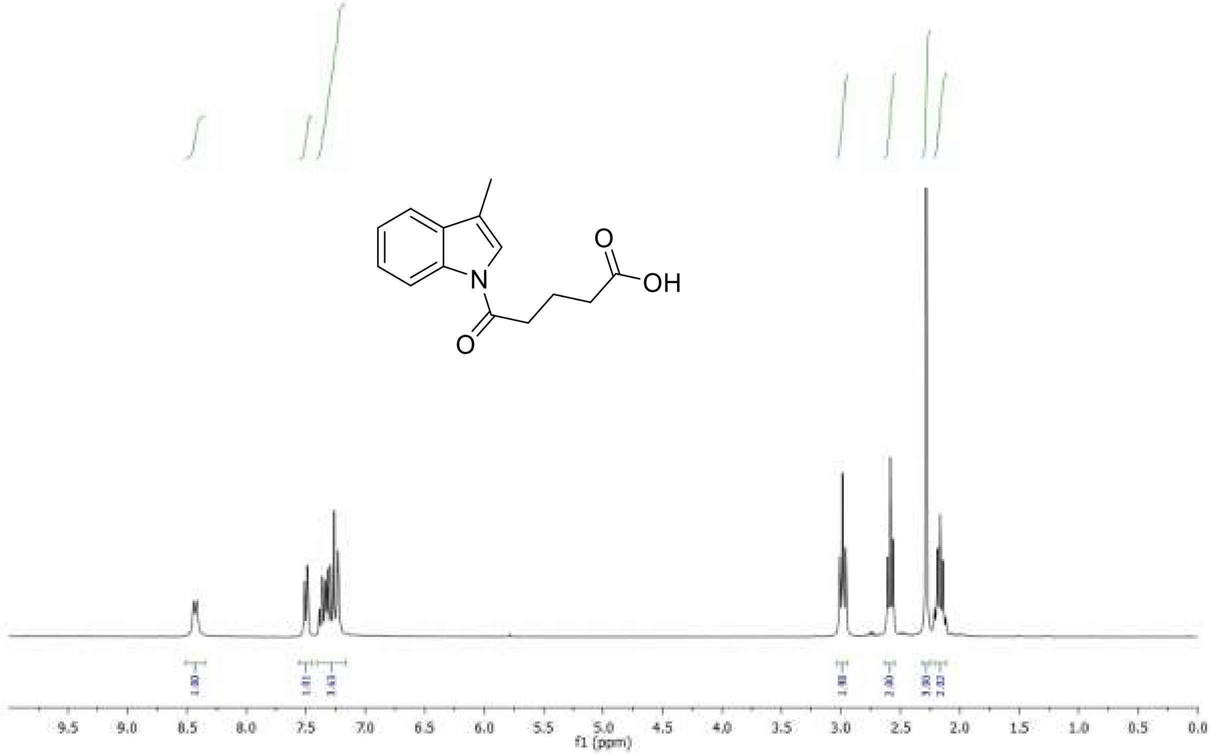

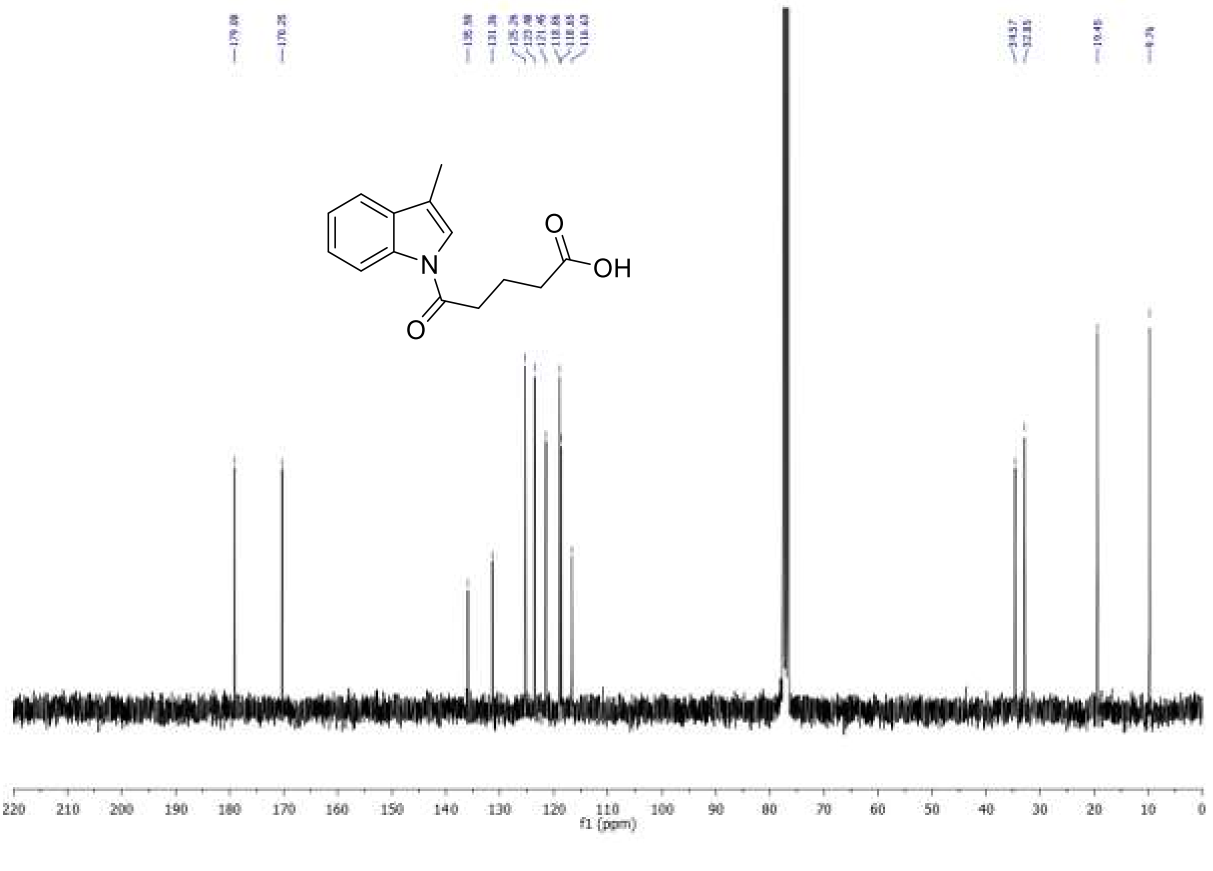

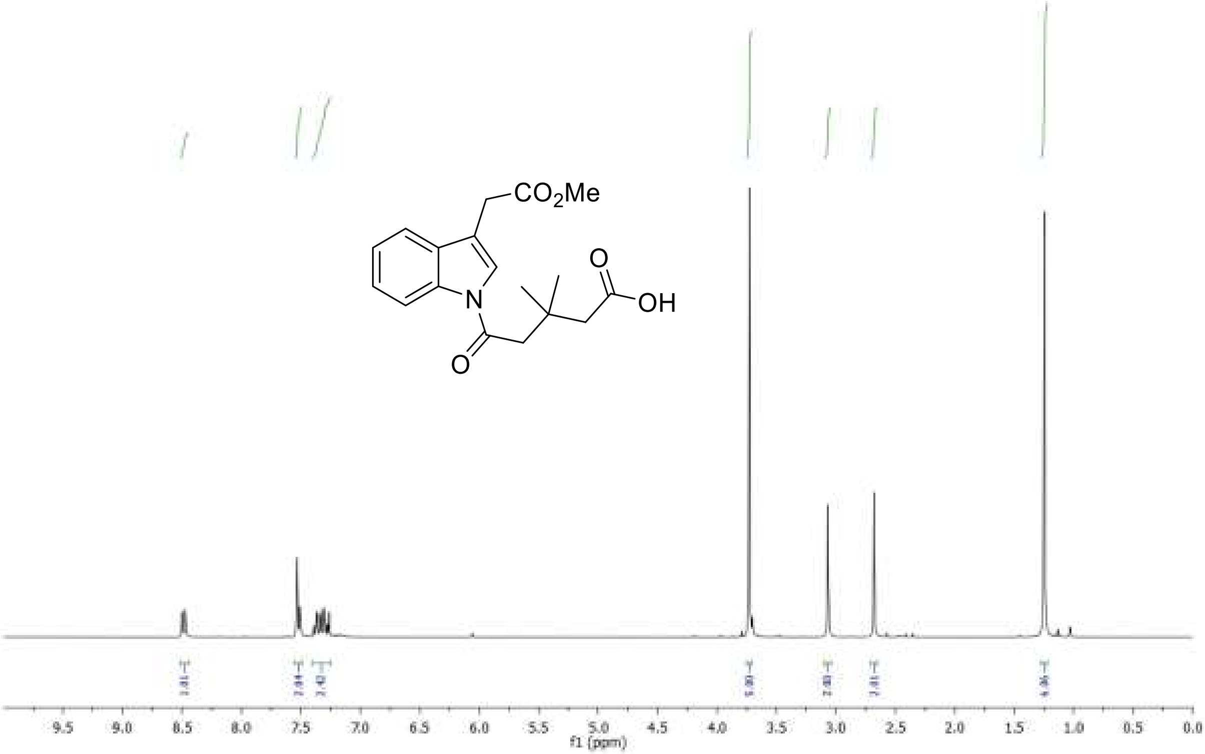
CO2Me
N
O
OH
O
f1 (ppm)

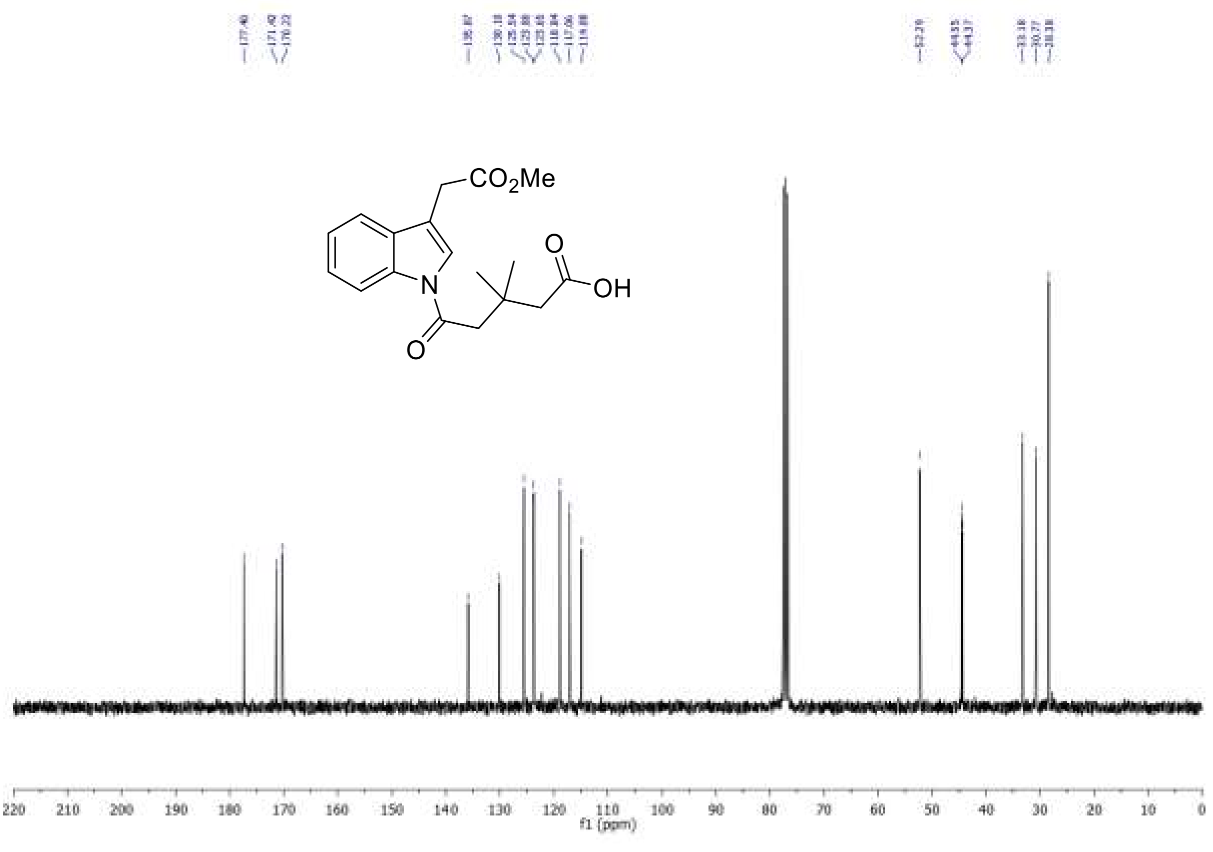
CO2Me
N
O
OH
O
f1 (ppm)

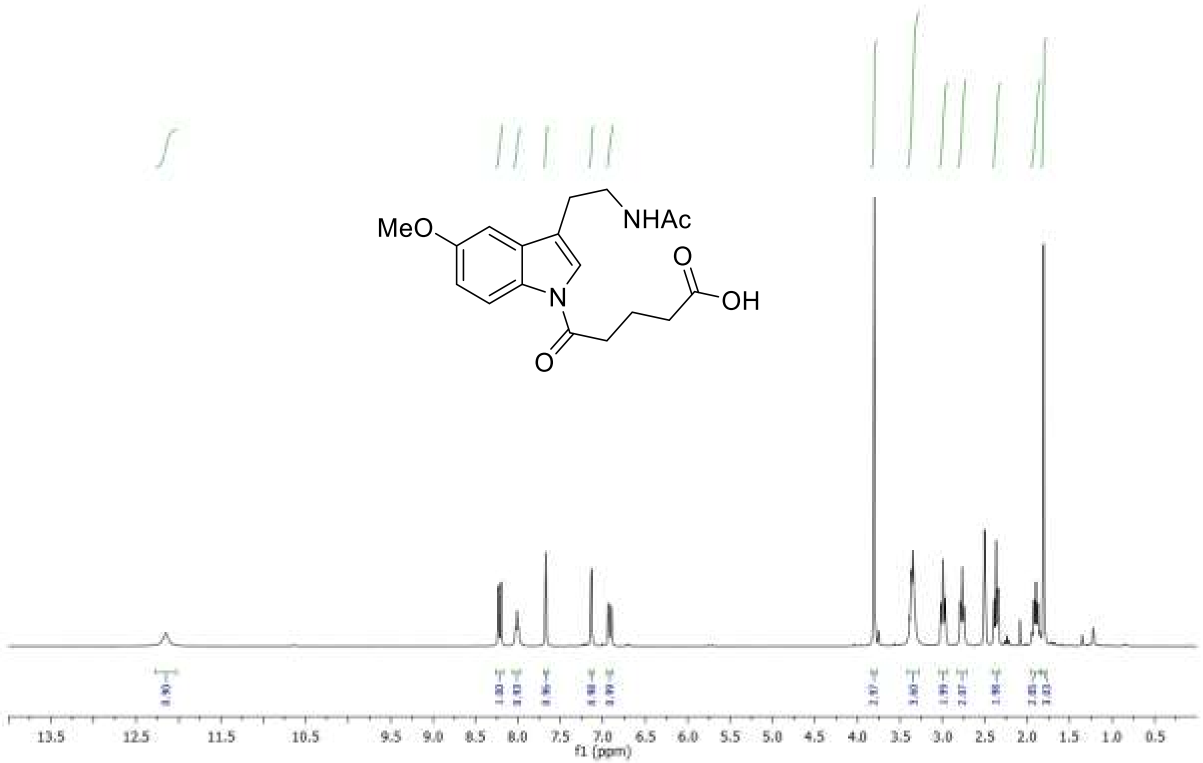
MeO
NHAc
N
O
OH
O
f1 (ppm)

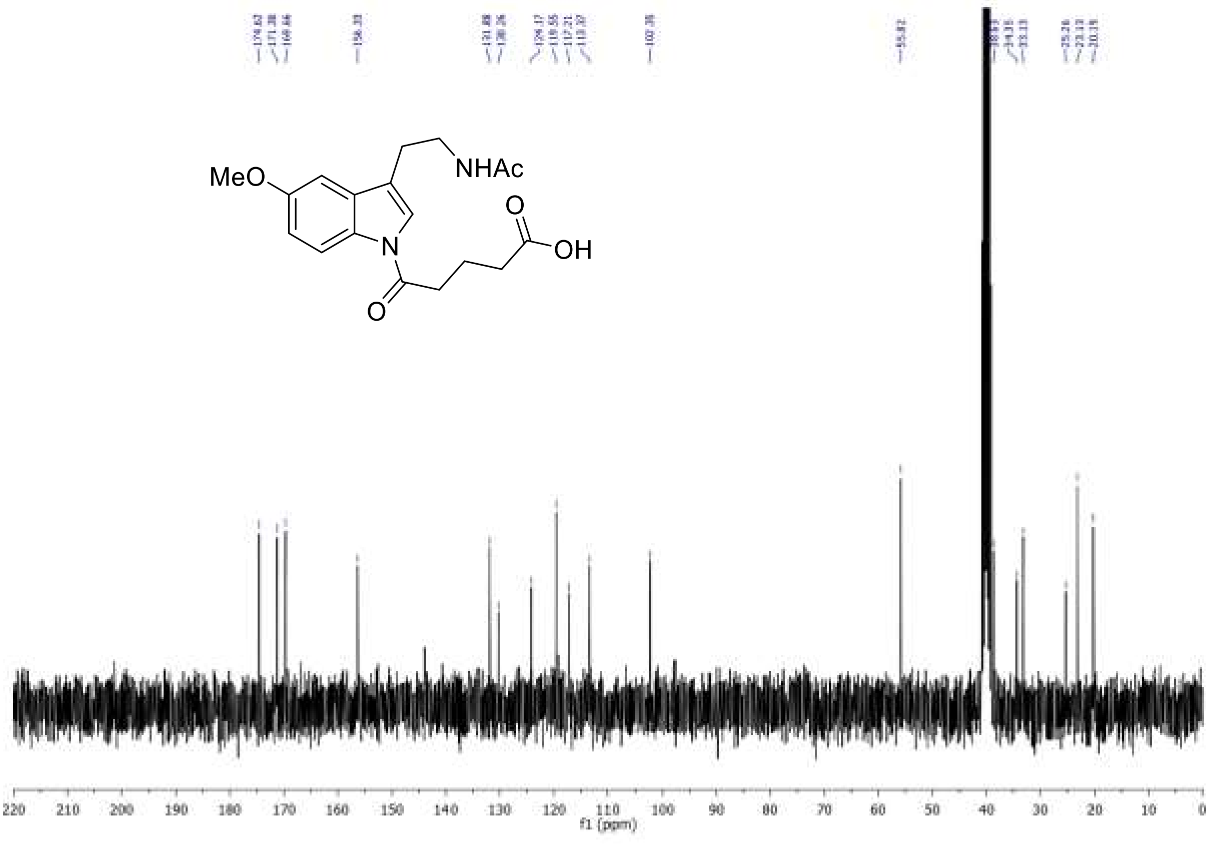
MeO
NHAc
N
O
OH
O
f1 (ppm)

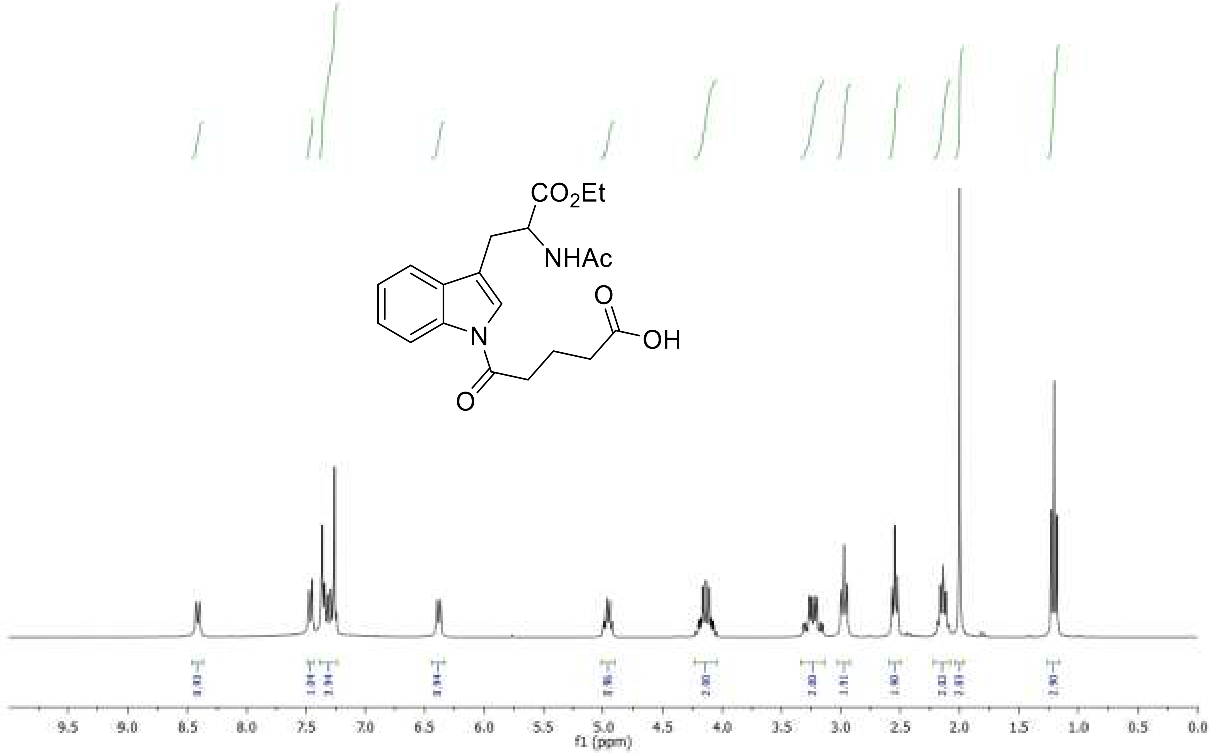
CO2Et
NHAc
N
O
OH
O
f1 (ppm)

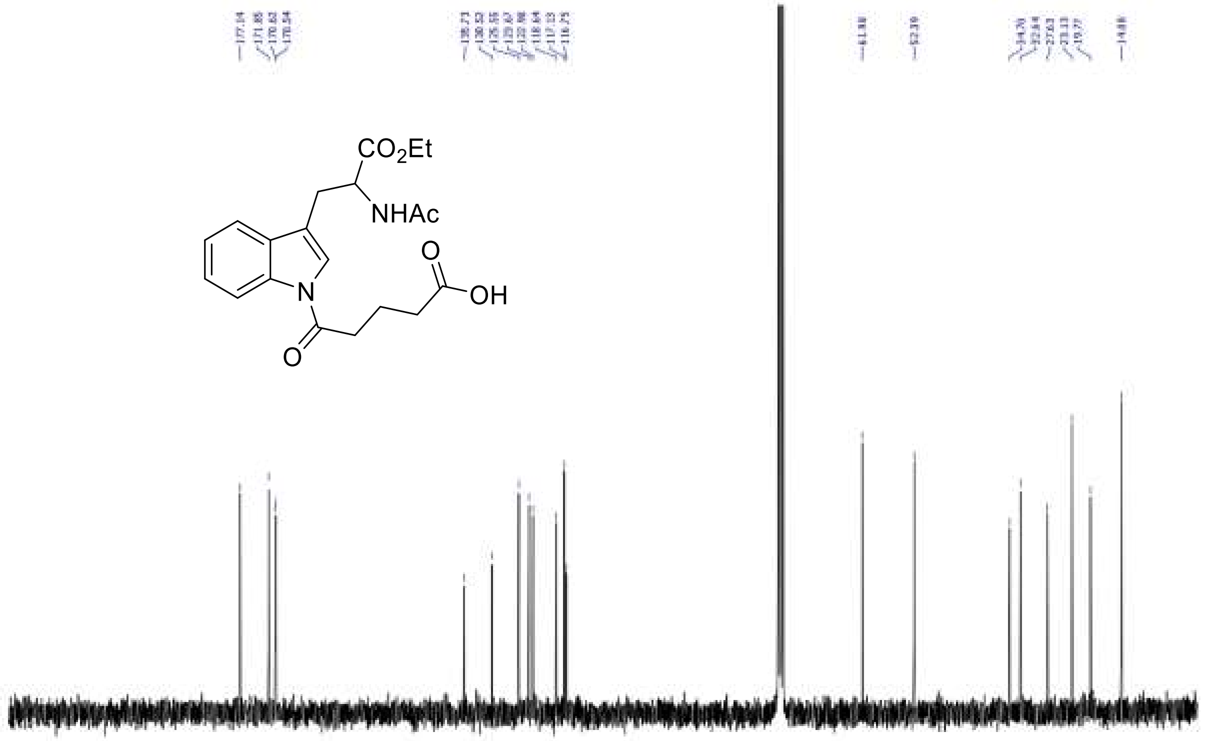
CO2Et
NHAc
N
O
OH
O
f1 (ppm)

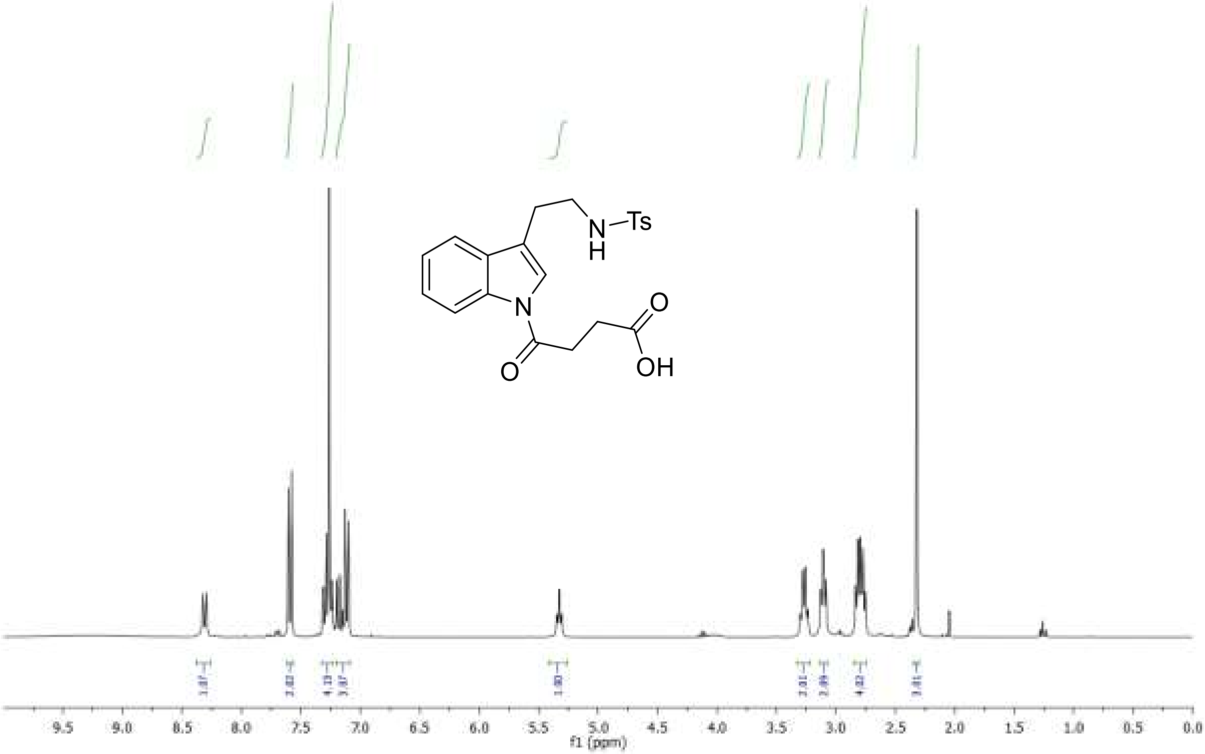
N–Ts
H
N
O
O
OH
9.5 9.0 8.5 8.0 7.5 7.0 6.5 6.0 5.5 5.0 4.5 4.0 3.5 3.0 2.5 2.0 1.5 1.0 0.5 0.0
f1 (ppm)

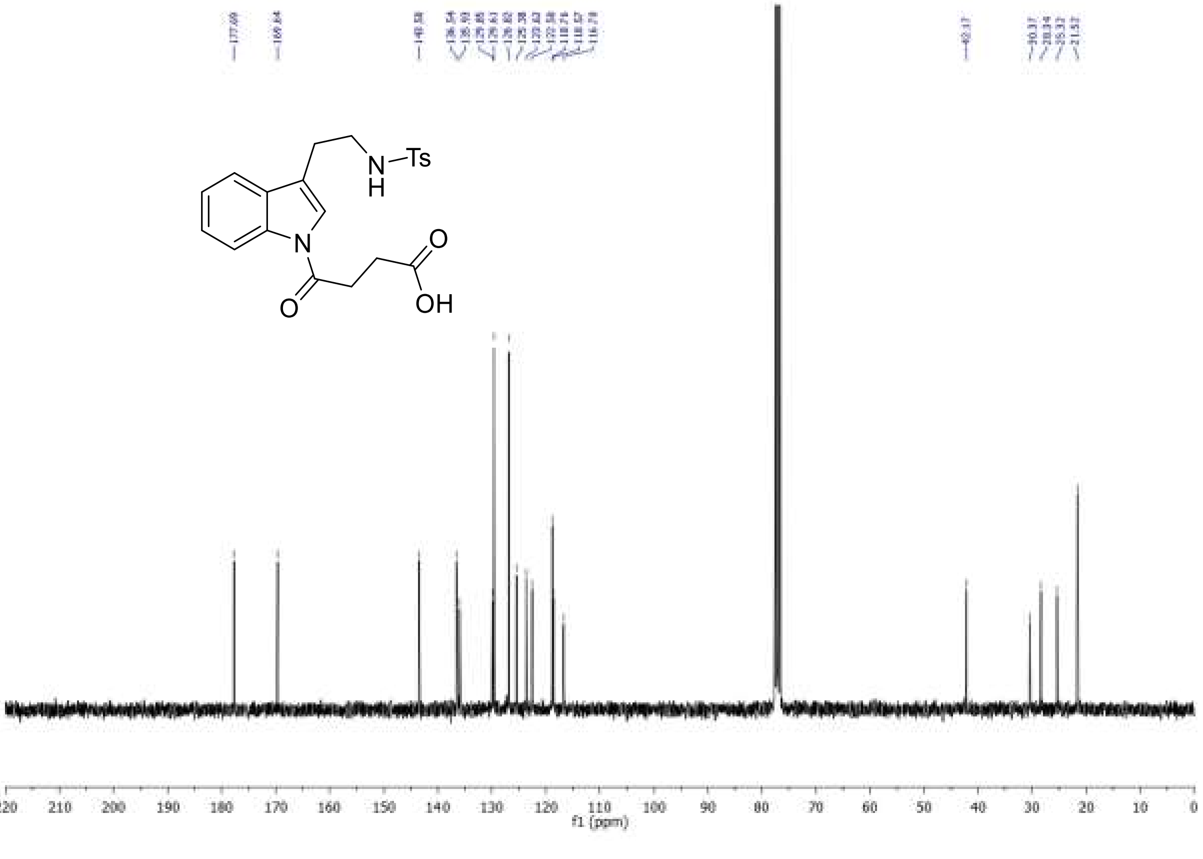
N–Ts
H
N
O
O
OH
220 210 200 190 180 170 160 150 140 130 120 110 100 90 80 70 60 50 40 30 20 10 0
f1 (ppm)

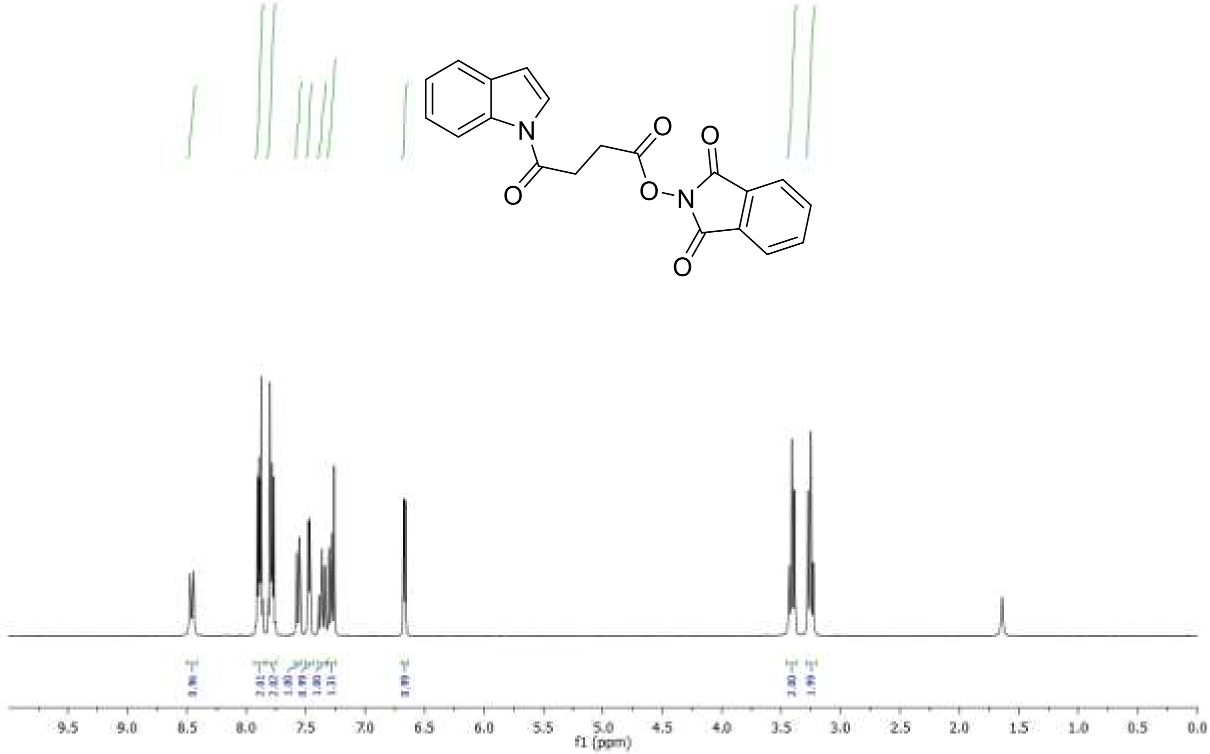

f1 (ppm)

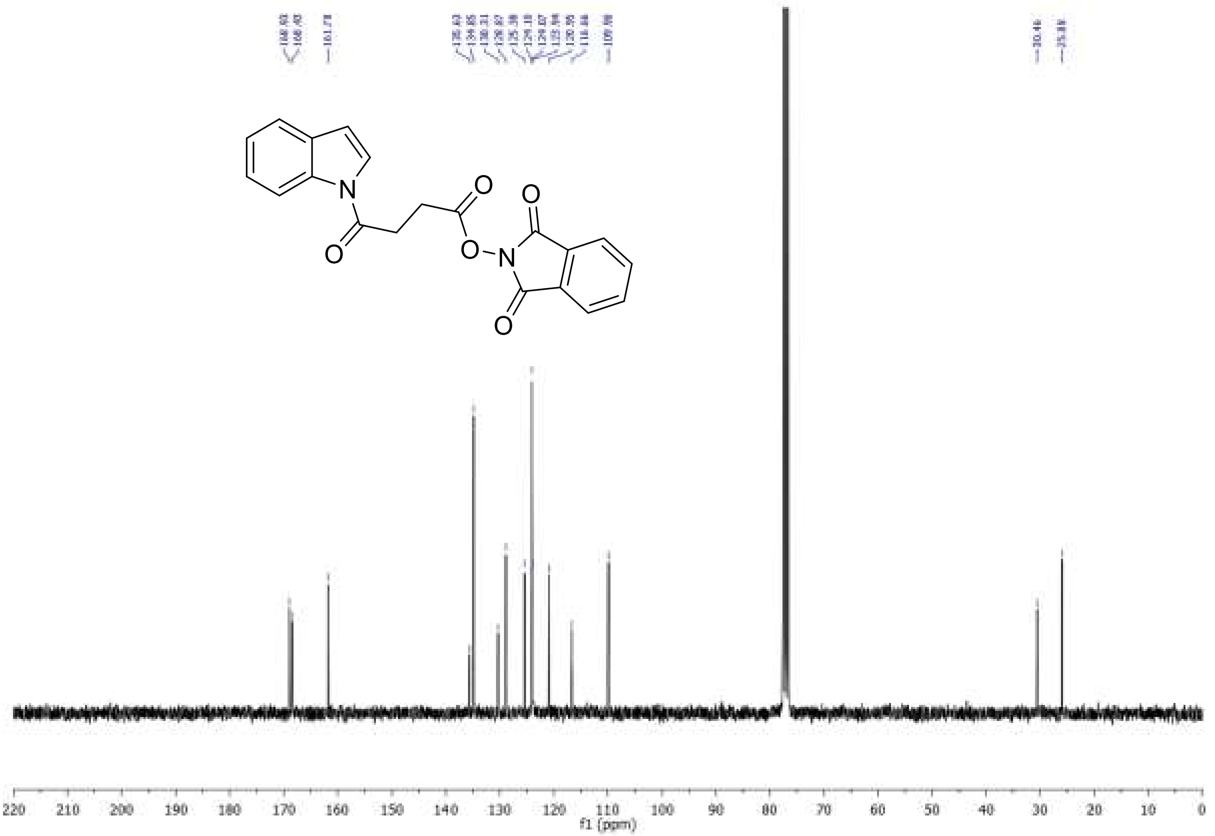

f1 (ppm)

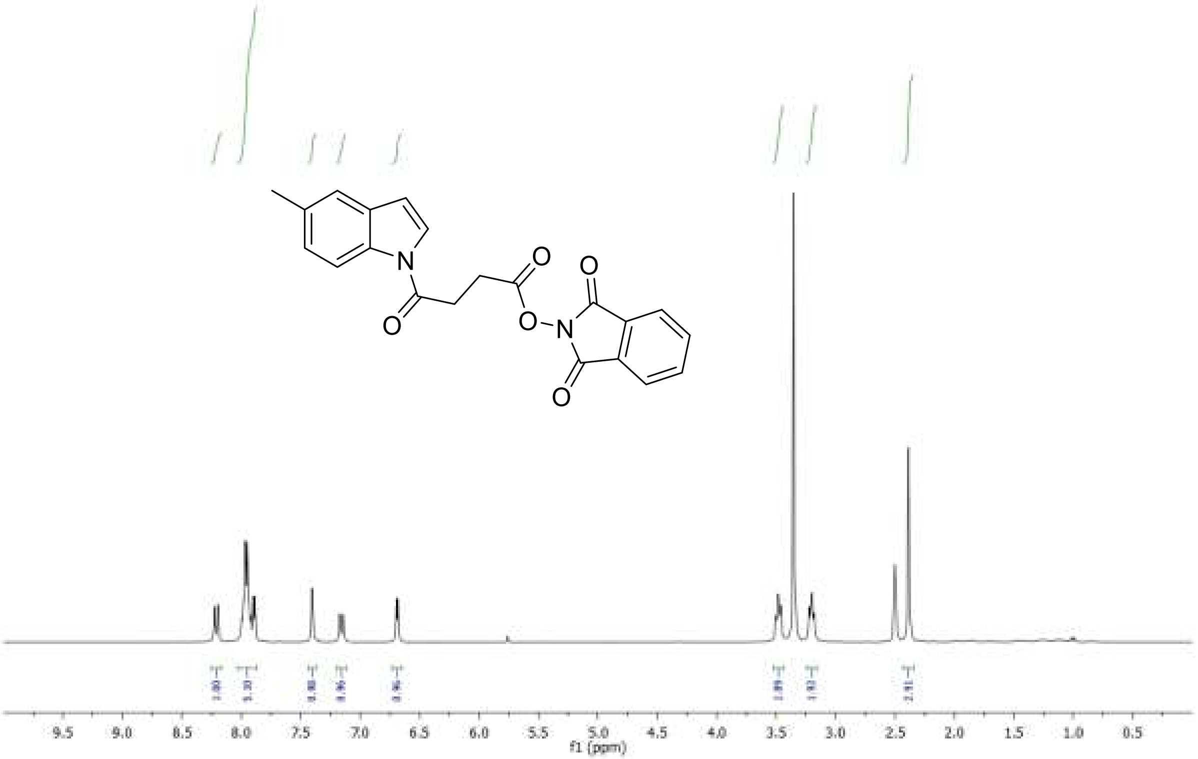

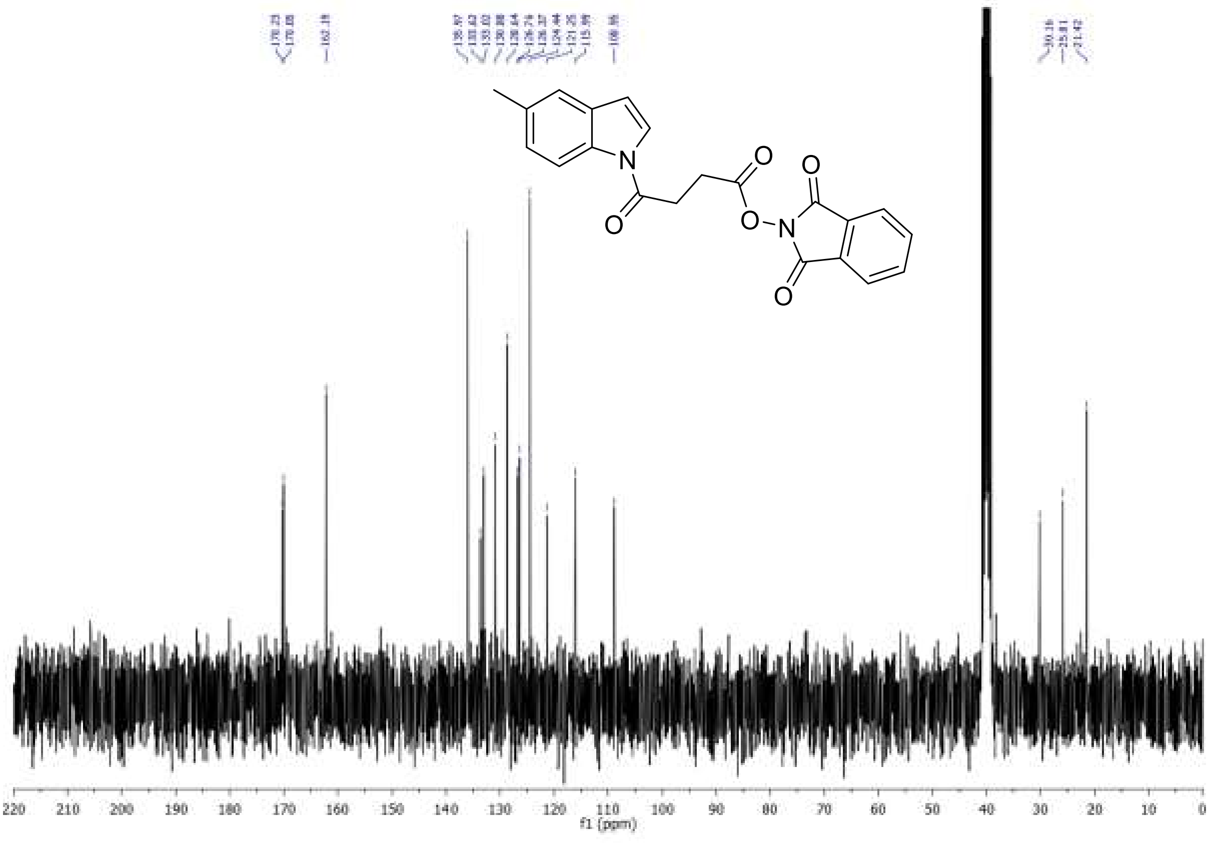

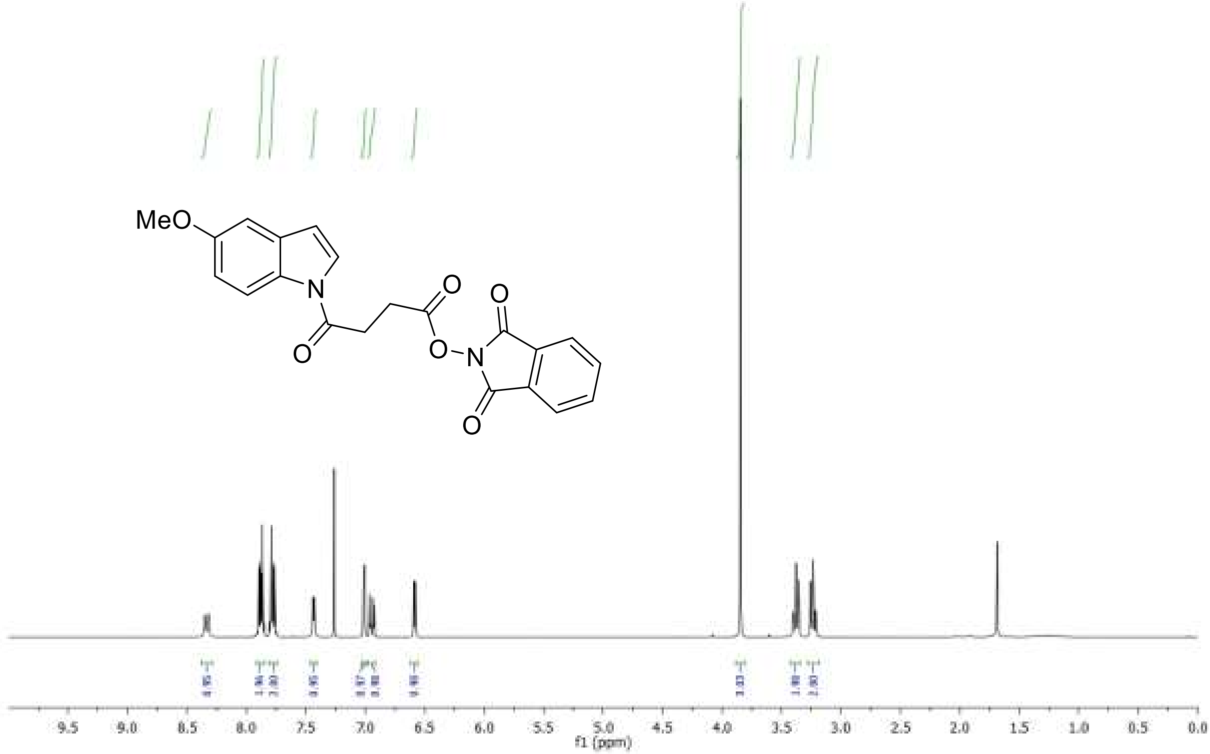

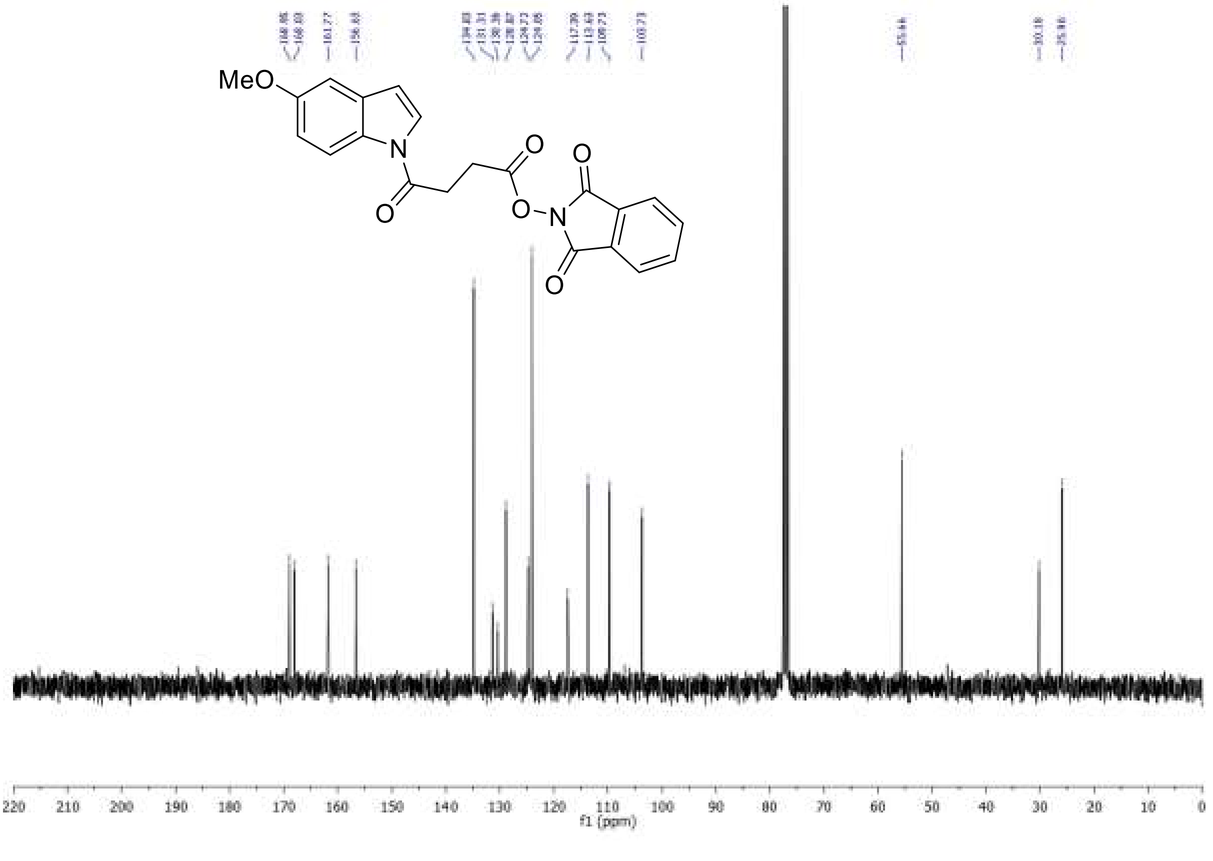

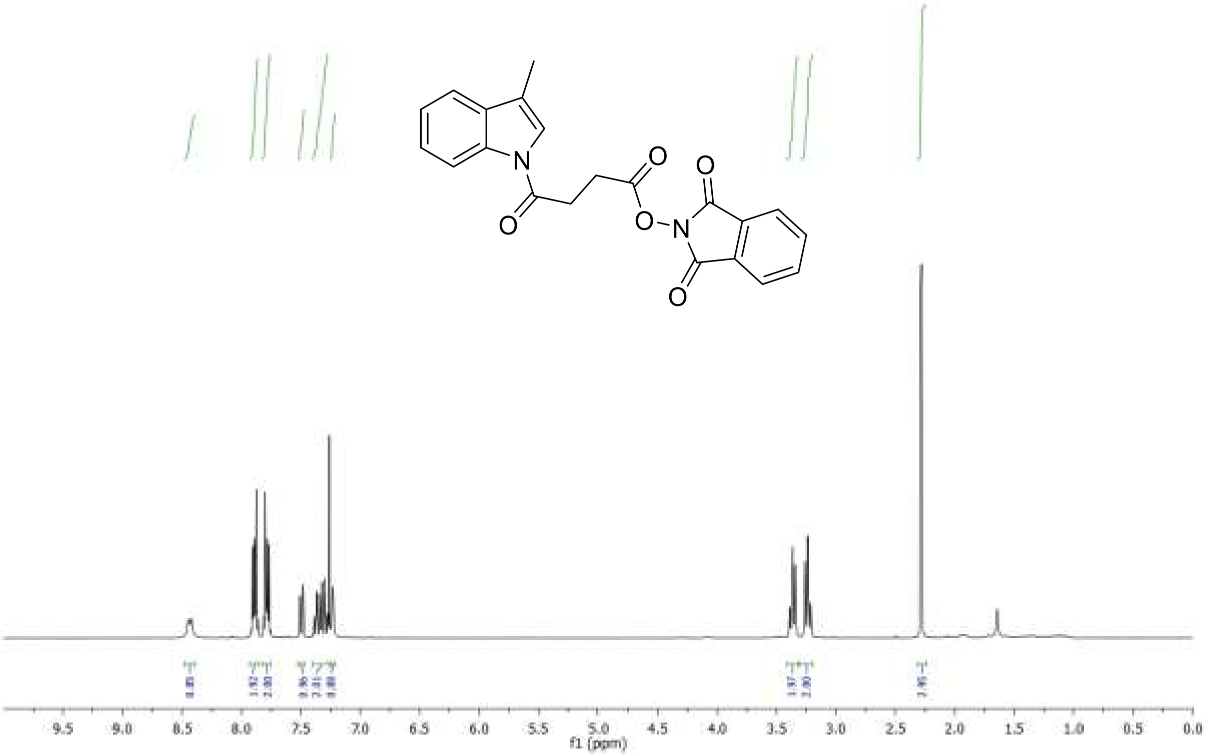

f1 (ppm)

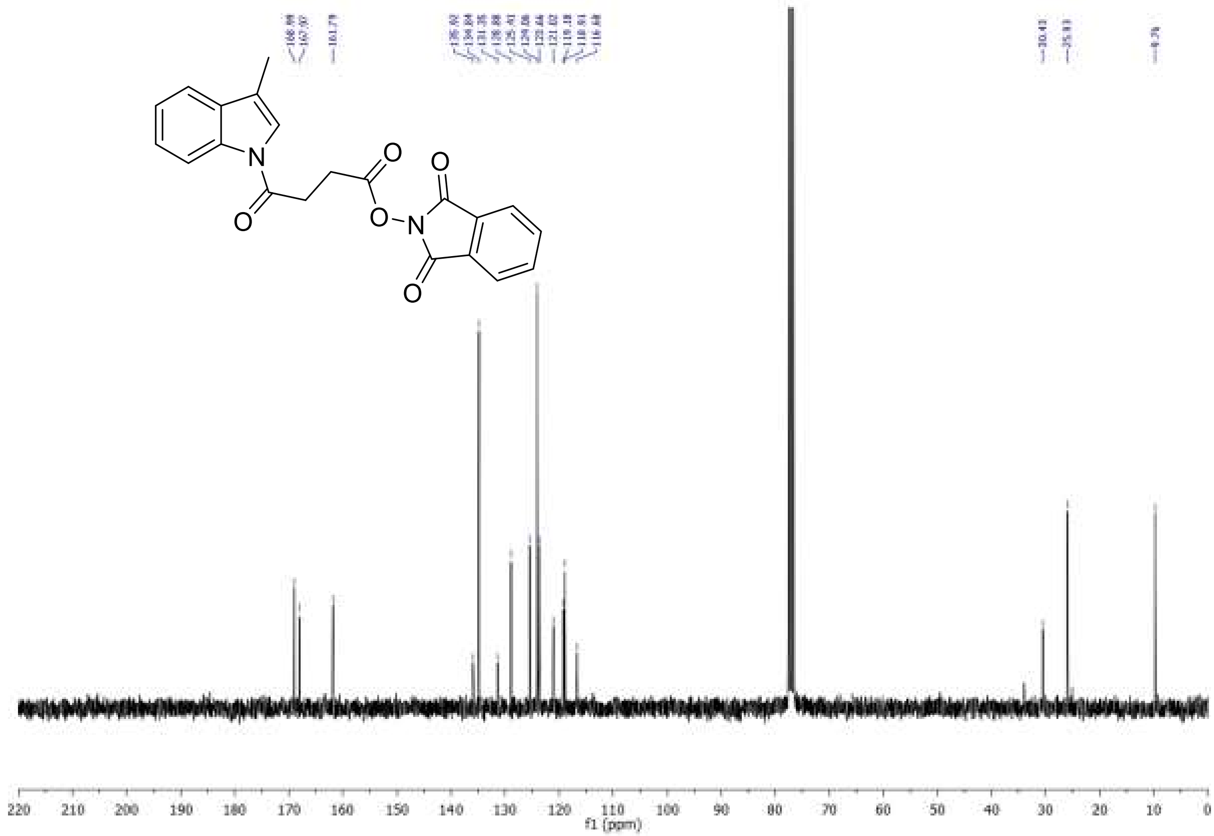

f1 (ppm)

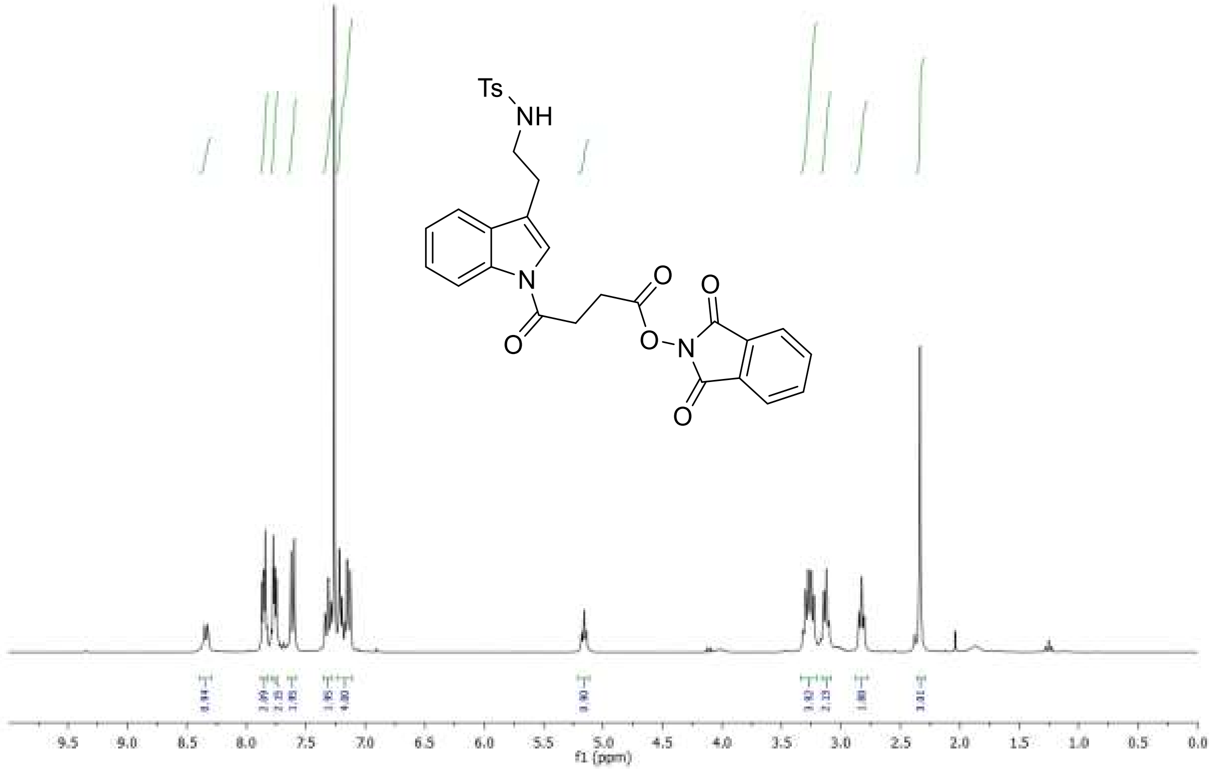

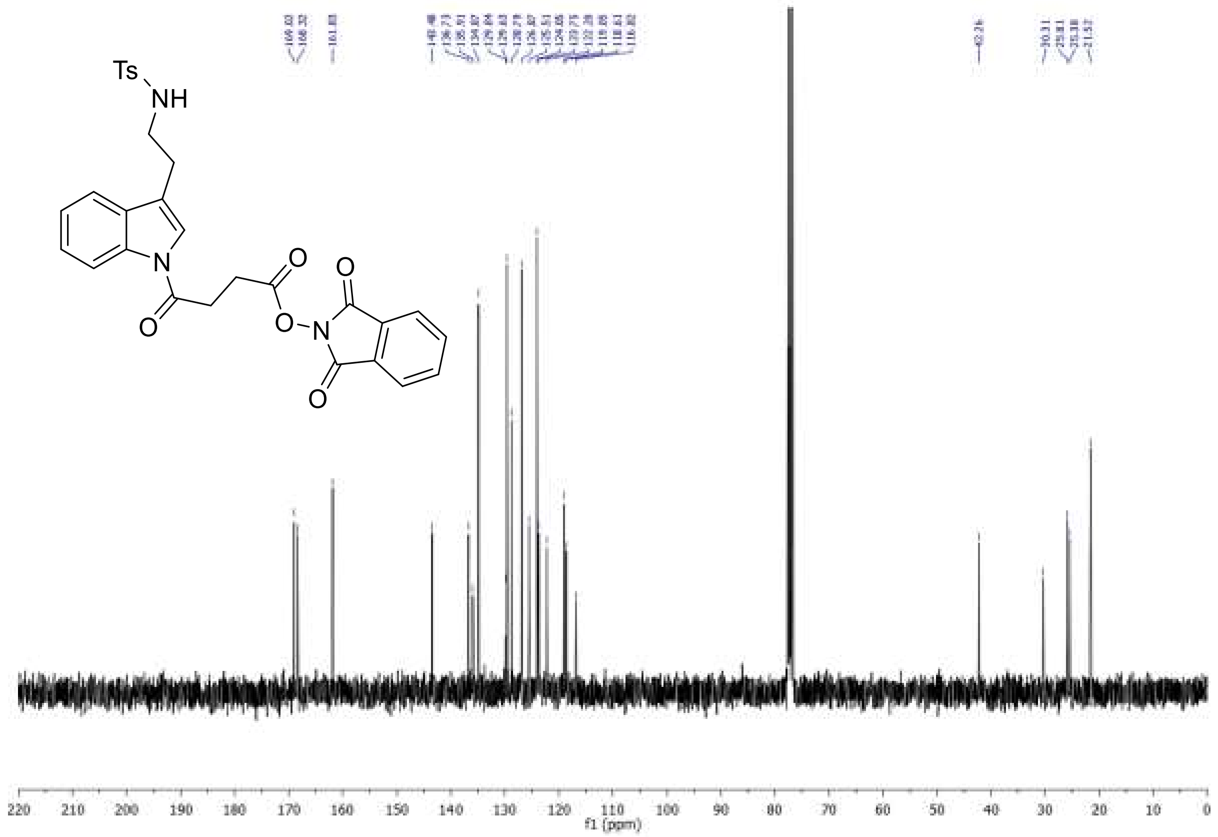

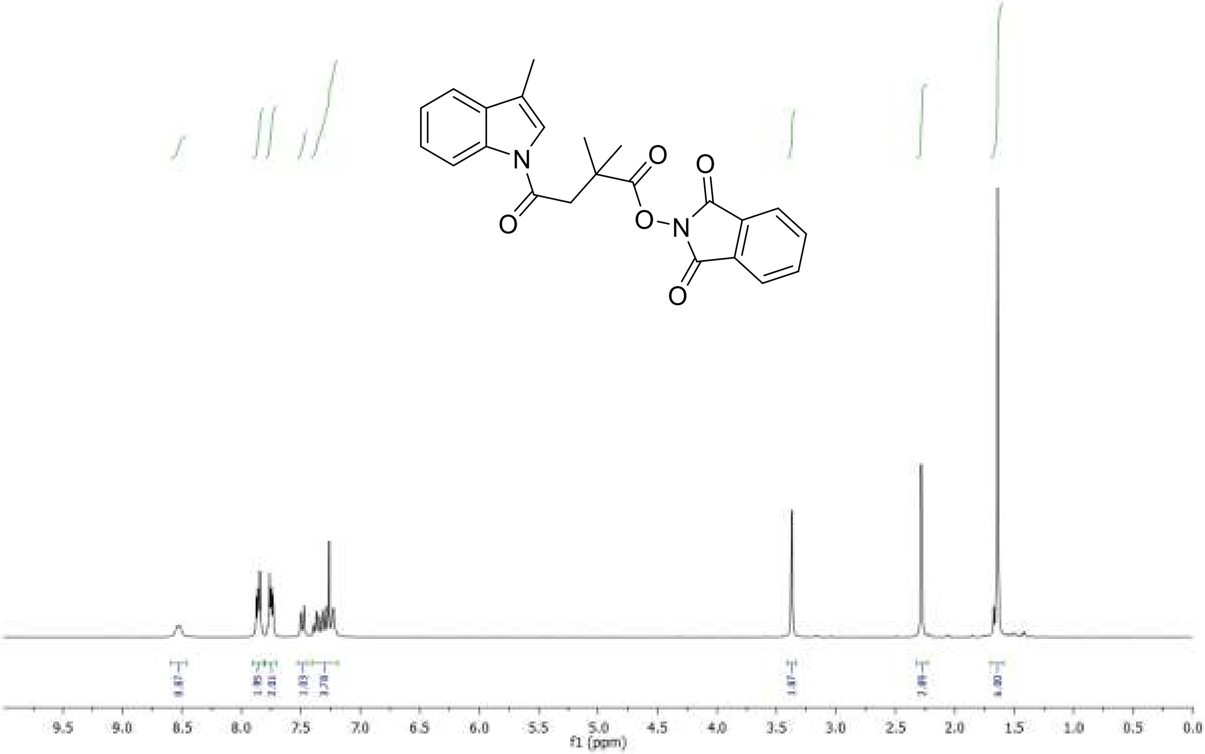

f1 (ppm)

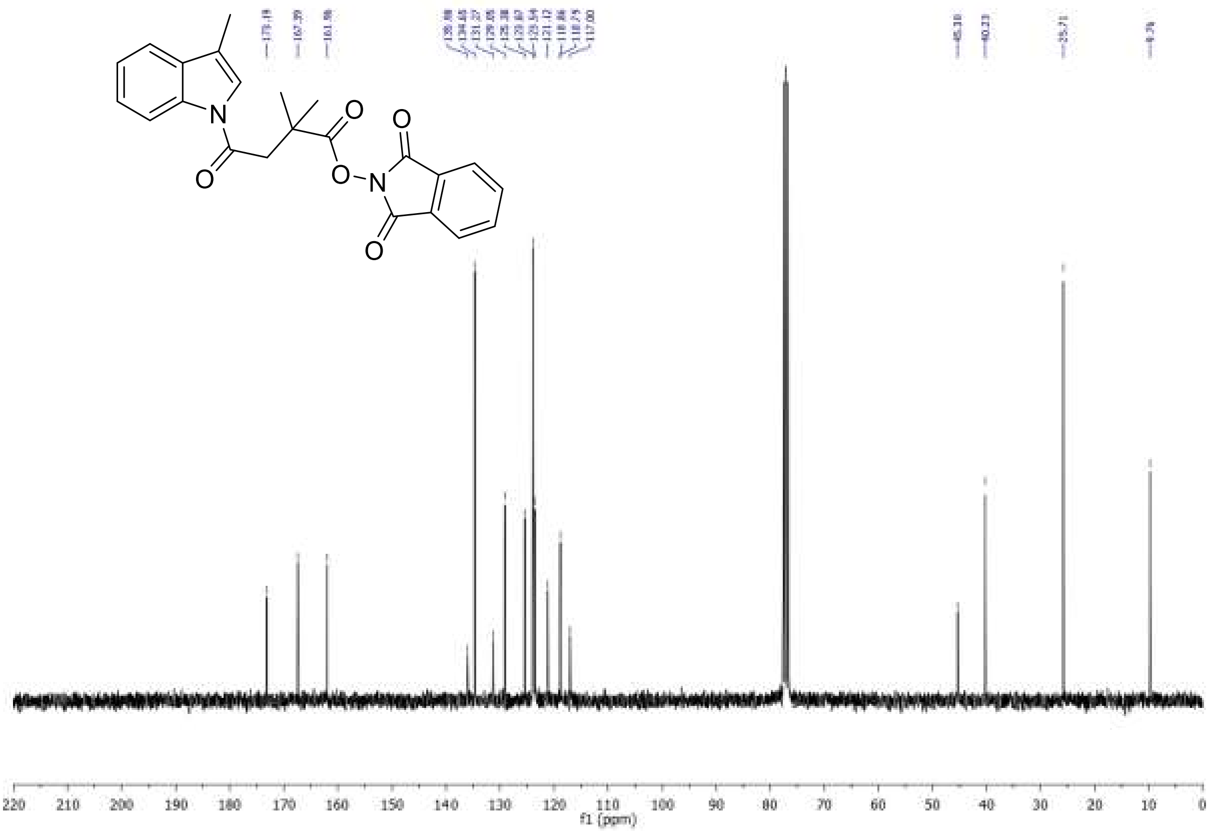

f1 (ppm)

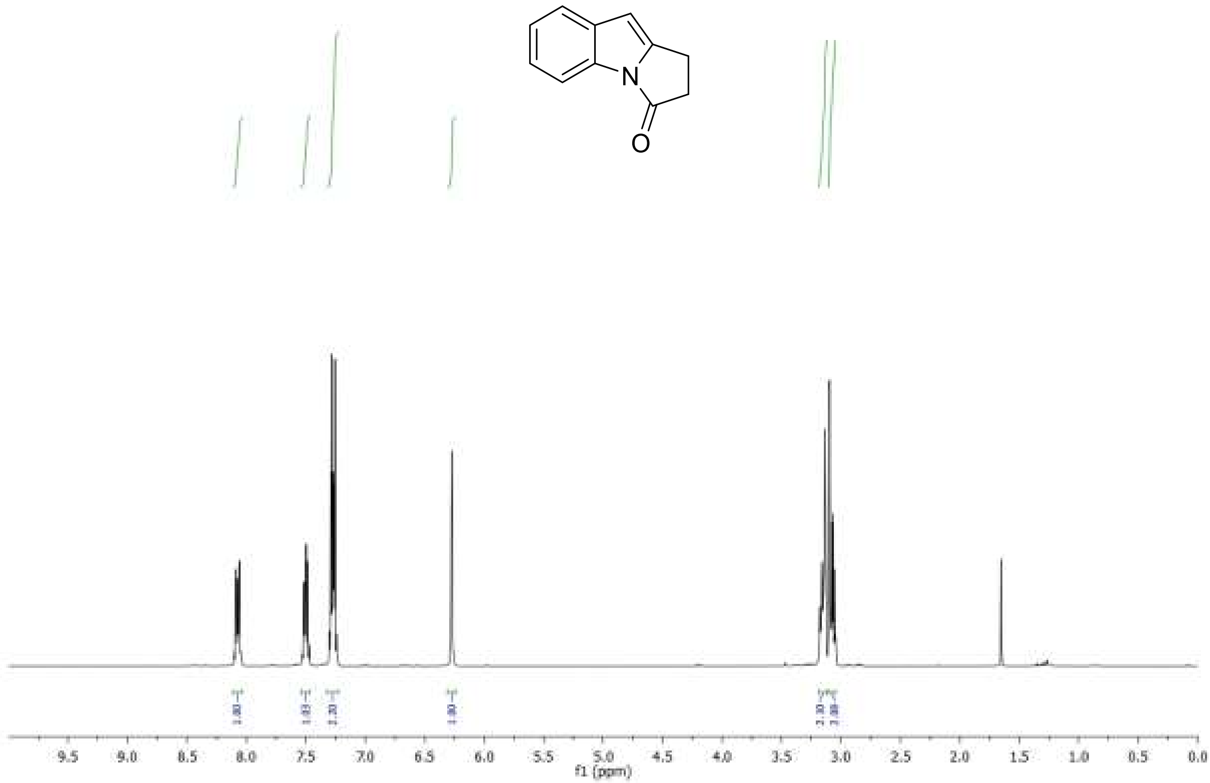
N
O
9.5 9.0 8.5 8.0 7.5 7.0 6.5 6.0 5.5 5.0 4.5 4.0 3.5 3.0 2.5 2.0 1.5 1.0 0.5 0.0
f1 (ppm)

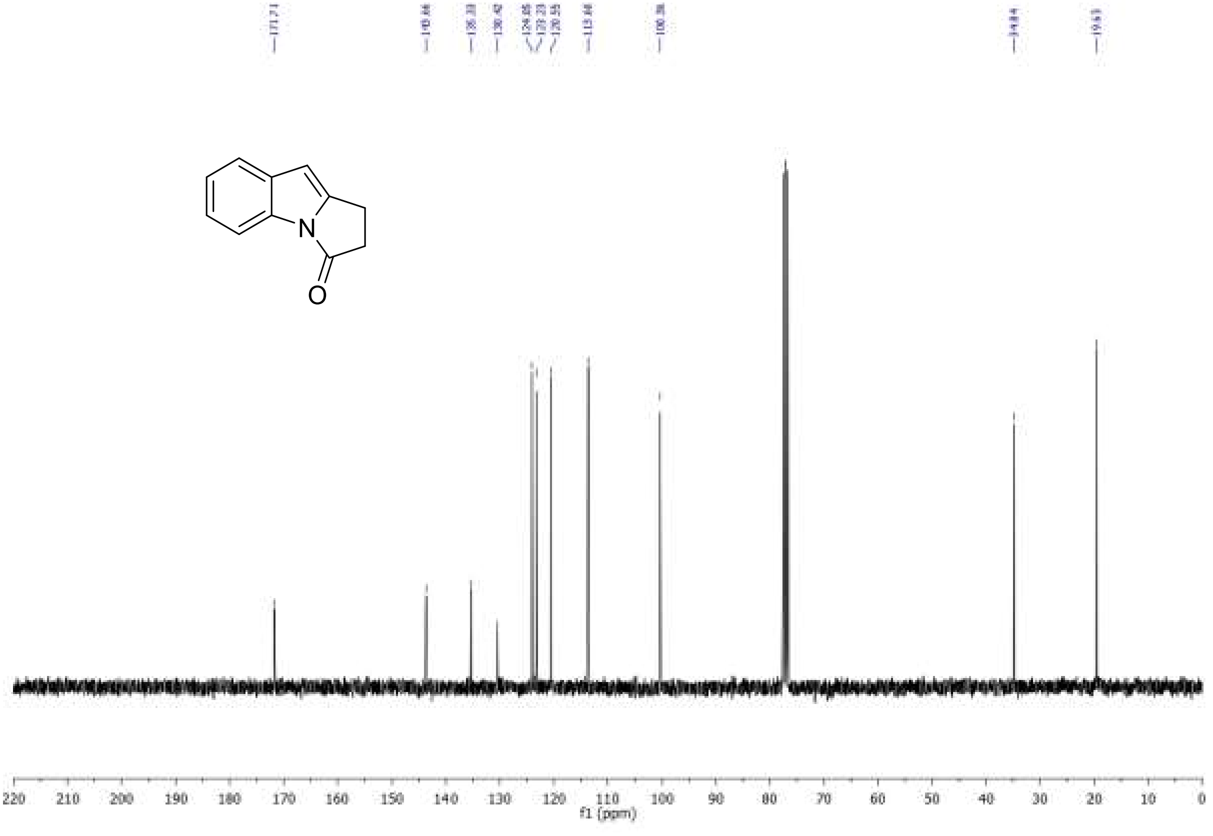
N
O
220 210 200 190 180 170 160 150 140 130 120 110 100 90 80 70 60 50 40 30 20 10 0
f1 (ppm)

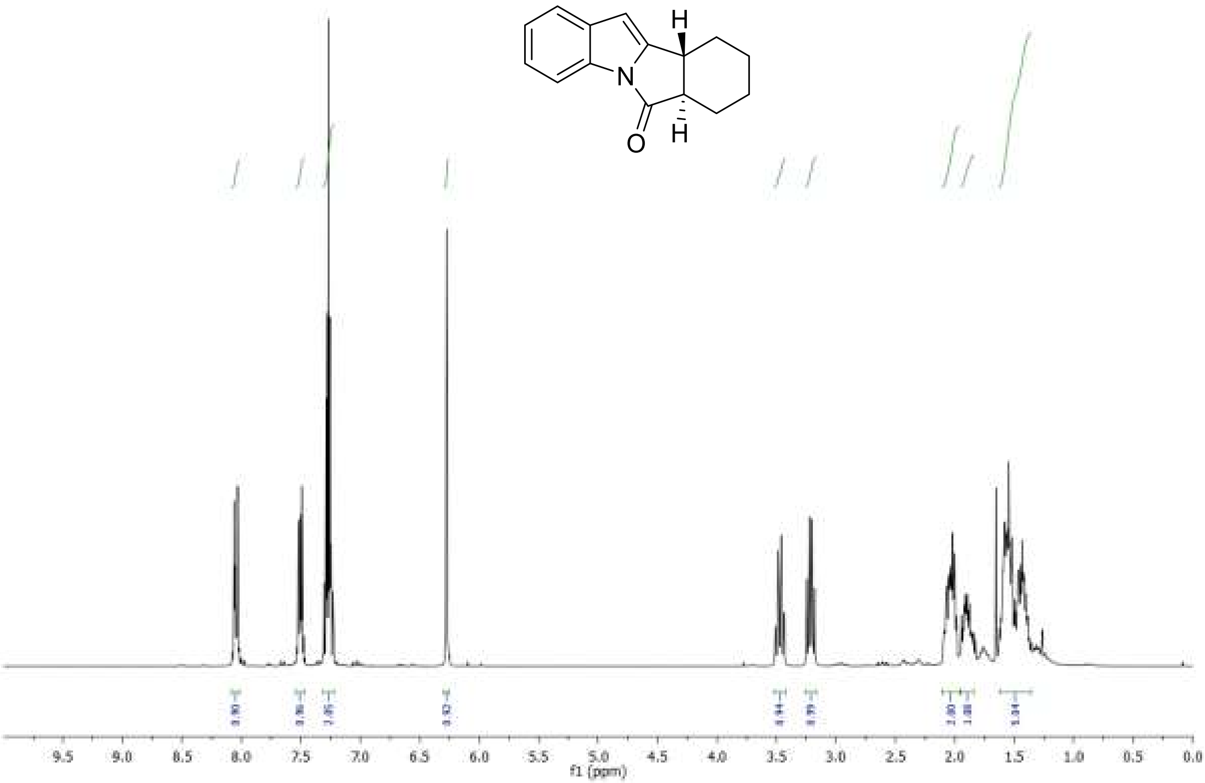
9.5 9.0 8.5 8.0 7.5 7.0 6.5 6.0 5.5 5.0 4.5 4.0 3.5 3.0 2.5 2.0 1.5 1.0 0.5 0.0
f1 (ppm)

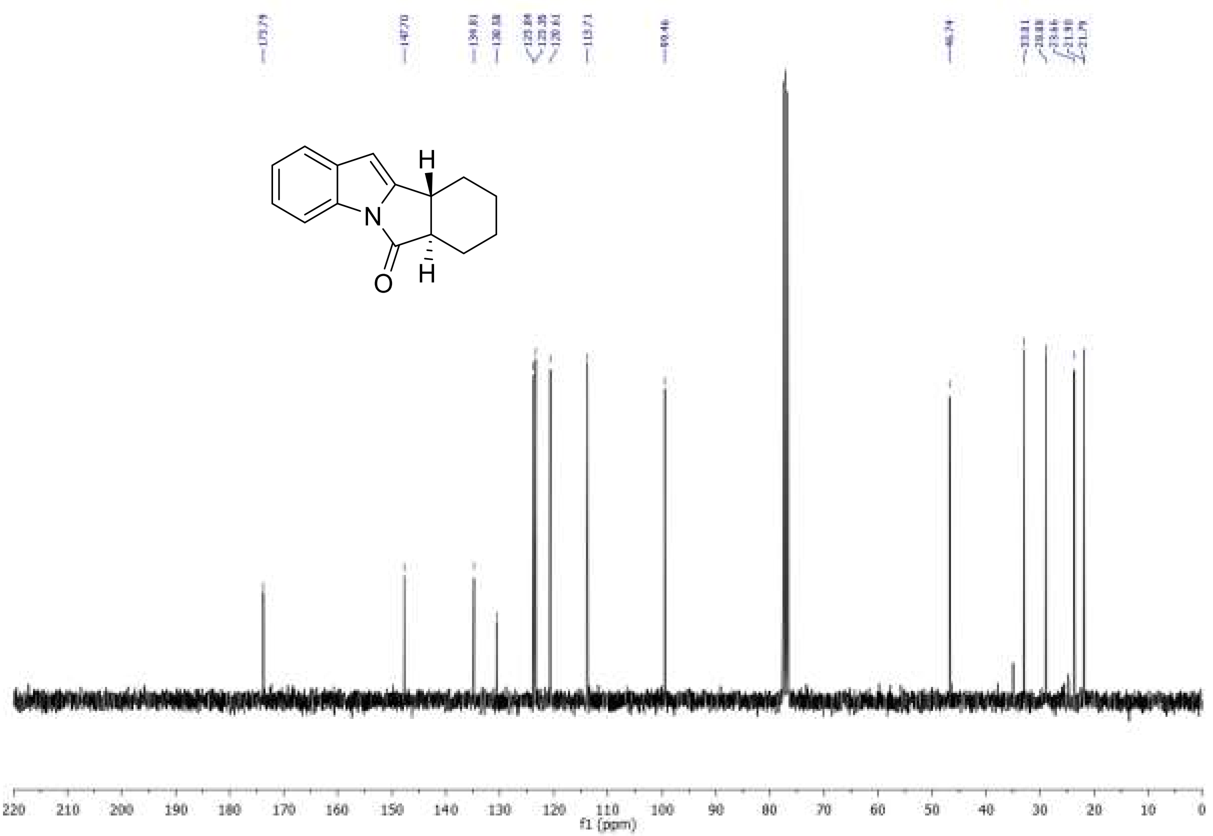
220 210 200 190 180 170 160 150 140 130 120 110 100 90 80 70 60 50 40 30 20 10 0
f1 (ppm)

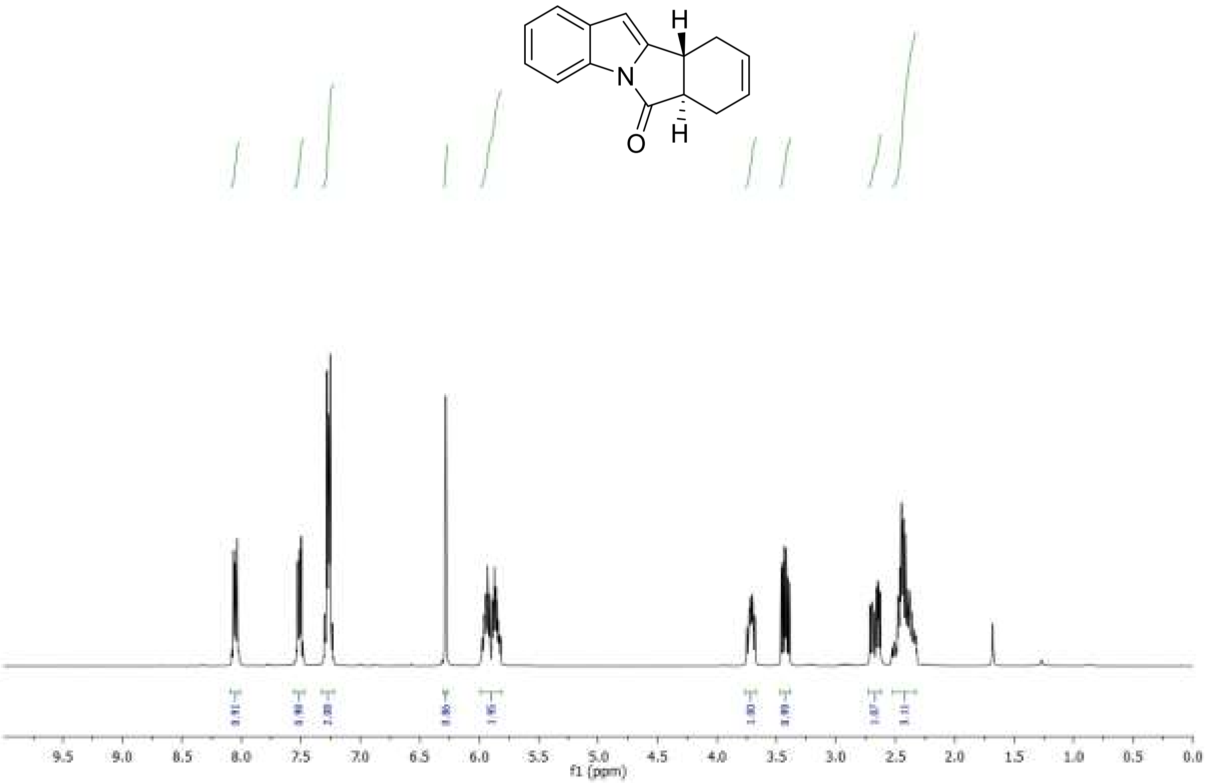
f1 (ppm)

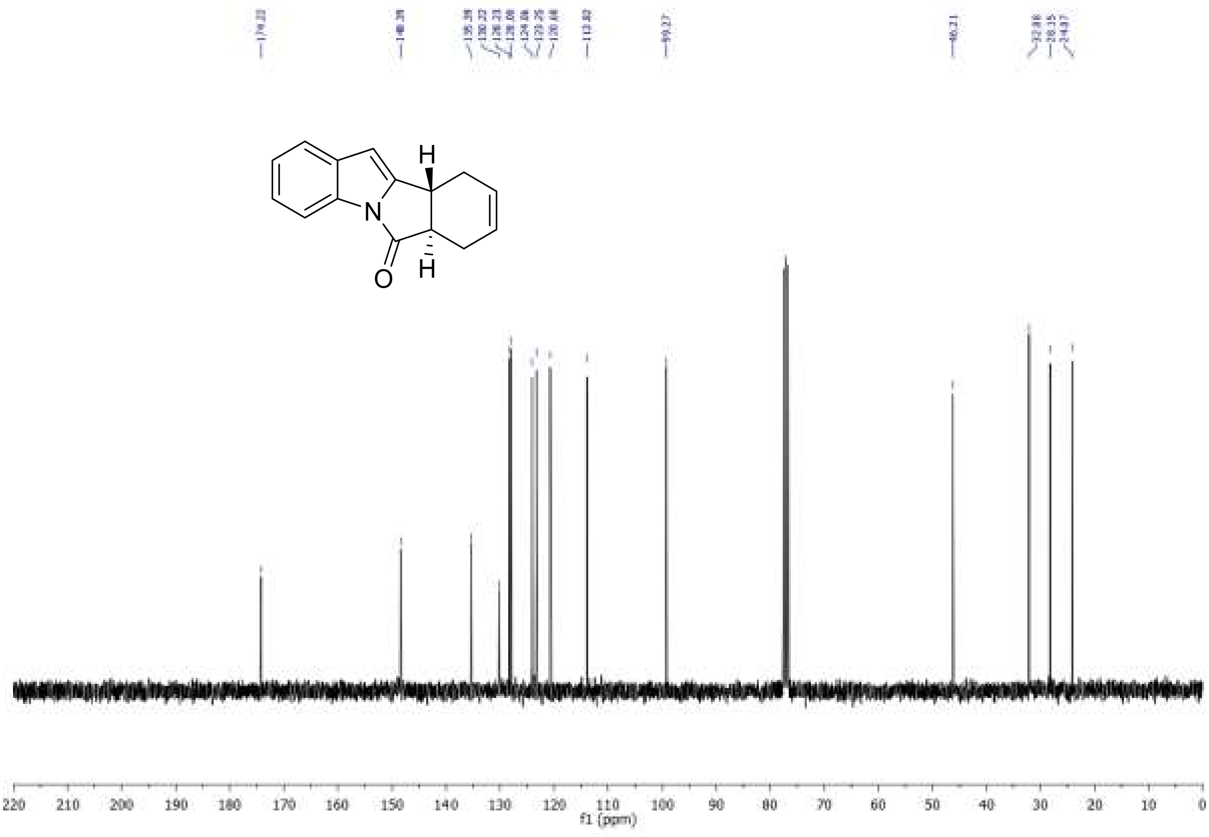
f1 (ppm)

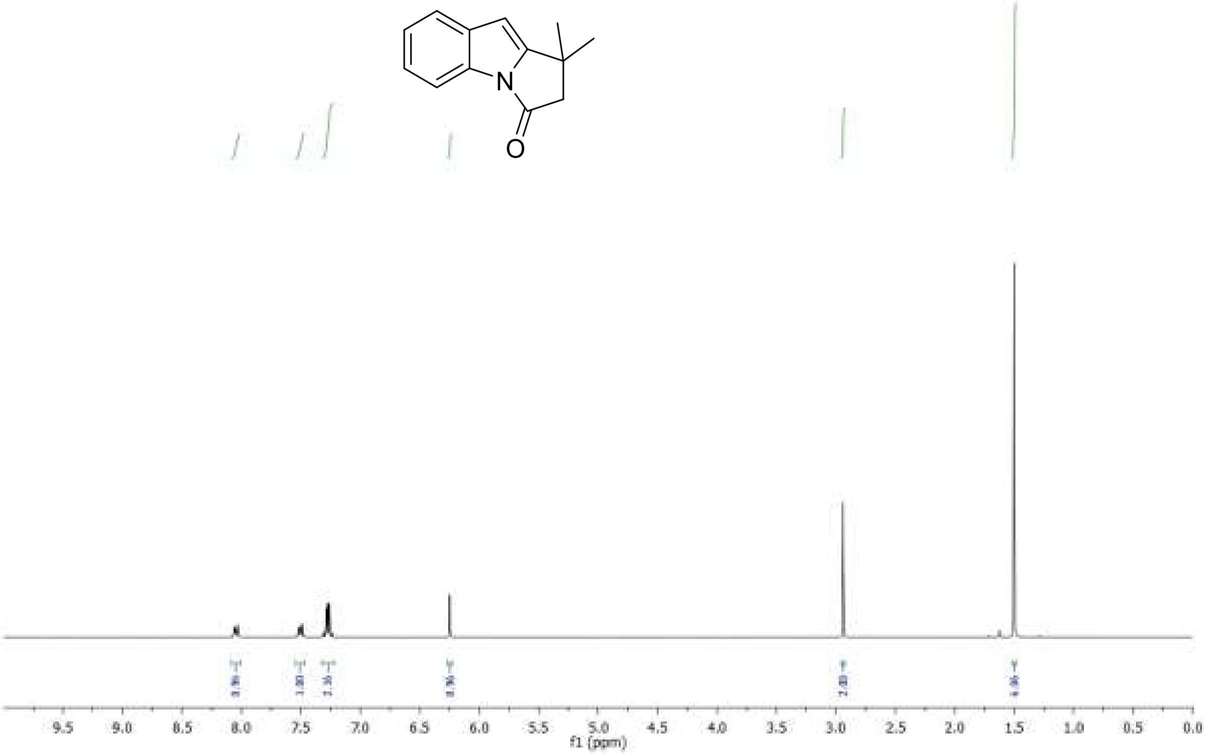

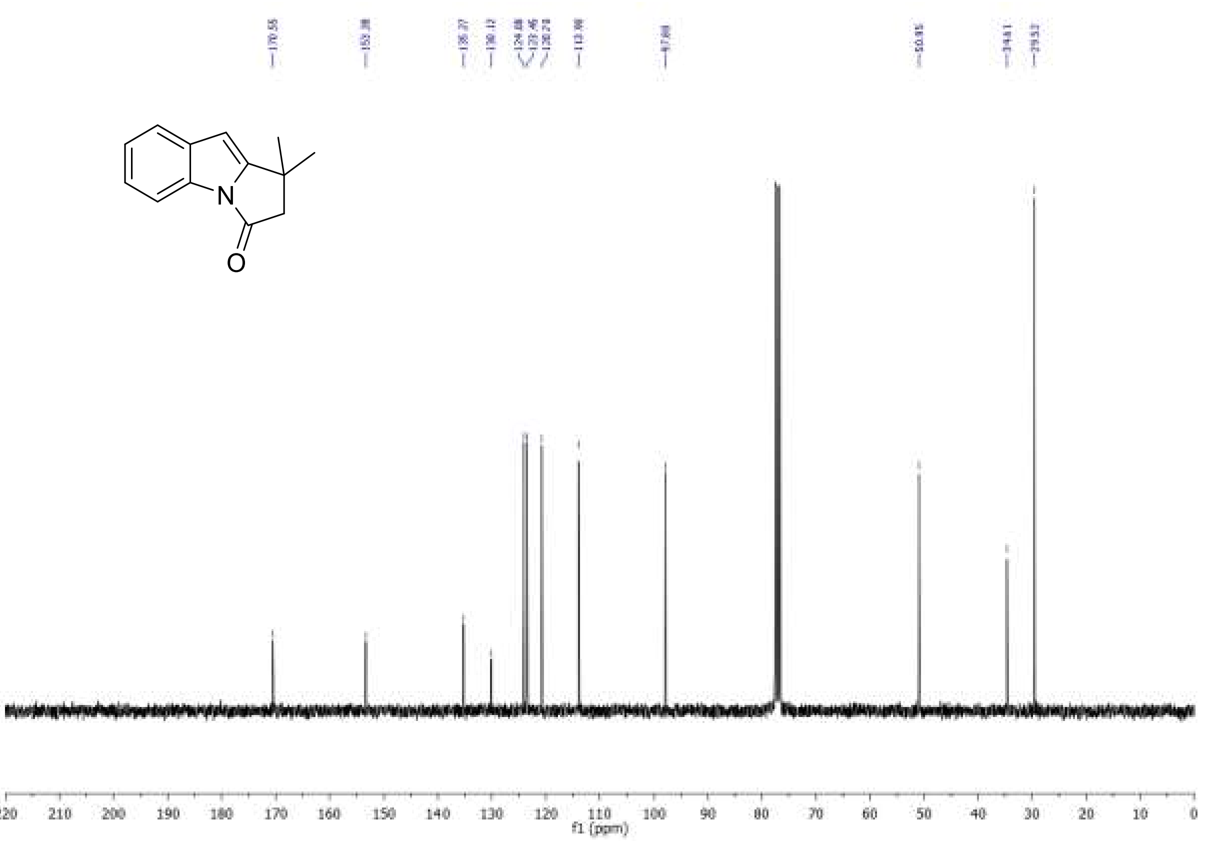

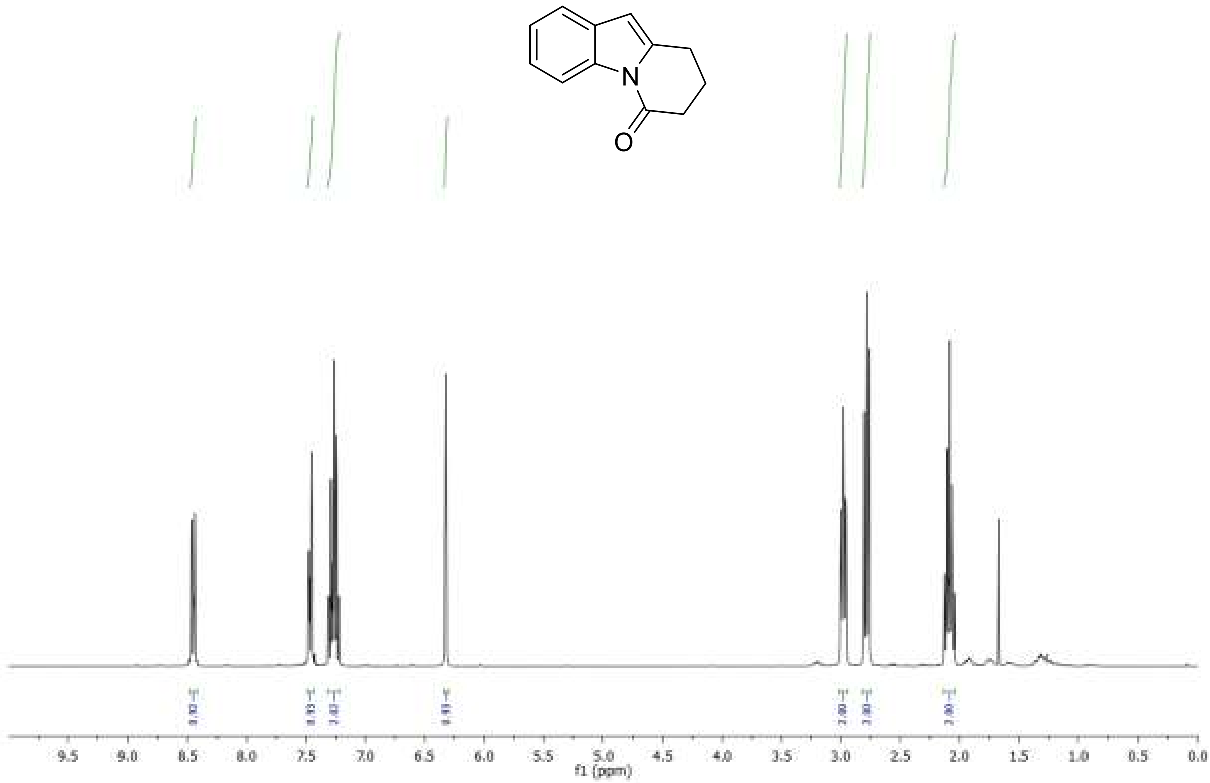
N
O
9.5 9.0 8.5 8.0 7.5 7.0 6.5 6.0 5.5 5.0 4.5 4.0 3.5 3.0 2.5 2.0 1.5 1.0 0.5 0.0
f1 (ppm)

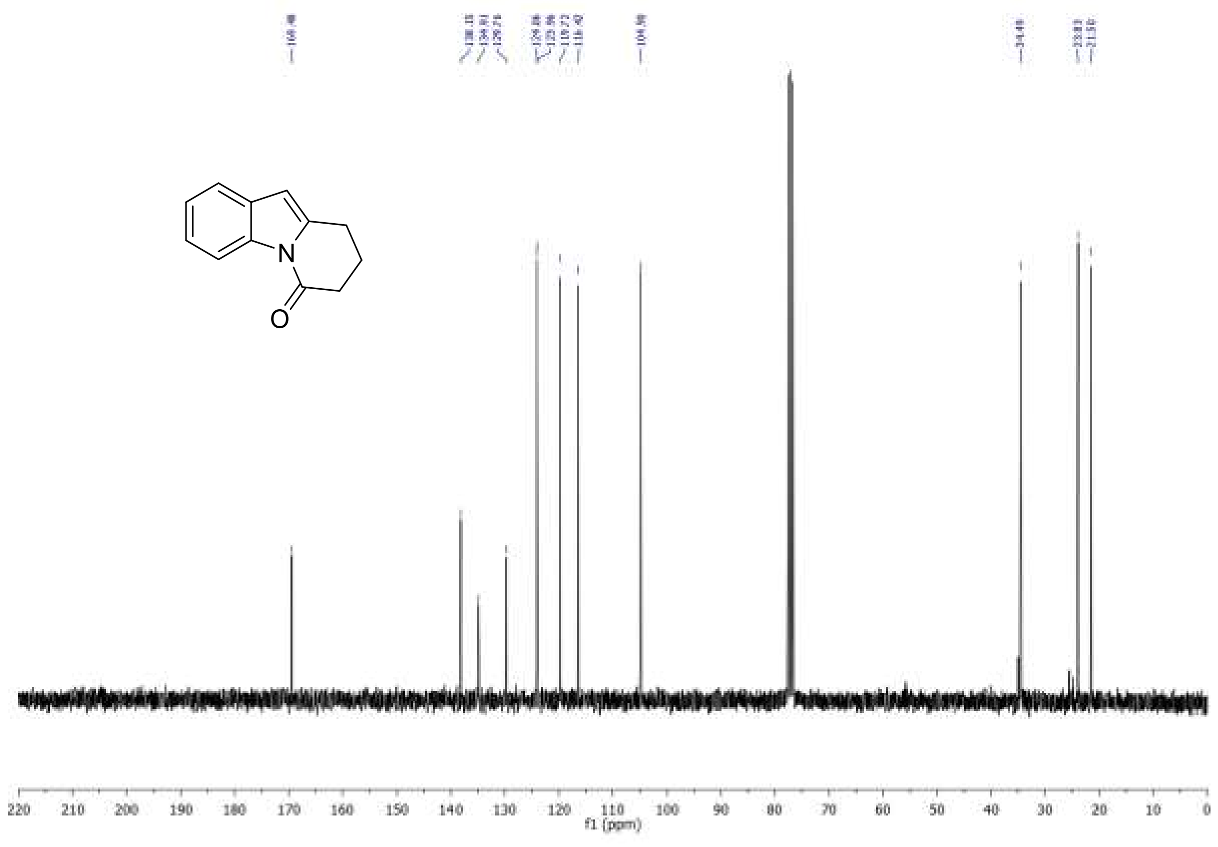
N
O
220 210 200 190 180 170 160 150 140 130 120 110 100 90 80 70 60 50 40 30 20 10 0
f1 (ppm)

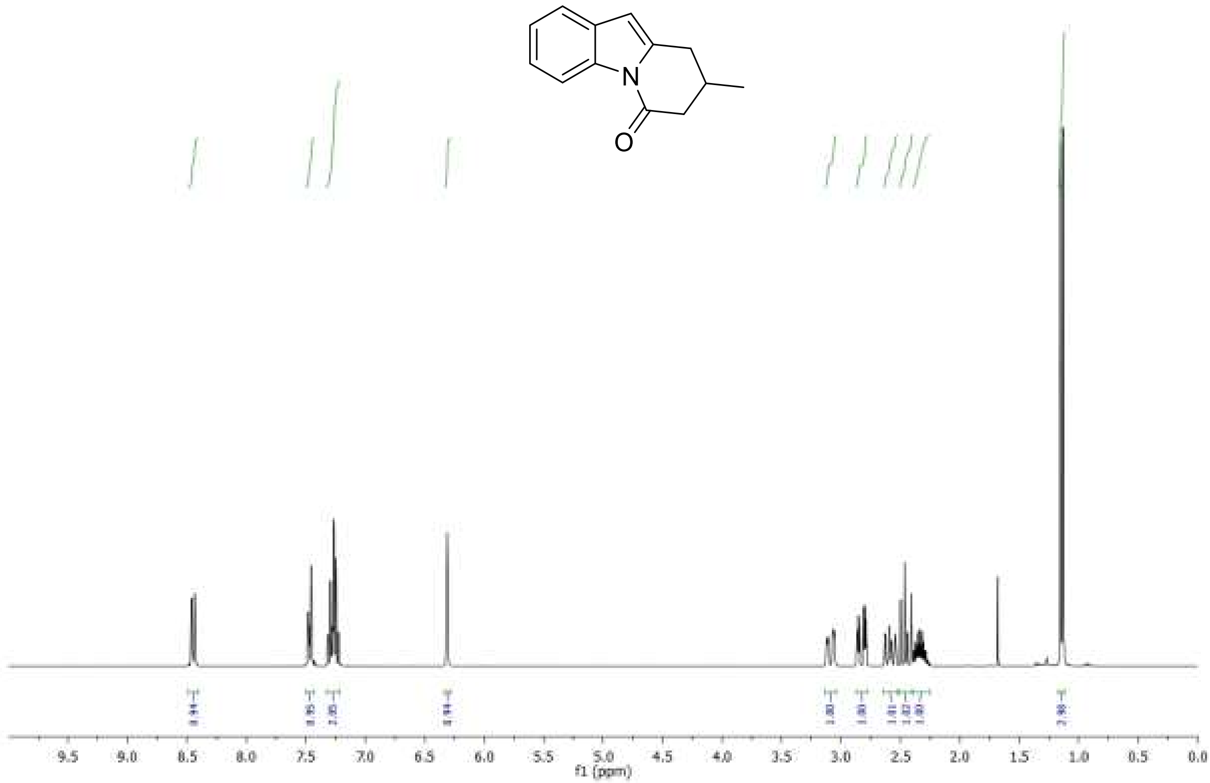
N
O
f1 (ppm)

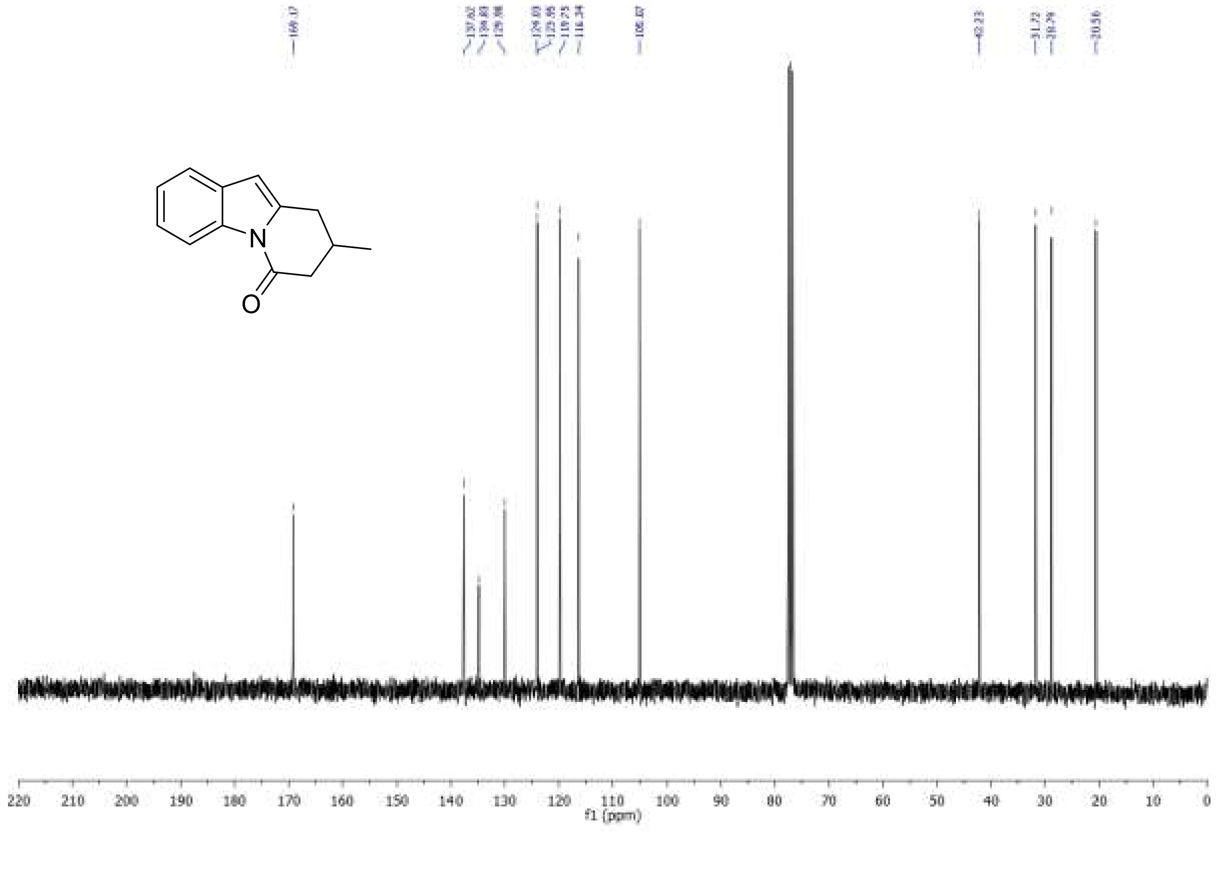
N
O
f1 (ppm)

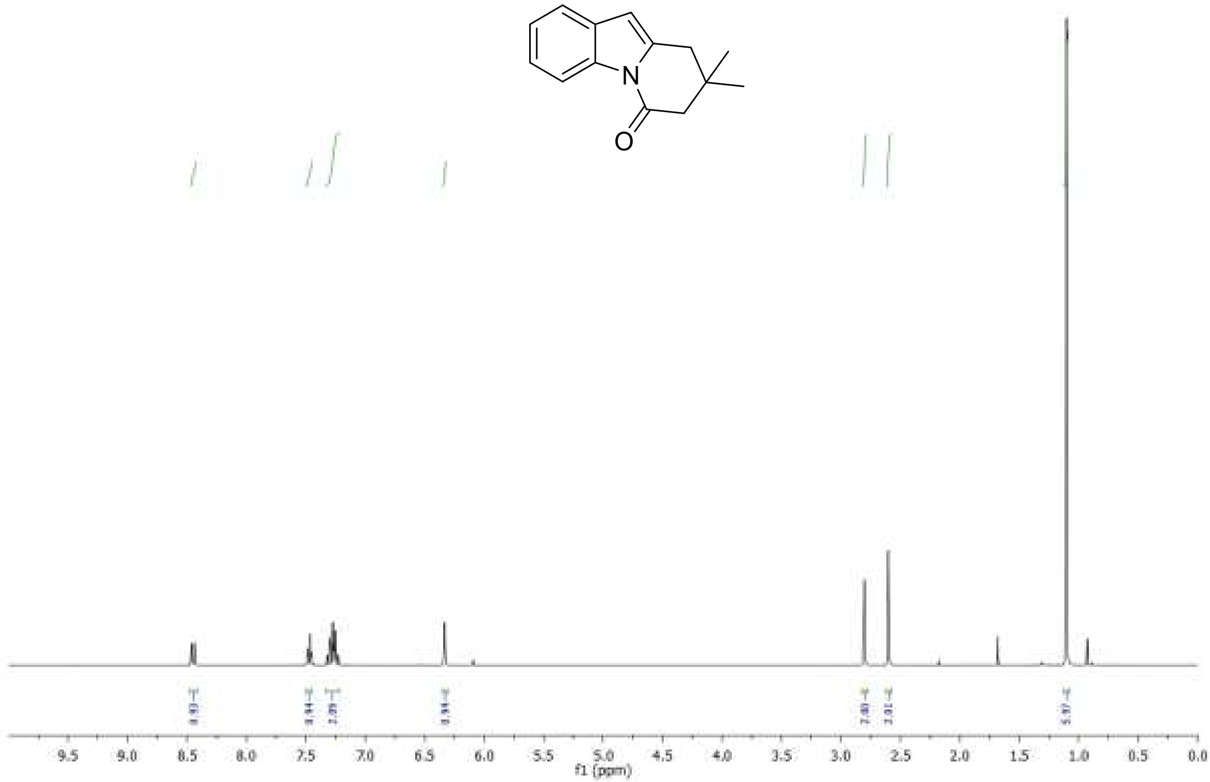

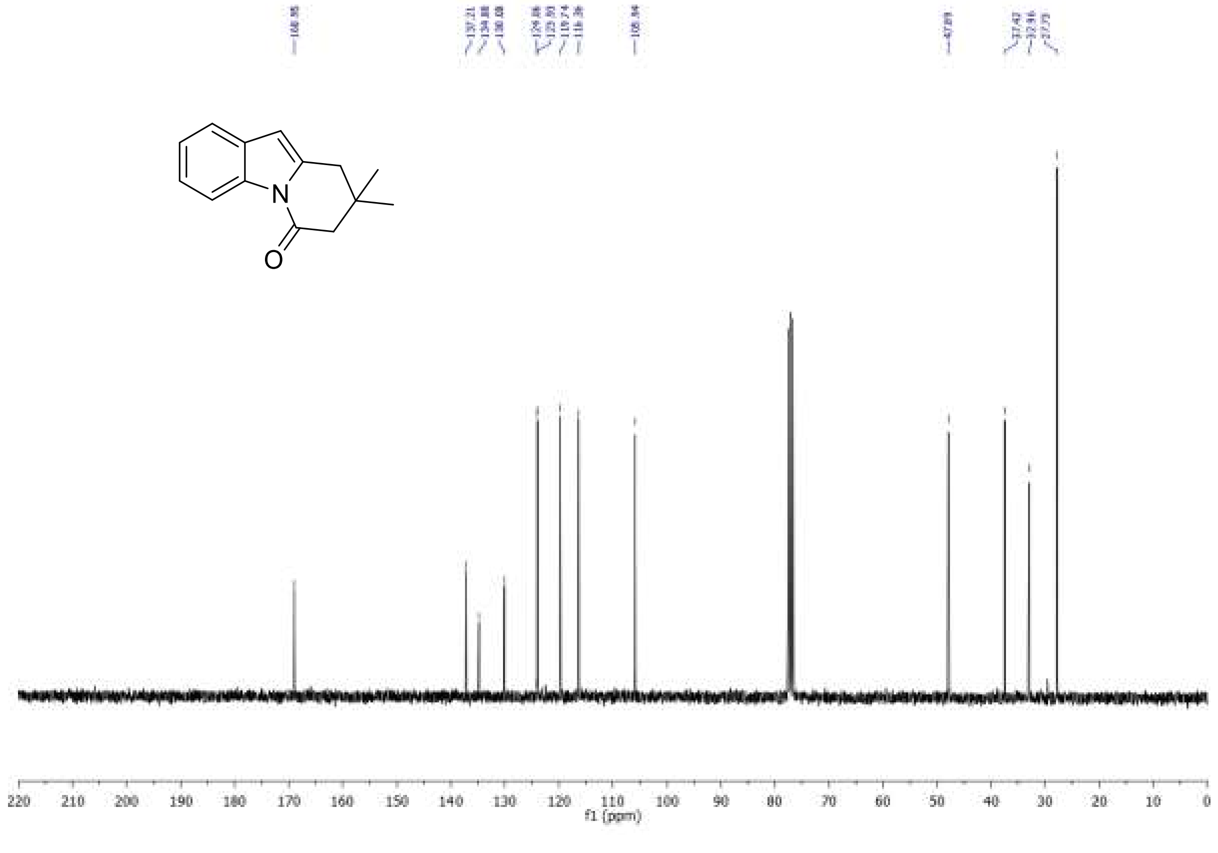

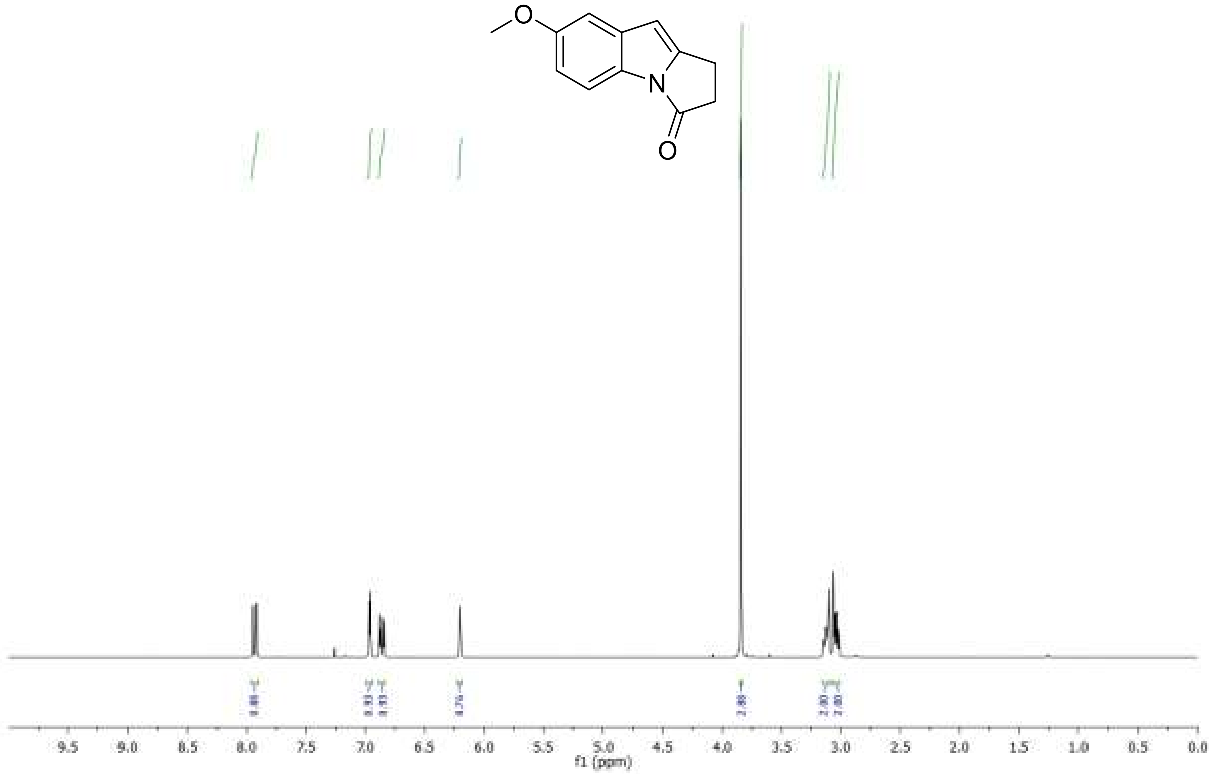
f1 (ppm)

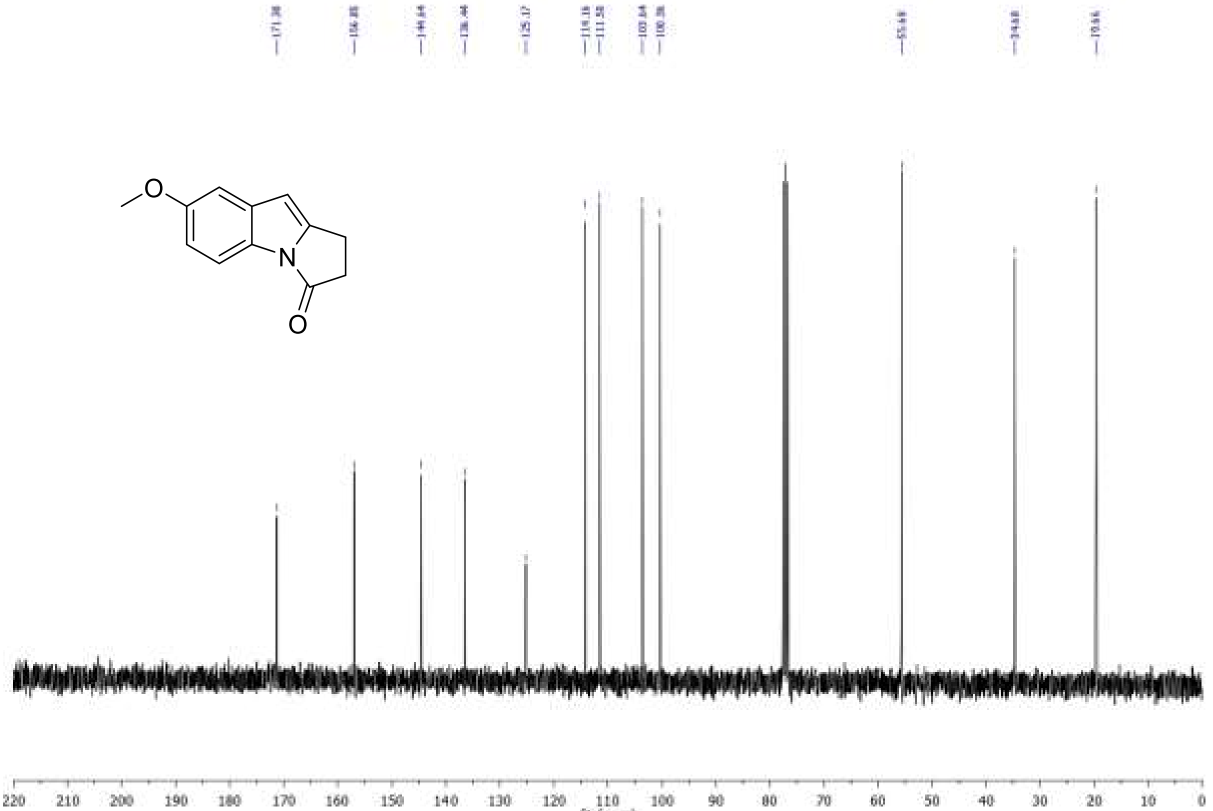
f1 (ppm)

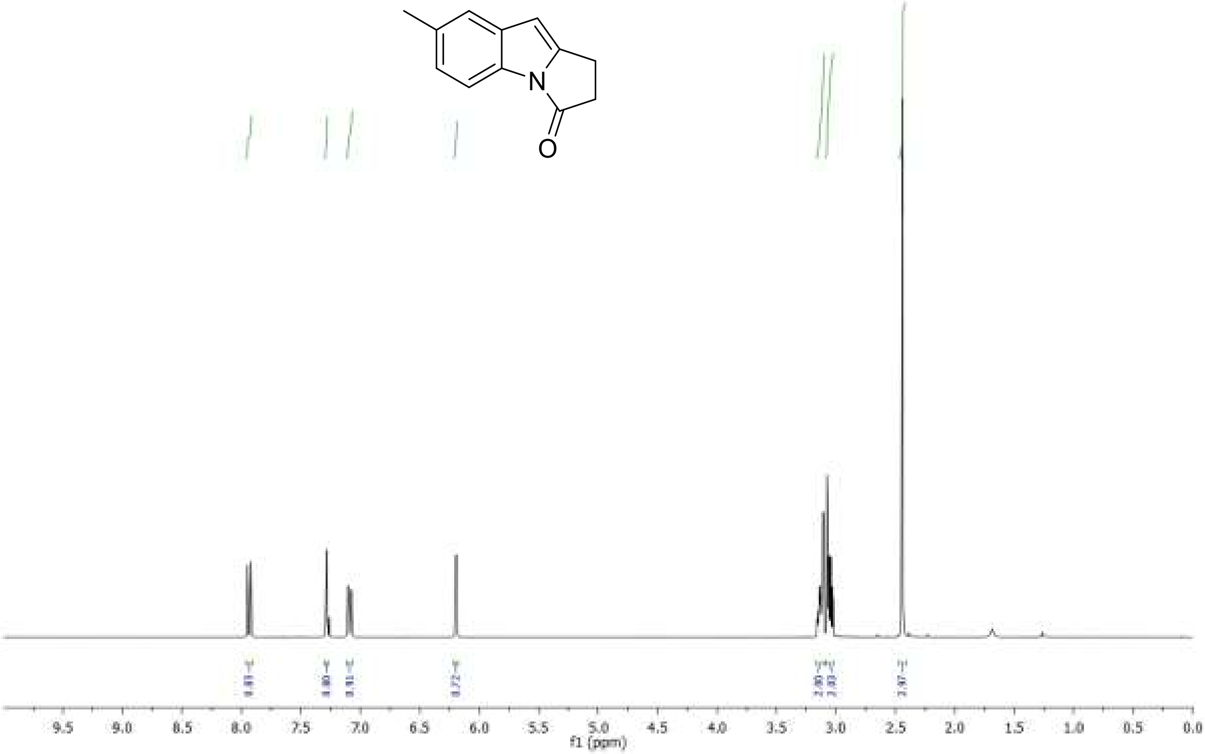
N
O
f1 (ppm)

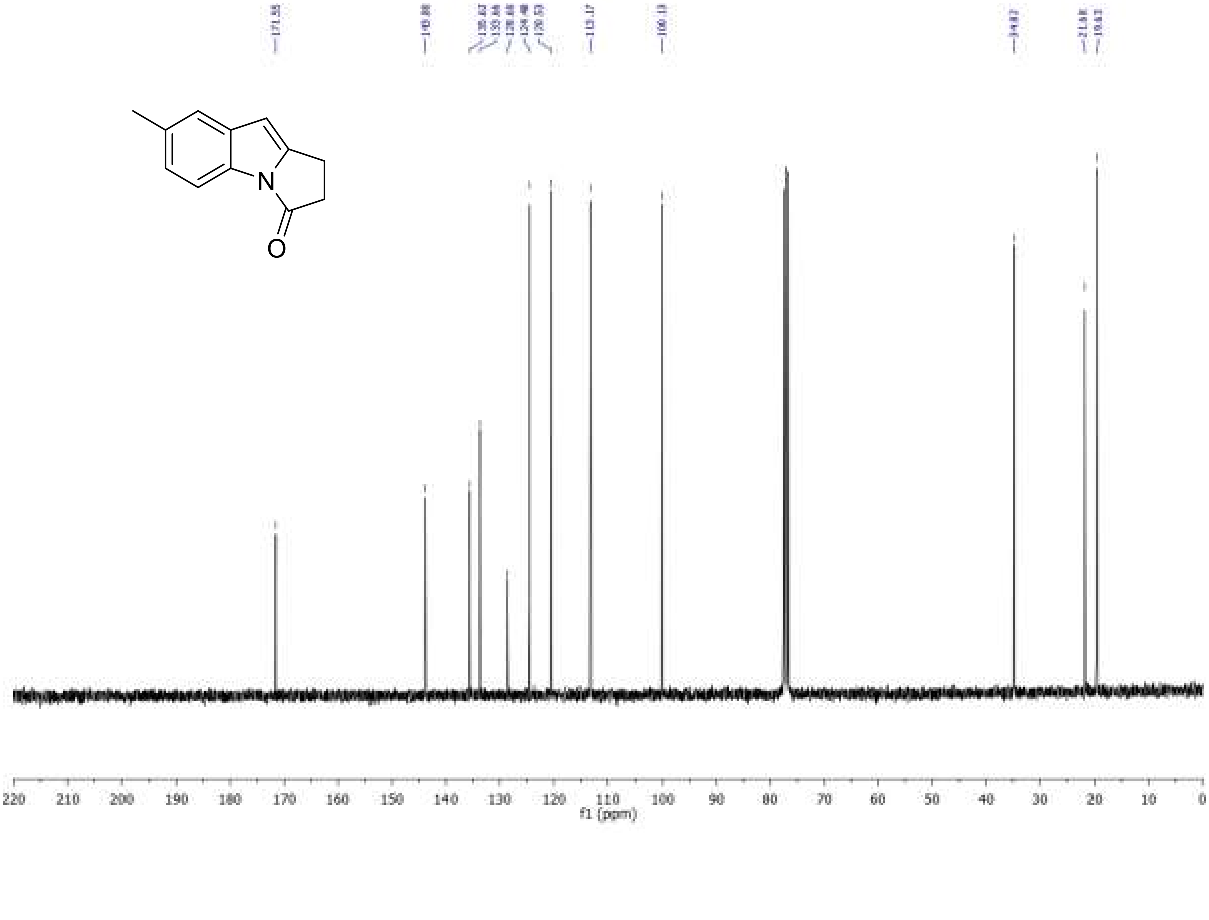
N
O
f1 (ppm)

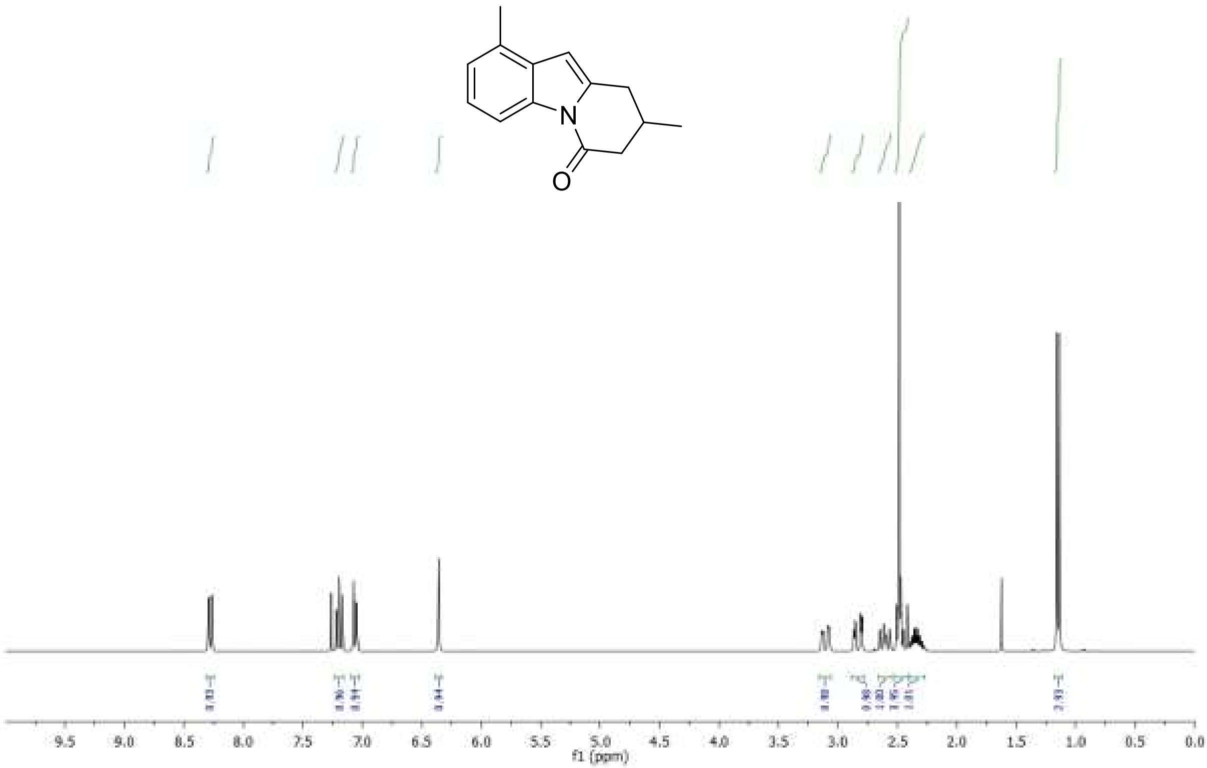
N
O
9.5 9.0 8.5 8.0 7.5 7.0 6.5 6.0 5.5 5.0 4.5 4.0 3.5 3.0 2.5 2.0 1.5 1.0 0.5 0.0
f1 (ppm)

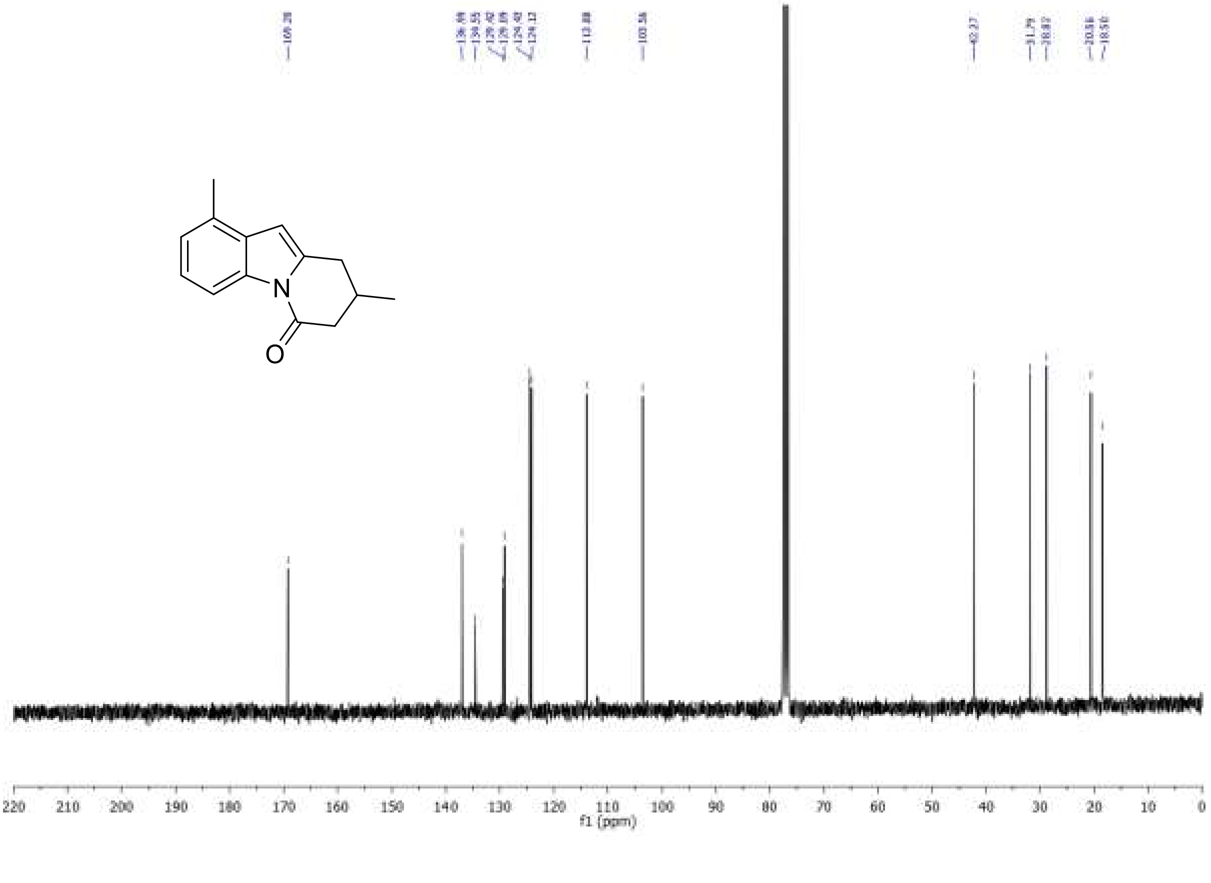
N
O
220 210 200 190 180 170 160 150 140 130 120 110 100 90 80 70 60 50 40 30 20 10 0
f1 (ppm)

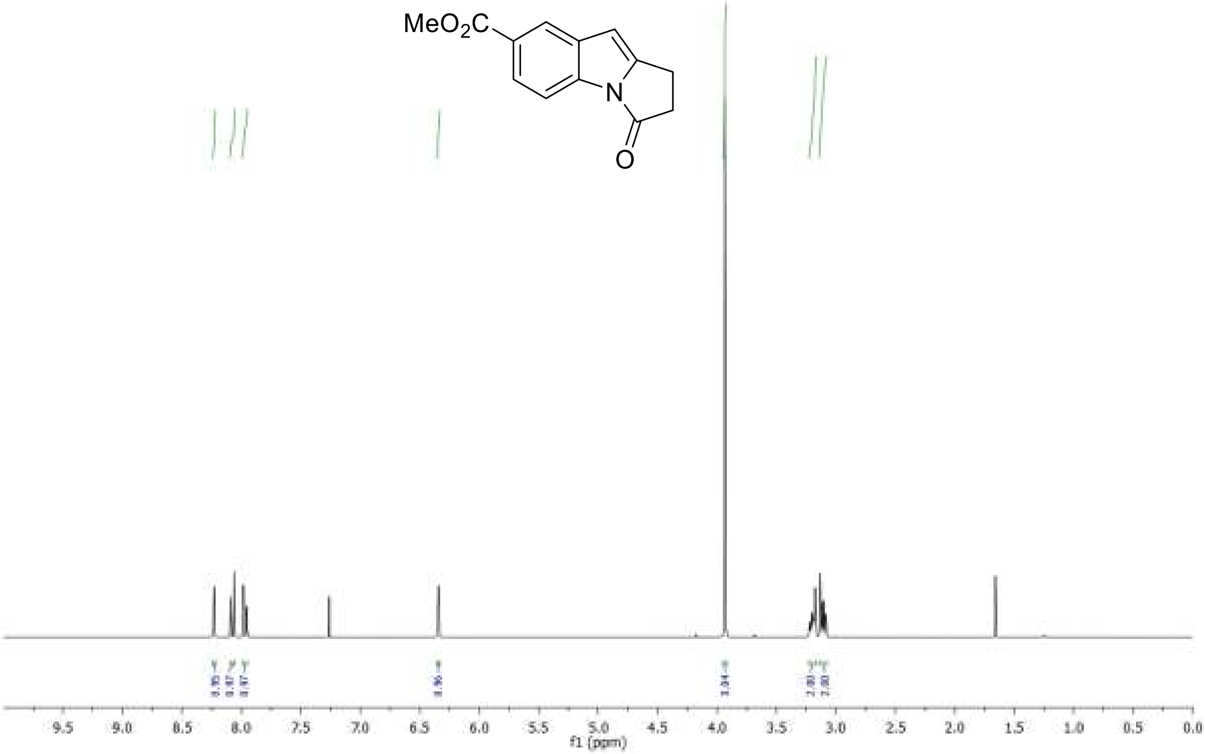
MeO2C
N
O
f1 (ppm)

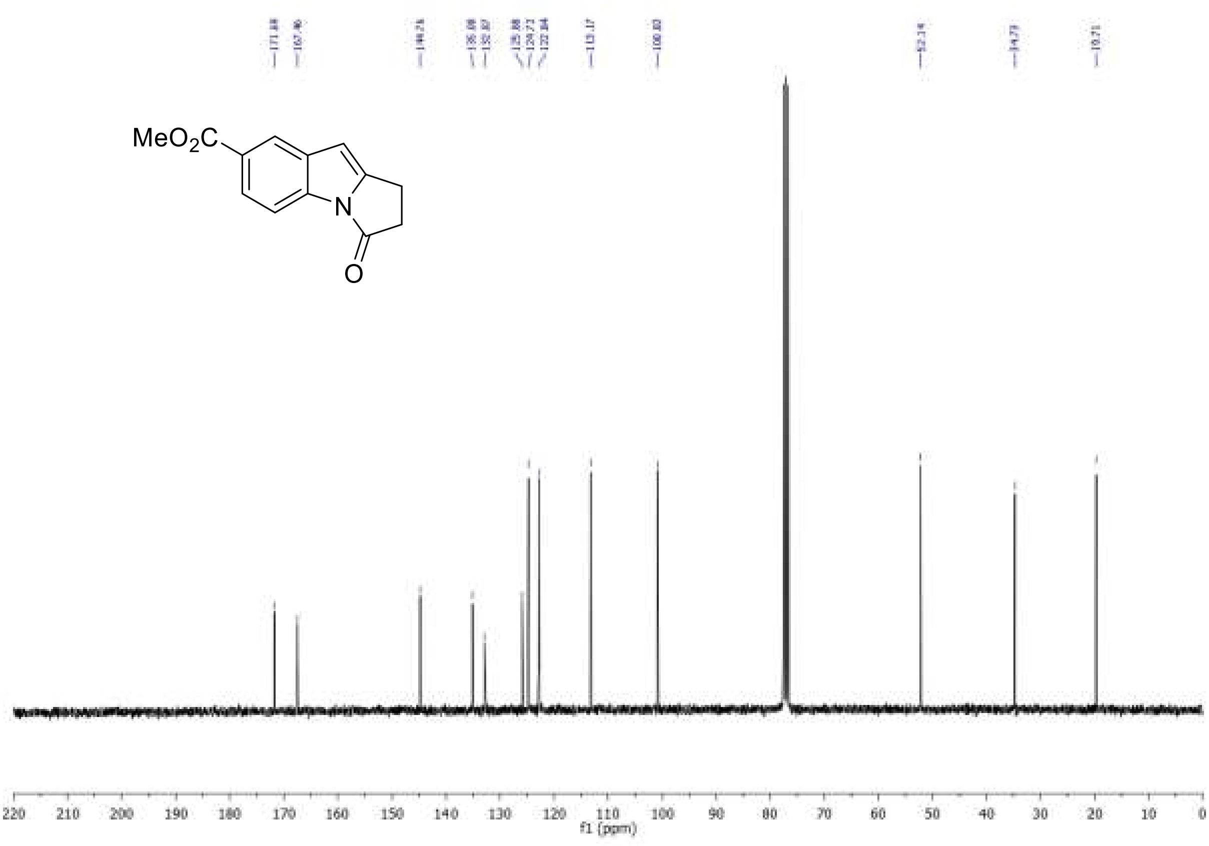
MeO2C
N
O
f1 (ppm)

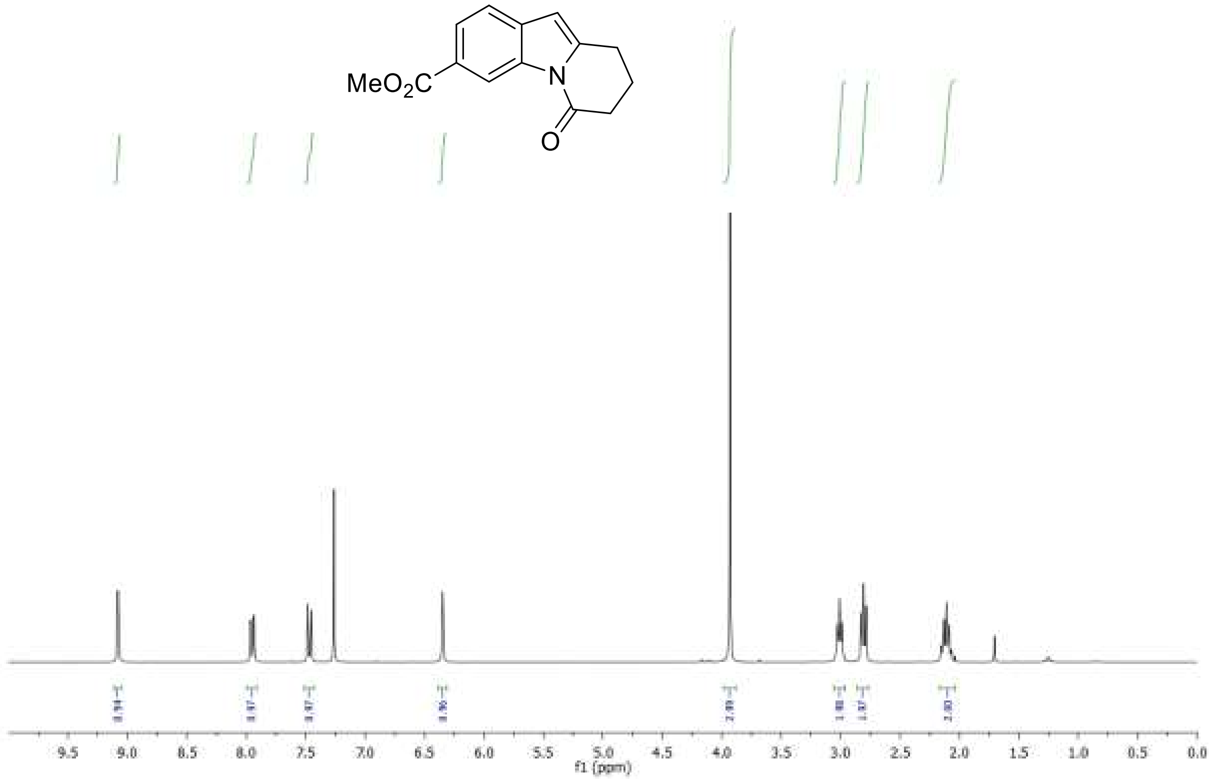
MeO2C
N
O
f1 (ppm)

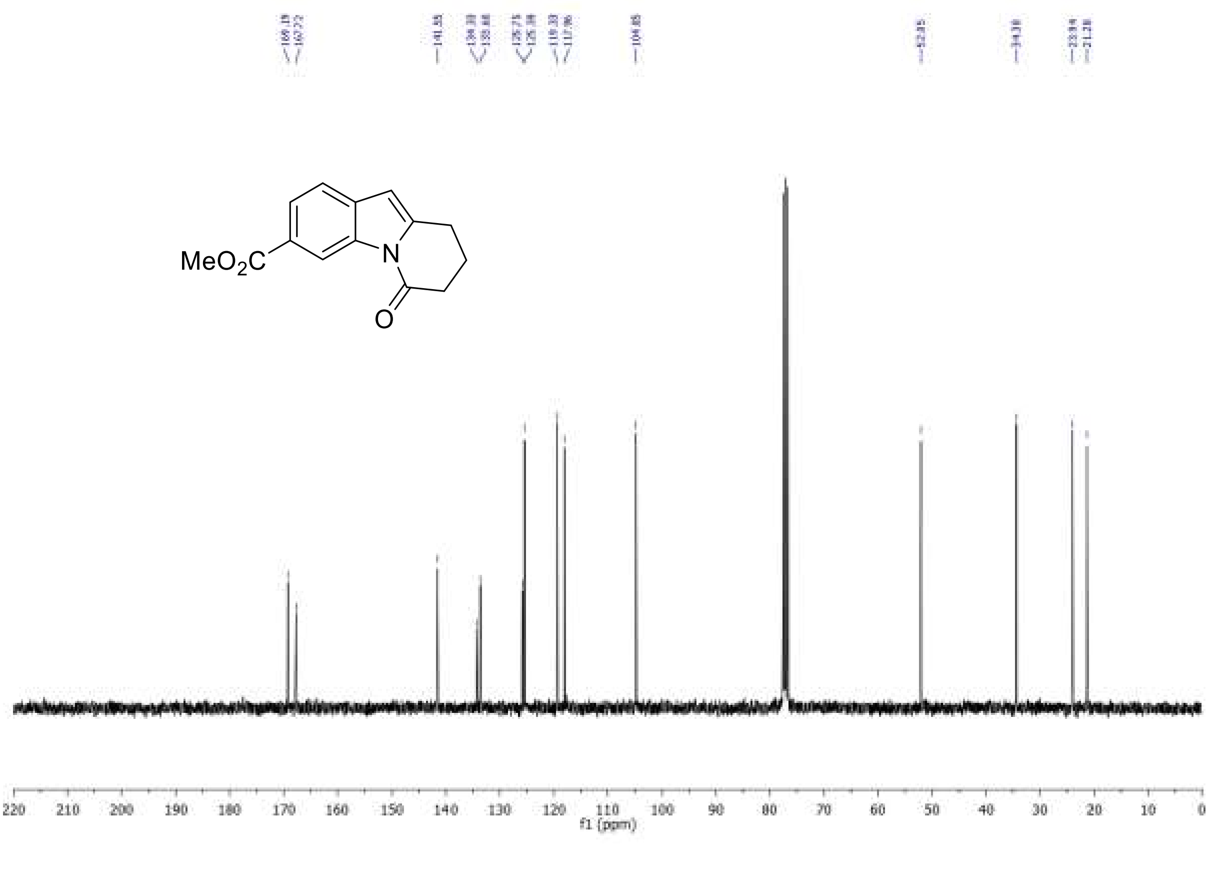
MeO2C
N
O
f1 (ppm)

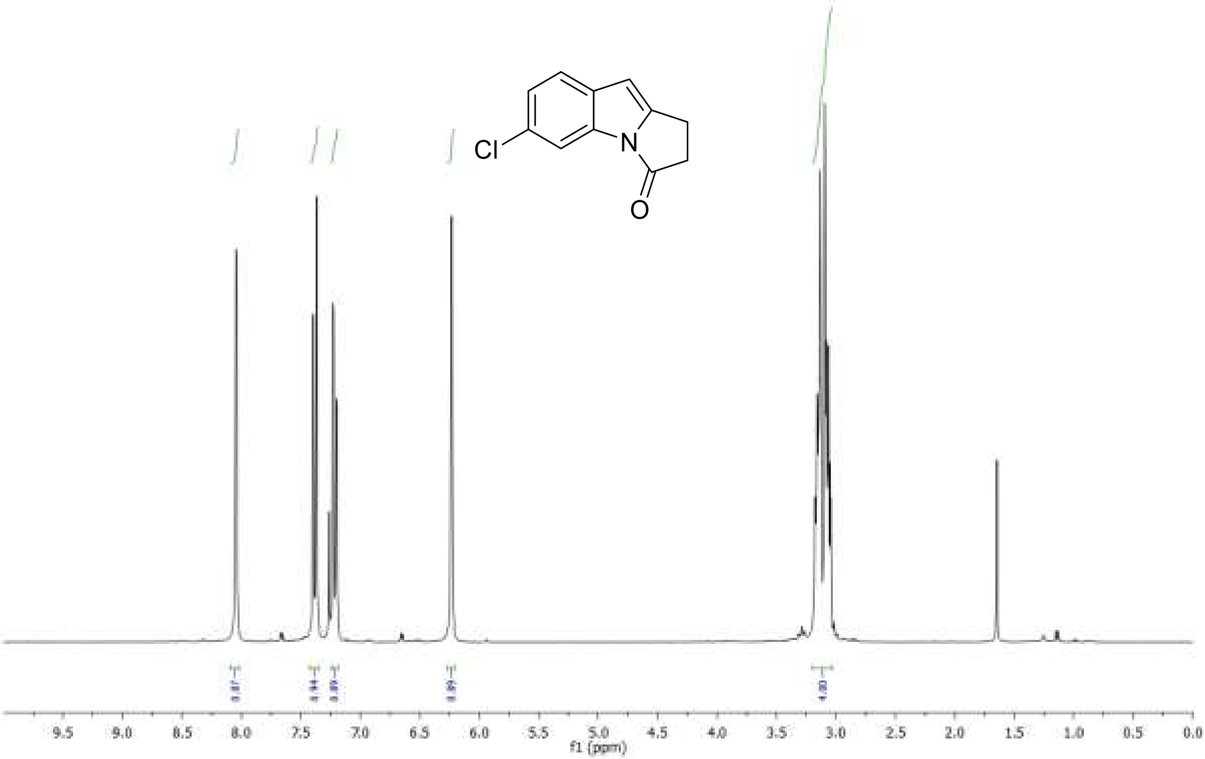

Cl
N
O
9.5 9.0 8.5 8.0 7.5 7.0 6.5 6.0 5.5 5.0 4.5 4.0 3.5 3.0 2.5 2.0 1.5 1.0 0.5 0.0
f1 (ppm)

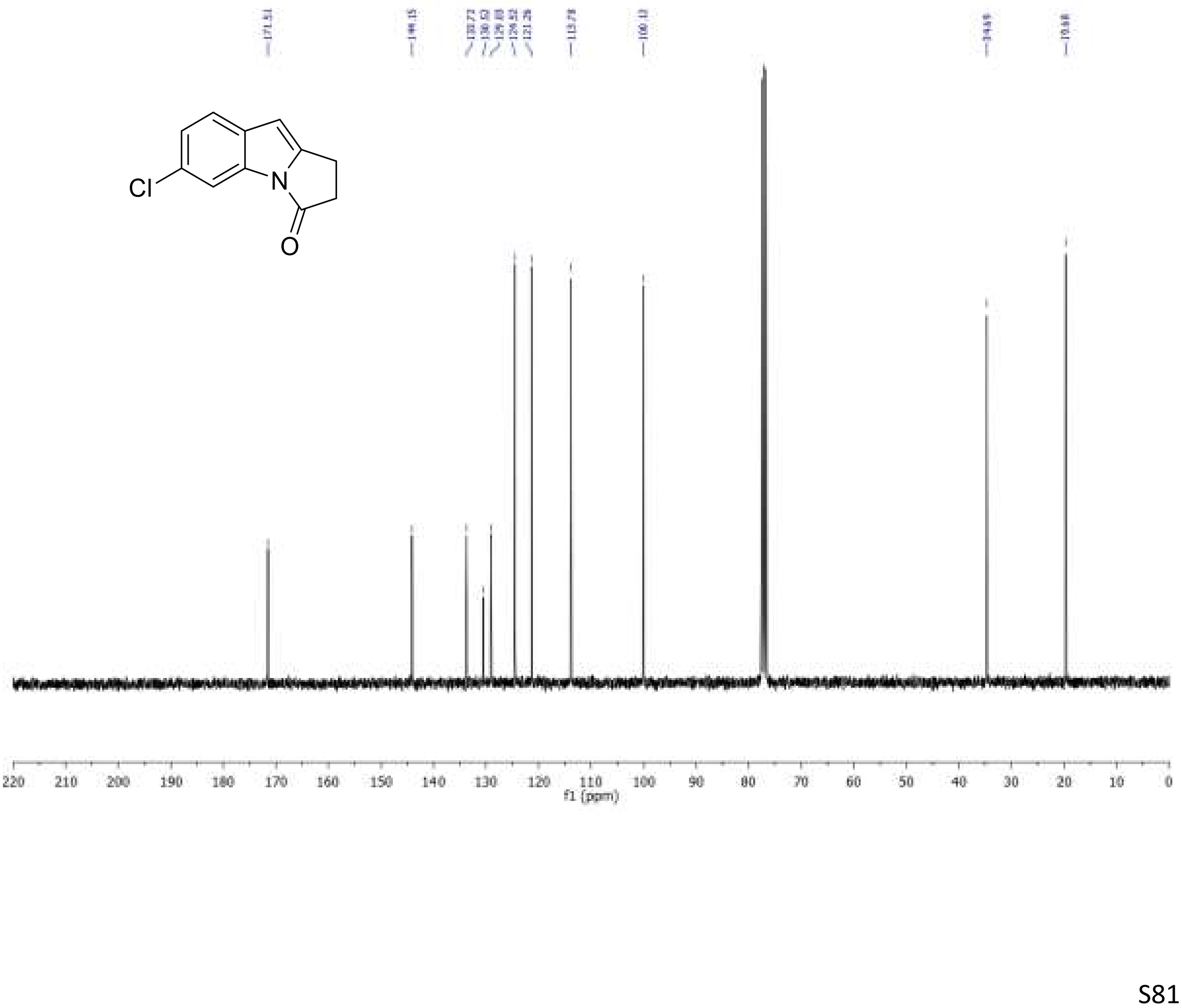

Cl
N
O
220 210 200 190 180 170 160 150 140 130 120 110 100 90 80 70 60 50 40 30 20 10 0
f1 (ppm)

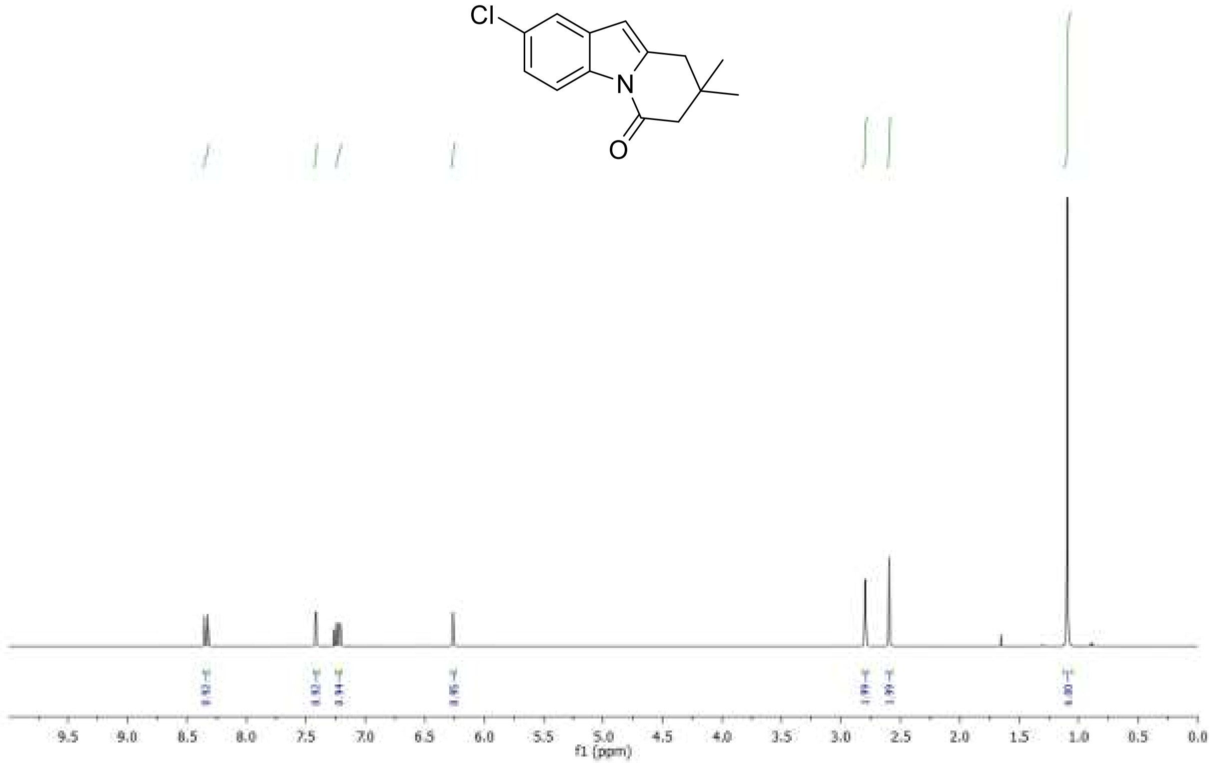
Cl
N
O
f1 (ppm)

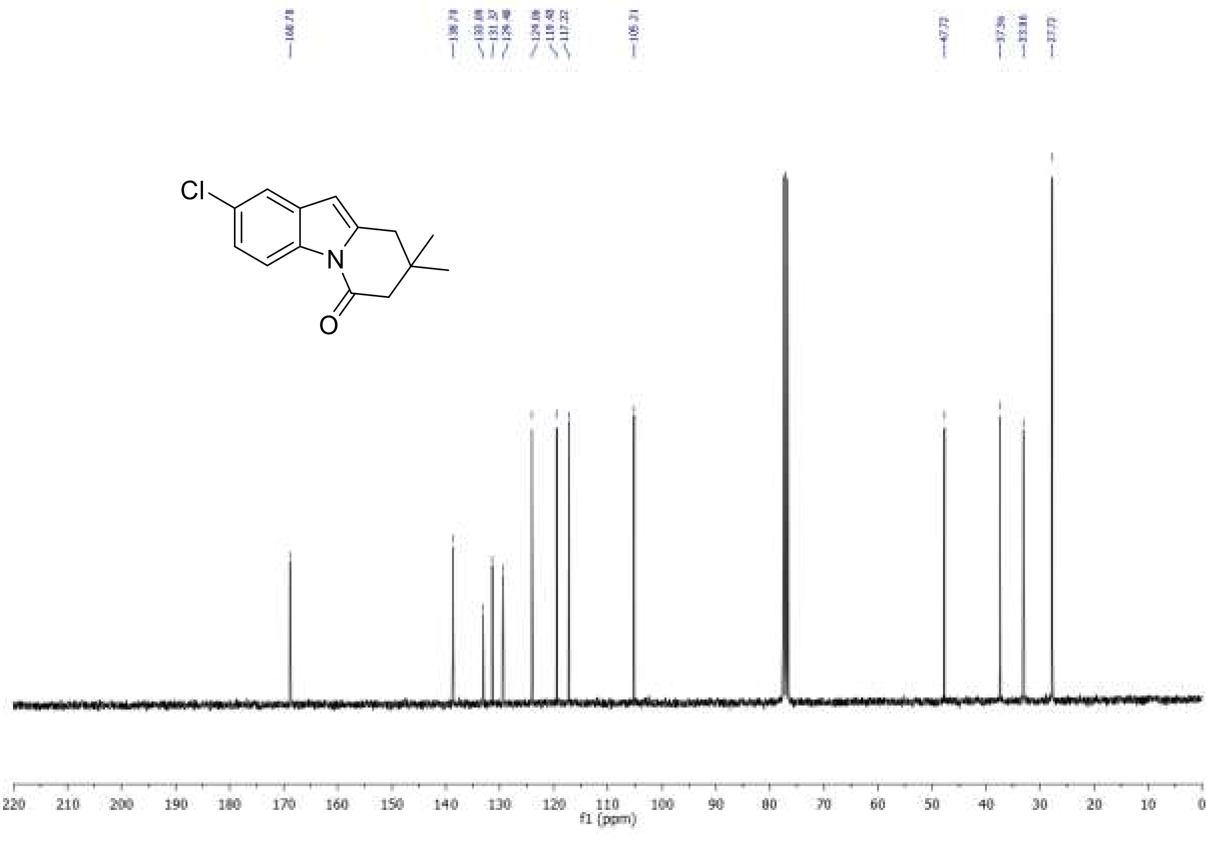
Cl
N
O
f1 (ppm)

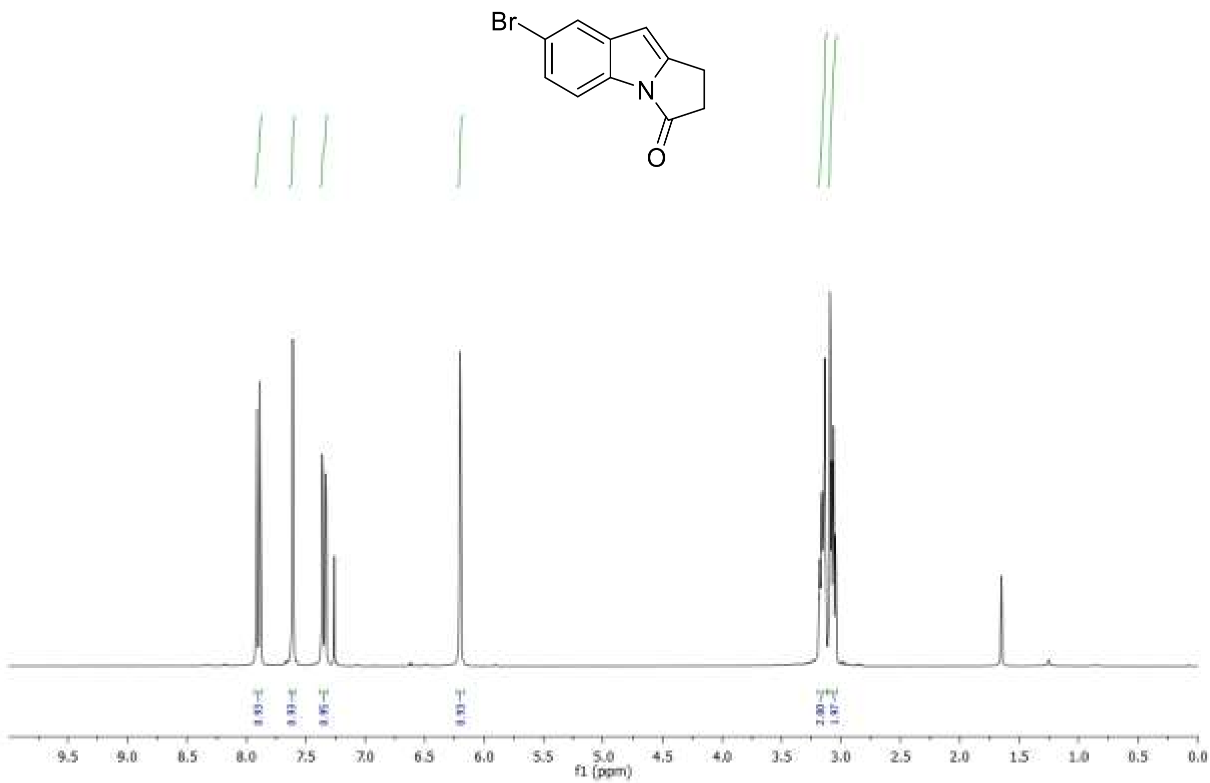

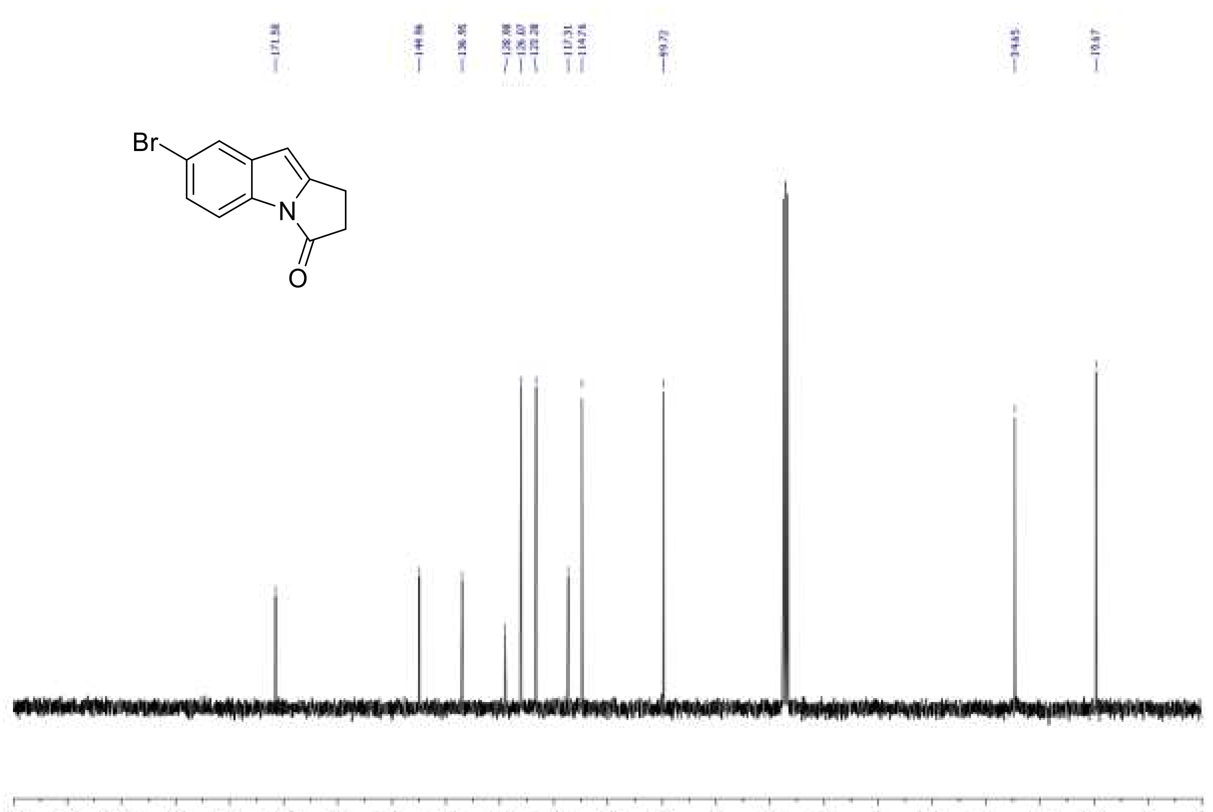

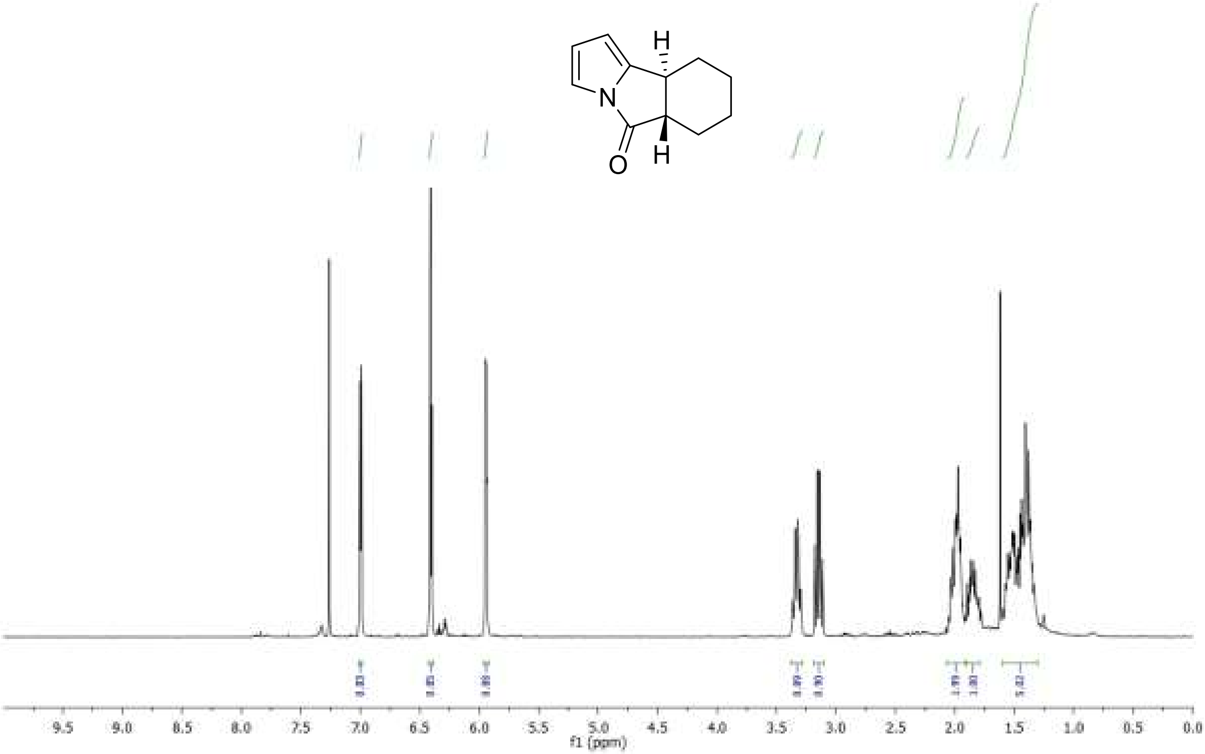
H
N
H
O
9.5 9.0 8.5 8.0 7.5 7.0 6.5 6.0 5.5 5.0 4.5 4.0 3.5 3.0 2.5 2.0 1.5 1.0 0.5 0.0
f1 (ppm)

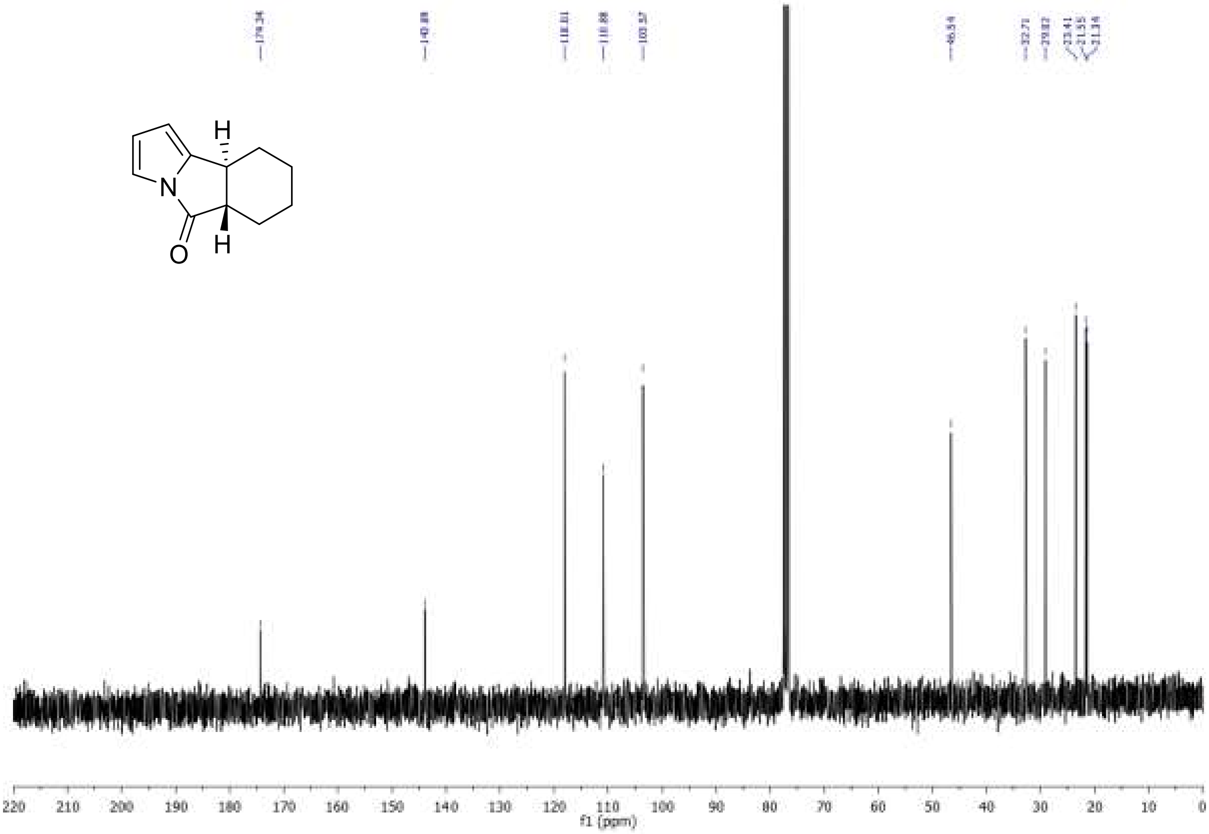
H
N
H
O
220 210 200 190 180 170 160 150 140 130 120 110 100 90 80 70 60 50 40 30 20 10 0
f1 (ppm)

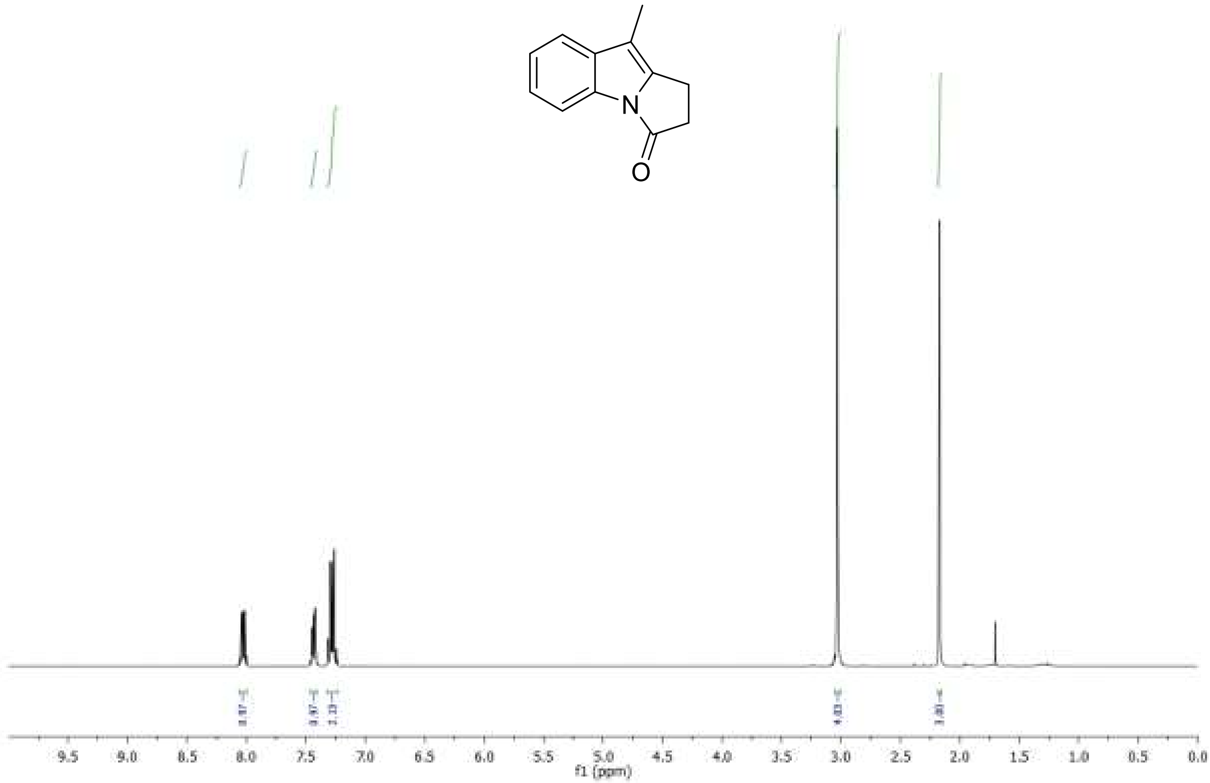

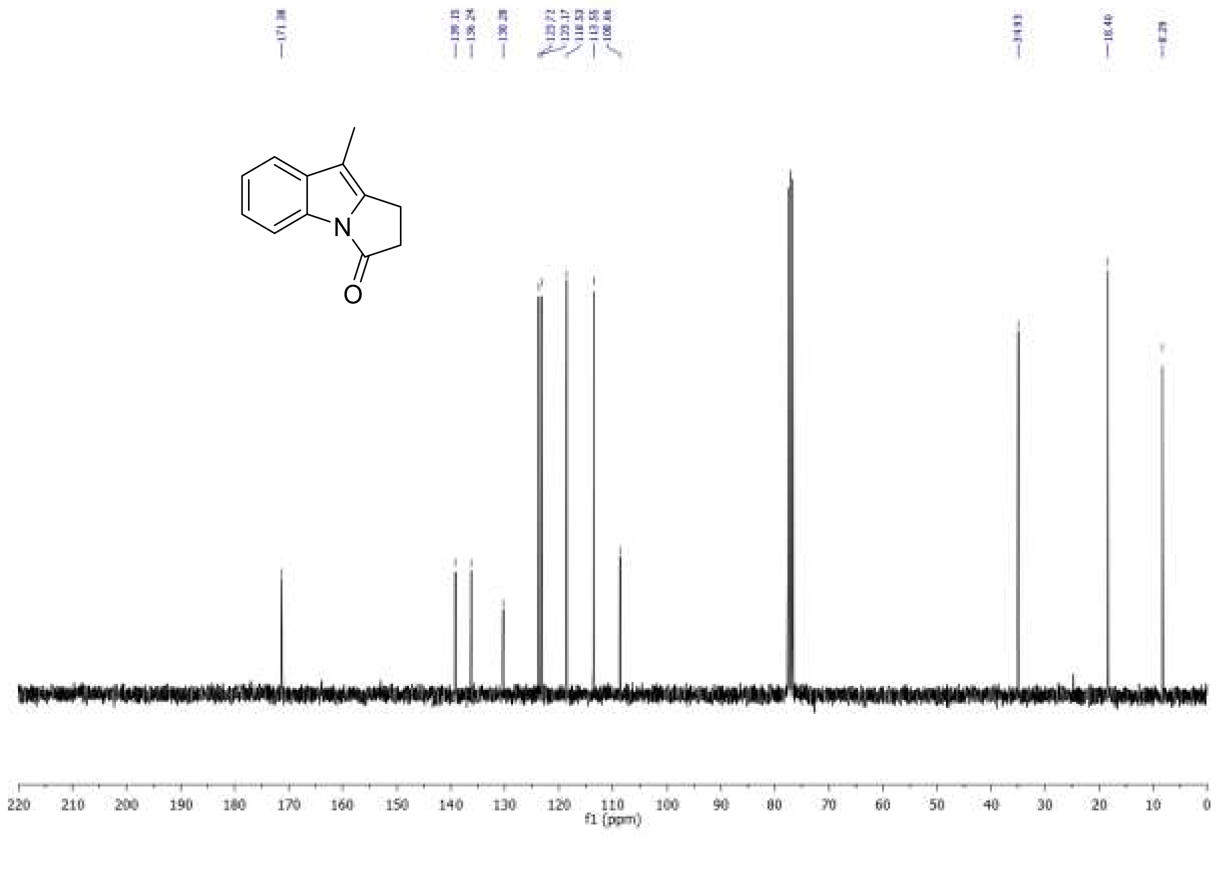

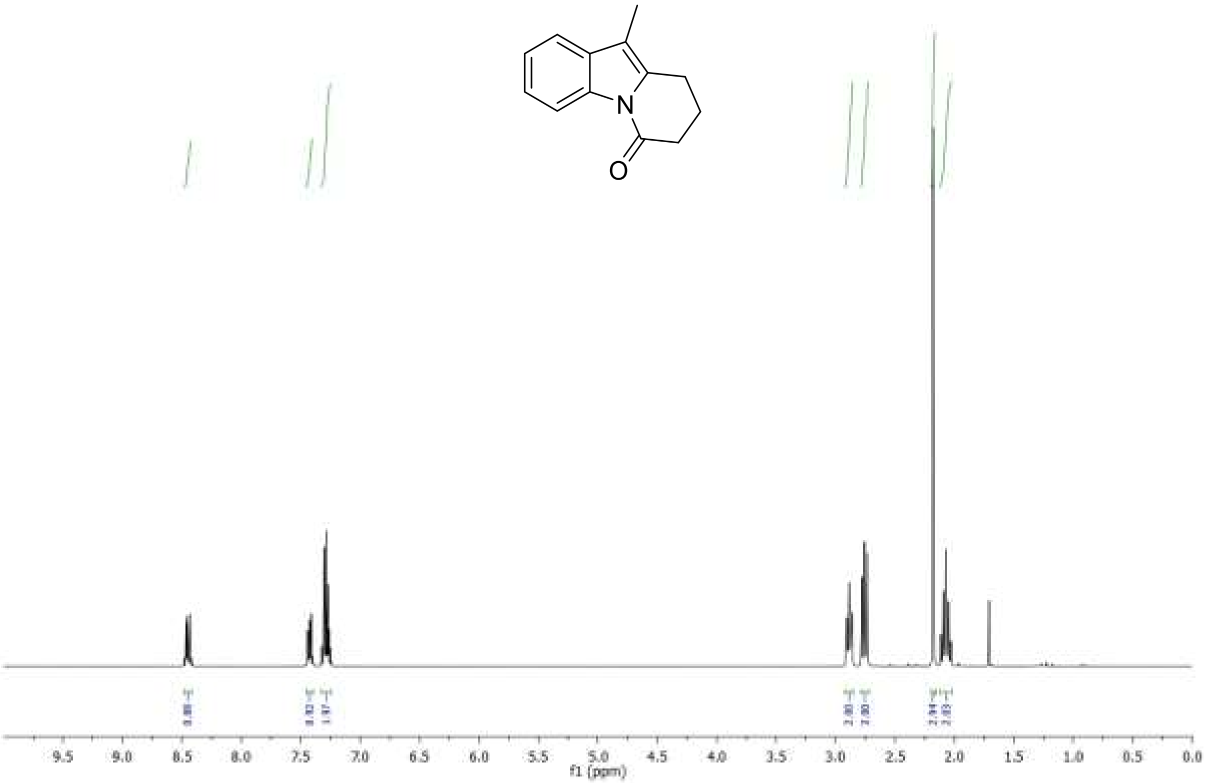
N
O
f1 (ppm)

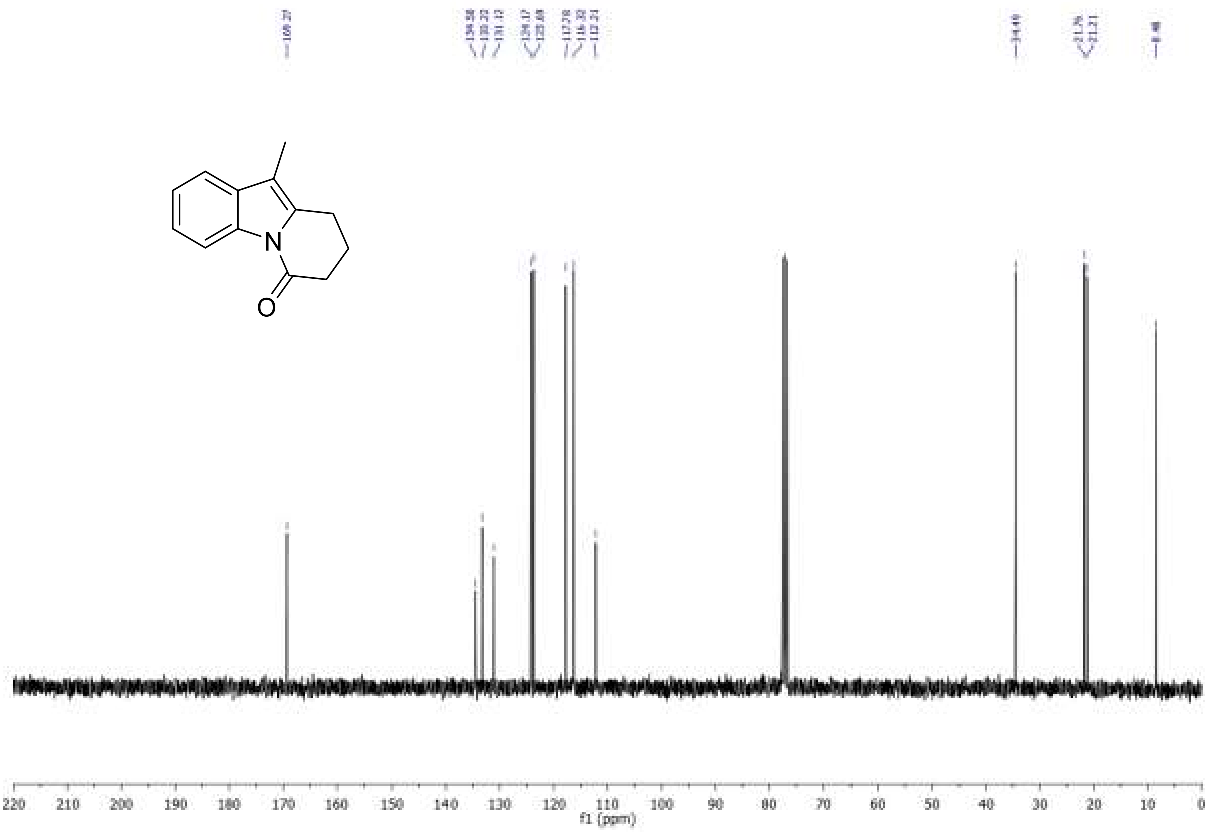
N
O
f1 (ppm)

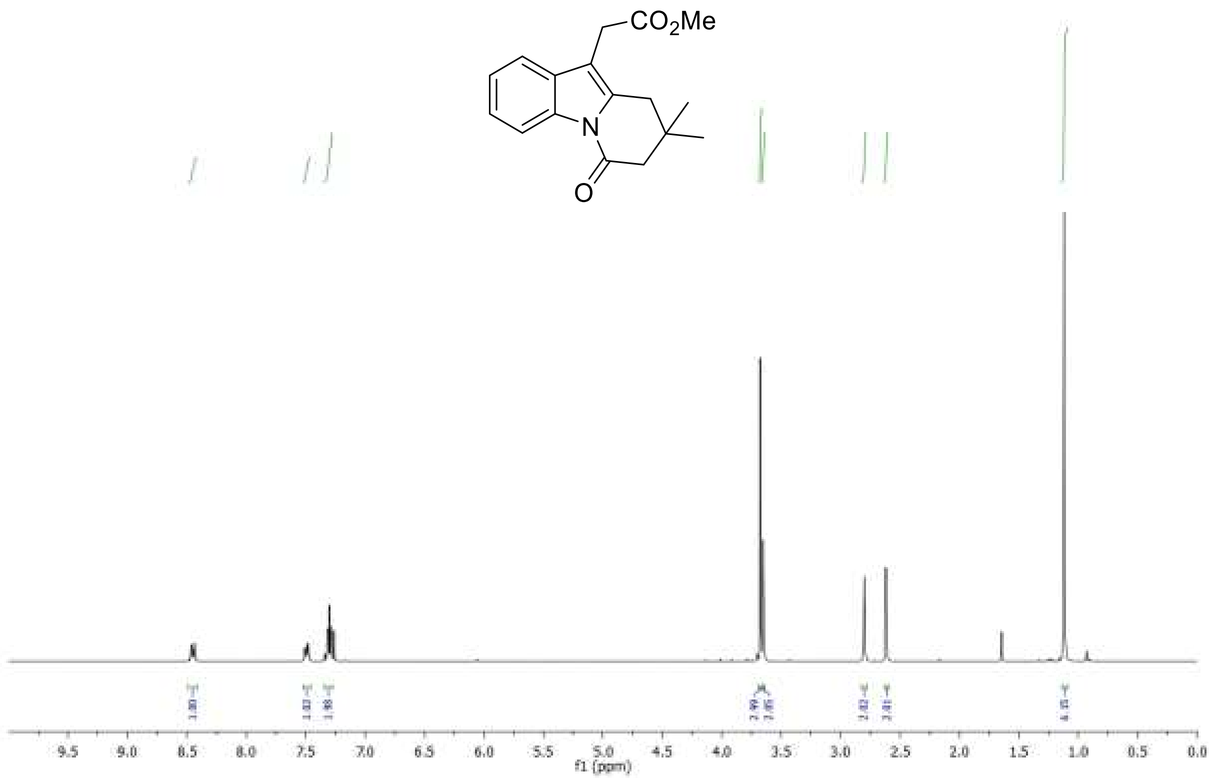
CO2Me
N
O
f1 (ppm)

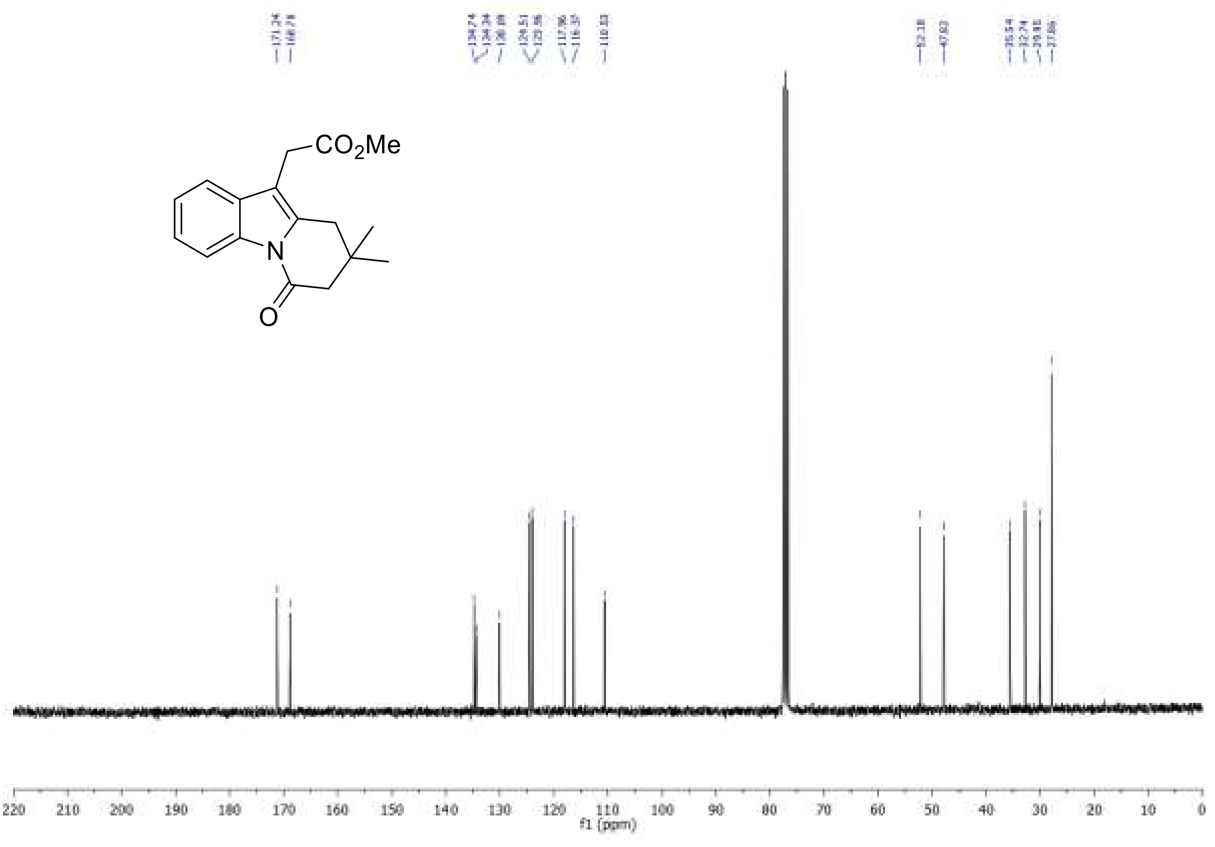
CO2Me
N
O
f1 (ppm)

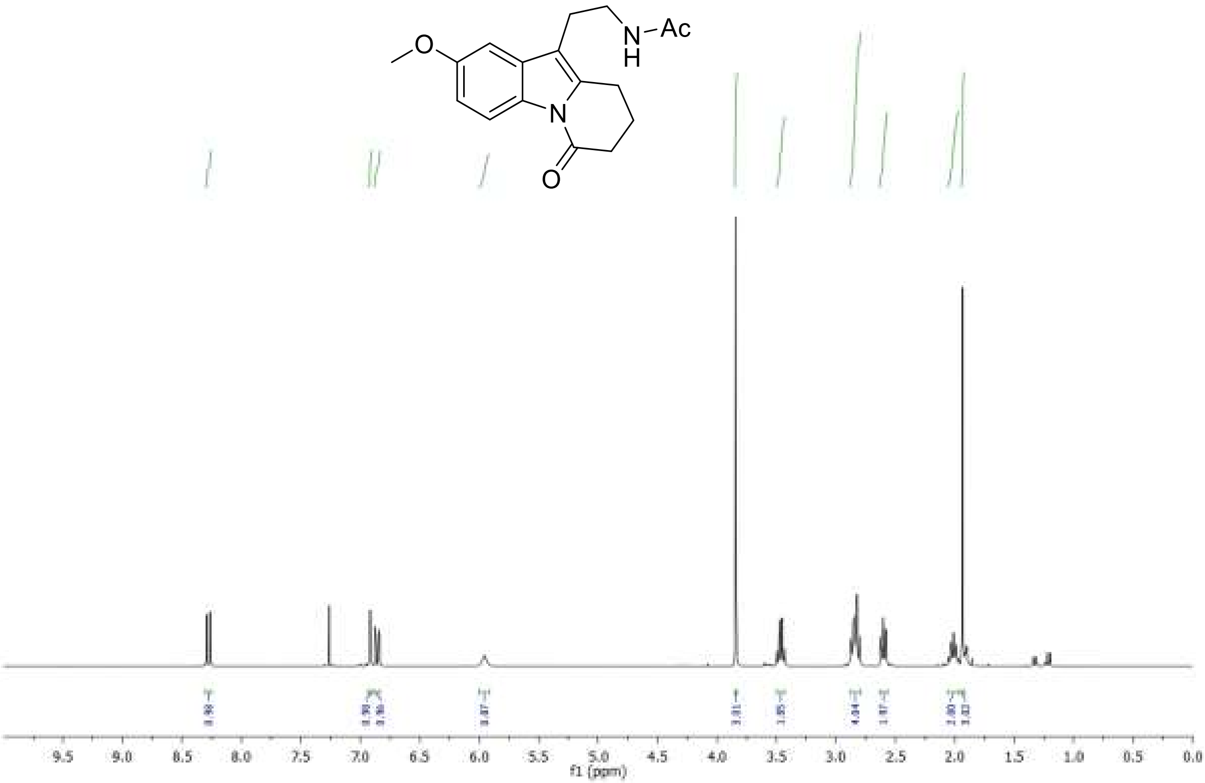

O
N
N-Ac
H
O
9.5 9.0 8.5 8.0 7.5 7.0 6.5 6.0 5.5 5.0 4.5 4.0 3.5 3.0 2.5 2.0 1.5 1.0 0.5 0.0
f1 (ppm)

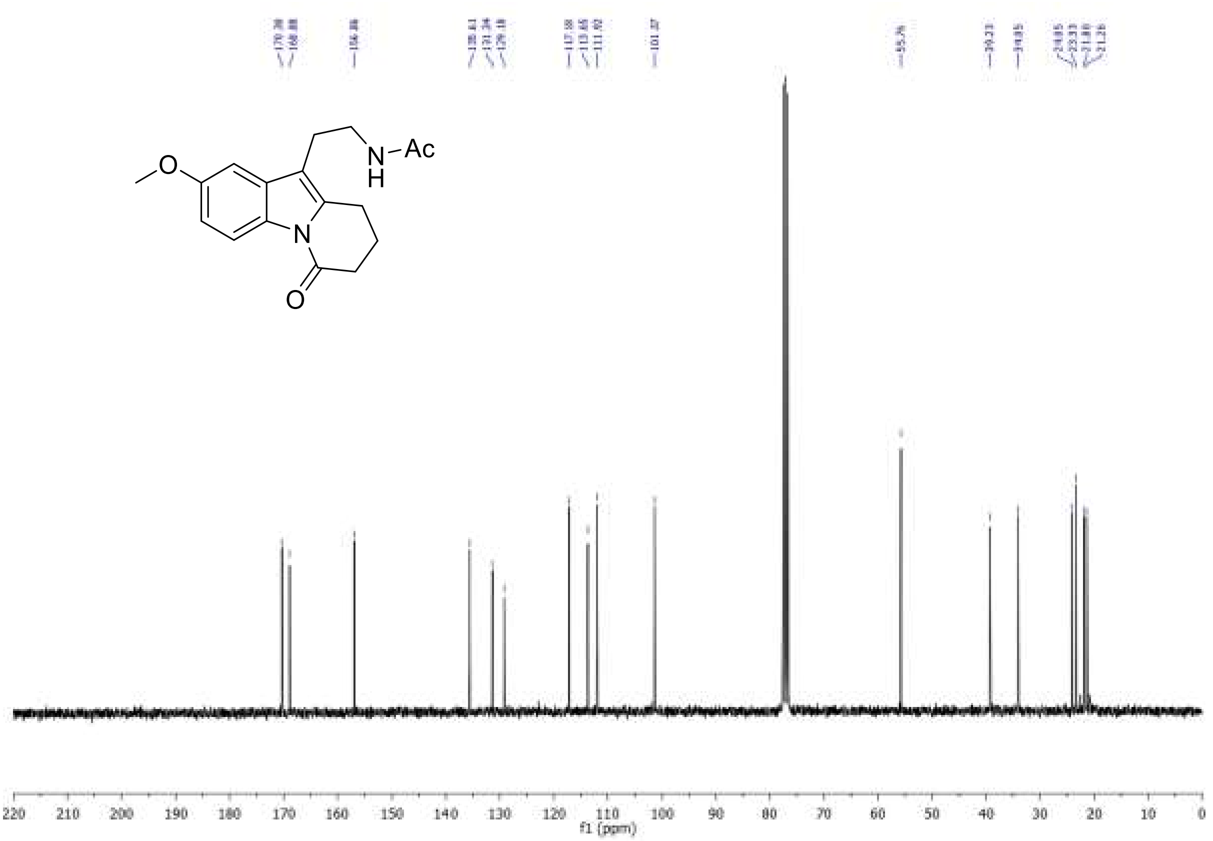

O
N
N-Ac
H
O
220 210 200 190 180 170 160 150 140 130 120 110 100 90 80 70 60 50 40 30 20 10 0
f1 (ppm)

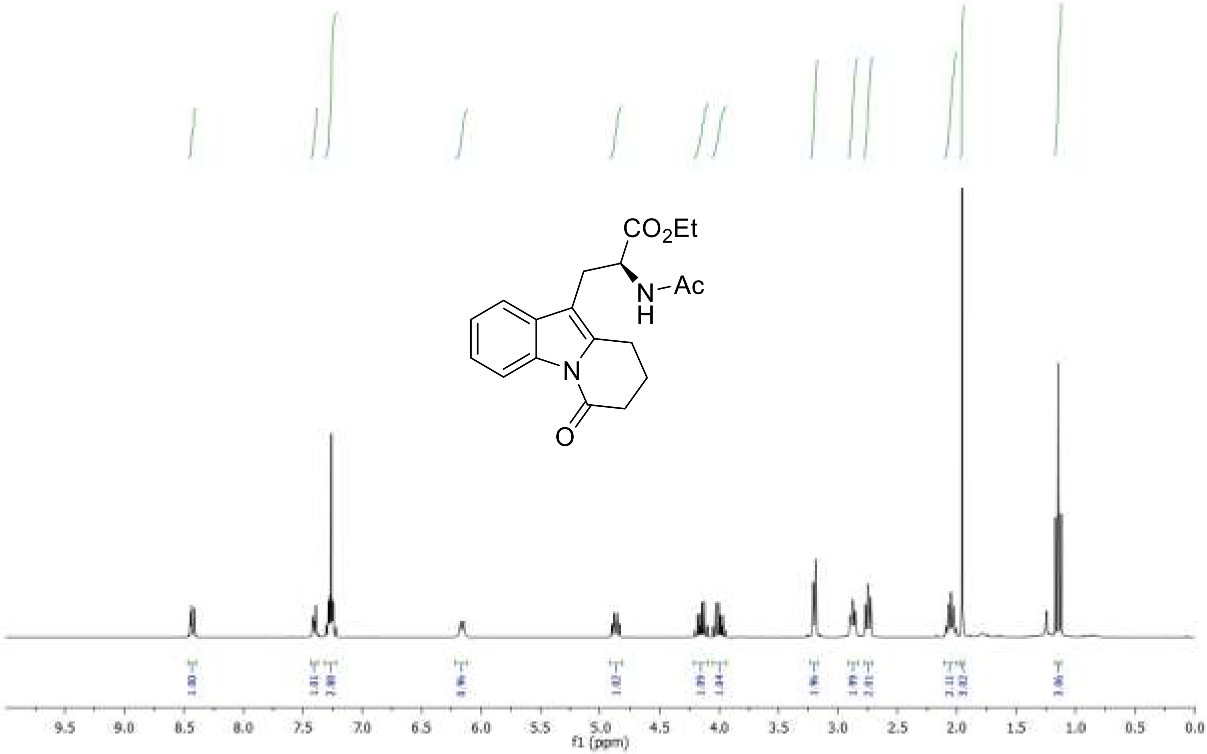
CO2Et
N-Ac
H
N
O
f1 (ppm)

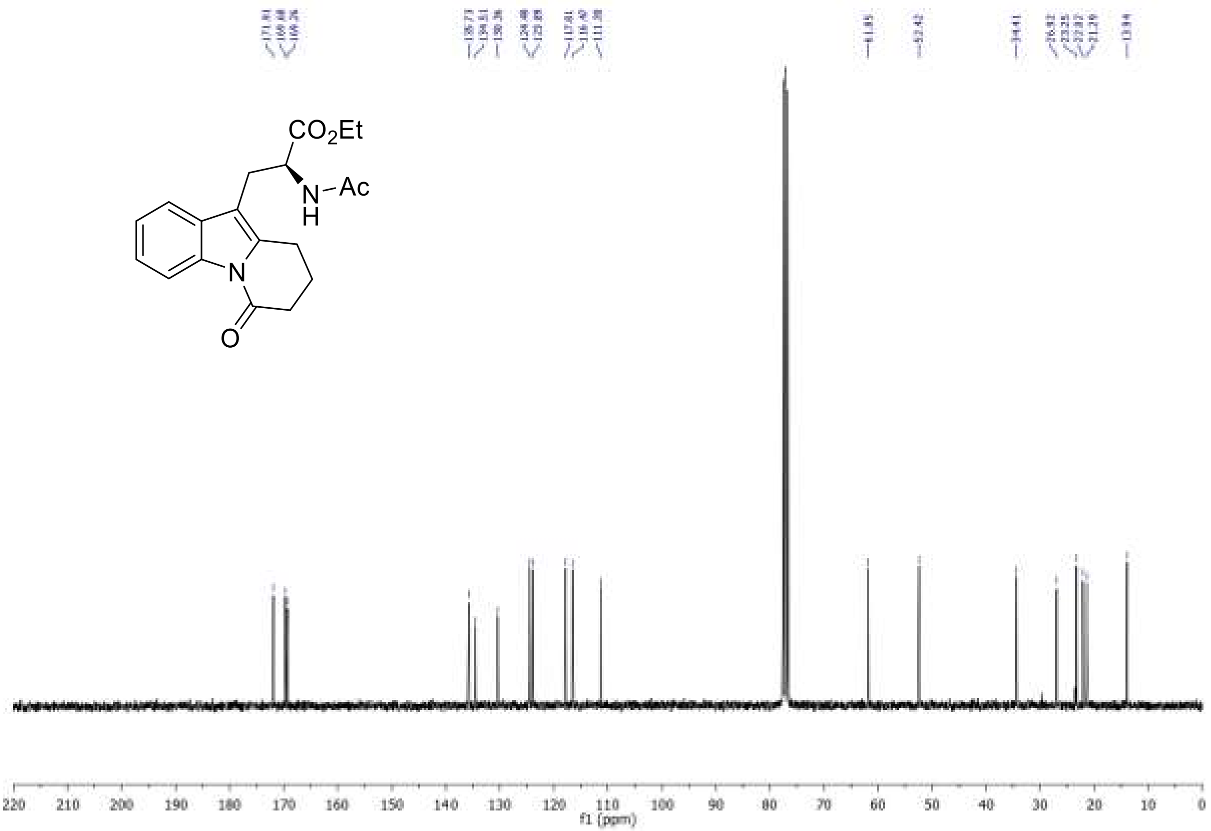
CO2Et
N-Ac
H
N
O
f1 (ppm)

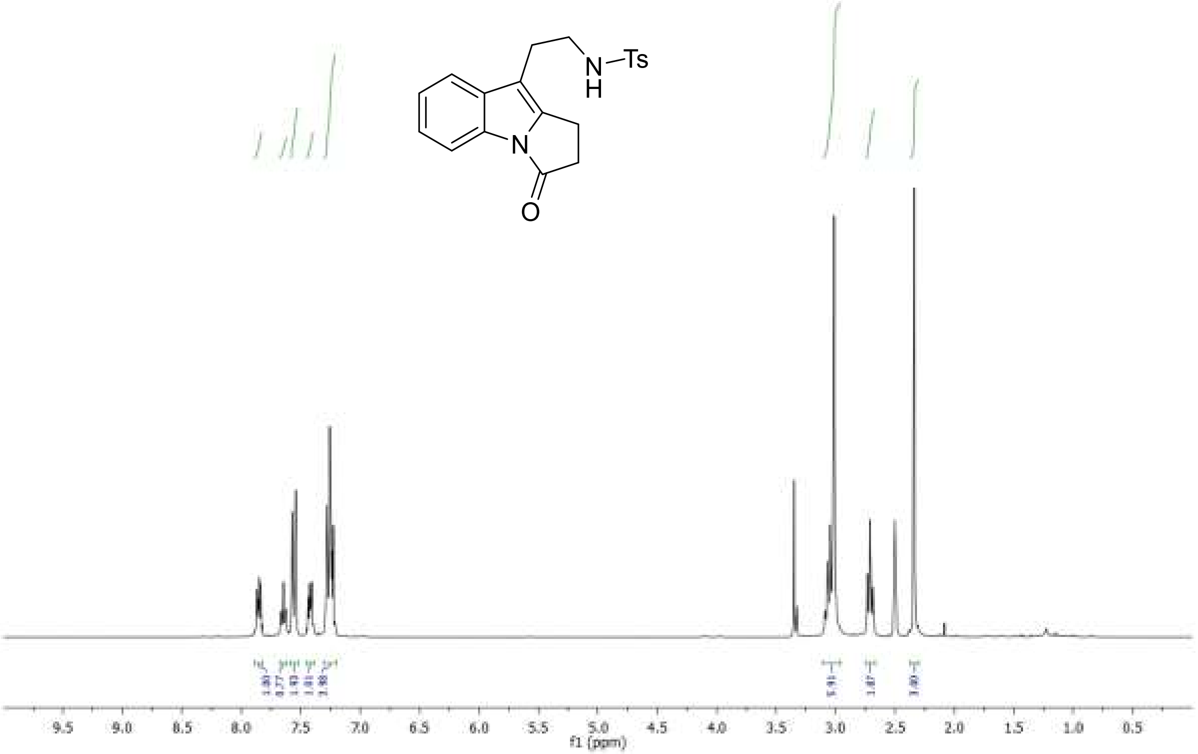
N–Ts
H
N
O
9.5 9.0 8.5 8.0 7.5 7.0 6.5 6.0 5.5 5.0 4.5 4.0 3.5 3.0 2.5 2.0 1.5 1.0 0.5
f1 (ppm)

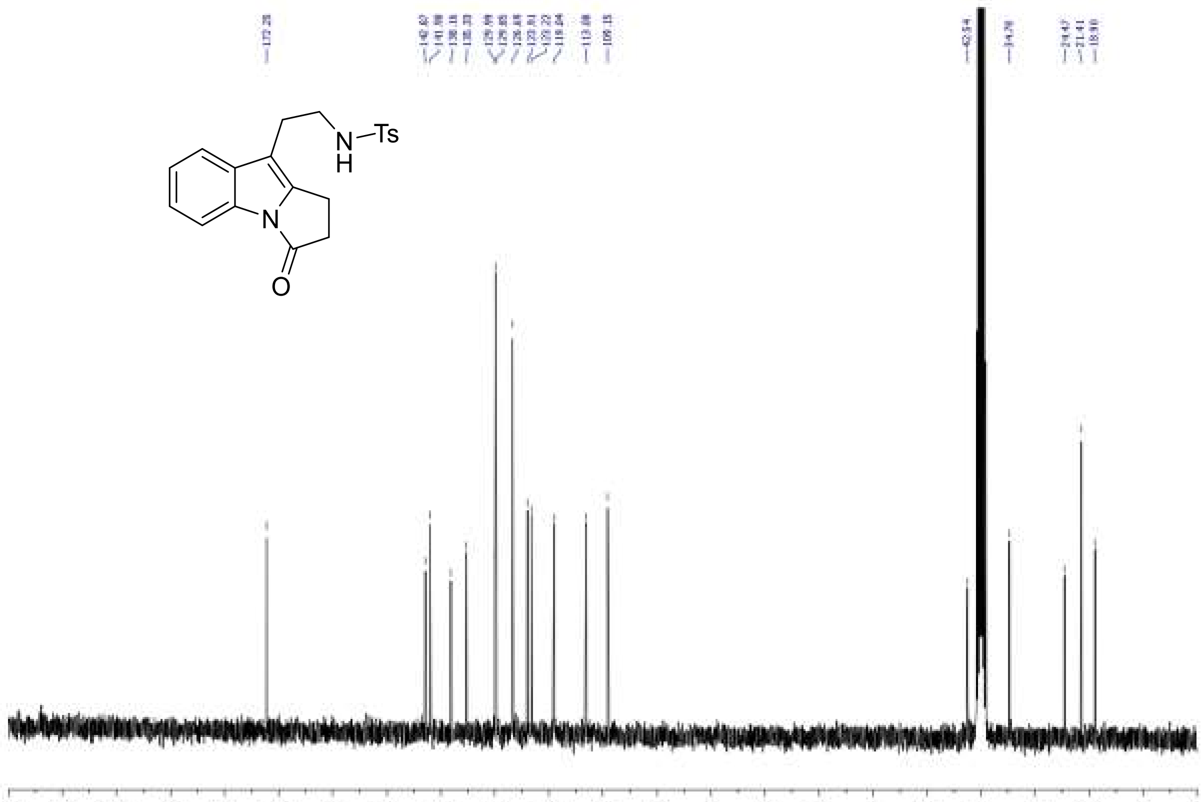
N–Ts
H
N
O
220 210 200 190 180 170 160 150 140 130 120 110 100 90 80 70 60 50 40 30 20 10 0
f1 (ppm)

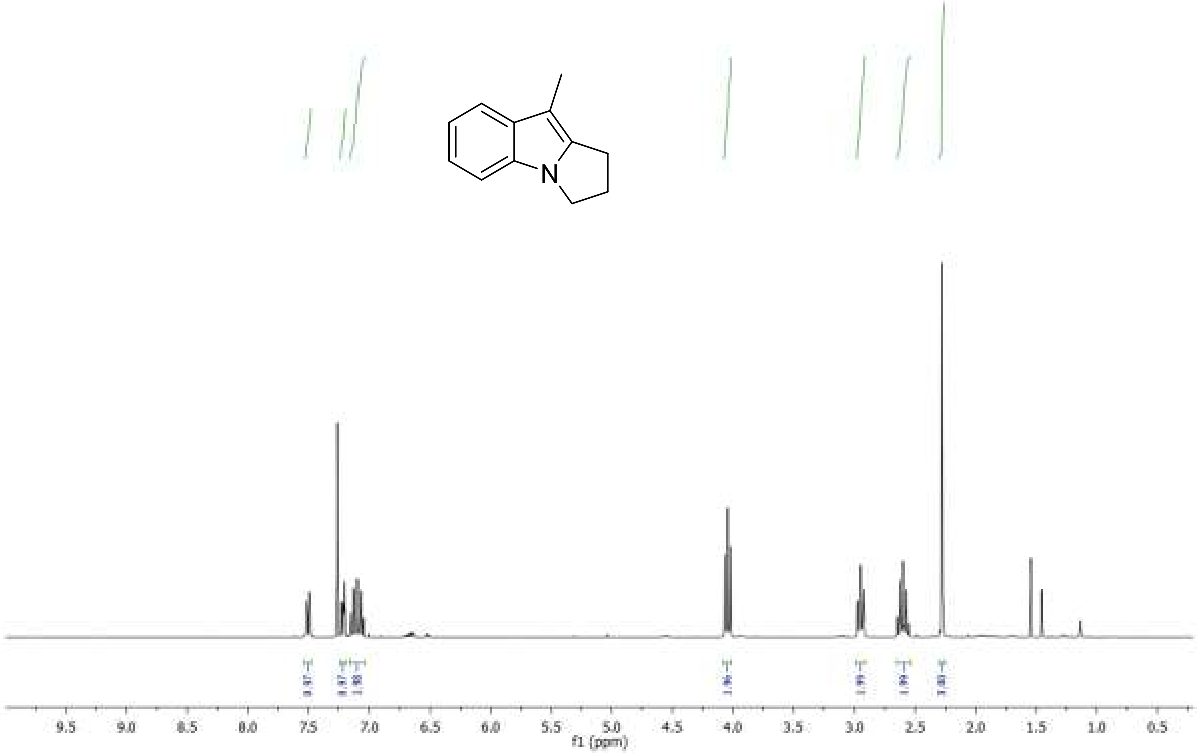
N
f1 (ppm)

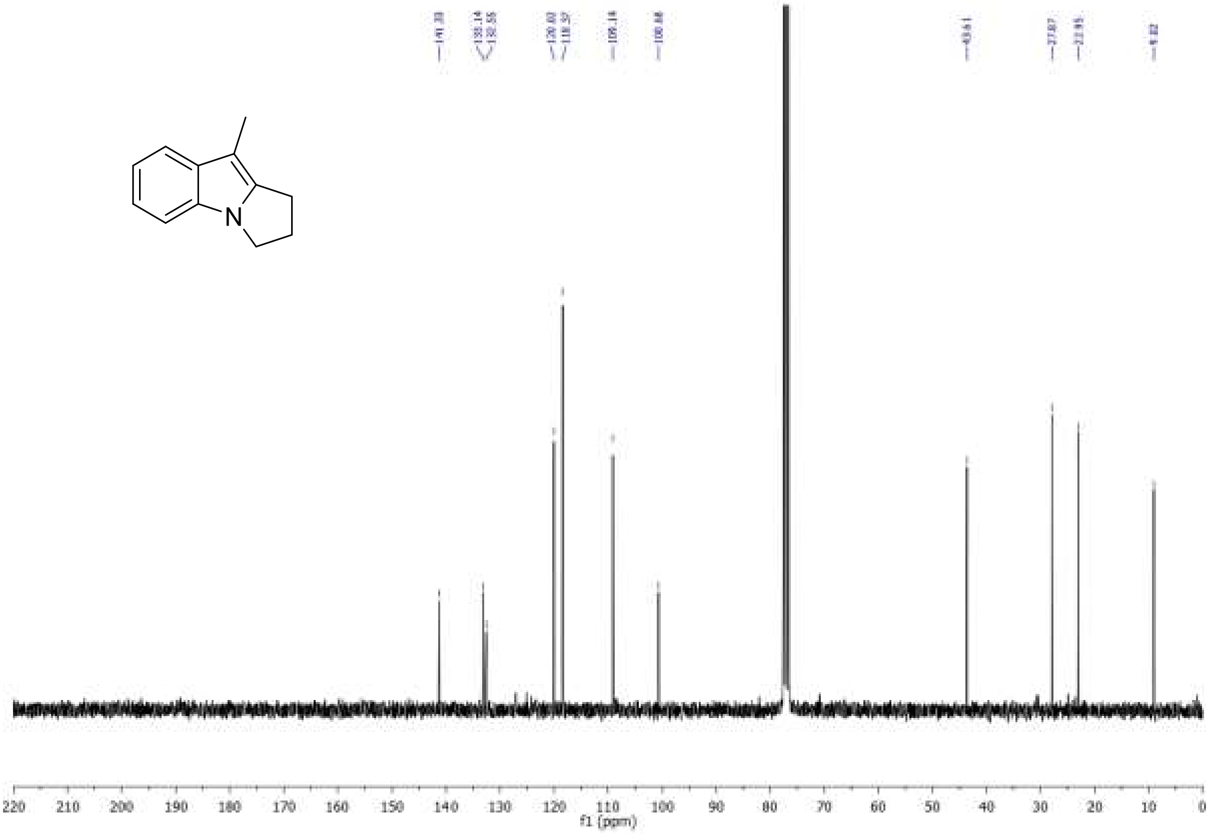
N
f1 (ppm)

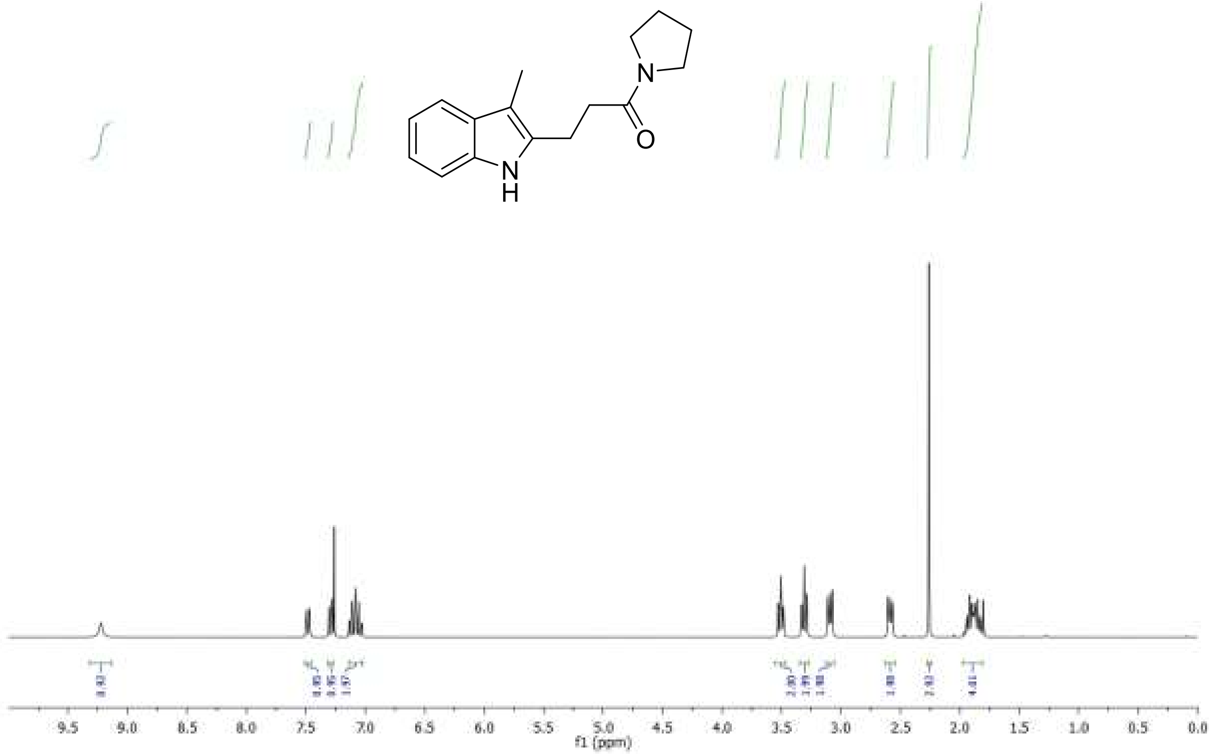
f1 (ppm)

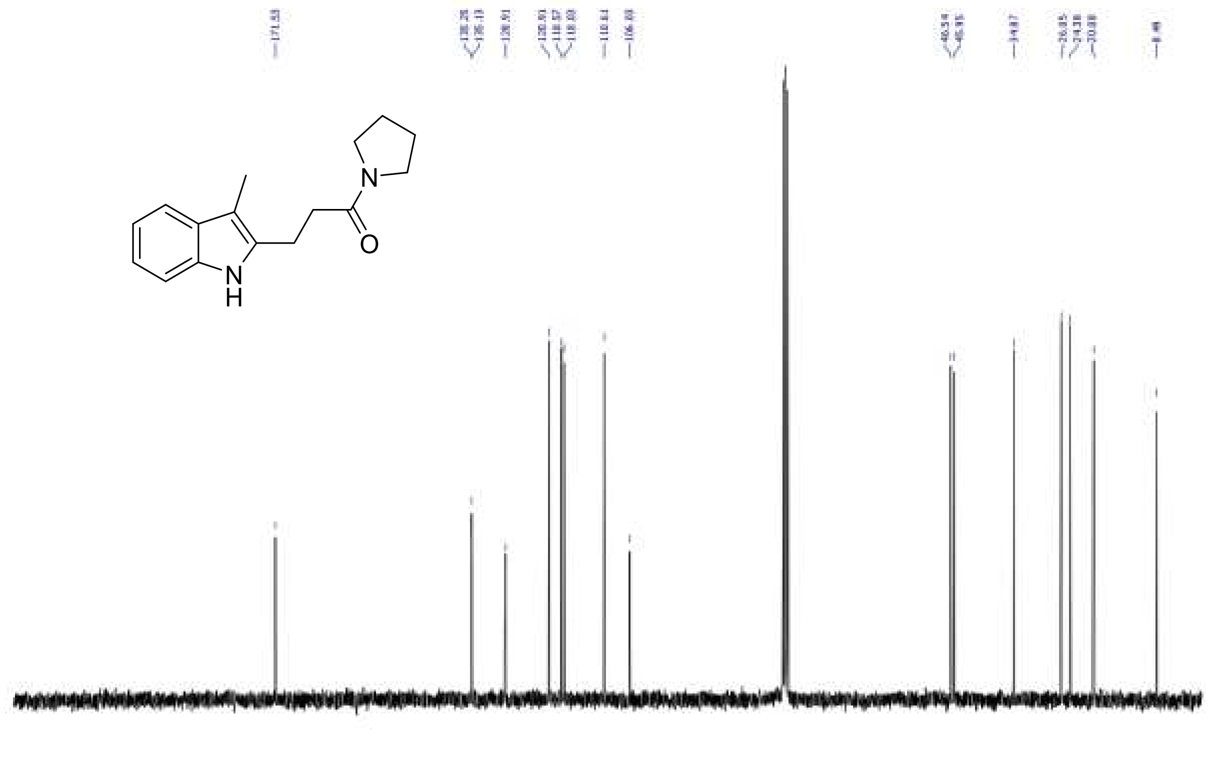
f1 (ppm)

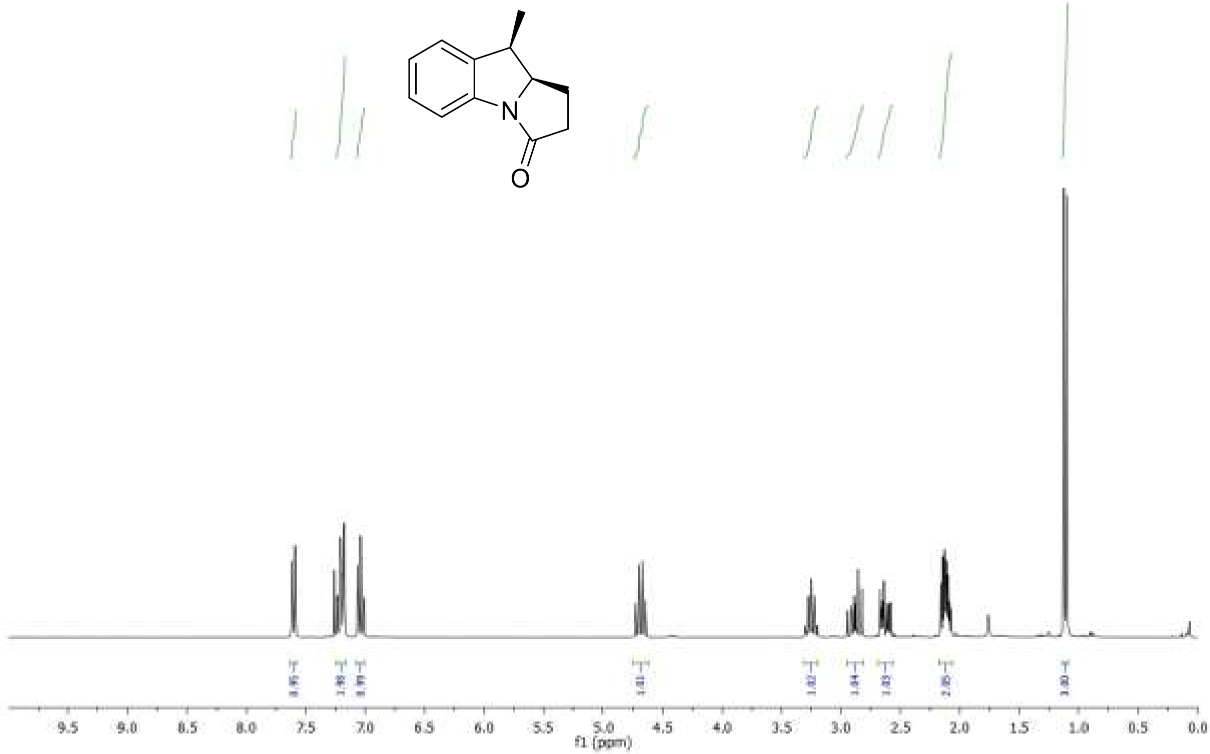
N
O
f1 (ppm)

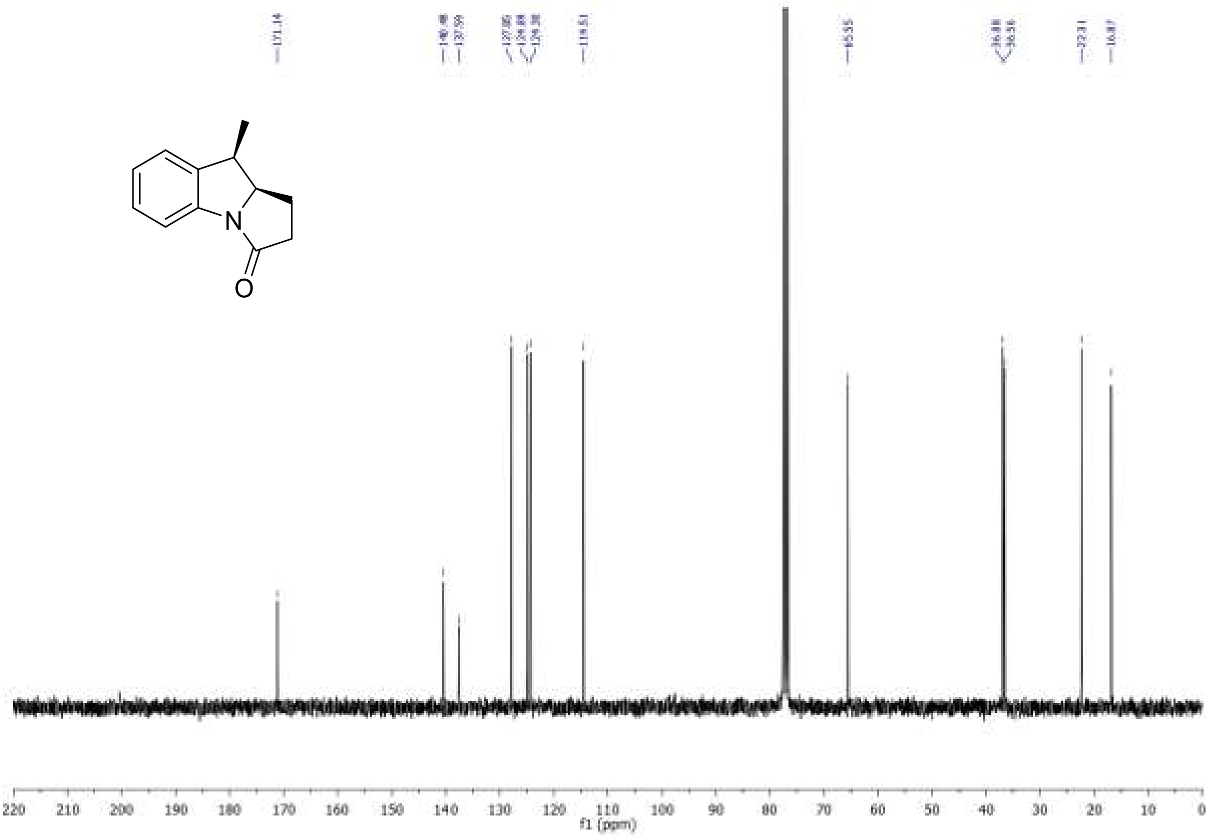
N
O
f1 (ppm)

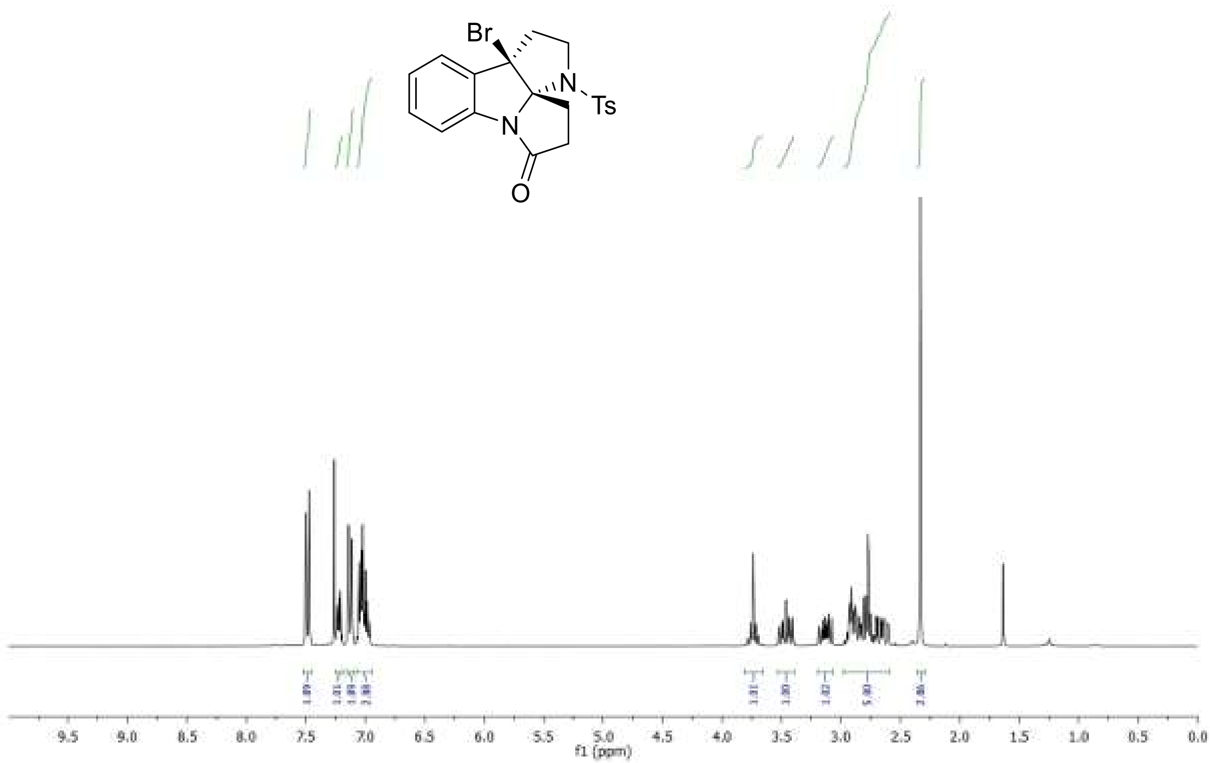

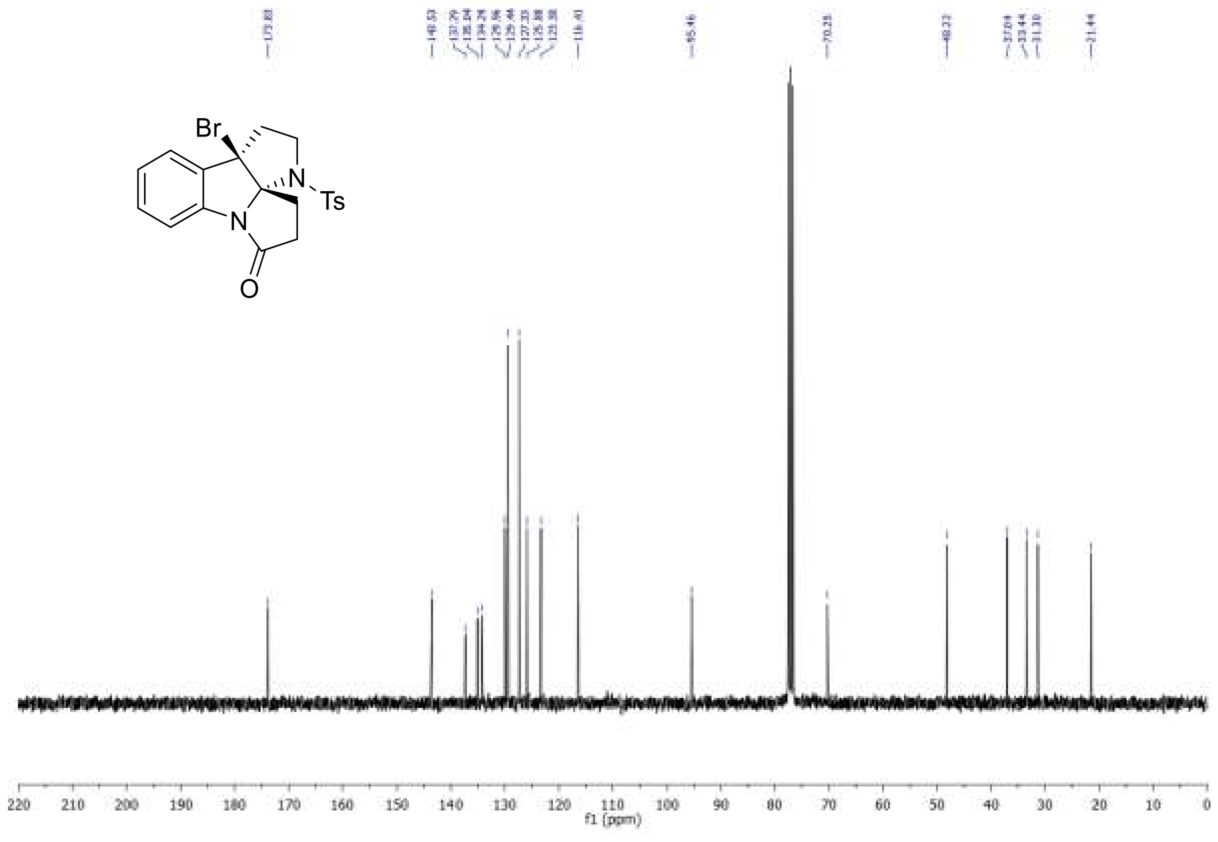

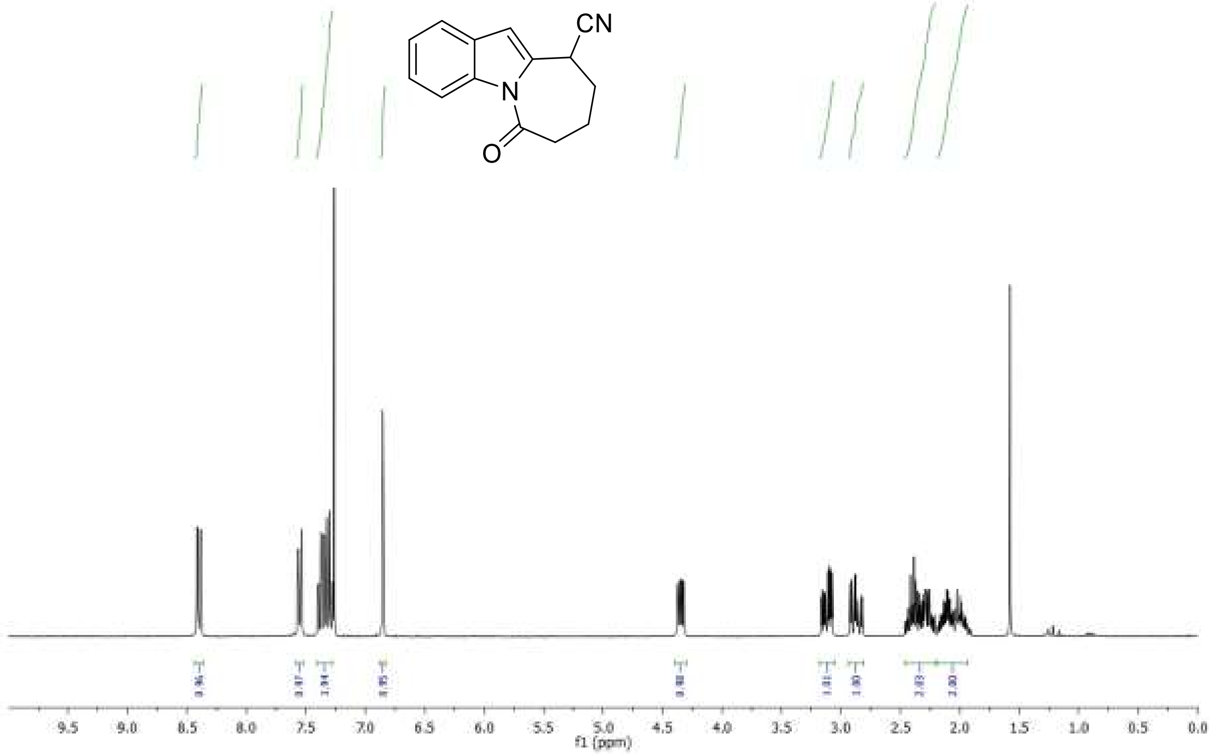
CN
N
O
f1 (ppm)

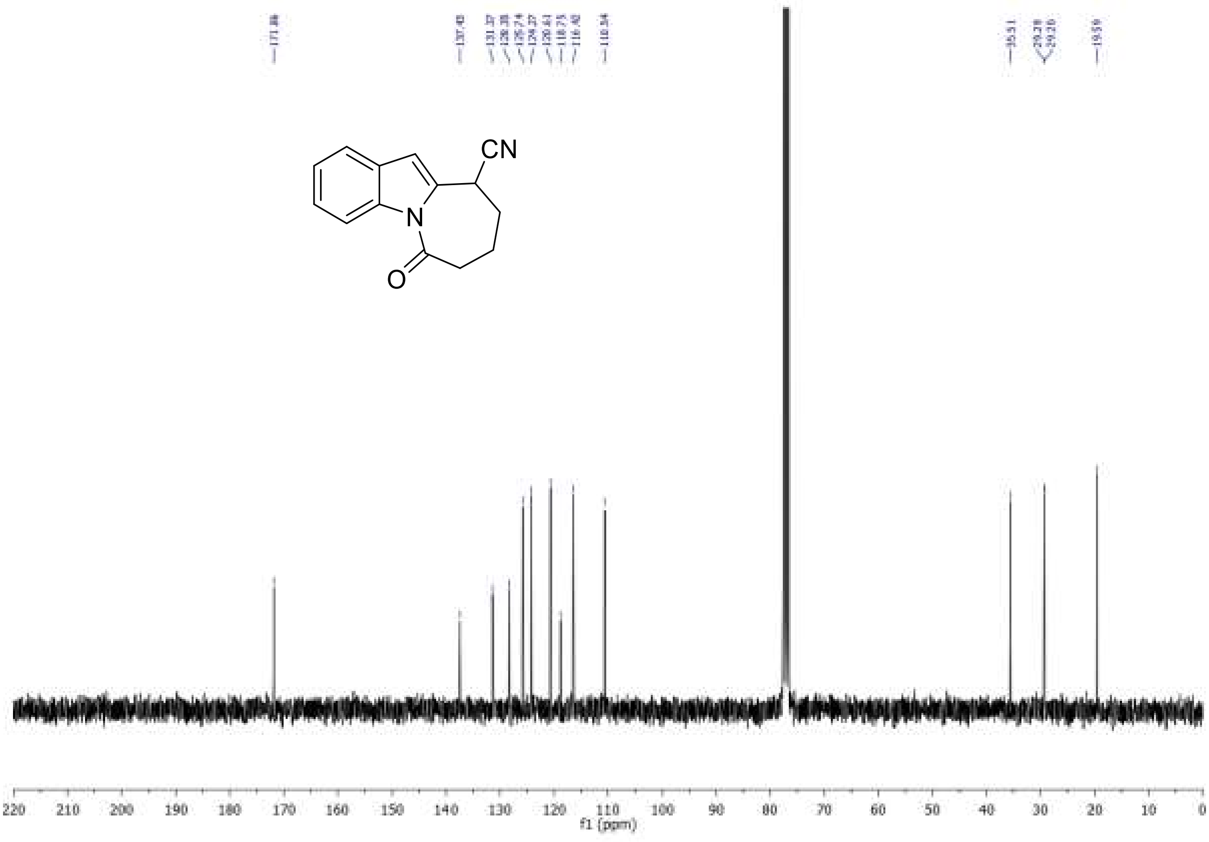
CN
N
O
f1 (ppm)

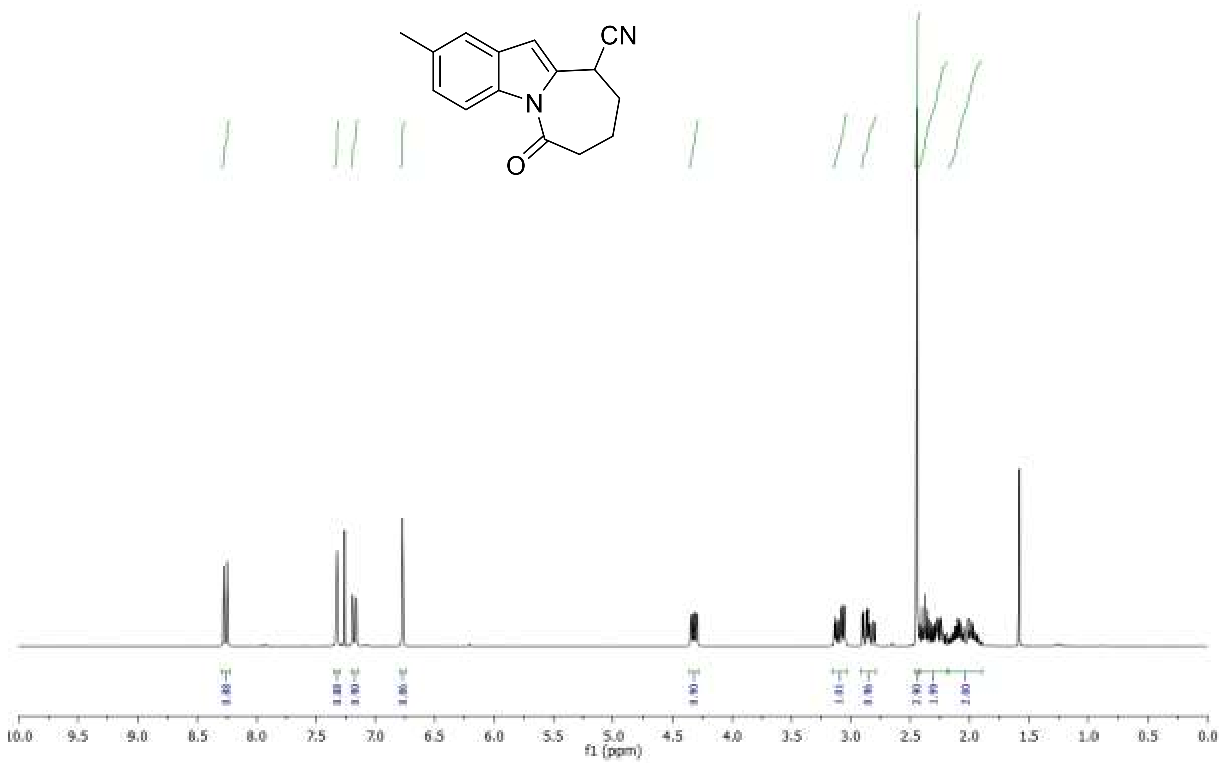

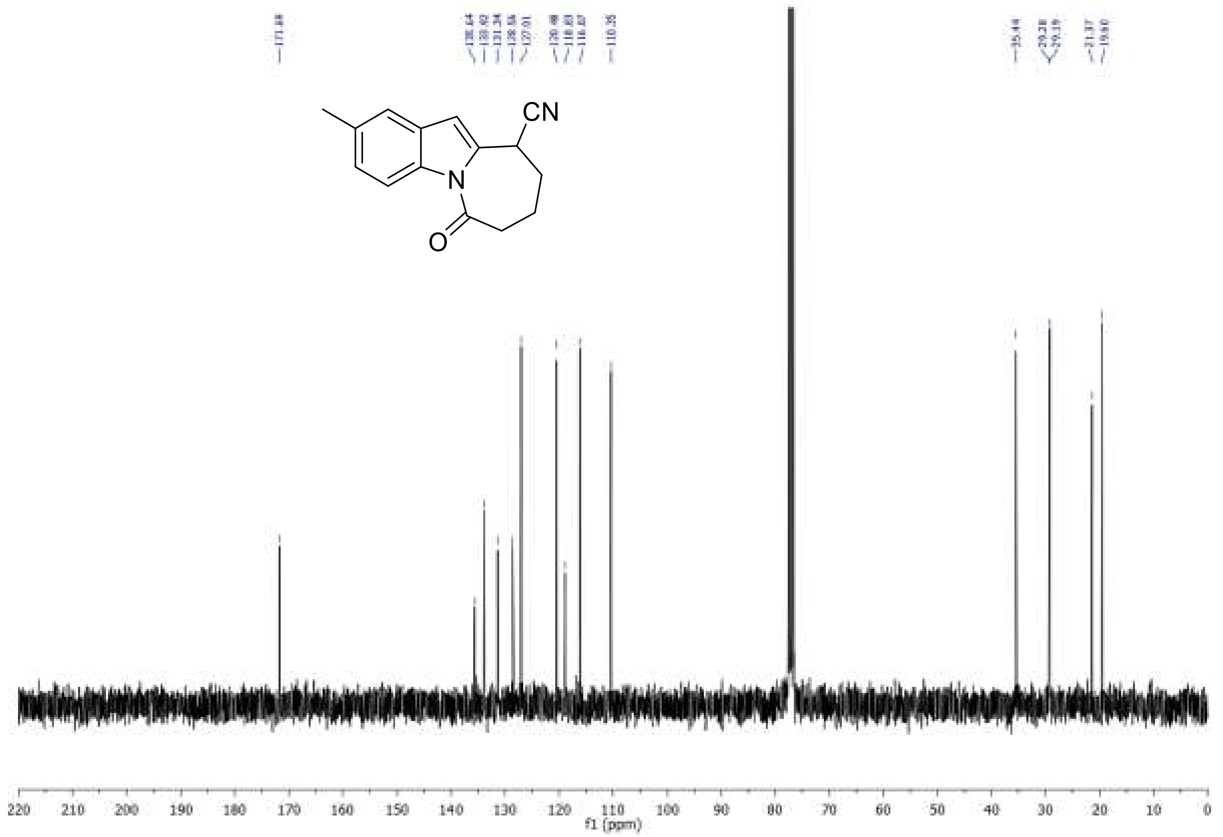

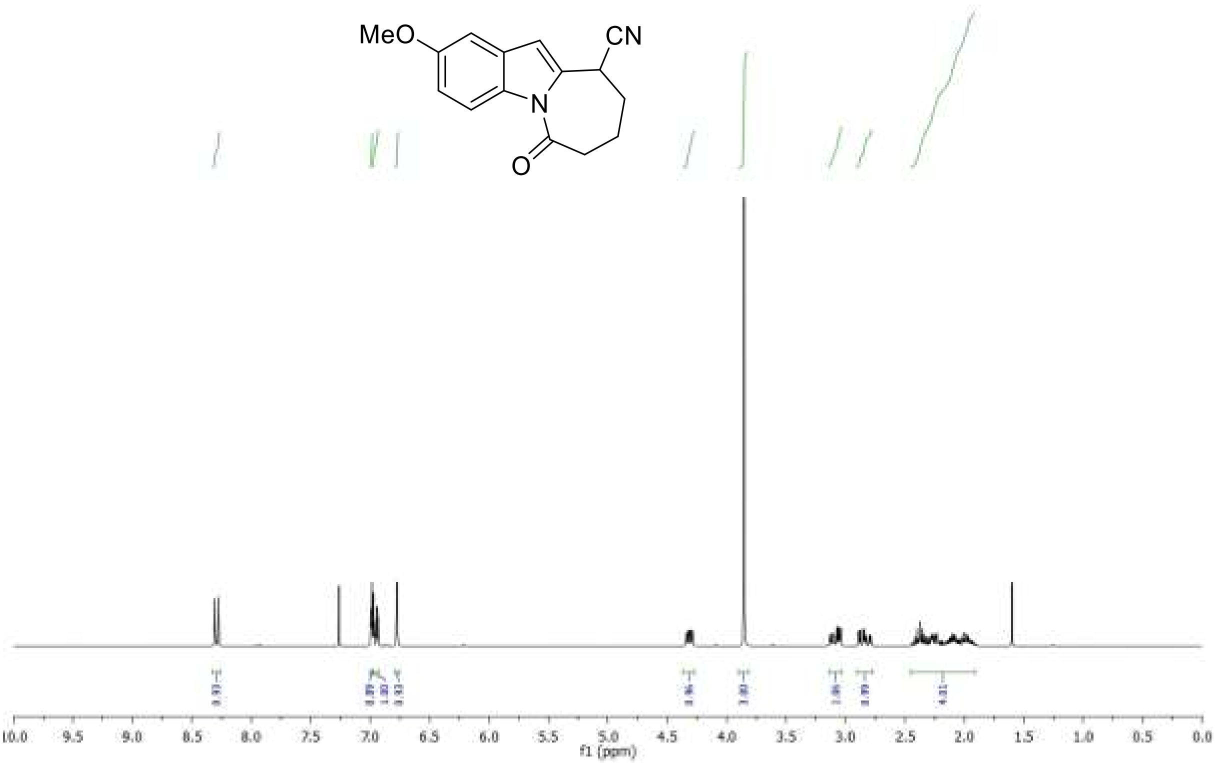
MeO
CN
N
O
f1 (ppm)

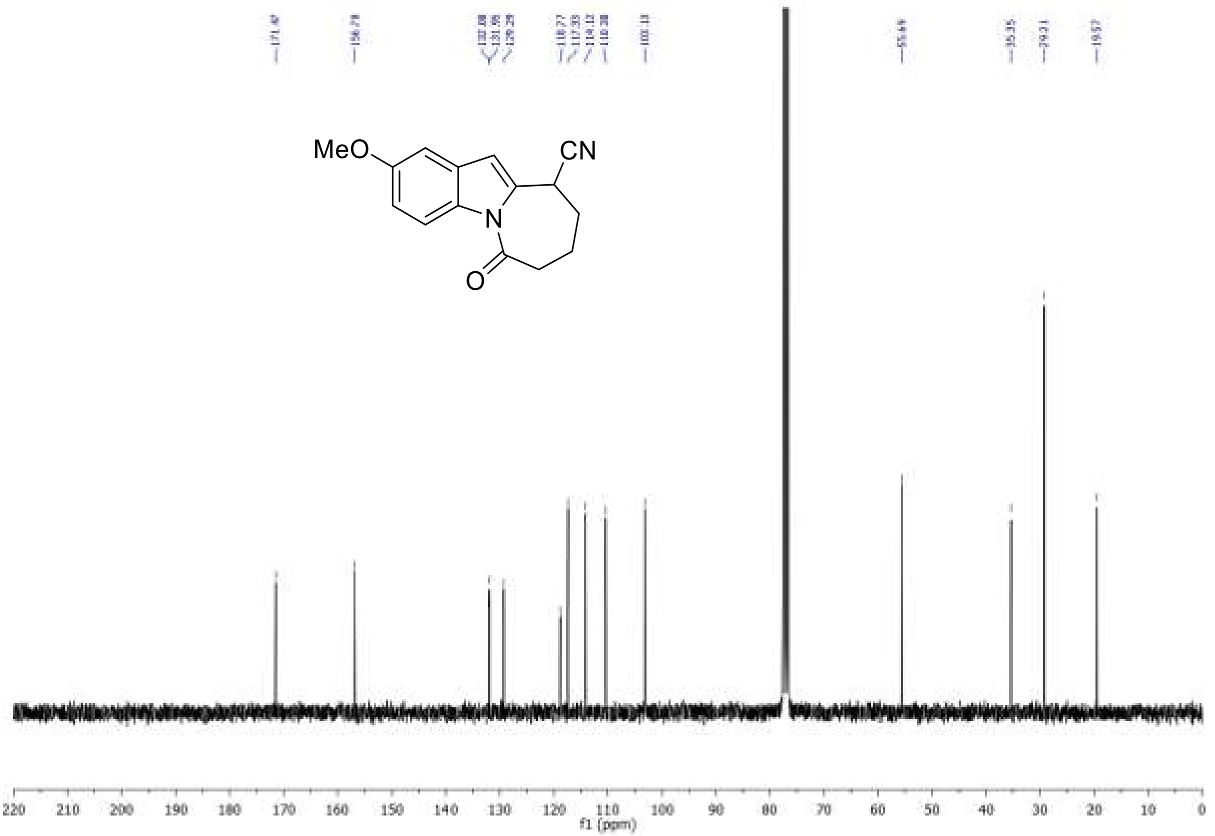
MeO
CN
N
O
f1 (ppm)

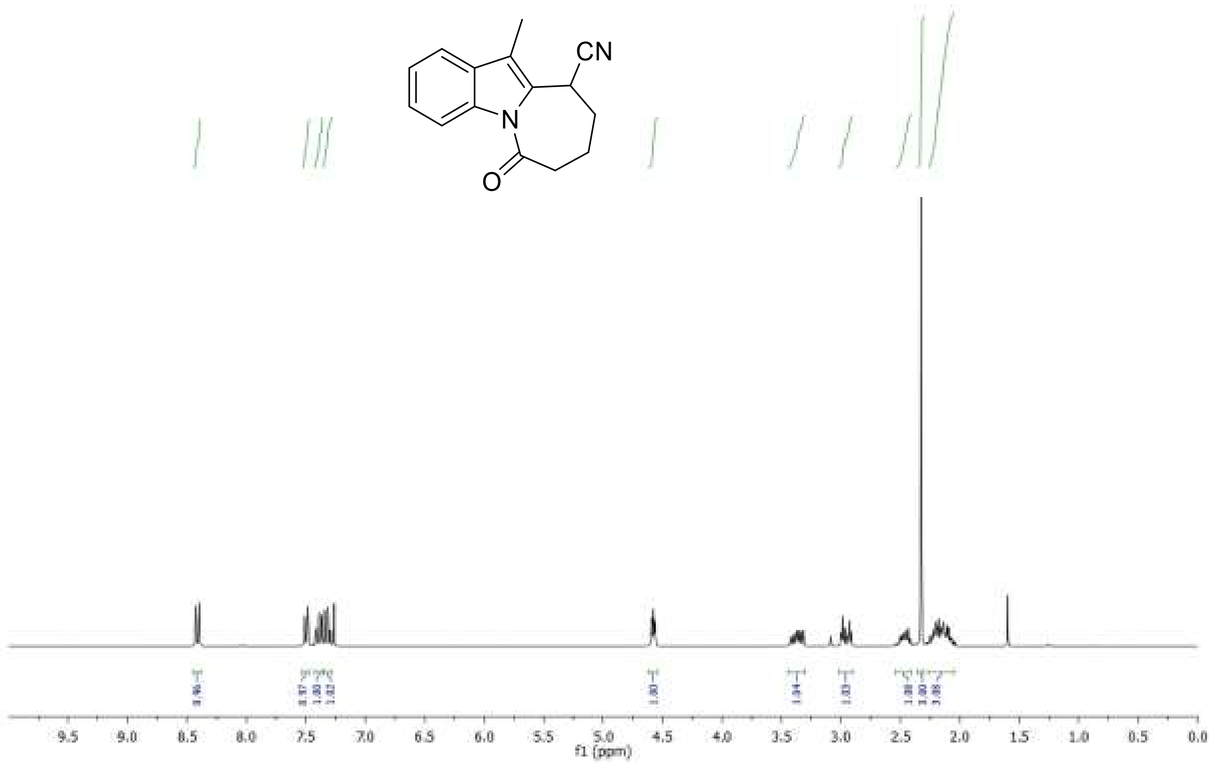
CN
N
O
f1 (ppm)

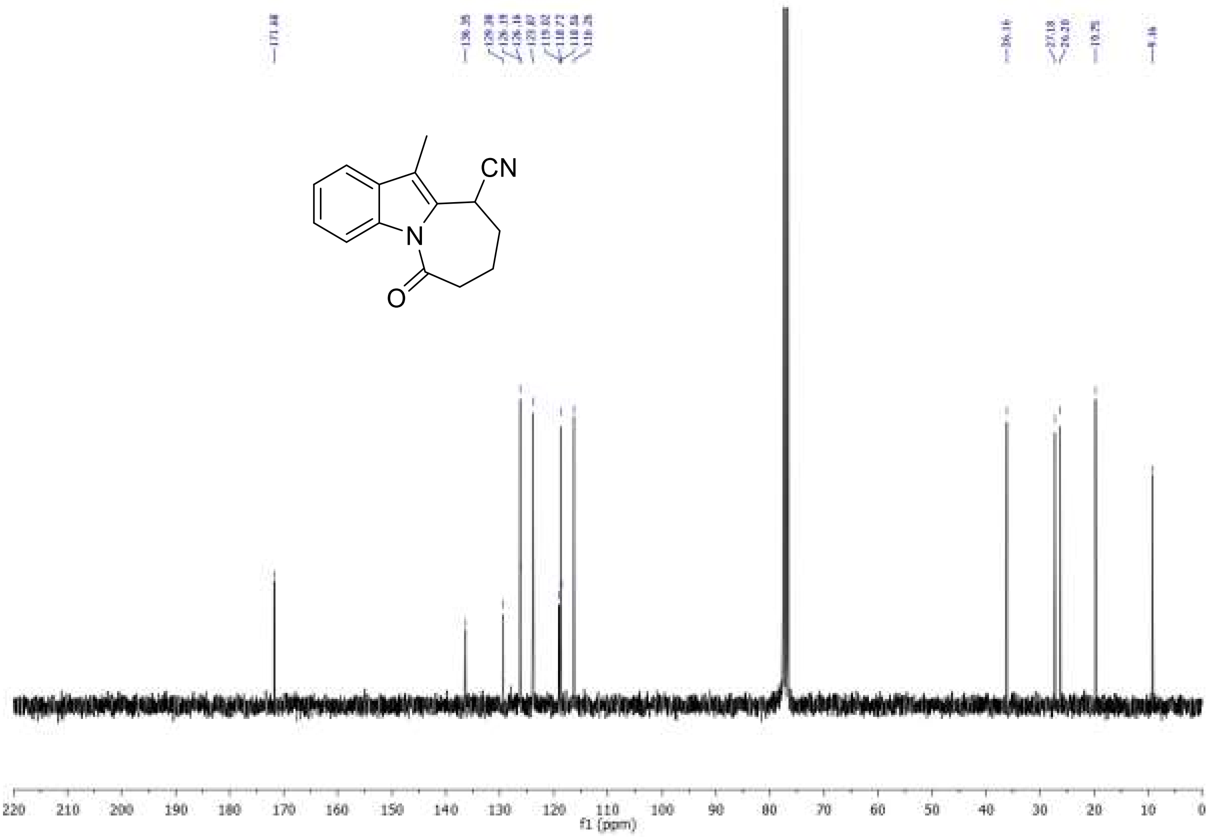
CN
N
O
f1 (ppm)

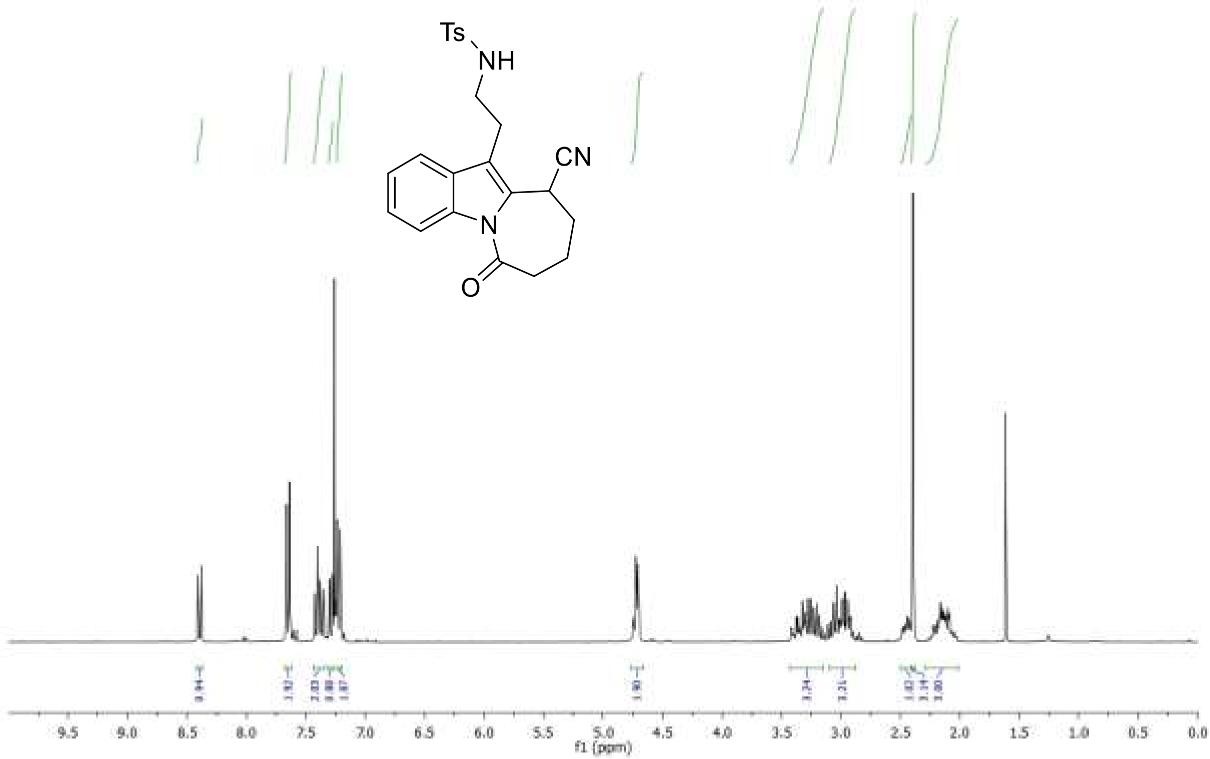
Ts
NH
CN
N
O
9.5 9.0 8.5 8.0 7.5 7.0 6.5 6.0 5.5 5.0 4.5 4.0 3.5 3.0 2.5 2.0 1.5 1.0 0.5 0.0
f1 (ppm)

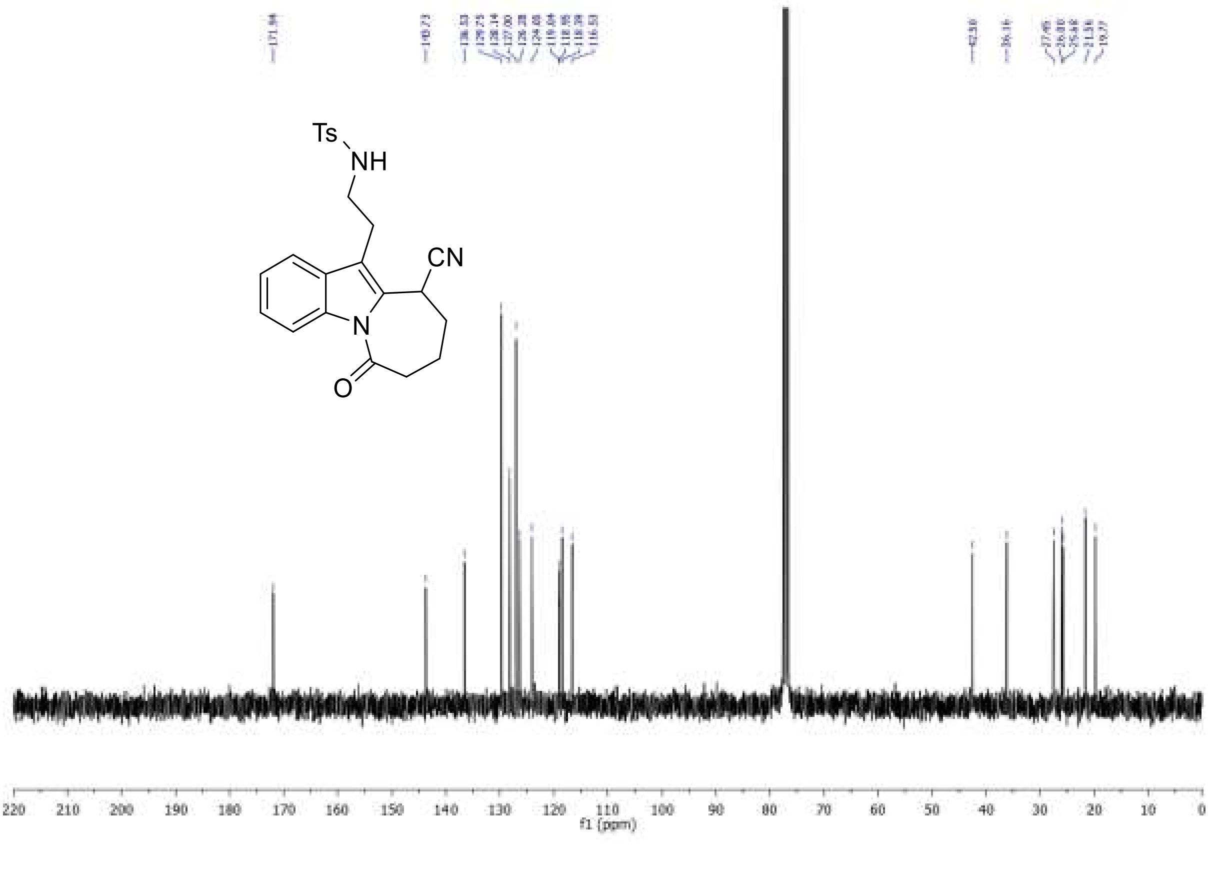
Ts
NH
CN
N
O
220 210 200 190 180 170 160 150 140 130 120 110 100 90 80 70 60 50 40 30 20 10 0
f1 (ppm)

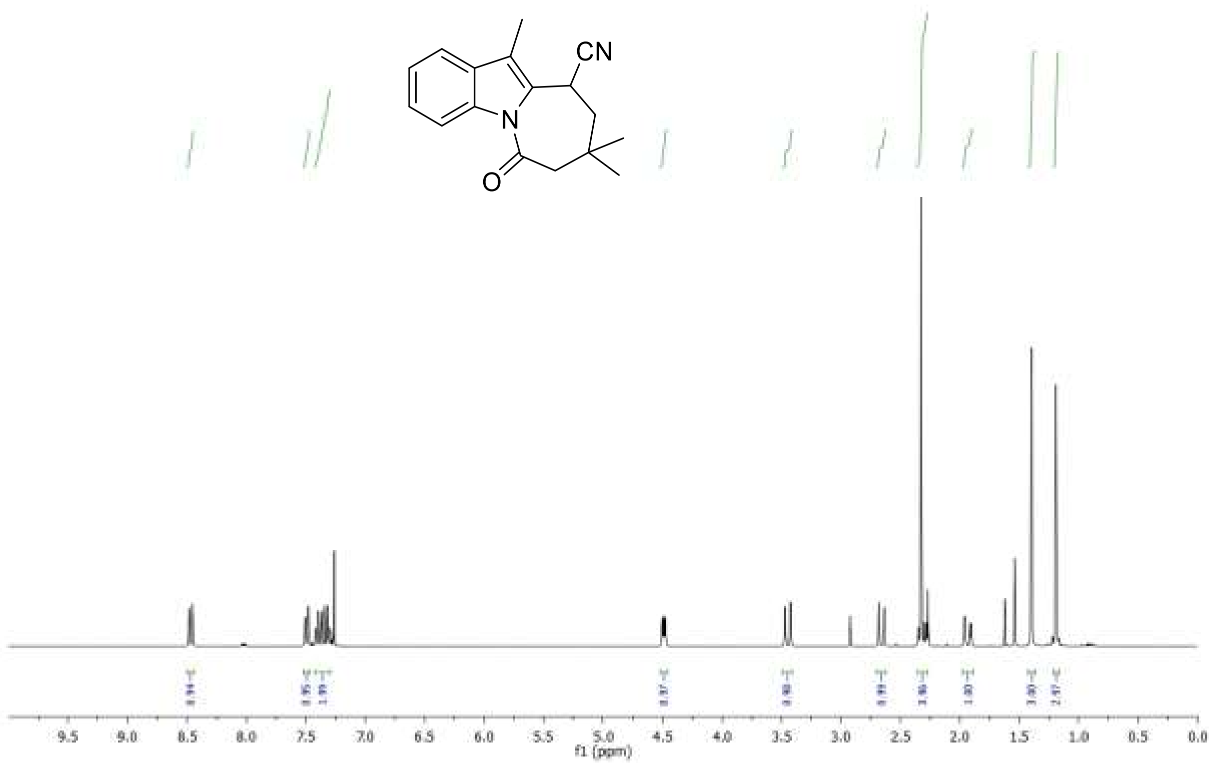
CN
N
O
9.5 9.0 8.5 8.0 7.5 7.0 6.5 6.0 5.5 5.0 4.5 4.0 3.5 3.0 2.5 2.0 1.5 1.0 0.5 0.0
f1 (ppm)

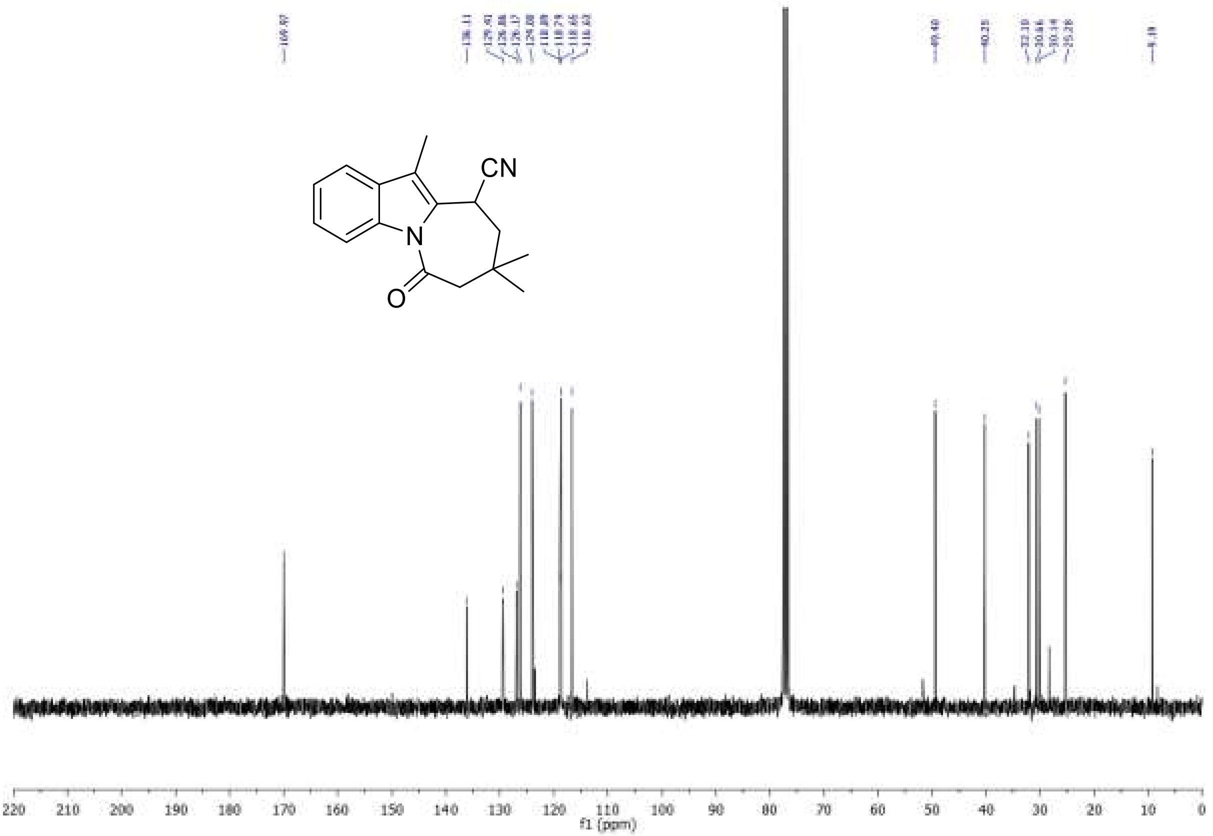
CN
N
O
220 210 200 190 180 170 160 150 140 130 120 110 100 90 80 70 60 50 40 30 20 10 0
f1 (ppm)
